# A Chemo- and Regioselective Tandem [3 + 2]Heteroannulation Strategy for Carbazole Synthesis: Combining Two Mechanistically Distinct Bond-Forming Processes

**DOI:** 10.1021/acs.joc.1c02943

**Published:** 2022-03-18

**Authors:** Emma Campbell, Andrea Taladriz-Sender, Olivia I. Paisley, Alan R. Kennedy, Jacob T. Bush, Glenn A. Burley

**Affiliations:** † Department of Pure Applied Chemistry, University of Strathclyde, Thomas Graham Building, 295 Cathedral Street, Glasgow G1 1XL, U.K.; ‡ GlaxoSmithKline, Medicines Research Centre, Gunnels Wood Road, Stevenage, Hertfordshire SG1 2NY, U.K.

## Abstract

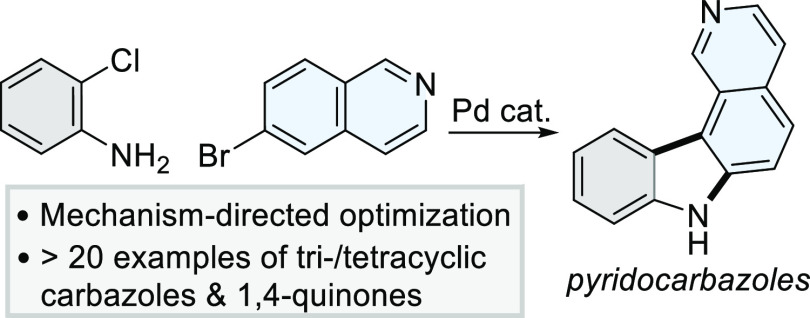

A modular approach to prepare tri- and tetracyclic carbazoles by a sequential [3 + 2]heteroannulation is described. First, optimization of Pd-catalyzed Buchwald–Hartwig amination followed by C/N-arylation in a one-pot process is established. Second, mechanistic analyses identified the origins of chemo- and regioselective sequential control of both bond-forming steps. Finally, the substrate scope is demonstrated by the preparation of a range of tri- and tetracyclic carbazoles, including expedient access to several natural products and anti-cancer agents.

## Introduction

**Figure 1:**
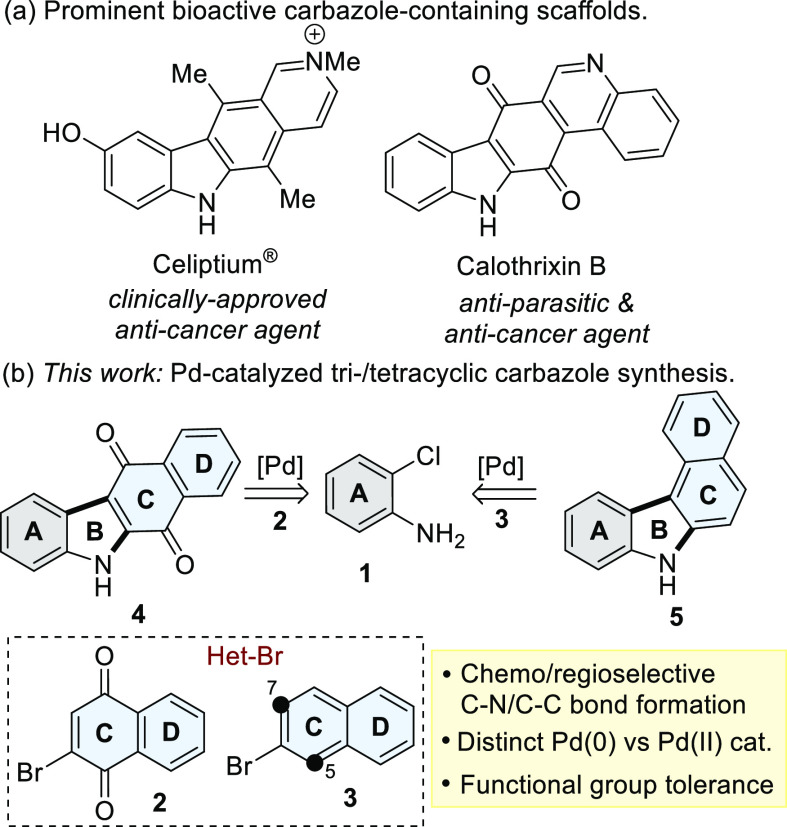
Generalized approach for the synthesis of tri–/tetracyclic carbazoles by Pd-catalyzed [3 + 2]heteroannulation.

Carbazoles are ubiquitous N-heterocycles used throughout medicinal chemistry and material sciences.^1−3^ From a pharmaceutical perspective, the carbazole core features extensively in drugs and natural products, many of which exhibit potent anti-proliferative activities (Figure 1a).^4−9^ The breadth of applications has inspired the development of numerous methodologies for their preparation.^2,9,10^ Pd-catalyzed processes in particular enable a [3 + 2]heteroannulation via Pd(0)-catalyzed Buchwald–Hartwig amination^11^ followed by Pd(II)-catalyzed *C*-arylation at a late stage in a synthetic workflow.^12−25^ However, a generalized set of guidelines that control the chemoselectivity of each C–N/C–C bond-forming reaction, and the factors that influence Pd(0)^23^ versus Pd(II)^17^ catalysis in a one-pot process have not been established. Furthermore, a mechanistic understanding of any regiocontrol underpinning the second C–H activation step for the formation of tetracyclic carbazoles is unknown.

In this manuscript, we establish reaction guidelines to prepare tri- and tetracyclic carbazoles by controlling the chemoselectivity of the first Pd(0)-catalyzed C–N bond-forming step and the regioselectivity of the second Pd(II)-catalyzed C/N-arylation (Figure 1b). Our rationale was to use *o-*chloroanilines (**1**) to define the A-ring of a carbazole core and heteroaryl bromides to form the C/D-rings of tricyclic and tetracyclic products. An initial version of this work was deposited in *ChemRxiv* on the 24th of November 2021.^26^

## Results and Discussion

The motivation for this work was to develop a robust, one-pot synthetic framework that can access both tri- and tetracyclic carbazoles. The synthesis of tetracyclic carbazoles using a heteroaryl bromide substrate such as **3** has potentially two competing C–H activation sites. If the *C-*arylation proceeded *via* a Pd(II)-catalyzed intermediate, then we surmised that the more nucleophilic 5-position of an isoquinoline will result in preferential C–H bond activation at this site.^27^ However, the mechanistic determinants that control the regioselectivity of such Pd(II)-catalyzed *C*-arylation are not known with respect to a convergent [3 + 2]heteroannulation approach.^28−31^

**Scheme 1:**
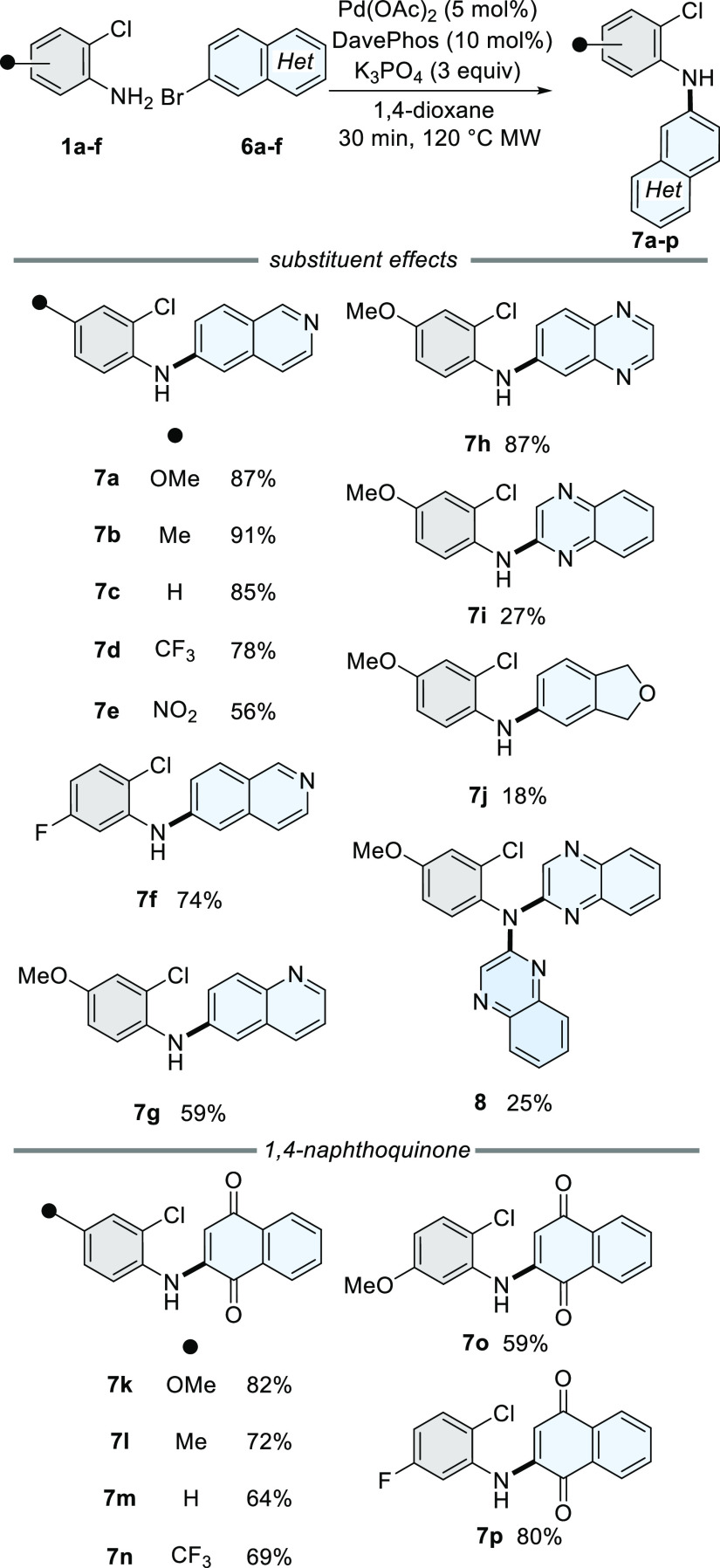
Scope (Isolated Yields) of Buchwald–Hartwig Amination

Our studies commenced with optimizing reaction conditions for the Pd(0)-catalyzed C–N bond-forming step using 4-methoxy-2-chloroaniline (**1a**) and 6-bromoisoquinoline (**6a**) as exemplar substrates (Scheme 1). An extensive screen (Table S1) identified DavePhos and K_3_PO_4_ as the optimal ligand/base pairing, which formed **7a** in 87% yield.

Using these conditions, secondary anilines **7b–e** (56–91%) were obtained using chloroanilines (**1b–e**) bearing electron-rich and electron-withdrawing substituents when reacted with 6-bromoisoquinoline (**6a**). The conditions tolerated a range of heteroaryl bromides to form **7g–j**, except for 2-bromoquinoxaline (**6d**). In this case, a mixture of secondary (**7i**, 27%) and tertiary anilines (**8**, 25%) was formed. Using 5-bromo-1,3-dihydroisobenzofuran (**6e**) formed **7j** in 18% yield. This process was also compatible with 2-bromonaphthalene-1,4-dione (**6f**) producing 1,4-napthoquinones^32^ (**7k–p**) in 59–82%. Whilst this reaction could conceivably proceed *via* Michael addition, the reaction afforded the secondary anilines **7k–p** in 5–7% in the absence of a Pd catalyst.

**Scheme 2:**
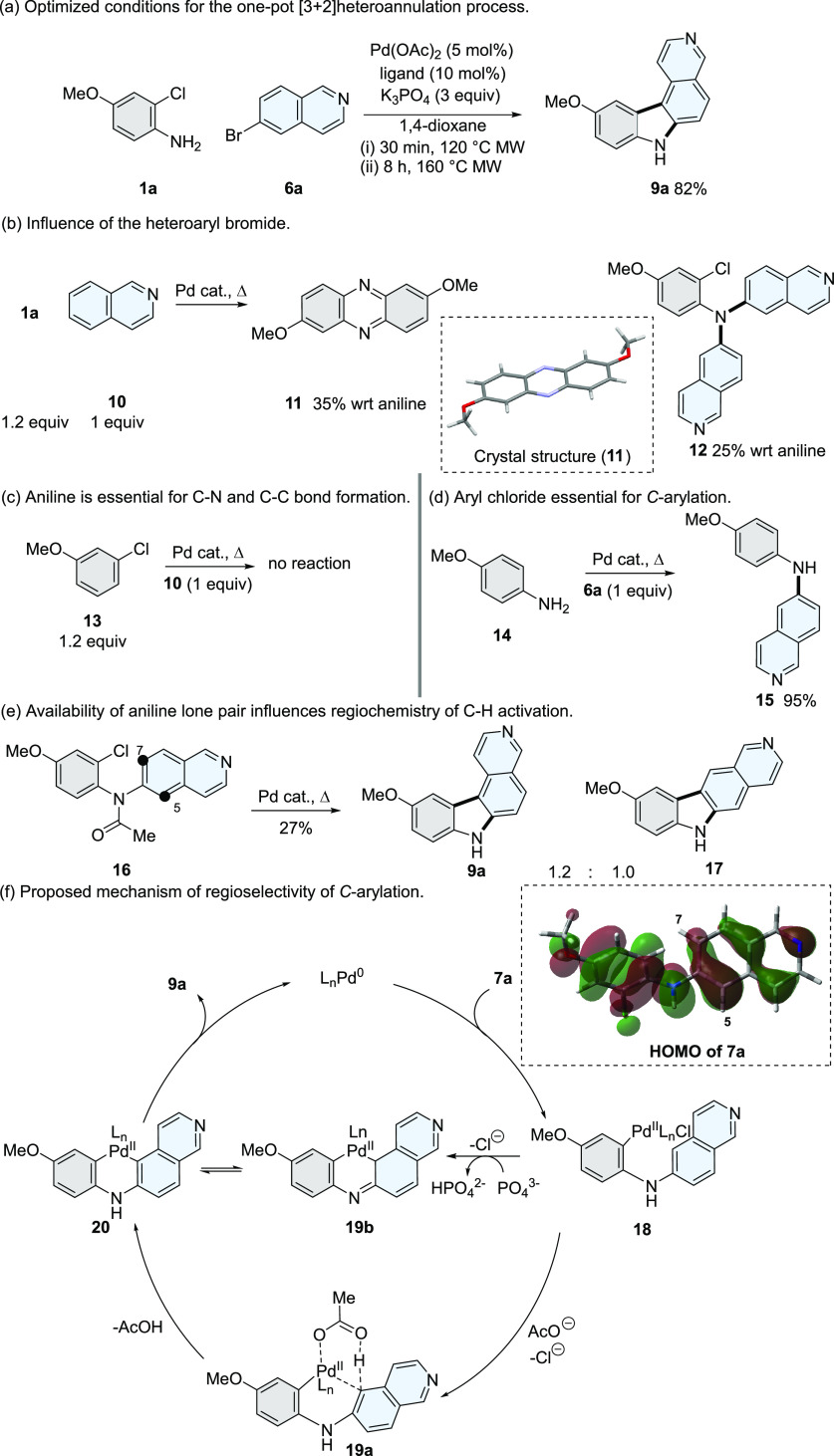
Mechanistic Studies of the [3 + 2]Heteroannulation Reaction conditions: Pd(OAc)_2_ (5 mol %), HPCy_3_BF_4_ (10 mol %), K3PO_4_ (3 equiv), 1,4-dioxane; (i) 30 min, 120 °C MW; (ii) 8 h, 160 °C MW; wrt = with respect to.

With conditions for the Pd(0)-catalyzed C–N bond-forming step established, the optimization of the one-pot process was explored (Table S2). The optimal ligand/base pairing of HPCy_3_BF_4_ with K_3_PO_4_ was identified, which formed **9a** from **1a** and **6a** in 82% yield (Scheme 2a). This highlights that the second C–H activation step is regioselective for the 7-position of **6a**. To further understand how the nature of the chloroaniline and heteroaryl bromide substrates influenced the chemo- and regioselectivity of both bond-forming steps, a series of test reactions was undertaken. Exchanging 6-bromoisoquinoline (**6a**) for isoquinoline (**10**) formed dimethoxyphenazine (**11**) and the tertiary aniline (**12**) in 35 and 25% isolated yields, respectively (Scheme 2b). No dihydrophenazine was isolated from the reaction, which suggests that an *in situ* oxidation occurred.^33^ No reaction occurred when isoquinoline (**10**) was the coupling partner with 1-chloro-3-methoxybenzene (**13**, Scheme 2c). Only secondary aniline (**15**) was isolated when *para*-anisidine (**14**) was reacted with 6-bromoisoquinoline (**6a**, Scheme 2d). Taken collectively, these test reactions highlighted the following requirements for the preparation of the tetracyclic core: (i) aryl bromide is essential and prevents dimerization of the *o-*chloroaniline, (ii) whilst the absence of a bromo substituent in the heteroaryl substrate results in C–H activation at the same site, there is little regiocontrol, (iii) a chloro substituent is essential for *C*-arylation.

The influence of the electron-donating aniline lone pair in the direct *C-*arylation step was then explored (Scheme 2e). We surmised that the acetylated substrate (**16**) would deactivate the C-ring and render the *C*-arylation less efficient. This indeed occurred as highlighted by the formation of deacetylated *C*-arylated regioisomers, **9a** and **17**, in 27% total yield (1.2:1.0, **9a**:**17**). We assume that deacetylation occurs *in situ* because of the high temperatures and the presence of a base in the reaction mixture. An unexpected result was the formation of the linear tetracyclic carbazole **17**, which arises from C–H activation of the isoquinoline 7-position of **16**. The formation of **17** suggests that C–H activation of the 7-position is favored if the acetyl group is present prior to *C*-arylation. In contrast, if **7a** is present, presumably formed by deactylation of **16**, C–H activation at the 5-position occurs. DFT calculations confirmed that the 5-position is indeed the more electron-rich site (Scheme 2e and the Supporting Information Section 6). We speculate that the acetyl group (*i.e.*, **16**) directs C–H activation at this site *via* coordination of a Pd(II) species through the amide carbonyl.^34−36^

This series of reactions has guided us to propose that oxidative addition of the C–Cl bond in **7a** occurs first and proceeds *via* a Pd(0) species to form **18** (Scheme 2f). Pd(II)-catalyzed *C-*arylation forms the palladacycle (**20**), which could proceed through a concerted metalation–deprotonation pathway *via* the formation of **19a**.^12^

**Scheme 3:**
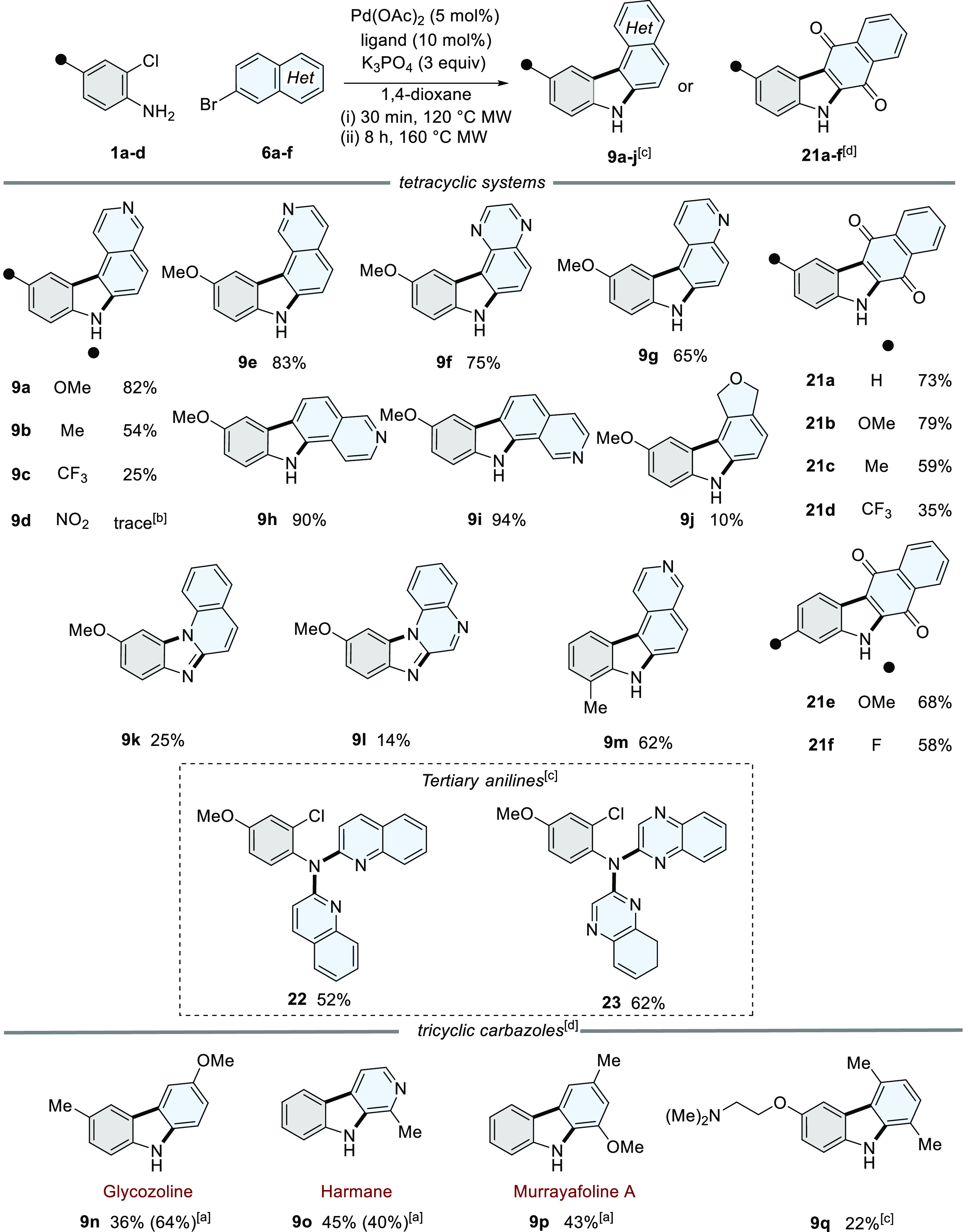
Scope and Isolated Yields of the [3 + 2]Heteroannulation^,^^,^^,^ Addition of PivOH (30 mol %). Formation of **7e** in 79%; traces of **9d** detected by LCMS. Ligand = HPtBu_3_BF_4_. Ligand = DavePhos.

Alternatively, since the efficiency of the reaction is higher with the lone pair of aniline (**18**) donating into the ring, a Friedel–Crafts-like electrophilic aromatic substitution mechanism proceeding *via* imine (**19b**) followed by tautomerization might also be possible to form **20**.^37^ Finally, reductive elimination of **20** produces the *C*-arylated product (**9a**). With mechanistic knowledge of the second C–C bond-forming step established, the substrate scope of the process was investigated (Scheme 3).

The reaction conditions formed 7*H*-pyrido[3,4-*c*]carbazole analogues (**9a–c**). The presence of a nitro group resulted in only trace amounts of **9d** formed, with the secondary aniline (**7e**) isolated in 79% yield. The reaction conditions also tolerated heteroatom changes and saturation in the D-ring of the heteroaryl bromide (**9e–j**).^30^ The [3 + 2]heteroannulation strategy was also compatible with the formation of carbazole-1,4-quinones (**21a–f**).^28,38^ Access to *N*-arylated products is also possible, forming a mixture of fused imidazoles (**9k–l**) *via* an *N-*arylation step, alongside tertiary anilines (**22–23**). Steric bulk *ortho* to the aniline substrate is also tolerated, forming **9m** in 62%. However, when 3-methyl-2-chloroaniline is used as a substrate, a mixture of regioisomers was formed as an inseparable mixture (see Supporting Information).

The modularity of this strategy is also exemplified by the preparation of natural products glycozoline (**9n**),^39^ harmane (**9o**),^40^ and murrayafoline A (**9p**).^41,42^ In addition, preparation of **9q** demonstrates that the reaction conditions tolerate functional groups bearing potential Pd-chelating sites and bulky substituents *ortho* to the corresponding aryl bromide.

**Scheme 4:**
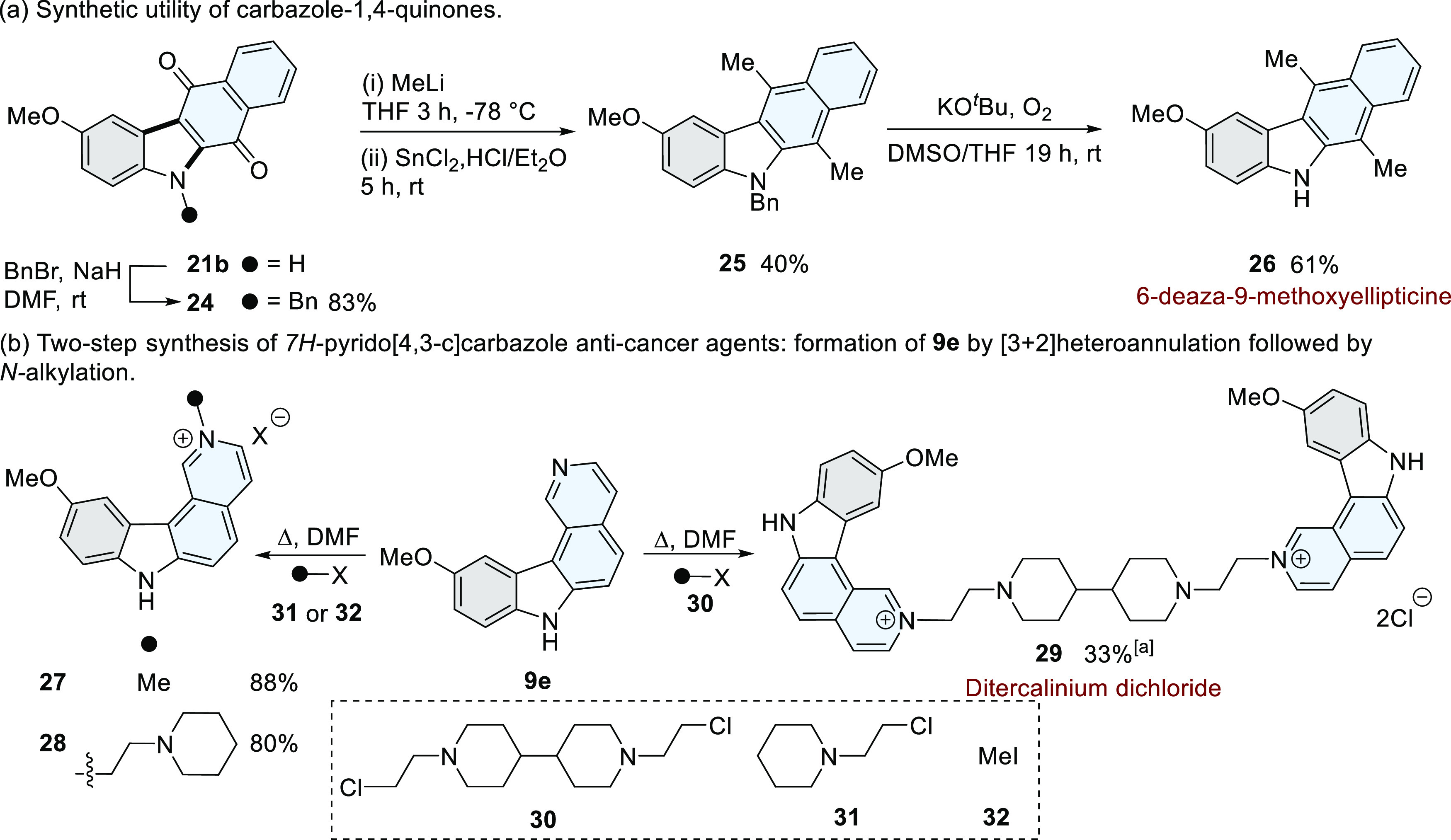
Application of the [3 + 2]Heteroannulation Process for the Targeted Synthesis of Biologically Active Tetracyclic Carbazoles. Yield calculated from the alkylation of **9e**; X = iodide for **27** and chloride for **28**.

Our [3 + 2]heteroannulation strategy was extended to the targeted synthesis of biologically active tetracyclic carbazoles. Carbazole-1,4-quinones (*e.g*., **21a–f**) have established anti-cancer activity *via* topoisomerase inhibition or by the production of reactive oxygen species.^38,43^ We used **21b** as a key intermediate for the targeted synthesis of a deaza analogue of the natural product 9-methoxyellipticine (Scheme 4a), producing **26** in three steps and in an overall yield of 20%.^44^

Further exemplification of our strategy was demonstrated by the preparation of alkylated 7*H*-pyridocarbazoles (*e.g*., **9a–e**), which have well-established anti-cancer activity.^45,46^ Previous preparative methods of this series of compounds have involved a six-step linear synthesis affording compound **9e** in 28% overall yield.^47^ Our two-step convergent strategy accessed the 7*H*-pyrido[4,3-*c*]carbazole core **9e** in a single step (83%), followed by alkylation (**30–32**) to produce mono-*N*-alkylated examples (**27–28**) and the potent anti-cancer agent ditercalinium dichloride (**29**, Scheme 4b).^48^

## Conclusions

In summary, we have established a mechanistic framework for the preparation of fused tetracyclic carbazoles. The key to the modularity of this [3 + 2]heteroannulation approach is knowledge of the molecular hallmarks that underpin both the chemo- and regioselectivity of the process. The strategy is amenable for the diversification of tri- and tetracyclic carbazoles and is a scalable method for target-focused synthesis of tetracyclic carbazoles. We envisage that this convergent approach could find application in medicinal chemistry for structure–activity profiling and in broader synthetic applications that require step-efficient access to carbazole scaffolds.

## Experimental Section

### General Information

All reagents and solvents were obtained from commercial suppliers and were used without further purification unless otherwise stated. Purification was carried out according to standard laboratory methods. Starting materials were purchased from commercial suppliers and used without further purification unless otherwise stated. Dry solvents for reactions were purchased from Sigma-Aldrich and stored under nitrogen. Dichloromethane, chloroform, methanol, ethyl acetate, and petroleum ether (40–60 °C) for purification purposes were used as obtained from suppliers, without further purification. Reactions were carried out using conventional glassware for the preparation of starting materials. Microwave reactions were carried out in capped 2–5 mL microwave vials purchased from Biotage. Microwave reactions were carried out at elevated temperatures using a Biotage Initiator+ equipped with a Robot Eight microwave system. Thin-layer chromatography was carried out using Merck silica plates coated with a fluorescent indicator UV254, and they were analyzed under both 254 and 375 nm UV light or developed using potassium permanganate solution. Normal phase flash chromatography was carried out using 60 Å, 40–63 μm silica gel from Fluorochem. Semi-preparative reversed-phase HPLC purification was carried out on a Kinetex 5 μm, 150 × 21.2 mm XB C18 using a DIONEX 3000 series HPLC system equipped with a VWD3400 variable wavelength detector. Preparative purifications of small molecules were performed using a 30–90% gradient B (solvent A: 0.1% TFA in water, solvent B: 0.1% TFA in acetonitrile), with a flow rate of 12.0 mL/min. The absorbance of the UV-active material was detected at 254 nm. Analytical reversed-phase HPLC (RP-HPLC) was carried out on a Shimadzu Prominence instrument equipped with a PDA detector scanning from 190 to 600 nm using a Thermofisher Hypersil GOLD column 100 × 4.6 mm, with a particle size of 5 μm. The Fourier transform infrared (FTIR) spectra were obtained on a Shimadzu IR Affinity-1 instrument. Only major absorbance bands are reported. The ^1^H NMR, ^13^C NMR, and ^19^F NMR spectra were obtained on a Bruker AV 400 at 400, 101, and 376 MHz, respectively. Chemical shifts are reported in parts per million (ppm), and coupling constants are reported in hertz with DMSO-*d*_6_ referenced at 2.50 (^1^H) and 39.52 ppm (^13^C) and MeOD-*d*_4_ referenced at 3.31 (^1^H) and 49.0 ppm (^13^C). Assignment of ^13^C NMR signals is based on HSQC and HMBC experiments. The COSY and NOESY spectra were used to assign unequivocally atom connectivities. The high-resolution mass spectra were recorded on a Bruker microTOF II mass spectrometer at the SIRCAMs facility at the University of Edinburgh or on an LTQ Orbitrap xL at the EPSRC National Facility in Swansea.

### General Experimental Procedure for Microwave-Assisted Buchwald–Hartwig Amination

Aryl bromide (0.50 mmol, 1.00 equiv), chloroaniline (0.60 mmol, 1.2 equiv), Pd(OAc)_2_ (5 mol %), DavePhos (10 mol %), and K_3_PO_4_ (1.50 mmol, 3 equiv) were added to a microwave vial (2–5 mL). 1,4-Dioxane (5 mL, 0.1 M) was added and the vial was capped, evacuated and purged with argon three times, and then heated at 120 °C for 30 min under microwave irradiation in a Biotage microwave. The reaction was allowed to cool to rt, diluted with ethyl acetate (50 mL), and the solid was filtered under vacuum. The organic phase was washed with water and brine, dried with Na_2_SO_4_, filtered, and the solvent was removed *in vacuo*. The crude compound was purified by silica column chromatography.

### *N*-(2-Chloro-4-methoxyphenyl)isoquinolin-6-amine (**7a**)

The reaction was carried out according to the general procedure using 6-bromoisoquinoline (104 mg, 0.50 mmol, 1.0 equiv) and 4-methoxy-2-chloroaniline (95 mg, 0.60 mmol, 1.2 equiv) as starting materials. The crude residue was purified by silica gel column chromatography, petroleum ether (40–60 °C):EtOAc (7: 3), to obtain compound **7a** in 87% yield (123 mg, brown solid).

^1^H NMR ((CD_3_)_2_SO, 400 MHz): δ 8.95 (s, 1H, *H*^1^), 8.29 (s, 1H, N*H*), 8.20 (d, 1H, *J* = 4.6 Hz, *H*^3^), 7.87 (d, 1H, *J* = 7.1 Hz, *H*^8^), 7.41–7.38 (m, 2H, *H*^6′,4^), 7.24 (dd, 1H, *J* = 7.1, 1.7 Hz, *H*^7^), 7.18 (d, 1H, *J* = 2.3 Hz, *H*^3′^), 7.00 (dd, 1H, *J* = 7.0, 2.3 Hz, *H*^5′^), 6.67 (d, 1H, *J* = 1.7 Hz, *H*^5^), 3.81 (s, 3H, −OC*H_3_*). ^13^C{^1^H} NMR ((CD_3_)_2_SO, 101 MHz): δ 157.1 (*C*^4′^), 151.0 (*C*^1^), 147.9 (*C*^6^), 143.1 (*C*^3^), 137.3 (*C*^4a^), 130.6 (*C*^2′^), 130.3 (*C*^1′^), 128.8 (*C*^8^), 127.9 (*C*^6′^), 122.9 (*C*^8a^), 119.2 (*C*^7^), 118.6 (*C*^4^), 115.2 (*C*^3′^), 114.3 (*C*^5′^), 103.0 (*C*^5^), 55.7 (−O*C*H_3_).

IR *v̅*_max_ (cm^–1^): 3230 (N–H stretch), 1618 (C=N stretch), 1476 (C=C stretch), 1284 (C–N stretch), 1212 (C–O stretch), 1039 (C–O stretch), 847 (C–Cl stretch).

HRMS (ESI) *m*/*z*: [M + H]^+^ calcd for C_16_H_14_O_1_N_2_Cl, 285.0789; found, 285.0787.

### *N*-(2-Chloro-4-methylphenyl)isoquinolin-6-amine (**7b**)

The reaction was carried out according to the general procedure using 6-bromoisoquinoline (104 mg, 0.50 mmol, 1.0 equiv) and 4-methyl-2-chloroaniline (85 mg, 0.60 mmol, 1.2 equiv) as starting materials. The crude residue was purified by silica gel column chromatography, petroleum ether (40–60 °C):EtOAc (6:4), to obtain compound **7b** in 91% yield (122 mg, brown solid).

^1^H NMR ((CD_3_)_2_SO, 400 MHz): δ 8.97 (s, 1H, *H*^1^), 8.35 (s, 1H, N*H*), 8.23 (d, 1H, *J* = 5.8 Hz, *H*^3^), 7.89 (d, 1H, *J* = 8.9 Hz, *H*^8^), 7.45 (d, 1H, *J* = 5.8 Hz, *H*^4^), 7.40 (d, 1H, *J* = 1.4 Hz, *H*^3′^), 7.38 (d, 1H, *J* = 8.1 Hz, *H*^6′^), 7.32 (dd, 1H, *J* = 8.9, 2.2 Hz, *H*^7^), 7.19 (dd, 1H, *J* = 8.1, 1.5 Hz, *H*^5′^), 6.90 (d, 1H, *J* = 2.1 Hz, *H*^5^), 2.32 (s, 3H, −C*H_3_*). ^13^C{^1^H} NMR ((CD_3_)_2_SO, 101 MHz): 151.1 (*C*^1^), 146.8 (*C*^6^), 143.1 (*C*^3^), 137.1 (*C*^4a^), 135.3 (*C*^2′^), 135.1 (*C*^1′^), 130.5 (*C*^3′^), 128.8 (*C*^8^), 128.7 (*C*^5′^), 127.5 (*C*^4′^), 124.9 (*C*^6′^), 123.2 (*C*^8a^), 119.8 (*C*^7^), 118.8 (*C*^4^), 104.3 (*C*^5^), 20.1 (−*C*H_3_).

IR *v̅*_max_ (cm^–1^): 3234 (N–H stretch), 3031 (C–H stretch), 1608 (C=N stretch), 1474 (C=C stretch), 821 (C–Cl stretch).

HRMS (ESI) *m*/*z*: [M + H]^+^ calcd for C_16_H_14_N_2_Cl, 269.0840; found, 269.0841.

### *N*-(2-Chlorophenyl)isoquinolin-6-amine (**7c**)

The reaction was carried out according to the general procedure using 6-bromoisoquinoline (104 mg, 0.50 mmol, 1.0 equiv) and 2-chloroaniline (77 mg, 0.60 mmol, 1.2 equiv) as starting materials. The crude residue was purified by silica gel column chromatography, petroleum ether (40–60 °C):EtOAc (1:1), to obtain compound **7c** in 85% yield (108 mg, green solid).

^1^H NMR ((CD_3_)_2_SO, 400 MHz): δ 9.00 (s, 1H, *H*^1^), 8.43 (s, 1H, N*H*), 8.26 (d, 1H, *J* = 5.8 Hz, *H*^3^), 7.93 (d, 1H, *J* = 8.9 Hz, *H*^8^), 7.55 (dd, 1H, *J* = 8.0, 1.4 Hz, *H*^3′^), 7.51 (dd, 1H, *J* = 8.0, 1.4 Hz, *H*^7^), 7.49–7.47 (m, 1H, *H*^6′^), 7.41–7.33 (m, 2H, *H*^4,5′^), 7.18–7.12 (m, 1H, *H*^4′^), 7.06 (d, 1H, *J* = 2.1 Hz, *H*^5^). ^13^C{^1^H} NMR ((CD_3_)_2_SO, 101 MHz): δ 151.1 (*C*^1^), 146.1 (*C*^6^), 143.1 (*C*^3^), 138.2 (*C*^1′^), 137.0 (*C*^4a^), 130.3 (*C*^3′^), 128.9 (*C*^8^), 128.1 (*C*^5′^), 126.7 (*C*^2′^), 124.8 (*C*^4′^), 123.7 (*C*^7^), 123.5 (*C*^8a^), 120.2 (*C*^4^), 118.9 (*C*^6′^), 105.5 (*C*^5^).

IR *v̅*_max_ (cm^–1^): 3215 (N–H stretch), 3029 (C–H stretch), 1627 (C=N stretch), 1588 (C=C stretch), 1471 (C=C stretch), 737 (C–Cl stretch).

HRMS (ESI) *m*/*z*: [M + H]^+^ calcd for C_15_H_12_N_2_Cl, 255.0684; found, 255.0688.

### *N*-(2-Chloro-4-(trifluoromethyl)phenyl)isoquinolin-6-amine (**7d**)

The reaction was carried out according to the general procedure using 6-bromoisoquinoline (104 mg, 0.50 mmol, 1.0 equiv) and 2-chloro-4-(trifluoromethyl)aniline (117 mg, 0.60 mmol, 1.2 equiv) as starting materials. The crude residue was purified by silica gel column chromatography, petroleum ether (40–60 °C):EtOAc (6:4), to obtain compound **7d** in 78% yield (116 mg, yellow crystalline solid).

^1^H NMR ((CD_3_)_2_SO, 400 MHz): δ 9.12 (s, 1H, *H*^1^), 8.71 (s, 1H, N*H*), 8.35 (d, 1H, *J* = 5.7 Hz, *H*^3^), 8.03 (d, 1H, *J* = 8.9 Hz, *H*^8^), 7.88 (s, 1H, *H*^5′^), 7.64–7.61 (m, 3H, *H*^4, 3′, 6′^), 7.55 (dd, 1H, *J* = 8.8, 2.2 Hz, *H*^7^), 7.49 (d, 1H, *J* = 2.0 Hz, *H*^5^). ^13^C{^1^H} NMR ((CD_3_)_2_SO, 101 MHz): δ 151.4 (*C*^1^), 143.6 (*C*^6^), 143.3 (*C*^3^), 142.9 (*C*^1′^), 136.6 (*C*^4a^), 129.0 (*C*^8^), 127.2 (q, *J* = 3.7 Hz, *C*^5′^), 125.2 (q, *J* = 3.7 Hz, *C*^3′^), 124.4 (*C*^8a^), 123.7 (q, *J* = 272.9 Hz, −*C*F_3_), 123.6 (*C*^2′^), 122.4 (q, *J* = 33.2 Hz, *C*^4′^), 121.8 (*C*^7^), 119.4 (*C*^4, 6′^), 110.3 (*C*^5^). ^19^F NMR ((CD_3_)_2_SO, 471 MHz): δ −60.20.

IR *v̅*_max_ (cm^–1^): 3220 (N–H stretch), 1612 (C=N stretch), 1487 (C=C stretch), 823 (C–Cl stretch).

HRMS (ESI) *m*/*z*: [M + H]^+^ calcd for C_16_H_11_N_2_ClF_3_, 323.0557; found, 323.0542.

### *N*-(2-Chloro-4-nitrophenyl)isoquinolin-6-amine (**7e**)

The reaction was carried out following the general procedure using 6-bromoisoquinoline (104 mg, 0.50 mmol, 1.0 equiv) and 4-nitro-2-chloroaniline (104 mg, 0.60 mmol, 1.2 equiv) as starting materials. The crude residue was purified by silica gel column chromatography, petroleum ether (40–60 °C):EtOAc (1:1), to obtain compound **7e** in 56% yield (83 mg, yellow solid).

^1^H NMR ((CD_3_)_2_SO, 400 MHz): δ 9.20 (s, 1H, *H*^1^), 9.07 (s, 1H, N*H*), 8.42 (d, 1H, *J* = 5.8 Hz, *H*^3^), 8.34 (d, 1H, *J* = 2.7 Hz, *H*^3′^), 8.12 (d, 1H, *J* = 8.8 Hz, *H*^8^), 8.10 (dd, 1H, *J* = 9.2, 2.7 Hz, *H*^5′^), 7.73 (d, 1H, *J* = 2.0 Hz, *H*^5^), 7.71 (d, 1H, *J* = 5.8 Hz, *H*^4^), 7.64 (dd, 1H, *J* = 8.8, 2.0 Hz, *H*^7^), 7.48 (d, 1H, *J* = 9.2 Hz, *H*^6′^). ^13^C{^1^H} NMR (101 MHz, DMSO-*d*_6_): δ 151.7 (*C*^1^), 146.3 (*C*^4′^), 143.4 (*C*^3^), 142.1 (*C*^6^), 139.5 (*C*^1′^), 136.3 (*C*^8a^), 129.2 (*C*^8^), 125.9 (*C*^3′^), 125.2 (*C*^4a^), 124.3 (*C*^5′^), 123.2 (*C*^7^), 121.0 (*C*^2′^), 119.7 (*C*^4^), 115.7 (*C*^6′^), 114.4 (*C*^5^).

IR *v̅*_max_ (cm^–1^): 3221 (N–H stretch), 1584 (C=N bend), 1508 (N–O stretch), 1474 (C=C stretch), 1333 (N–O stretch), 821 (C–Cl stretch).

HRMS (ESI) *m*/*z*: [M + H]^+^ calcd for C_15_H_11_O_2_N_3_Cl, 300.0534; found, 300.0538.

### *N*-(2-Chloro-5-fluorophenyl)isoquinolin-6-amine (**7f**)

The reaction was carried out according to the general procedure using 6-bromoisoquinoline (104 mg, 0.50 mmol, 1.0 equiv) and 2-chloro-5-fluoroaniline (87 mg, 0.60 mmol, 1.2 equiv) as starting materials. The crude residue was purified by silica gel column chromatography, petroleum ether (40–60 °C):EtOAc (6:4), to obtain compound **7f** in 74% yield (101 mg, yellow solid).

^1^H NMR ((CD_3_)_2_SO, 400 MHz): δ 9.06 (s, 1H, *H*^1^), 8.51 (s, 1H, N*H*), 8.31 (d, 1H, *J* = 5.8 Hz, *H*^3^), 7.98 (d, 1H, *J* = 8.9 Hz, *H*^8^), 7.58 (d, 1H, *J* = 5.8 Hz, *H*^4^), 7.57 (dd, 1H, *J* = 9.0, 6.0 Hz, *H*^3′^), 7.47 (dd, 1H, *J* = 8.9, 2.2 Hz, *H*^7^), 7.33–7.27 (m, 2H, *H*^5,6′^), 6.95 (ddd, 1H, *J* = 8.8, 8.0, 3.0 Hz, *H*^4′^). ^13^C{^1^H} NMR ((CD_3_)_2_SO, 101 MHz): δ 161.2 (d, *J* = 244.7 Hz, *C*^5′^), 151.3 (*C*^1^), 144.7 (*C*^4a^), 143.2 (*C*^3^), 140.2 (d, *J* = 10.8 Hz, *C*^1′^) 136.9 (*C*^8a^), 131.5 (d, *J* = 10.0 Hz, *C*^3′^), 129.0 (*C*^8^), 124.0 (*C*^6^), 120.8 (*C*^7^), 120.5 (d, *J* = 3.3 Hz, *C*^2′^), 119.2 (*C*^4^), 110.5 (d, *J* = 20.9 Hz, *C*^4′^), 108.5 (d, *J* = 26.0 Hz, *C*^6′^), 107.7 (*C*^5^). ^19^F NMR ((CD_3_)_2_SO, 376 MHz): δ −113.1.

IR *v̅*_max_ (cm^–1^): 3221 (N–H stretch), 1592 (C=N stretch), 1474 (C=C stretch), 852 (C–Cl stretch).

HRMS (ESI) *m*/*z*: [M + H]^+^ calcd for C_15_H_11_N_2_ClF, 273.0589; found, 273.0594.

### *N*-(2-Chloro-4-methoxyphenyl)quinolin-6-amine (**7g**)

The reaction was carried out according to the general procedure using 6-bromoquinoline (104 mg, 0.50 mmol, 1.0 equiv) and 4-methoxy-2-chloroaniline (93 mg, 0.60 mmol, 1.2 equiv) as starting materials. The crude residue was purified by silica gel chromatography petroleum ether (40–60 °C):EtOAc (7:3), to obtain compound **7g** in 59% yield (84 mg, brown solid).

^1^H NMR ((CD_3_)_2_SO, 400 MHz): δ 8.56 (dd, *J* = 4.2, 1.6 Hz, 1H, *H*^2^), 8.03–7.97 (m, 2H, N*H*, *H*^4^), 7.82 (d, *J* = 9.1 Hz, 1H, *H*^8^), 7.40 (dd, *J* = 9.0, 2.6 Hz, 1H, *H*^7^), 7.38 (d, *J* = 8.8 Hz, 1H, *H*^6′^), 7.31 (dd, *J* = 8.3, 4.2 Hz, 1H, *H*^3^), 7.16 (d, *J* = 2.8 Hz, 1H, *H*^3′^), 6.97 (dd, *J* = 8.8, 2.8 Hz, 1H, *H*^5′^), 6.85 (d, *J* = 2.5 Hz, 1H, *H*^5^), 3.80 (s, 3H, −OC*H_3_*). ^13^C{^1^H} NMR ((CD_3_)_2_SO, 101 MHz): δ 156.4 (*C*^4′^), 146.3 (*C*^2^), 144.1 (*C*^6^), 143.0 (*C*^8a^), 133.7 (*C*^4^), 131.6 (*C*^1^), 129.8 (*C*^4a^), 129.3 (*C*^8^), 129.2 (*C*^2′^), 126.5 (*C*^6′^), 121.8 (*C*^7^), 121.5 (*C*^3^), 115.2 (*C*^3′^), 114.2 (*C*^5′^), 105.6 (*C*^5^), 55.7 (−O*C*H_3_).

IR *v̅*_max_ (cm^–1^): 3215 (C–N stretch), 3016 (C–H stretch), 2846 (C–H stretch), 1631 (C=N stretch), 1498 (C=C stretch), 1214 (C–O stretch), 1050 (C–O stretch), 839 (C–Cl stretch).

HRMS (ESI) *m*/*z*: [M + H]^+^ calcd for C_16_H_14_O_2_N_2_Cl, 285.0789; found, 285.0793.

### *N*-(2-Chloro-4-methoxyphenyl)quinoxalin-6-amine (**7h**)

The reaction was carried out according to the general procedure using 6-bromoquinoxaline (105 mg, 0.50 mmol, 1.0 equiv) and 4-methoxy-2-chloroaniline (95 mg, 0.60 mmol, 1.2 equiv) as starting materials. The crude residue was purified by silica gel column chromatography, petroleum ether (40–60 °C):EtOAc (7:3) to obtain compound **7h** in 87% yield (124 mg, yellow solid).

^1^H NMR ((CD_3_)_2_SO, 400 MHz): δ 8.66 (d, 1H, *J* = 2.0 Hz, *H*^3^), 8.55 (d, 1H, *J* = 2.0 Hz, *H*^2^), 8.42 (s, 1H, N*H*), 7.87 (d, 1H, *J* = 9.1 Hz, *H*^8^), 7.46 (dd, 1H, *J* = 9.1, 2.6 Hz, *H*^7^), 7.42 (d, 1H, *J* = 8.8 Hz, *H*^6′^), 7.20 (d, 1H, *J* = 2.9 Hz, *H*^3′^), 7.01 (dd, 1H, *J* = 8.8, 2.9 Hz, *H*^5′^), 6.83 (d, 1H, *J* = 2.6 Hz, *H*^5^), 3.82 (s, 3H, −OC*H_3_*). ^13^C{^1^H} NMR ((CD_3_)_2_SO, 101 MHz): δ 157.2 (*C*^4′^), 147.7 (*C*^6^), 145.3 (*C*^3^), 144.4 (*C*^4a^), 141.0 (*C*^2^), 137.4 (*C*^8a^), 130.5 (*C*^1′^), 130.2 (*C*^8^), 129.8 (*C*^2′^), 127.8 (*C*^6′^), 122.1 (*C*^7^), 115.3 (*C*^3′^), 114.3 (*C*^5′^), 105.3 (*C*^5^), 55.7 (−O*C*H_3_).

IR *v̅*_max_ (cm^–1^): 3301 (N–H stretch), 3068 (C–H stretch), 2837 (C–H stretch), 1618 (C=N stretch), 1504 (C=C stretch), 1221 (C–O stretch), 1037 (C–O stretch), 852 (C–Cl stretch).

HRMS (ESI) *m*/*z*: [M + H]^+^ calcd for C_15_H_13_ON_3_Cl, 286.0742; found, 286.0740.

### *N*-(2-Chloro-4-methoxyphenyl)quinoxalin-2-amine (**7i**)

The reaction was carried out according to the general procedure using 2-bromoquinoxaline (105 mg, 0.50 mmol, 1.0 equiv) and 4-methoxy-2-chloroaniline (95 mg, 0.60, 1.2 equiv) as starting materials. The crude residue was purified by silica gel column chromatography, petroleum ether (40–60 °C):EtOAc (85:15), to obtain compound **7i** in 27% yield (38 mg, yellow solid).

^1^H NMR ((CD_3_)_2_SO, 400 MHz): δ 9.20 (s, 1H, N*H*), 8.64 (s, 1H, *H*^3^), 7.92 (d, 1H, *J* = 8.9 Hz, *H*^6′^), 7.84 (dd, 1H, *J* = 8.2, 1.2 Hz, *H*^8^), 7.62–7.53 (m, 2H, *H*^5,6^), 7.46–7.41 (m, 1H, *H*^7^), 7.15 (d, 1H, *J* = 2.9 Hz, *H*^3′^), 7.00 (dd, 1H, *J* = 8.9, 2.9 Hz, *H*^5′^), 3.81 (s, 3H, −OC*H_3_*). ^13^C{^1^H} NMR ((CD_3_)_2_SO, 101 MHz): δ 156.6 (*C*^4′^), 150.5 (*C*^2^), 140.8 (*C*^3^), 139.8 (*C*^8a^), 137.1 (*C*^4a^), 129.9 (*C*^5^), 128.9 (*C*^1′^), 128.4 (*C*^8^), 128.0 (*C*^2′^), 126.9 (*C*^6′^), 126.2 (*C*^6^), 124.7 (*C*^7^), 114.6 (*C*^3′^), 113.6 (*C*^5′^), 55.7 (−O*C*H_3_).

IR *v̅*_max_ (cm^–1^): 3340 (N–H stretch), 2934 (C–H stretch), 1590 (C=N stretch), 1536 (C=N stretch), 1502 (C=C stretch), 1214 (C–O stretch), 1040 (C–O stretch), 841 (C–Cl stretch).

HRMS (ESI) *m*/*z*: [M + H]^+^ calcd for C_15_H_13_ON_3_Cl, 286.0742; found, 286.0745.

### *N*-(2-Chloro-4-methoxyphenyl)-*N*-(quinoxalin-2-yl)quinoxalin-2-amine (**8**)

The reaction was carried out according to the general procedure using 2-bromoquinoxaline (105 mg, 0.50 mmol, 1.0 equiv) and 4-methoxy-2-chloroaniline (95 mg, 0.60 mmol, 1.2 equiv) as starting materials. The crude residue was purified by silica gel column chromatography, petroleum ether (40–60 °C):EtOAc (85:15) to obtained compound **8** in 25% yield (26 mg, yellow solid).

^1^H NMR ((CD_3_)_2_SO, 400 MHz): δ 8.83 (s, 2H, 2 × *H*^3^), 8.06–8.00 (m, 2H, 2 × *H*^5^), 7.76–7.68 (m, 6H, 2 × *H*^6/7/8^), 7.58 (d, 1H, *J* = 8.8 Hz, *H*^6′^), 7.32 (d, 1H, *J* = 2.9 Hz, *H*^3′^), 7.12 (dd, 1H, *J* = 8.8, 2.9 Hz, *H*^5′^), 3.88 (s, 3H, −OC*H_3_*). ^13^C{^1^H} NMR ((CD_3_)_2_SO, 101 MHz): δ 159.6 (*C*^4′^), 150.4 (2 × *C*^2^), 141.5 (2 × *C*^3^), 140.0 (2 × *C*^8a^), 138.8 (2 × *C*^4a^), 133.1 (*C*^2′^), 132.3 (*C*^6′^), 130.9 (*C*^1′^), 130.6 (2 × *C*^6^ or C^7^ or C^8^), 128.5 (2 × *C*^5^), 128.0 (2 × *C*^6^ or C^7^ or C^8^), 127.4 (2 × *C*^6^ or C^7^ or C^8^), 115.9 (*C*^3′^), 115.0 (*C*^5′^), 55.9 (−O*C*H_3_).

IR *v̅*_max_ (cm^–1^): 1606 (C=N stretch), 1558 (C=N stretch), 1498 (C=C stretch), 1420 (C=C stretch), 1229 (C–O stretch), 1022 (C–O stretch), 761 (C–Cl stretch).

HRMS (ESI) *m*/*z*: [M + H]^+^ calcd for C_23_H_17_ON_5_Cl, 414.1116; found, 414.1115.

### *N*-(2-Chloro-4-methoxyphenyl)-1,3-dihydroisobenzofuran-5-amine (**7j**)

The reaction was carried out according to the general procedure using 5-bromo-1,3-dihydroisobenzofuran (100 mg, 0.50 mmol, 1.0 equiv) and 4-methoxy-2-chloroaniline (95 mg, 0.60, 1.2 equiv) as starting materials. The crude residue was purified by reversed-phase semi-preparative HPLC over a 5–95% gradient B, (solvent A: 0.1% TFA in water, solvent B: 0.1% TFA in acetonitrile) to obtain compound **7j** in 18% yield (35 mg, white solid) as the TFA salt.

^1^H NMR ((CD_3_)_2_SO, 400 MHz): δ 7.49 (s, 1H, N*H*), 7.22 (d, 1H, *J* = 8.9 Hz, *H*^6′^), 7.08 (d, 1H, *J* = 2.8 Hz, *H*^3′^), 7.07 (d, 1H, *J* = 8.4 Hz, *H*^7^), 6.89 (dd, 1H, *J* = 8.9, 2.8 Hz, *H*^5′^), 6.71 (dd, 1H, *J* = 8.4, 2.0 Hz, *H*^6^), 6.66 (d, 1H, *J* = 2.0 Hz, *H*^4^), 4.88 (s, 4H, 2 × −C*H_2_*), 3.75 (s, 3H, −OC*H_3_*).

^13^C{^1^H} NMR ((CD_3_)_2_SO, 101 MHz): δ 155.4 (*C*^4′^), 144.9 (*C*^4a^), 140.0 (*C*^3a^), 132.8 (*C*^1′^), 128.9 (*C*^7a^), 127.7 (*C*^2′^), 124.7 (*C*^6′^), 121.4 (*C*^7^), 115.0 (*C*^3′^), 114.8 (*C*^6^), 114.0 (*C*^5′^), 107.3 (*C*^4^), 72.5 (*C*^1^ or C^3^), 72.3 (*C*^1^ or C^3^), 55.6 (−O*C*H_3_).

IR *v̅*_max_ (cm^–1^): 3319 (N–H stretch), 2854 (C–H stretch), 1621 (C=C stretch), 1497 (C=C stretch), 1273 (C–O stretch), 1217 (C–O stretch), 1031 (C–O stretch), 821 (C–Cl stretch).

HRMS (ESI) *m*/*z*: [M + H]^+^ calcd for C_15_H_15_O_2_NCl, 276.0786; found, 276.0792.

### 2-((2-Chloro-4-methoxyphenyl)amino)naphthalene-1,4-dione (**7k**)

The reaction was carried out according to the general procedure using 2-bromo-1,4-napthoquinone (119 mg, 0.50 mmol, 1.0 equiv) and 4-methoxy-2-chloroaniline (95 mg, 0.60 mmol, 1.2 equiv) as starting materials. The crude residue was purified by silica gel column chromatography, petroleum ether (40–60 °C):EtOAc:NEt_3_ (100:8:1), to obtain compound **7k** in 82% yield (129 mg, dark red solid). mp 155–158 °C.

^1^H NMR ((CD_3_)_2_SO, 400 MHz): δ 9.02 (s, 1H, N*H*), 8.06 (dd, 1H, *J* = 7.5, 1.0 Hz, *H*^5^), 7.94 (dd, 1H, *J* = 8.8, 1.2 Hz, *H*^8^), 7.86 (ddd, 1H, *J* = 7.5, 7.5, 1.4 Hz, *H*^6^), 7.79 (ddd, 1H, *J* = 7.5, 7.5, 1.4 Hz, *H*^7^), 7.36 (d, 1H, *J* = 8.8 Hz, *H*^6′^), 7.22 (d, 1H, *J* = 2.8 Hz, *H*^3′^), 7.04 (dd, 1H, *J* = 8.8, 2.8 Hz, *H*^5′^), 5.31 (s, 1H, *H*^3^), 3.82 (s, 3H, −OC*H_3_*). ^13^C{^1^H} NMR ((CD_3_)_2_SO, 101 MHz): δ 182.1 (*C*^1^), 181.3 (*C*^4^), 158.7 (*C*^4′^), 147.7 (*C*^2^), 135.0 (*C*^6^), 132.6 (*C*^7^), 131.2 (*C*^1′^), 130.7 (*C*^8a^), 130.3 (*C*^4a^), 129.5 (*C*^3′^), 127.4 (*C*^2′^), 126.0 (*C*^5^), 125.4 (*C*^8^), 115.1 (*C*^6′^), 114.4 (*C*^5′^), 102.3 (*C*^3^), 55.8 (−O*C*H_3_).

IR *v̅*_max_ (cm^–1^): 3331 (N–H stretch), 3021 (C–H stretch), 1677 (C=O stretch), 1577 (C=C stretch), 1489 (C=C stretch), 1216 (C–O stretch), 1031 (C–O stretch), 778 (C–Cl stretch).

HRMS (ESI) *m*/*z*: [M + H]^+^ calcd for C_17_H_13_O_3_NCl, 314.0578; found, 314.0577.

### 2-((2-Chloro-4-methylphenyl)amino)naphthalene-1,4-dione (**7l**)

The reaction was carried out according to the general procedure using 2-bromo-1,4-napthoquinone (119 mg, 0.50 mmol, 1.0 equiv) and 4-methyl-2-chloroaniline (85 mg, 0.60 mmol, 1.2 equiv) as starting materials. The crude residue was purified by silica gel column chromatography, petroleum ether (40–60 °C):EtOAc:NEt_3_ (90:10:1), to obtain compound **7l** in 72% yield (107 mg, red solid). mp 150–154 °C.

^1^H NMR ((CD_3_)_2_SO, 400 MHz): δ 9.01 (s, 1H, N*H*), 8.09–8.05 (m, 1H, *H*^5^), 7.96–7.92 (m, 1H, *H*^8^), 7.89–7.84 (m, 1H, *H*^6^), 7.82–7.77 (m, 1H, *H*^7^), 7.47 (d, 1H, *J* = 1.1 Hz, *H*^3′^), 7.35 (d, 1H, *J* = 8.1 Hz, *H*^6′^), 7.27 (dd, 1H, *J* = 8.1, 1.3 Hz, *H*^5′^), 5.41 (s, 1H, *H*^3^), 2.35 (s, 3H, −*CH_3_*). ^13^C{^1^H} NMR ((CD_3_)_2_SO, 101 MHz): δ 182.3 (*C*^4^), 181.2 (*C*^1^), 147.0 (*C*^2^), 138.5 (*C*^1′^), 135.0 (*C*^6^), 132.7 (*C*^4a^), 132.6 (*C*^7^), 132.1 (*C*^2′^), 130.5 (*C*^8a^), 130.3 (*C*^3′^), 129.6 (*C*^4′^), 129.0 (*C*^5′^), 127.9 (*C*^6′^), 126.1 (*C*^5^), 125.4 (*C*^8^), 102.7 (*C*^3^), 20.3 (−*C*H_3_).

IR *v̅*_max_ (cm^–1^): 3342 (N–H stretch), 2927 (C–H stretch), 1675 (C=O stretch), 1599 (C=O stretch), 1526 (C=C stretch), 774 (C–Cl).

HRMS (ESI) *m*/*z*: [M + H]^+^ calcd for C_17_H_13_O_2_NCl, 298.0629; found, 298.0633.

### 2-((2-Chlorophenyl)amino)naphthalene-1,4-dione (**7m**)

The reaction was carried out according to the general procedure using 2-bromo-1,4-napthoquinone (119 mg, 0.50 mmol, 1.0 equiv) and 2-chloroaniline (77 mg, 0.60 mmol, 1.2 equiv) as starting materials. The crude residue was purified by silica gel column chromatography, petroleum ether (40–60 °C):EtOAc (9:1) to obtain compound **7m** in 64% yield (90 mg, brown solid).

^1^H NMR ((CD_3_)_2_SO, 400 MHz): δ 9.08 (s, 1H, N*H*), 8.08 (dd, 1H, *J* = 7.7, 0.9 Hz, *H*^5^), 7.95 (dd, 1H, *J* = 7.5, 1.1 Hz, *H*^8^), 7.87 (ddd, 1H, *J* = 7.4, 7.4, 1.4 Hz, *H*^6^), 7.80 (ddd, 1H, *J* = 8.0, 6.5, 2.7 Hz, *H*^7^), 7.64 (dd, 1H, *J* = 7.5, 1.1 Hz, *H*^3′^), 7.51–7.44 (m, 2H, *H*^4′,6′^), 7.38 (ddd, 1H, *J* = 8.0, 6.5, 2.7 Hz, *H*^5′^), 5.47 (s, 1H, *H*^3^). ^13^C{^1^H} NMR ((CD_3_)_2_SO, 101 MHz): δ 182.3 (*C*^4^), 181.2 (*C*^1^), 146.7 (*C*^2^), 135.0 (*C*^6^), 134.2 (*C*^1′^), 132.8 (*C*^7^), 132.5 (*C*^4a^), 130.3 (*C*^3′^), 129.7 (*C*^8a^), 128.4 (*C*^2′^), 128.3 (*C*^5′^), 128.0 (*C*^4′, 6′^), 126.1 (*C*^5^), 125.4 (*C*^8^), 102.9 (*C*^3^).

IR *v̅*_max_ (cm^–1^): 3310 (N–H stretch), 3068 (C–H stretch), 2854 (C–H stretch), 1675 (C=O stretch), 1618 (C=O stretch), 1592 (C=C stretch), 1446 (C=C stretch), 741 (C–Cl stretch).

HRMS (ESI) *m*/*z*: [M + H]^+^ calcd for C_16_H_11_O_2_NCl, 284.0473; found, 284.0480.

### 2-((2-Chloro-4-(trifluoromethyl)phenyl)amino)naphthalene-1,4-dione (**7n**)

The reaction was carried out according to the general procedure using 2-bromo-1,4-napthoquinone (119 mg, 0.50 mmol, 1.0 equiv) and 2-chloro-4-(trifluoromethyl)aniline (117 mg, 0.60 mmol, 1.2 equiv) as starting materials. The crude residue was purified by silica gel column chromatography, petroleum ether (40–60 °C):EtOAc (9:1) to obtain compound **7n** in 69% yield (122 mg, orange solid). mp 180–183 °C.

^1^H NMR ((CD_3_)_2_SO, 400 MHz): δ 9.10 (s, 1H, N*H*), 8.08 (dd, 1H, *J* = 7.5, 1.1 Hz, *H*^8^), 8.06 (d, 1H, *J* = 1.6 Hz, *H*^3′^), 7.96 (dd, 1H, *J* = 7.6, 1.2 Hz, *H*^5^), 7.88 (ddd, 1H, *J* = 7.5, 7.5, 1.5 Hz, *H*^6^), 7.85–7.78 (m, 2H, *H*^5′, 7^), 7.75 (d, 1H, *J* = 8.5 Hz, *H*^6′^), 5.77 (s, 1H, *H*^3^).

^13^C{^1^H} NMR ((CD_3_)_2_SO, 101 MHz): δ 182.7 (*C*^4^), 181.0 (*C*^1^), 146.0 (*C*^2^), 138.9 (*C*^1′^), 135.1 (*C*^6^), 133.0 (*C*^7^), 132.3 (*C*^4a^), 130.2 (*C*^8a^), 129.5 (*C*^2′^), 127.5 (q, *J* = 32.7 Hz, *C*^4′^), 127. 4 (q, *J* = 3.8 Hz, *C*^3′^), 127.2 (*C*^6′^), 126.2 (*C*^8^), 125.5 (*C*^5^), 125.4 (q, *J* = 3.8 Hz, *C*^5′^), 123.3 (q, *J* = 272.9 Hz, −*C*F_3_), 104.6 (*C*^3^). ^19^F NMR ((CD_3_)_2_SO, 376 MHz): δ −60.85.

IR *v̅*_max_ (cm^–1^): 3338 (N–H stretch), 3055 (C–H stretch), 1677 (C=O stretch), 1642 (C=O stretch), 1580 (C=C stretch), 782 (C–Cl stretch).

HRMS (ESI) *m*/*z*: [M + H]^+^ calcd for C_17_H_10_O_2_NClF_3_, 352.0347; found, 352.0347.

### 2-((2-Chloro-5-methoxyphenyl)amino)naphthalene-1,4-dione (**7o**)

The reaction was carried out according to the general procedure using 2-bromo-1,4-napthoquinone (119 mg, 0.50 mmol, 1.0 equiv) and 5-methoxy-2-chloroaniline (95 mg, 0.60 mmol, 1.2 equiv) as starting materials. The crude residue was purified by silica gel column chromatography, petroleum ether (40–60 °C):EtOAc (85:15), to obtain compound **7o** in 59% yield (92 mg, red solid). mp 152–156 °C.

^1^H NMR ((CD_3_)_2_SO, 400 MHz): δ 9.05 (s, 1H, N*H*), 8.07 (dd, 1H, *J* = 7.6, 1.2 Hz, *H*^8^), 7.95 (dd, 1H, *J* = 7.6, 1.2 Hz, *H*^5^), 7.90–7.84 (m, 1H, *H*^6^), 7.83–7.77 (m, 1H, *H*^7^), 7.52 (d, 1H, *J* = 8.9 Hz, *H*^3′^), 7.03 (d, 1H, *J* = 3.0 Hz, *H*^6′^), 6.97 (dd, 1H, *J* = 8.9, 3.0 Hz, *H*^4′^), 5.48 (s, 1H, *H*^3^), 3.79 (s, 3H, −OC*H_3_*). ^13^C{^1^H} NMR ((CD_3_)_2_SO, 101 MHz): δ 182.3 (*C*^4^=O), 181.2 (*C*^1^=O), 158.8 (*C*^5′^), 146.6 (*C*^2^), 135.8 (*C*^1′^), 135.0 (*C*^6^), 132.8 (*C*^7^), 132.6 (*C*^4a^), 130.7 (*C*^3′^), 130.3 (*C*^8a^), 126.1 (*C*^8^), 125.4 (*C*^5^), 120.9 (*C*^2′^), 114.0 (*C*^4′^), 113.3 (*C*^6′^), 103.3 (*C*^3^), 55.7 (−O*C*H_3_).

IR *v̅*_max_ (cm^–1^): 3299 (N–H stretch), 2843 (C–H stretch), 1675 (C=O stretch), 1647 (C=O stretch), 1579 (C=C stretch), 1541 (C=C stretch), 1219 (C–O stretch), 1067 (C–O stretch), 776 (C–Cl stretch).

HRMS (ESI) *m*/*z*: [M + H]^+^ cald for C_17_H_13_O_3_NCl, 314.0578; found, 314.0586.

### 2-((2-Chloro-5-fluorophenyl)amino)naphthalene-1,4-dione (**7p**)

The reaction was carried out according to the general procedure using 2-bromo-1,4-napthoquinone (119 mg, 0.50 mmol, 1.0 equiv) and 2-chloro-5-fluoroaniline (87 mg, 0.60 mmol, 1.2 equiv) as starting materials. The crude residue was purified by silica gel column chromatography, petroleum ether (40–60 °C):EtOAc (9:1) to obtain compound **7p** in 80% yield (120 mg, orange solid).

^1^H NMR ((CD_3_)_2_SO, 400 MHz): δ 9.08 (s, 1H, N*H*), 8.10–8.06 (m, 1H, *H*^8^), 7.98–7.94 (m, 1H, *H*^5^), 7.91–7.85 (m, 1H, *H*^6^), 7.85–7.79 (m, 1H, *H*^7^), 7.68 (dd, 1H, *J* = 9.0, 5.7 Hz, *H*^3′^), 7.43 (dd, 1H, *J* = 9.5, 3.0 Hz, *H*^6′^), 7.26 (ddd, 1H, *J* = 8.8, 8.1, 3.0 Hz, *H*^4′^), 5.60 (s, 1H, *H*^3^).

^13^C{^1^H} NMR ((CD_3_)_2_SO, 101 MHz): δ 182.6 (*C*^4^), 181.0 (*C*^1^), 160.9 (d, *J* = 246.8 Hz, *C*^5′^), 146.0 (*C*^2^), 136.4 (d, *J* = 10.8 Hz, *C*^1′^), 135.1 (*C*^6^), 132.9 (*C*^7^), 132.4 (*C*^4a^), 131.6 (d, *J* = 9.6 Hz, *C*^3′^), 130.2 (*C*^8a^), 126.2 (*C*^8^), 125.4 (*C*^5^), 125.1 (*C*^2′^), 115.2 (d, *J* = 22.8 Hz, *C*^4′^), 114.8 (d, *J* = 24.8 Hz, *C*^6′^), 103.9 (*C*^3^). ^19^F NMR ((CD_3_)_2_SO, 376 MHz): δ −112.74.

IR *v̅*_max_ (cm^–1^): 3310 (N–H stretch), 3074 (C–H stretch), 2858 (C–H stretch), 1675 (C=O stretch), 1647 (C=O stretch), 1599 (C=C stretch), 1541 (C=C stretch), 722 (C–Cl stretch).

HRMS (ESI) *m*/*z*: [M + H]^+^ calcd for C_16_H_10_O_2_NClF, 302.0379; found, 302.0380.

### General Experimental Procedure A for Microwave-Assisted One-Pot Buchwald–Hartwig Amination/Direct Arylation

Aryl bromide (0.50 mmol, 1 equiv), chloroaniline (0.60 mmol, 1.2 equiv), Pd(OAc)_2_ (5 mol %), DavePhos (10 mol %), and K_3_PO_4_ (1.50 mmol, 3 equiv) were added to a microwave vial (2–5 mL). 1,4-Dioxane (5.0 mL, 0.1 M) was added and the vial was capped, evacuated and purged with argon three times, and heated at 120 °C for 30 min followed by 160 °C for 8 h under microwave irradiation in a Biotage microwave. The reaction was allowed to cool to rt, diluted with DCM (50 mL), and the solid was filtered under vacuum. The solvent was removed *in vacuo*, and the crude sample was dry-loaded onto silica gel without work up. The crude compound was purified by silica column chromatography.

### General Experimental Procedure B for Microwave-Assisted One-Pot Buchwald–Hartwig Amination/Direct Arylation

Aryl bromide (0.50 mmol, 1 equiv), chloroaniline (0.60 mmol, 1.2 equiv), Pd(OAc)_2_ (5 mol %), P(Cy_3_), HBF_4_ (10 mol %), and K_3_PO_4_ (1.50 mmol, 3 equiv) were added to a microwave vial (2–5 mL). 1,4-Dioxane (5.0 mL, 0.1 M) was added, and the vial was capped, evacuated and purged with argon three times, and heated at 120 °C for 30 min followed by 160 °C for 8 h under microwave irradiation in a Biotage microwave. The reaction was allowed to cool to rt and diluted with DCM (50 mL). The solvent was removed *in vacuo*, and the crude sample dry-loaded onto silica gel without work up. The crude compound was purified by silica column chromatography.

### 10-Methoxy-7*H*-pyrido[3,4-*c*]carbazole (**9a**)

The reaction was carried out according to the general procedure B using 6-bromoisoquinoline (104 mg, 0.50 mmol, 1.0 equiv) and 2-chloro-4-methoxyaniline (95 mg, 0.60 mmol, 1.2 equiv) as starting materials. The crude residue was purified by silica gel column chromatography, petroleum ether (40–60 °C):EtOAc (1:1), to obtain compound **9a** in 82% yield (102 mg, brown solid).

^1^H NMR ((CD_3_)_2_SO, 400 MHz): δ 11.94 (s, 1H, −N*H*), 9.30 (s, 1H, *H*^4^), 8.63 (d, 1H, *J* = 5.8 Hz, *H*^2^), 8.58 (d, *J* = 5.8 Hz, 1H, *H*^1^), 8.05–7.99 (m, 2H, *H*^5,11^), 7.85 (d, 1H, *J* = 8.8 Hz, *H*^6^), 7.60 (d, 1H, *J* = 8.8 Hz, *H*^8^), 7.13 (dd, 1H, *J* = 8.8, 2.4 Hz, *H*^9^), 3.96 (s, 3H, −OC*H_3_*). ^13^C{^1^H} NMR ((CD_3_)_2_SO, 101 MHz): δ 154.1 (*C*^10^), 152.0 (*C*^4^), 144.1 (*C*^2^), 140.0 (*C*^6a^), 133.6 (*C*^7a^), 132.1 (*C*^11c^), 125.9 (*C*^5^), 123.6 (*C*^11b^), 123.0 (*C*^11a^), 116.4 (*C*^1^), 115.0 (*C*^6^), 114.3 (*C*^9^), 112.6 (*C*^8^), 112.3 (*C*^4a^), 103.9 (*C*^11^), 55.8 (−O*C*H_3_).

IR *v̅*_max_ (cm^–1^): 2830 (C–H stretch), 1616 (C=N stretch), 1458 (C=C stretch), 1229 (C–O stretch), 1031 (C–O stretch).

HRMS (ESI) *m*/*z*: [M + H]^+^ calcd for C_16_H_13_ON_2_, 249.1022; found, 249.1021.

### 10-Methyl-7*H*-pyrido[3,4-*c*]carbazole (**9b**)

The reaction was carried out according to the general procedure B using 6-bromoisoquinoline (104 mg, 0.50 mmol, 1.0 equiv) and 2-chloro-4-methylaniline (85 mg, 0.60 mmol, 1.2 equiv) as starting materials. The crude residue was purified by silica gel column chromatography, petroleum ether (40–60 °C):EtOAc (1:1) to obtain compound **9b** in 54% yield (63 mg, brown solid).

^1^H NMR ((CD_3_)_2_SO, 400 MHz): δ 11.97 (s, 1H, −N*H*), 9.31 (s, 1H, *H*^4^), 8.63 (d, 1H, *J* = 5.7 Hz, *H*^2^), 8.58 (d, 1H, *J* = 5.7 Hz, *H*^1^), 8.41 (s, 1H, *H*^11^), 8.04 (d, 1H, *J* = 8.8 Hz, *H*^5^), 7.86 (d, 1H, *J* = 8.8 Hz, *H*^6^), 7.58 (d, 1H, *J* = 8.3 Hz, *H*^8^), 7.30 (dd, 1H, *J* = 8.3, 1.2 Hz, *H*^9^), 2.58 (s, 3H, −C*H*_3_). ^13^C{^1^H} NMR ((CD_3_)_2_SO, 101 MHz): δ 152.0 (*C*^4^), 143.9 (*C*^2^), 139.7 (*C*^6a^), 137.0 (*C*^7a^), 132.0 (*C*^11c^), 129.0 (*C*^10^), 126.1 (*C*^9^), 125.8 (*C*^5^), 123.7 (*C*^4a^), 122.9 (*C*^11a^), 121.3 (*C*^11^), 116.4 (*C*^1^), 114.9 (*C*^6^), 112.2 (*C*^11b^), 111.6 (*C*^8^), 21.3 (−*C*H_3_).

IR *v̅*_max_ (cm^–1^): 2917 (C–H stretch), 1616 (C=N stretch), 1474 (C=C stretch).

HRMS (ESI) *m*/*z*: [M + H]^+^ calcd for C_16_H_13_N_2_, 233.1073; found, 233.1068.

### 10-(Trifluoromethyl)-7*H*-pyrido[3,4-*c*]carbazole (**9c**)

The reaction was carried out according to the general procedure B using 6-bromoisoquinoline (104 mg, 0.50 mmol, 1.0 equiv) and 2-chloro-4-(trifluoromethyl)aniline (117 mg, 0.60 mmol, 1.2 equiv) as starting materials. The crude residue was purified by silica gel column chromatography, petroleum ether (40–60 °C):EtOAc (7:2), to obtain compound **9c** in 25% yield (35 mg, yellow solid).

^1^H NMR ((CD_3_)_2_SO, 400 MHz): δ 12.53 (s, 1H, −N*H*), 9.38 (s, 1H, *H*^4^), 8.90 (s, 1H, *H*^11^), 8.74–8.63 (m, 2H, *H*^1, 2^), 8.17 (d, 1H, *J* = 8.8 Hz, *H*^5^), 7.95 (d, 1H, *J* = 8.8 Hz, *H*^6^), 7.88 (d, 1H, *J* = 8.6 Hz, *H*^8^), 7.78 (d, 1H, *J* = 8.6 Hz, *H*^9^). ^13^C{^1^H} NMR ((CD_3_)_2_SO, 101 MHz): δ 152.3 (*C*^4^), 144.6 (*C*^2^), 140.8 (*C*^7a^), 140.5 (*C*^6a^), 131.8 (*C*^11c^), 127.5 (*C*^5^), 125.4 (q, *J* = 272.6 Hz, −*C*F_3_), 124.0 (*C*^4a^), 122.2 (*C*^11a^), 121.2 (q, *J* = 4.0 Hz, *C*^9^), 120.8 (q, *J* = 30.8 Hz, *C*^10^), 118.8 (q, *J* = 4.0 Hz, *C*^11^), 116.6 (*C*^1^), 115.0 (*C*^6^), 112.6 (*C*^8^), 112.4 (*C*^11b^). ^19^F NMR (376 MHz, DMSO-*d*_6_): δ −58.1.

IR *v̅*_max_ (cm^–1^): 1638 (C=N stretch), 1618 (C=N stretch), 1463 (C=C stretch), 1420 (C=C stretch).

HRMS (ESI) *m*/*z*: [M + H]^+^ calcd for C_16_H_10_N_2_F_3_, 287.0791; found, 287.0791.

### 10-Methoxy-7*H*-pyrido[4,3-*c*]carbazole (**9e**)

The reaction was carried out according to the general procedure B using 7-bromoisoquinoline (104 mg, 0.50 mmol, 1.0 equiv) and 2-chloro-4-methoxyaniline (95 mg, 0.60 mmol, 1.2 equiv) as starting materials. The crude residue was purified by silica gel column chromatography, petroleum ether (40–60 °C):EtOAc (1:1), to obtain compound **9e** in 83% yield (103 mg, brown solid).

^1^H NMR ((CD_3_)_2_SO, 400 MHz): δ 11.89 (s, 1H, −N*H*), 10.14 (s, 1H, *H*^1^), 8.53 (d, 1H, *J* = 5.5 Hz, *H*^3^), 8.07 (d, 1H, *J* = 2.4 Hz, *H*^11^), 7.99 (d, 1H, *J* = 8.8 Hz, *H*^6^), 7.96 (d, 1H, *J* = 5.5 Hz, *H*^4^), 7.91 (d, *J* = 8.8 Hz, 1H, *H*^5^), 7.61 (d, *J* = 8.8 Hz, 1H, *H*^8^), 7.14 (dd, *J* = 8.8, 2.4 Hz, 1H, *H*^9^), 3.98 (s, 3H, −OC*H_3_*). ^13^C{^1^H} NMR ((CD_3_)_2_SO, 101 MHz): δ 154.0 (*C*^10^), 146.7 (*C*^1^), 140.5 (*C*^3^), 138.3 (*C*^6a^), 133.8 (*C*^7a^), 131.0 (*C*^11b^), 124.7 (*C*^5, 11c^), 122.1 (*C*^11a^), 121.6 (*C*^4^), 118.3 (*C*^6^), 115.0 (*C*^9^), 113.0 (*C*^4a^), 112.7 (*C*^8^), 104.2 (*C*^11^), 55.8 (−O*C*H_3_).

IR *v̅*_max_ (cm^–1^): 2932 (C–H stretch), 1588 (C=N stretch), 1210 (C–O stretch), 1063 (C–O stretch).

HRMS (ESI) *m*/*z*: [M + H]^+^ calcd for C_16_H_13_ON_2_, 249.1022; found, 249.1028.

### 10-Methoxy-7*H*-pyrazino[2,3-*c*]carbazole (**9f**)

The reaction was carried out according to the general procedure B using 6-bromoquinoxaline (105 mg, 0.50 mmol, 1.0 equiv) and 2-chloro-4-methoxyaniline (95 mg, 0.60 mmol, 1.2 equiv) as starting materials. The crude residue was purified by silica gel column chromatography, petroleum ether (40–60 °C):EtOAc (7:2), to obtain compound **9f** in 75% yield (93 mg, yellow solid).

^1^H NMR ((CD_3_)_2_SO, 400 MHz): δ 11.96 (s, 1H, −N*H*), 9.04 (d, 1H, *J* = 2.0 Hz, *H*^2^), 8.86 (d, 1H, *J* = 2.0 Hz, *H*^3^), 8.29 (d, 1H, *J* = 2.6 Hz, *H*^11^), 8.05 (d, 1H, *J* = 9.1 Hz, *H*^6^), 7.98 (d, 1H, *J* = 9.1 Hz, *H*^5^), 7.61 (d, 1H, *J =* 8.8 Hz, *H*^8^), 7.13 (dd, 1H, *J* = 8.8, 2.6 Hz, *H*^9^), 3.91 (s, 3H, −OC*H_3_*).

^13^C{^1^H} NMR ((CD_3_)_2_SO, 101 MHz): δ 154.1 (*C*^10^), 144.2 (*C*^2^), 141.2 (*C*^3^), 140.3 (*C*^11c^), 139.5 (*C*^6a^), 138.7 (*C*^4a^), 133.8 (*C*^7a^), 126.5 (*C*^5^), 123.3 (*C*^11a^), 118.0 (*C*^6^), 115.0 (*C*^9^), 114.5 (*C*^11b^), 112.7 (*C*^8^), 104.4 (*C*^11^), 55.5 (−O*C*H_3_).

IR *v̅*_max_ (cm^–1^): 3338 (N–H stretch), 2837 (C–H stretch), 1601 (C=N stretch), 1523 (C=C stretch), 1495 (C=C stretch), 1214 (C–O stretch), 1029 (C–O stretch).

HRMS (ESI) *m*/*z*: [M + H]^+^ calcd for C_15_H_12_ON_3_, 250.0975; found, 250.0970.

### 10-Methoxy-7*H*-pyrido[2,3-*c*]carbazole (**9g**)

The reaction was carried out according to the general procedure B using 6-bromoquinoline (104 mg, 0.50 mmol, 1.0 equiv) and 2-chloro-4-methoxyaniline (95 mg, 0.60 mmol, 1.2 equiv) as starting materials. The crude residue was purified by silica gel column chromatography, petroleum ether (40–60 °C):EtOAc (1:1), to obtain compound **9g** in 65% yield (80 mg, off-white solid).

^1^H NMR ((CD_3_)_2_SO, 400 MHz): δ 11.77 (s, 1H, −*N*H), 9.17 (d, 1H, *J* = 8.3 Hz, *H*^1^), 8.82 (dd, 1H, *J* = 4.2, 1.4 Hz, *H*^3^), 8.02 (d, 1H, *J* = 2.3 Hz, *H*^11^), 7.99–7.92 (m, 2H, *H*^5,6^), 7.67 (dd, 1H, *J* = 8.3, 4.2 Hz, *H*^2^), 7.58 (d, 1H, *J* = 8.8 Hz, *H*^8^), 7.11 (dd, 1H, *J* = 8.8, 2.3 Hz, *H*^9^), 3.95 (s, 3H, −OC*H_3_*). ^13^C{^1^H} NMR ((CD_3_)_2_SO, 101 MHz): δ 153.8 (*C*^10^), 146.1 (*C*^3^), 144.2 (*C*^4a^), 137.4 (*C*^6a^), 133.9 (*C*^7a^), 130.8 (*C*^1^), 127.4 (*C*^5^), 124.4 (*C*^11c^), 123.1 (*C*^11a^), 121.5 (*C*^2^), 116.9 (*C*^6^), 114.2 (*C*^9^), 113.3 (*C*^11b^), 112.6 (*C*^8^), 103.9 (*C*^11^), 55.8 (−O*C*H_3_).

IR *v̅*_max_ (cm^–1^): 3146 (N–H stretch), 2997 (C–H stretch), 1571 (C=N stretch), 1534 (C=C stretch), 1493 (C=C stretch), 1227 (C–O stretch), 1035 (C–O stretch).

HRMS (ESI) *m*/*z*: [M + H]^+^ calcd for C_16_H_13_ON_2_, 249.1022; found, 249.1026.

### 8-Methoxy-11*H*-pyrido[4,3-*a*]carbazole (**9h**)

The reaction was carried out according to the general procedure B using 5-bromoisoquinoline (104 mg, 0.50 mmol, 1.0 equiv) and 2-chloro-4-methoxyaniline (95 mg, 0.60 mmol, 1.2 equiv) as starting materials. The crude residue was purified by silica gel column chromatography, hexane:EtOAc (3:7), to obtain compound **9h** in 90% yield (111 mg, gray solid).

^1^H NMR ((CD_3_)_2_SO, 400 MHz): δ 12.30 (s, 1H, −N*H*), 9.36 (s, 1H, *H*^4^), 8.62 (d, 1H, *J* = 5.7 Hz, *H*^2^), 8.38 (d, 1H, *J* = 8.5 Hz, *H*^6^), 8.32 (d, *J* = 5.7 Hz, 1H, *H*^1^), 7.81 (d, 1H, *J* = 2.3 Hz, *H*^7^), 7.75 (d, 1H, *J* = 8.5 Hz, *H*^5^), 7.60 (d, 1H, *J* = 8.8 Hz, *H*^10^), 7.13 (dd, 1H, *J* = 8.8, 2.3, *H*^9^), 3.89 (s, 3H, −OC*H_3_*). ^13^C{^1^H} NMR ((CD_3_)_2_SO, 101 MHz): δ 153.7 (*C*^8^), 152.0 (*C*^4^), 142.8 (*C*^2^), 134.1 (*C*^10a^), 133.7 (*C*^4a^), 126.6 (*C*^11a^), 124.1 (*C*^11b^), 123.0 (*C*^6b^), 121.2 (*C*^6^), 120.3 (*C*^6a^), 117.4 (*C*^5^), 115.7 (*C*^9^), 114.9 (*C*^1^), 112.5 (*C*^10^), 102.3 (*C*^7^), 55.6 (−O*C*H_3_).

IR *v̅*_max_ (cm^–1^): 3149 (N–H stretch), 1619 (C=N stretch), 1480 (C=C stretch), 1439 (C=C stretch), 1215 (C–O stretch), 1027 (C–O stretch).

HRMS (ESI) *m*/*z*: [M + H]^+^ calcd for C_16_H_13_ON_2_, 249.1022; found, 249.1020.

### 8-Me*t*hoxy-11*H*-pyrido[3,4-*a*]carbazole (**9i**)

The reaction was carried out according to the general procedure B using 8-bromoisoquinoline (104 mg, 0.50 mmol, 1.0 equiv) and 2-chloro-4-methoxyaniline (95 mg, 0.60 mmol, 1.2 equiv) as starting materials. The crude residue was purified by silica gel column chromatography, hexane:EtOAc (3:7), to obtain **9i** in 94% yield (104 mg, gray solid).

^1^H NMR ((CD_3_)_2_SO, 400 MHz): δ 12.39 (s, 1H, −N*H*), 9.86 (s, 1H, *H*^1^), 8.55 (d, 1H, *J* = 5.6 Hz, *H*^3^), 8.48 (d, 1H, *J* = 8.5 Hz, *H*^6^), 7.91 (d, 1H, *J* = 5.6 Hz, *H*^4^), 7.79 (d, 1H, *J* = 2.5 Hz, *H*^7^), 7.61 (d, 1H, *J* = 8.5 Hz, *H*^5^), 7.59 (d, 1H, *J* = 8.8 Hz, *H*^10^), 7.10 (dd, 1H, *J* = 8.8, 2.5, *H*^9^), 3.89 (s, 3H, −OC*H_3_*). ^13^C{^1^H} NMR ((CD_3_)_2_SO, 101 MHz): δ 153.8 (*C*^8^), 146.4 (*C*^1^), 142.6 (*C*^3^), 134.7 (*C*^4a^), 134.4 (*C*^11a^), 133.7 (*C*^10a^), 124.6 (*C*^6^), 123.2 (*C*^6b^), 121.1 (*C*^4^), 118.8 (*C*^6a^), 116.8 (*C*^11b^), 116.6 (*C*^5^), 114.9 (*C*^9^), 112.3 (*C*^10^), 102.2 (*C*^7^), 55.6 (−O*C*H_3_).

IR *v̅*_max_ (cm^–1^): 3074 (N–H stretch), 2962 (C–H stretch), 1627 (C=N stretch), 1474 (C=C stretch), 1437 (C=C stretch), 1217 (C–O stretch), 1027 (C–O stretch).

HRMS (ESI) *m*/*z*: [M + H]^+^ calcd for C_16_H_13_ON_2_, 249.1022; found, 249.1032.

### 9-Methoxy-3,6-dihydro-1*H*-furo[3,4-*c*]carbazole (**9j**)

The reaction was carried out according to the general procedure A using 5-bromo-1,3-dihydroisobenzofuran (99 mg, 0.50 mmol, 1.0 equiv) and 2-chloro-4-methoxyaniline (95 mg, 0.60 mmol, 1.2 equiv) as starting materials. The crude residue was purified by reversed-phase semi-preparative HPLC over a 5–95% gradient B, (solvent A: 0.1% TFA in water, solvent B: 0.1% TFA in acetonitrile) to obtain compound **9j** in 10% yield (35 mg, white solid).

^1^H NMR ((CD_3_)_2_SO, 400 MHz): δ 11.15 (s, 1H, −N*H*), 7.41 (d, 1H, *J* = 8.8 Hz, *H*^7^), 7.38 (d, 1H, *J* = 8.3 Hz, *H*^5^), 7.30 (d, 1H, *J* = 2.6 Hz, *H*^10^), 7.29 (d, 1H, *J* = 8.3 Hz, *H*^4^), 7.06 (dd, 1H, *J* = 8.8, 2.6 Hz, *H*^8^), 5.52 (t, 2H, *J* = 2.2 Hz, 2 × *H*^1^), 5.14 (t, 2H, *J* = 2.2 Hz, 2 × *H*^3^), 3.84 (s, 3H, −OC*H_3_*). ^13^C{^1^H} NMR ((CD_3_)_2_SO, 101 MHz): δ 153.1 (*C*^9^), 140.0 (*C*^5a^), 134.8 (*C*^6a^), 131.5 (*C*^10c^), 128.2 (*C*^3a^), 121.6 (*C*^10a^), 118.0 (*C*^4^), 116.0 (*C*^10b^), 114.6 (*C*^8^), 111.6 (*C*^7^), 110.0 (*C*^5^), 104.2 (*C*^10^), 72.8 (*C*^3^), 72.4 (*C*^1^), 55.7 (−O*C*H_3_).

IR *v̅*_max_ (cm^–1^): 3246 (N–H stretch), 2867 (C–H stretch), 1478 (C=C stretch), 1439 (C=C stretch), 1214 (C–O stretch), 1014 (C–O stretch).

HRMS (ESI) *m*/*z*: [M + H]^+^ calcd for C_15_H_14_O_2_N, 240.1019; found, 240.1030.

### 10-Methoxybenzo[4,5]imidazo[1,2-*a*]quinolone (**9k**)

The reaction was carried out according to the general procedure B using 2-bromoquinoline (104 mg, 0.50 mmol, 1.0 equiv) and 2-chloro-4-methoxyaniline (95 mg, 0.60 mmol, 1.2 equiv) as starting materials. The crude residue was purified by silica gel column chromatography, hexane:EtOAc (3:7), to obtain compound **9k** in 25% yield (31 mg, brown solid).

^1^H NMR ((CD_3_)_2_SO, 400 MHz): δ 8.78 (d, *J* = 8.5 Hz, 1H, *H*^1^), 8.08 (d, 1H, *J* = 2.3 Hz, *H*^11^), 8.03 (dd, 1H, *J* = 7.8, 1.5 Hz, *H*^4^), 7.88–7.82 (m, 3H, *H*^2,5,8^), 7.59 (d, 1H, *J* = 9.5 Hz, *H*^6^), 7.59–7.54 (m, 1H, *H*^3^), 7.21 (dd, 1H, *J* = 8.9, 2.3 Hz, *H*^9^), 4.00 (s, 3H, −OC*H_3_*). ^13^C{^1^H} NMR ((CD_3_)_2_SO, 101 MHz): δ 156.0 (*C*^10^), 147.1 (*C*^6a^), 138.8 (*C*^7a^), 134.8 (*C*^12a^), 130.7 (*C*^11a^), 130.4 (*C*^5^), 130.0 (*C*^2^), 129.5 (*C*^4^), 124.4 (*C*^3^), 123.0 (*C*^4a^), 120.3 (*C*^8^), 117.5 (*C*^6^), 115.6 (*C*^1^), 113.8 (*C*^9^), 98.3 (*C*^11^), 56.1 (−O*C*H_3_).

IR *v̅*_max_ (cm^–1^): 1636 (C=N stretch), 1608 (C=N stretch), 1487 (C=C stretch), 1448 (C=C stretch), 1219 (C–O stretch), 1026 (C–O stretch).

HRMS (ESI) *m*/*z*: [M + H]^+^ calcd for C_16_H_13_ON_2_, 249.1022; found, 249.1026.

### *N*-(2-Chloro-4-methoxyphenyl)-*N*-(quinolin-2-yl)quinolin-2-amine (**22**)

The reaction was carried out according to the general procedure B using 2-bromoquinoline (104 mg, 0.50 mmol, 1.0 equiv) and 2-chloro-4-methoxyaniline (95 mg, 0.60 mmol, 1.2 equiv) as starting materials. The crude residue was purified by silica gel column chromatography, hexane:EtOAc (3:7) to obtain compound **22** in 26% yield (54 mg, brown solid).

^1^H NMR ((CD_3_)_2_SO, 400 MHz): δ 8.22 (d, 2H, *J* = 9.0 Hz, 2 × *H*^4^), 7.88 (d, 2H, *J* = 8.0 Hz, 2 × *H*^5^), 7.66–7.59 (m, 4H, 2 × *H*^7,8^), 7.45 (ddd, 2H, *J* = 8.1, 5.5, 2.8 Hz, 2 × *H*^6^), 7.38 (d, 1H, *J* = 8.8 Hz, *H*^6′^), 7.28 (d, 2H, *J* = 8.9 Hz, 2 × *H*^3^), 7.24 (d, 1H, *J* = 2.9 Hz, *H*^3′^), 7.07 (dd, 1H, *J* = 8.8, 2.9 Hz, *H*^5′^), 3.87 (s, 3H, −OC*H_3_*). ^13^C{^1^H} NMR ((CD_3_)_2_SO, 101 MHz): δ 158.9 (*C*^4′^), 155.3 (2 × *C*^2^), 146.4 (2 × *C*^8a^), 137.3 (2 × *C*^4^), 133.5 (*C*^1′, 2′^), 132.6 (*C*^6′^), 129.7 (2 × *C*^7^), 127.6 (2 × *C*^5^), 127.2 (2 × *C*^8^), 125.2 (2 × *C*^4a^), 124.7 (2 × *C*^6^), 116.6 (2 × *C*^3^), 115.5 (*C*^3′^), 114.6 (*C*^5′^), 55.8 (−O*C*H_3_).

IR *v̅*_max_ (cm^–1^): 1597 (C=N stretch), 1575 (C=N stretch), 1502 (C=C stretch), 1428 (C=C stretch), 1230 (C–O stretch), 1040 (C–O stretch).

HRMS (ESI) *m*/*z*: [M + H]^+^ calcd for C_25_H_19_ON_3_Cl, 412.1211; found, 412.1230.

### 10-Methoxybenzo[4,5]imidazo[1,2-*a*]quinoxaline (**9l**)

The reaction was carried out according to the general procedure B using 2-bromoquinoxaline (105 mg, 0.50 mmol, 1.0 equiv) and 2-chloro-4-methoxyaniline (95 mg, 0.60 mmol, 1.2 equiv) as starting materials. The crude residue was purified by silica gel column chromatography, hexane:EtOAc (3:7), to obtain compound **9l** in 14% yield (17 mg, white solid).

^1^H NMR ((CD_3_)_2_SO, 400 MHz): δ 9.23 (s, 1H, *H*^6^), 8.81 (d, 1H, *J* = 8.3 Hz, *H*^1^), 8.14 (dd, 1H, *J* = 9.0, 1.4 Hz, *H*^4^), 8.10 (d, 1H, *J* = 2.3 Hz, *H*^11^), 8.00 (d, 1H, *J* = 9.0 Hz, *H*^8^), 7.87 (ddd, 1H, *J* = 8.3, 7.6, 1.4 Hz, *H*^2^), 7.69 (ddd, 1H, *J* = 8.0, 7.6, 1.0 Hz, *H*^3^), 7.33 (dd, 1H, *J* = 9.0, 2.3 Hz, *H*^9^), 4.04 (s, 3H, −OC*H_3_*). ^13^C{^1^H} NMR ((CD_3_)_2_SO, 101 MHz): δ 157.6 (*C*^10^), 146.1 (*C*^6^), 140.5 (*C*^6a^), 138.5 (*C*^7a^), 135.2 (*C*^4a^), 130.2 (*C*^4^), 130.0 (*C*^2, 11a^), 129.1 (*C*^12a^), 125.7 (*C*^3^), 122.1 (*C*^8^), 116.3 (*C*^9^), 115.9 (*C*^1^), 97.4 (*C*^11^), 56.2 (−O*C*H_3_).

IR *v̅*_max_ (cm^–1^): 2843 (C–H stretch), 1580 (C=N stretch), 1590 (C=N stretch), 1463 (C=C stretch), 1225 (C–O stretch), 1026 (C–O stretch).

HRMS (ESI) *m*/*z*: [M + H]^+^ calcd for C_15_H_12_ON_3_, 250.0975; found, 250.0982.

### 8-Methyl-7*H*-pyrido[3,4-*c*]carbazole (**9m**)

The reaction was carried out according to the general procedure B using 6-bromoisoquinoline (104 mg, 0.50 mmol, 1.0 equiv) and 2-chloro-6-methylaniline (85 mg, 0.60 mmol, 1.2 equiv) as starting materials. The crude residue was purified by silica gel column chromatography, DCM:MeOH (0 to 2% MeOH), to obtain compound **9m** in 62% yield (72 mg, white solid).

^1^H NMR ((CD_3_)_2_SO, 400 MHz): δ 12.01 (s, 1H, −N*H*), 9.32 (s, 1H, *H*^4^), 8.63 (d, 1H, *J* = 5.8 Hz, *H*^2^), 8.56 (d, 1H, *J* = 5.7 Hz, *H*^1^), 8.42 (dd, 1H, *J* = 7.2, 2.4 *H*^11^), 8.06 (d, 1H, *J* = 8.9 Hz, *H*^5^), 7.91 (d, 1H, *J* = 8.9 Hz, *H*^6^), 7.31–7.24 (m, 2H, *H*^9^,*H*^10^), 2.65 (s, 3H, −C*H_3_*). ^13^C{^1^H} NMR ((CD_3_)_2_SO, 101 MHz): δ 152.2 (*C*^4^), 144.1 (*C*^2^), 139.6 (*C*^6a^), 138.1 (*C*^7a^), 132.0 (*C*^11c^), 125.9 (*C*^9^), 125.2 (*C*^5^), 123.9 (*C*^4a^), 122.4 (*C*^11a^), 121.1 (*C*^11^), 120.3 (*C*^8^), 119.1 (*C*^10^), 116.3 (*C*^1^), 114.9 (*C*^6^), 112.8 (*C*^11b^), 17.5 (−*C*H_3_).

HRMS (ESI) *m*/*z*: [M + H]^+^ calcd for C_16_H_13_N_2_, 233.1073; found, 233.1070.

### Glycozoline (**9n**)^1^

#### Preparation 1

The reaction was carried out according to the general procedure A using 4-bromotoluene (85 mg, 0.50 mmol, 1.0 equiv) and 2-chloro-4-methoxyaniline (95 mg, 0.60 mmol, 1.2 equiv) as starting materials. The crude residue was purified by silica gel column chromatography, petroleum ether (40–60 °C):EtOAc (100:6), to obtain compound **9m** in 36% yield (38 mg, brown crystalline solid).

#### Preparation 2

The reaction was carried out according to the general procedure A using 4-bromotoluene (85 mg, 0.50 mmol, 1.0 equiv) and 2-chloro-4-methoxyaniline (95 mg, 0.60 mmol, 1.2 equiv) as starting materials with the addition of PivOH (15.3 mg, 0.15 mmol, 0.3 equiv). The crude residue was purified by silica gel column chromatography, petroleum ether (40–60 °C):EtOAc (100:6) to obtain compound **9n** in 64% yield (66 mg, brown crystalline solid).

^1^H NMR ((CD_3_)_2_SO, 400 MHz): δ 10.85 (s, 1H, −N*H*), 7.87 (d, 1H, *J* = 1.0 Hz, *H*^5^), 7.61 (d, 1H, *J* = 2.5 Hz, *H*^4^), 7.34 (d, 1H, *J* = 8.8 Hz, *H*^1^), 7.32 (d, 1H, *J* = 8.3 Hz, *H*^8^), 7.16 (dd, 1H, *J* = 8.3, 1.0 Hz, *H*^7^), 6.98 (dd, 1H, *J* = 8.8, 2.5 Hz, *H*^2^), 3.83 (s, 3H, −OC*H_3_*), 2.45 (s, 3H, −C*H_3_*). ^13^C{^1^H} NMR ((CD_3_)_2_SO, 101 MHz): δ 152.8 (*C*^3^), 138.6 (*C*^8a^), 134.8 (*C*^9a^), 126.7 (*C*^6, 7^), 126.5 (*C*^4a^), 122.5 (*C*^4b^), 119.9 (*C*^5^), 114.5 (*C*^2^), 111.5 (*C*^1^), 110.6 (*C*^8^), 102.8 (*C*^4^), 55.5 (−O*C*H_3_), 21.0 (−*C*H_3_).

IR *v̅*_max_ (cm^–1^): 3401 (N–H stretch), 3003 (C–H stretch), 2835 (C–H stretch), 1474 (C=C stretch), 1458 (C=C stretch), 1212 (C–O stretch), 1037 (C–O stretch).

HRMS (ESI) *m*/*z*: [M + H]^+^ calcd for C_14_H_14_ON, 212.1070; found, 212.1079.

### Harmane (**9o**)^48^

The reaction was carried out according to the general procedure A using 3-bromo-2-methylpyridine (86 mg, 0.50 mmol, 1.0 equiv) and 2-chloroaniline (77 mg, 0.60 mmol, 1.2 equiv) as starting materials. The crude residue was purified by silica gel column chromatography, EtOAc (100%), to obtain compound **9o** in 45% yield (40 mg, purple solid).

^1^H NMR ((CD_3_)_2_SO, 400 MHz): δ 11.53 (s, 1H, N*H*), 8.20 (d, 1H, *J* = 5.3 Hz, *H*^3^), 8.18 (d, 1H, *J* = 8.0 Hz, *H*^5^), 7.92 (d, 1H, *J* = 5.3 Hz, *H*^4^), 7.61–7.57 (m, 1H, *H*^8^), 7.52 (ddd, 1H, *J* = 8.3, 7.0, 1.2 Hz, *H*^7^), 7.22 (ddd, 1H, *J* = 8.0, 7.0, 1.2 Hz, *H*^6^), 2.76 (s, 3H, −C*H_3_*). ^13^C{^1^H} NMR ((CD_3_)_2_SO, 101 MHz): δ 142.1 (*C*^1^), 140.3 (*C*^8a^), 137.5 (*C*^3^), 134.4 (*C*^9a^), 127.7 (*C*^7^), 126.8 (*C*^4a^), 121.7 (*C*^5^), 121.0 (*C*^4b^), 119.1 (*C*^6^), 112.6 (*C*^4^), 111.9 (*C*^8^), 20.4 (−*C*H_3_).

IR *v̅*_max_ (cm^–1^): 3062 (N–H stretch), 2954 (C–H stretch), 1627 (C=N stretch), 1571 (C=C stretch), 1506 (C=C stretch).

HRMS (ESI) *m*/*z*: [M + H]^+^ calcd for C_12_H_11_N_2_, 183.0917; found, 183.0927.

### Murrayafoline A (**9p**)

The reaction was carried out according to the general procedure A using 2-bromo-5-methylanisole (101 mg, 0.50 mmol, 1.0 equiv) and 2-chloroaniline (77 mg, 0.60 mmol, 1.2 equiv) as starting materials with the addition of PivOH (15.3 mg, 0.15 mmol, 0.3 equiv). The crude residue was purified by silica gel column chromatography, hexane:EtOAc (7:3), to obtain compound **9p** in 43% yield (45 mg, brown crystalline solid).

^1^H NMR ((CD_3_)_2_SO, 400 MHz): δ 11.12 (s, 1H, −N*H*), 8.00 (d, 1H, *J* = 8.0 Hz, *H*^5^), 7.49–7.46 (m, 1H, *H*^4^), 7.45–7.41 (m, 1H, *H*^8^), 7.32 (ddd, 1H, *J* = 8.3, 7.1, 1.3 Hz, *H*^7^), 7.09 (ddd, 1H, *J* = 8.0, 7.1, 1.1 Hz, *H*^6^), 6.82 (d, 1H, *J* = 1.1 Hz, *H*^2^), 3.96 (s, 3H, −OC*H_3_*), 2.46 (s, 3H, −C*H_3_*).

^13^C{^1^H} NMR ((CD_3_)_2_SO, 101 MHz): δ 145.3 (*C*^1^), 139.7 (*C*^8a^), 128.0 (*C*^9a^), 127.8 (*C*^3^), 125.0 (*C*^7^), 123.4 (*C*^4a/4b^), 122.4 (*C*^4a/4b^), 120.0 (*C*^5^), 118.2 (*C*^6^), 112.2 (*C*^4^), 111.2 (*C*^8^), 107.7 (*C*^2^), 55.2 (−O*C*H_3_), 21.5 (−*C*H_3_).

IR *v̅*_max_ (cm^–1^): 3418 (N–H stretch), 2921 (C–H stretch), 1590 (C=C stretch), 1454 (C=C stretch), 1232 (C–O stretch), 1040 (C–O stretch).

HRMS (ESI) *m*/*z*: [M + H]^+^ calcd for C_14_H_14_ON, 212.1070; found, 212.1070.

### 2-((5,8-Dimethyl-9*H*-carbazol-3-yl)oxy)-*N*,*N*-dimethylethan-1-amine (**9p**)

The reaction was carried out according to the general procedure B using 2-bromo-*p*-xylene (93 mg, 0.50 mmol, 1.0 equiv) and 2-chloro-4-(2-(dimethylamino)ethoxy)aniline (128 mg, 0.60 mmol, 1.2 equiv) as starting materials and HPtBu_3_BF_4_ (14.5 mg, 10 mol %). The crude residue was purified by silica gel column chromatography, DCM:MeOH (100:3) to obtain compound **9p** in 22% yield (31 mg, brown solid).

^1^H NMR ((CD_3_)_2_SO, 400 MHz): δ 11.04 (s, −N*H*), 7.67 (d, 1H, *J* = 2.4 Hz, *H*^4^), 7.46 (d, 1H, *J* = 8.7 Hz, *H*^1^), 7.12 (dd, 1H, *J* = 8.7, 2.4 Hz, *H*^2^), 7.06 (d, 1H, *J* = 7.2 Hz, *H*^7^), 6.82 (d, 1H, *J* = 7.2 Hz, *H*^6^), 4.40 (t, 2H, *J* = 5.1 Hz, *H*^2′^), 3.44 (t, 2H, *J* = 5.1 Hz, *H*^1′^), 2.82 (s, 6H, −N(C*H_3_*)_2_), 2.77 (s, 3H, C^5^–C*H_3_*), 2.50 (s, 3H, C^8^–C*H_3_*). ^13^C{^1^H} NMR ((CD_3_)_2_SO, 101 MHz): δ 151.1 (*C*^3^), 139.8 (*C*^8a^), 135.2 (*C*^9a^), 129.6 (*C*^5^), 125.8 (*C*^7^), 123.6 (*C*^4a^), 120.3 (*C*^4b^), 119.6 (*C*^6^), 117.5 (*C*^8^), 114.1 (*C*^2^), 111.4 (*C*^1^), 107.0 (*C*^4^), 63.7 (*C*^2′^), 56.0 (*C*^1′^), 41.2 ((−N*C*H_3_)_2_), 20.2 (C^5^–*C*H_3_), 16.7 (C^8^–*C*H_3_).

IR *v̅*_max_ (cm^–1^): 3857 (N–H stretch), 2925 (C–H stretch), 1523 (C=C stretch), 1465 (C–H stretch), 1057 (C–O stretch).

HRMS (ESI) *m*/*z*: [M + H]^+^ calcd for C_18_H_23_ON_2_, 283.1805; found, 283.1812.

### 5*H*-Benzo[*b*]carbazole-6,11-dione (**21a**)

The reaction was carried out according to the general procedure A using 2-bromonaphthalene-1,4-dione (119 mg, 0.50 mmol, 1.0 equiv) and 2-chloroaniline (77 mg, 0.60 mmol, 1.2 equiv) as starting materials. The crude residue was purified by silica gel column chromatography, CHCl_3_ (100%), to obtain compound **21a** in 73% yield (90 mg, orange solid).

^1^H NMR ((CD_3_)_2_SO, 400 MHz): δ 13.08 (s, 1H, −N*H*), 8.22 (d, 2H, *J* = 8.0 Hz, *H*^1^), 8.12 (ddd, 2H, *J* = 7.4, 7.4, 1.5 Hz, *H*^7, 10^), 7.87 (ddd, 1H, *J* = 7.4, 7.4, 1.5 Hz, *H*^9^), 7.82 (ddd, 1H, *J* = 7.4, 7.4, 1.5 Hz, *H*^8^), 7.61 (d, 1H, *J* = 8.3 Hz, *H*^4^), 7.46 (ddd, 1H, *J* = 8.3, 7.0, 1.2 Hz, *H*^3^), 7.33–7.41 (m, 1H, *H*^2^). ^13^C{^1^H} NMR ((CD_3_)_2_SO, 101 MHz): δ 180.4 (*C*^11^), 177.6 (*C*^6^), 138.2 (*C*^4a^), 137.2 (*C*^5a^), 134.3 (*C*^9^), 134.1 (*C*^10a^), 133.2 (*C*^8^), 132.6 (*C*^6a^), 127.0 (*C*^3^), 126.1 (*C*^10^), 126.0 (*C*^7^), 124.0 (*C*^11b^), 123.9 (*C*^2^), 122.4 (*C*^1^), 117.4 (*C*^11a^), 113.9 (*C*^4^).

IR *v̅*_max_ (cm^–1^): 3263 (N–H stretch), 1649 (C=O stretch), 1590 (C=C stretch), 1474 (C=C stretch).

HRMS (ESI) *m*/*z*: [M + H]^+^ calcd for C_16_H_10_O_2_N, 248.0706; found, 248.0703.

### 2-Methoxy-5*H*-benzo[*b*]carbazole-6,11-dione (**21b**)

The reaction was carried out according to the general procedure A using 2-bromonaphthalene-1,4-dione (119 mg, 0.50 mmol, 1 equiv) and 2-chloro-4-methoxyaniline (94 mg, 0.60 mmol, 1.2 equiv) as starting materials. The crude residue was purified by silica gel column chromatography, petroleum ether (40–60 °C):EtOAc:NEt_3_ (70:30:1), to obtain compound **21b** in 79% yield (93 mg, orange solid).

^1^H NMR ((CD_3_)_2_SO, 400 MHz): δ 13.02 (s, 1H, −N*H*), 8.15–8.08 (m, 2H, *H*^7,10^), 7.86 (dd, 1H, *J* = 7.5, 1.5 Hz, *H*^9^), 7.81 (dd, 1H, *J* = 7.4, 1.7 Hz, *H*^8^), 7.63 (d, 1H, *J* = 2.5 Hz, *H*^1^), 7.51 (d, 1H, *J* = 9.1 Hz, *H*^4^), 7.10 (dd, 1H, *J* = 9.0, 2.6 Hz, *H*^3^), 3.86 (s, 3H, −OC*H_3_*). ^13^C{^1^H} NMR ((CD_3_)_2_SO, 101 MHz): δ 180.2 (*C*^11^), 177.2 (*C*^6^), 157.0 (*C*^2^), 137.0 (*C*^5a^), 134.2 (*C*^9, 10a^), 133.4 (*C*^4a^), 133.1 (*C*^8^), 132.7 (*C*^6a^), 126.0 (*C*^7, 10^), 124.8 (*C*^11b^), 118.4 (*C*^3^), 117.0 (*C*^11a^), 115.0 (*C*^4^), 102.1 (*C*^1^), 55.4 (−O*C*H_3_).

IR *v̅*_max_ (cm^–1^): 3195 (N–H stretch), 1673 (C=O stretch), 1644 (C=O stretch), 1592 (C=C stretch), 1530 (C=O stretch), 1223 (C–O stretch), 1020 (C–O stretch).

HRMS (ESI) *m*/*z*: [M + H]^+^ calcd for C_15_H_12_ON_3_, 278.0812; found, 278.0812.

### 2-Methyl-5*H*-benzo[*b*]carbazole-6,11-dione (**21c**)

The reaction was carried out according to the general procedure A using 2-bromonaphthalene-1,4-dione (119 mg, 0.50 mmol, 1.0 equiv) and 4-methyl-2-chloroaniline (85 mg, 0.60 mmol, 1.2 equiv) as starting materials. The crude residue was purified by silica gel column chromatography, CHCl_3_ (100%), to obtain compound **21c** in 59% yield (77 mg, orange solid).

^1^H NMR ((CD_3_)_2_SO, 400 MHz): δ 12.98 (s, 1H, −N*H*), 8.15–8.06 (m, 2H, *H*^7,10^), 8.01 (s, 1H, *H*^1^), 7.89–7.84 (m, 1H, *H*^9^), 7.84–7.78 (m, 1H, *H*^8^), 7.50 (d, 1H, *J* = 8.5 Hz, *H*^4^), 7.29 (d, 1H, *J =* 8.5 Hz, *H*^3^), 2.46 (s, 3H, −C*H_3_*). ^13^C{^1^H} NMR ((CD_3_)_2_SO, 101 MHz): δ 180.3 (*C*^11^), 177.5 (*C*^6^), 137.1 (*C*^5a^), 136.7 (*C*^4a^), 134.2 (*C*^10a^), 134.1 (*C*^9^), 133.4 (*C*^2^), 133.2 (*C*^8^), 132.7 (*C*^6a^), 128.9 (*C*^3^), 126.1 (*C*^10^), 126.0 (*C*^7^), 124.2 (*C*^11b^), 121.6 (*C*^1^), 116.9 (*C*^11a^), 113.5 (*C*^4^), 21.3 (−*C*H_3_).

IR *v̅*_max_ (cm^–1^): 3174 (N–H stretch), 1670 (C=O stretch), 1642 (C=O stretch), 1595 (C=C stretch), 1538 (C=C stretch).

HRMS (ESI) *m*/*z*: [M + H]^+^ calcd for C_17_H_12_O_2_N, 262.0863; found, 262.0861.

### 2-(Trifluoromethyl)-5*H*-benzo[*b*]carbazole-6,11-dione (**21d**)

The reaction was carried out according to the general procedure A using 2-bromonaphthalene-1,4-dione (119 mg, 0.50 mmol, 1.0 equiv) and 4-trifluoromethyl-2-chloroaniline (117 mg, 0.60 mmol, 1.2 equiv) as starting materials. The crude residue was purified by silica gel column chromatography, petroleum ether (40–60 °C):EtOAc:NEt_3_ (100:20:4), to obtain compound **21d** in 35% yield (54 mg, orange solid).

^1^H NMR ((CD_3_)_2_SO, 400 MHz): δ 13.44 (s, 1H, −N*H*), 8.49–8.45 (m, 1H, *H*^1^), 8.16–8.08 (m, 2H, *H*^7, 10^), 7.91–7.81 (m, 2H, *H*^8, 9^), 7.80–7.71 (m, 2H, *H*^3, 4^). ^13^C{^1^H} NMR ((CD_3_)_2_SO, 101 MHz): δ 180.3 (*C*^11^), 177.5 (*C*^6^), 139.5 (*C*^4a^), 139.0 (*C*^5a^), 134.5 (*C*^8^), 133.8 (*C*^9^), 133.5 (*C*^7a^), 132.5 (*C*^10a^), 126.2 (*C*^7, 10^), 124.7 (q, *J* = 272.9 Hz, −*C*F_3_), 124.3 (q, *J* = 31.8 Hz, *C*^2^), 123.1 (*C*^11b^), 123.0 (q, *J* = 3.4 Hz, *C*^3^), 119.6 (q, *J* = 4.3 Hz, *C*^1^), 117.6 (*C*^11a^), 115.1 (*C*^4^). ^19^F NMR ((CD_3_)_2_SO, 376 MHz): δ −59.7.

IR *v̅*_max_ (cm^–1^): 3234 (N–H stretch), 1655 (C=O stretch), 1631 (C=O stretch), 1586 (C=C stretch), 1541 (C=C stretch).

HRMS (ESI) *m*/*z*: [M + H]^+^ calcd for C_17_H_9_O_2_NF_3_, 316.0580; found, 316.0585.

### 3-Methoxy-5*H*-benzo[*b*]carbazole-6,11-dione (**21e**)

The reaction was carried out according to the general procedure A using 2-bromonaphthalene-1,4-dione (119 mg, 0.5 mmol, 1.0 equiv) and 2-chloro-5-methoxyaniline (95 mg, 0.60 mmol, 1.2 equiv) as starting materials. The crude residue was purified by silica gel column chromatography, petroleum ether (40–60 °C):EtOAc (9:1), to obtain compound **21e** in 68% yield (94 mg, red solid).

^1^H NMR ((CD_3_)_2_SO, 400 MHz): δ 12.89 (s, 1H, −N*H*), 8.11–8.03 (m, 3H, *H*^1, 7, 10^), 7.87–7.76 (m, 2H, *H*^8, 9^), 7.00 (dd, 1H, *J* = 8.8, 2.0 Hz, *H*^2^), 6.98 (d, 1H, *J* = 2.0 Hz, *H*^4^), 3.85 (s, 3H, −OC*H_3_*). ^13^C{^1^H} NMR ((CD_3_)_2_SO, 101 MHz): δ 180.5 (*C*^11^), 176.8 (*C*^6^), 159.4 (*C*^3^), 139.8 (*C*^4a^), 136.4 (*C*^5a^), 134.0 (*C*^6a^), 133.9 (*C*^8^), 133.2 (*C*^9^), 132.7 (*C*^10a^), 126.0 (*C*^7/10^), 125.9 (*C*^7/10^), 123.2 (*C*^1^), 118.0 (*C*^11a/11b^), 117.9 (*C*^11a/11b^), 115.5 (*C*^2^), 95.1 (*C*^4^), 55.4 (−O*C*H_3_).

IR *v̅*_max_ (cm^–1^): 3189 (N–H stretch), 2927 (C–H stretch), 1664 (C=O stretch), 1629 (C=O stretch), 1532 (C=C stretch).

HRMS (ESI) *m*/*z*: [M + H]^+^ calcd for C_17_H_12_O_3_N, 278.0812; found, 278.0809.

### 3-Fluoro-5*H*-benzo[*b*]carbazole-6,11-dione (**21f**)

The reaction was carried out according to the general procedure A using 2-bromonaphthalene-1,4-dione (119 mg, 0.50 mmol, 1.0 equiv) and 5-fluoro-2-chloroaniline (87 mg, 0.60 mmol, 1.2 equiv) as starting materials. The crude residue was purified by silica gel column chromatography, petroleum ether (40–60 °C):EtOAc:NEt_3_ (100:20:4) to obtain compound **21f** in 58% yield (76 mg, orange solid).

^1^H NMR ((CD_3_)_2_SO, 400 MHz): δ 13.12 (s, 1H, −N*H*), 8.19 (dd, 1H, *J* = 8.8, 5.5 Hz, *H*^1^), 8.11–8.05 (m, 2H, *H*^7, 10^), 7.87 (m, 2H, *H*^8, 9^), 7.29 (dd, 1H, *J* = 9.4, 2.2 Hz, *H*^4^), 7.27–7.20 (m, 1H, *H*^2^). ^13^C{^1^H} NMR ((CD_3_)_2_SO, 101 MHz): δ 180.3 (*C*^11^), 177.1 (*C*^6^), 161.5 (d, *J* = 243.5 Hz, *C*^3^), 138.6 (d, *J* = 13.2 Hz, *C*^4a^), 138.0 (*C*^5a^), 134.2 (*C*^8^), 133.8 (*C*^6a^), 133.3 (*C*^9^), 132.5 (*C*^10a^), 126.0 (*C*^7, 10^), 124.1 (d, *J* = 10.5 Hz, *C*^1^), 120.6 (*C*^11a/11b^), 117.4 (*C*^11a/11b^), 113.1 (d, *J* = 25.2 Hz, *C*^2^), 99.6 (d, *J* = 26.1 Hz, *C*^4^). ^19^F NMR ((CD_3_)_2_SO, 376 MHz): δ −113.2.

IR *v̅*_max_ (cm^–1^): 3230 (N–H stretch), 2928 (C–H stretch), 1647 (C=O stretch), 1588 (C=C stretch), 1472 (C=C stretch).

HRMS (ESI) *m*/*z*: [M + H]^+^ calcd for C_16_H_9_O_2_NF, 266.0612; found, 266.0609.

### 2-Methoxy-5-(4-methoxybenzyl)-5*H*-benzo[*b*]carbazole-6,11-dione (**24**)

2-Methoxy-5*H*-benzo[*b*]carbazole-6,11-dione (**21b**) (74 mg, 0.27 mmol, 1.0 equiv) was stirred in anhydrous DMF (8 mL) and cooled to 0 °C in an ice bath. NaH (16 mg, 0.66 mmol, 2.4 equiv) was added and stirred for 1 h. Next, benzyl bromide (0.078 mL, 0.66 mmol, 2.4 equiv) was added dropwise and the reaction was stirred for 2 h at rt. The reaction mixture was diluted with EtOAc, washed with 5% w/v aqueous solution of LiCl followed by water and brine, dried over Na_2_SO_4_, and filtered. The filtrate was then concentrated *in vacuo* and purified by silica gel flash column chromatography (petroleum ether (40–60 °C):EtOAc (10:1)) to afford the desired compound **24** in 83% yield (81 mg, orange solid).

^1^H NMR ((CD_3_)_2_SO, 500 MHz): δ 8.13 (dd, 1H, *J* = 7.5, 1.1 Hz, *H*^7^ or *H*^10^), 8.10 (dd, 1H, *J* = 7.5, 1.2 Hz, *H*^7^ or *H*^10^), 7.90–7.85 (m, 1H, *H*^9^), 7.85–7.80 (m, 1H, *H*^8^), 7.75 (d, 1H, *J* = 2.5 Hz, *H*^1^), 7.71 (d, 1H, *J* = 9.2 Hz, *H*^4^), 7.33–7.28 (m, 2H, 2 × *H*^2′^), 7.27–7.20 (m, 3H, 2 × *H*^3′^and *H*^4′^), 7.15 (dd, 1H, *J* = 9.2, 2.6 Hz, *H*^3^), 6.04 (s, 2H, −C*H_2_*), 3.88 (s, 3H, −OC*H_3_*). ^13^C{^1^H} NMR ((CD_3_)_2_SO, 125 MHz): δ 180.3 (*C*^11^), 177.9 (*C*^6^), 158.6 (*C*^4′^), 157.5 (*C*^2^), 134.6 (*C*^4a^), 134.4 (*C*^5a^), 134.2 (*C*^9^), 133.5 (*C*^8^), 133.3 (*C*^10a^), 133.2 (*C*^6a^), 128.6 (2 × *C*^2′^), 127.5 (*C*^4′^), 126.6 (2 × *C*^3′^), 126.3 (*C*^7^ or C^10^), 125.7 (*C*^7^ or C^10^), 124.2 (*C*^11b^), 118.8 (*C*^3^), 117.7 (*C*^11a^), 113.8 (*C*^4^), 102.3 (*C*^1^), 55.5 (Ar–O*C*H_3_), 47.8 (−*C*H_2_).

IR *v̅*_max_ (cm^–1^): 2926 (C–H stretch), 1647 (C=O stretch), 1489 (C–H bend), 1234 (C–O stretch).

HRMS (ESI) *m*/*z*: [M + H]^+^ calcd for C_24_H_18_O_3_N, 368.1281; found, 368.1284.

### 5-Benzyl-2-methoxy-6,11-dimethyl-5*H*-benzo[*b*]carbazole (**25**)

5-Benzyl-2-methoxy-5*H*-benzo[*b*]carbazole-6,11-dione (71 mg, 0.19 mmol, 1.0 equiv) was dissolved in anhydrous THF (7 mL) under an argon atmosphere. The reaction was cooled to −80 °C, and a 1.6 M MeLi in THF solution (0.55 mL, 0.87 mmol) was added dropwise over 5 min. The reaction mixture was stirred at −80 °C for 3 h. Next, the reaction was quenched with water and extracted with CHCl_3_. The combined organic phases were washed with water and brine, dried over Na_2_SO_4_, and concentrated *in vacuo*. The isolated crude solid was dissolved in anhydrous THF (5 mL) and added to a solution of SnCl_2_ (0.37 g, 1.94 mmol) in a mixture of 37% HCl (4.8 mL) and Et_2_O (4.8 mL). The reaction mixture was stirred vigorously for 5 h and then extracted with CHCl_3_. The combined organic phases were washed with water and brine, dried over Na_2_SO_4_, and the filtrate was concentrated *in vacuo*. The crude was purified by silica gel flash column chromatography, petroleum ether (40–60 °C):EtOAc (7:3), to afford the desired compound **25** in 40% yield (28 mg, cream solid).

^1^H NMR ((CD_3_)_2_SO, 400 MHz): δ 8.36 (dd, 1H, *J* = 8.8, 0.8 Hz, *H*^10^), 8.15 (dd, 1H, *J* = 8.9, 0.8 Hz, *H*^7^), 7.92 (d, 1H, *J* = 2.5 Hz, *H*^1^), 7.55–7.48 (m, 1H, *H*^8^), 7.48–7.42 (m, 1H, *H*^9^), 7.39 (d, 1H, *J* = 8.8 Hz, *H*^4^), 7.29 (m, 2H, 2 × *H*^3′^), 7.22 (m, 1H, *H*^4′^), 7.15 (dd, 1H, *J* = 8.8, 2.5 Hz, *H*^3^), 7.08 (m, 2H, 2 × *H*^2′^), 5.83 (s, 2H, −C*H_2_*), 3.90 (s, 3H, −OC*H_3_*), 3.20 (s, 3H, C^11^–C*H*_3_), 2.84 (s, 3H, C^6^–C*H*_3_). ^13^C{^1^H} NMR ((CD_3_)_2_SO, 101 MHz): δ 153.4 (*C*^2^), 139.5 (*C*^4a^), 139.2 (*C*^5a^), 139.1 (*C*^1′^), 131.8 (*C*^6a^), 128.7 (2 × *C*^3′^), 126.9 (*C*^4′,11a^), 126.7 (*C*^11^), 125.6 (2 × *C*^2′^), 124.8 (*C*^8^), 124.5 (*C*^10^), 123.7 (*C*^10a^), 123.4 (*C*^11b^), 123.3 (*C*^7^), 122.2 (*C*^9^), 114.4 (*C*^3^), 109.8 (*C*^4^), 109.6 (*C*^6^), 108.2 (*C*^1^), 55.8 (−O*C*H_3_), 49.0 (−*C*H_2_), 15.2 (C^11^–*C*H_3_), 13.6 (C^6^–*C*H_3_).

IR *v̅*_max_ (cm^–1^): 2922 (C–H stretch), 1599 (C=C stretch), 1437 (C–H bend), 1204 (C–O stretch).

HRMS (ESI) *m*/*z*: [M + H]^+^ calcd for C_27_H_25_O_2_N, 366.1852; found, 366.1854.

### 2-Methoxy-6,11-dimethyl-5*H*-benzo[*b*]carbazole (**26**)

The *N*-debenzylation protocol was based on a literature procedure.^49^ 5-Benzyl-2-methoxy-6,11-dimethyl-5*H*-benzo[*b*]carbazole (**25**) (10 mg, 0.027 mmol, 1 equiv) was dissolved in DMSO (0.5 mL) and added to a flame-dried vial. While stirring the solution at room temperature, KO^*t*^Bu (21 mg, 0.19 mmol, 7 equiv) in THF (0.5 mL) was added. An oxygen balloon was then bubbled into the solution for 30 min. After stirring at room temperature for 5 h, the reaction was quenched with a saturated ammonium chloride aqueous solution. The product was extracted three times with EtOAc. The combined organic phases were dried over Na_2_SO_4_, filtered, and concentrated *in vacuo*. The crude was purified by silica gel column chromatography using hexane:EtOAc (7:3) to afford the desired compound **26** in 61% yield (4.5 mg, yellow solid). Characterization of **26** is in agreement with that previously described in the literature.^5^

^1^H NMR ((CD_3_)_2_SO, 400 MHz): δ 10.83 (s, 1H, N*H*), 8.32 (dd, 1H, *J* = 8.7, 0.7 Hz, *H*^10^), 8.10 (d, 1H, *J* = 8.7, 0.7 Hz, *H*^7^), 7.86 (d, 1H, *J* = 2.5 Hz, *H*^1^), 7.51 (ddd, 1H, *J* = 8.7, 6.5, 1.3 Hz, *H*^8^), 7.45–7.39 (m, 2H, *H*^9,4^), 7.14 (dd, 1H, *J* = 8.7, 2.5 Hz, *H*^3^), 3.89 (s, 3H, Ar–OC*H*_3_), 3.16 (s, 3H, C^11^–C*H_3_*), 2.80 (s, 3H, C^6^–C*H*_3_). ^13^C{^1^H} NMR ((CD_3_)_2_SO, 101 MHz): δ 152.6 (C^2^), 138.9 (C^5a^), 137.5 (C^4a^), 130.6 (C^6a^), 126.4 (C^10a^), 125.8 (C^11^), 124.5 (C^10^), 124.3 (C^8^), 123.7 (C^11b^), 123.1 (C^7^), 122.7 (C^11a^), 121.5 (C^9^), 114.6 (C^3^), 110.7 (C^4^), 108.8 (C^1^), 107.7 (C^6^), 55.8 (Ar–O*C*H_3_), 15.1 (C^11^–CH_3_), 12.6 (C^6^–CH_3_).

LC-MS (ESI +ve mode): *m*/*z* = 276.1. Retention time = 8.19 min.

### 10-Methoxy-2-methyl-7*H*-pyrido[4,3-*c*]carbazol-2-ium (**27**)

To a solution of 10-methoxy-7*H*-pyrido[4,3-*c*]carbazole **9e** (80 mg, 0.32 mmol, 1.0 equiv) in DMF (1.3 mL) was added MeI (230 mg, 1.61 mmol, 5.3 equiv). The reaction was stirred for 19 h at 50 °C in a DrySyn heating block. The resulting yellow precipitate was filtered and washed with cold Et_2_O. The resulting solid was recrystallized from hot EtOH twice to yield compound **27** in 88% yield (111 mg, yellow solid).

^1^H NMR ((CD_3_)_2_SO, 400 MHz): δ 12.50 (s, 1H, N*H*), 10.28 (s, 1H, *H*^1^), 8.71 (dd, 1H, *J* = 6.7, 1.2 Hz, *H*^3^), 8.67 (d, 1H, *J* = 6.7 Hz, *H*^4^), 8.44 (d, 1H, *J* = 8.8 Hz, *H*^6^), 8.30 (d, 1H, *J* = 2.3 Hz, *H*^11^), 8.24 (d, 1H, *J* = 8.8 Hz, *H*^5^), 7.75 (d, 1H, *J* = 8.9 Hz, *H*^8^), 7.32 (dd, 1H, *J* = 8.9, 2.4 Hz, *H*^9^), 4.67 (s, 3H, −NC*H_3_*), 4.00 (s, 3H, −OC*H_3_*). ^13^C{^1^H} NMR ((CD_3_)_2_SO, 101 MHz): δ 154.7 (*C*^10^), 143.3 (*C*^1^), 139.4 (*C*^6a^), 134.4 (*C*^7a^), 133.5 (*C*^3^), 133.1 (*C*^4a^), 126.0 (*C*^4^), 124.3 (*C*^5^), 124.2 (*C*^6^), 123.4 (*C*^11c^), 121.3 (*C*^11a^), 116.1 (*C*^9^), 113.7 (*C*^11b^), 113.4 (*C*^8^), 105.5 (*C*^11^), 56.3 (−O*C*H_3_), 48.0 (−N*C*H_3_).

IR *v̅*_max_ (cm^–1^): 3129 (N–H stretch), 1631 (C=N stretch), 1562 (C=C stretch), 1497 (C=C stretch), 1029 (C–O stretch).

HRMS (ESI) *m*/*z*: [M]^+^ calcd for C_17_H_15_ON_2_, 263.1179; found, 263.1179.

### 10-Methoxy-2-(2-(piperidin-1-yl)ethyl)-7*H*-pyrido[4,3-*c*]carbazol-2-ium (**28**)^45^

To a solution of 10-methoxy-7*H*-pyrido[4,3-*c*]carbazole **9e** (60 mg, 0.24 mmol, 1.0 equiv) in DMF (0.5 mL) was added a solution of 1-(2-chloroethyl)piperidine hydrochloride (178 mg, 1.0 mmol, 4.0 equiv) in a 1:1 mixture of DMF:water (1 mL). The reaction was stirred for 19 h at 80 °C in a DrySyn heating block. The resulting yellow precipitate was filtered and washed with cold Et_2_O. The resulting solid was recrystallized from hot EtOH three times to yield compound **28** in 80% yield (77 mg, yellow solid).

^1^H NMR (400 MHz, DMSO-*d*_6_): δ 12.61 (s, 1H, N*H*), 11.00 (s, 1H, N*H*), 10.46 (s, 1H, *H*^1^) 8.86 (d, 1H, *J* = 6.7 Hz, *H*^3^), 8.76 (d, 1H, *J* = 6.7 Hz, *H*^4^), 8.50 (d, 1H, *J* = 8.9 Hz, *H*^6^), 8.41–8.34 (m, 1H, *H*^11^), 8.26 (d, 1H, *J* = 8.9 Hz, *H*^5^), 7.76 (d, 1H, *J* = 8.9 Hz, *H*^8^), 7.34 (dd, 1H, *J* = 8.9, 1.9 Hz, *H*^9^), 5.56–5.42 (m, 2H, 2 × *H*^1″^), 4.04 (s, 3H, −OC*H_3_*), 3.95–3.82 (m, 2H, 2 × *H*^2″^), 3.67–3.54 (m, 2H, 2 × *H*^2′^ or *H*^6′^), 3.11–2.94 (m, 2H, 2 × *H*^2′^ or *H*^6′^), 1.90–1.77 (m, 4H, 2 × *H*^3′,5′^), 1.76–1.70 (m, 1H, *H*^4′^), 1.49–1.40 (m, 1H, *H*^4′^). ^13^C{^1^H} NMR ((CD_3_)_2_SO, 101 MHz): δ 154.7 (*C*^10^), 143.8 (*C*^1^), 139.4 (*C*^6a^), 134.5 (*C*^7a^), 133.7 (*C*^4a^), 132.5 (*C*^3^), 126.4 (*C*^4^), 124.8 (*C*^6^), 124.3 (*C*^5^), 123.6 (*C*^11c^), 121.3 (*C*^11a^), 116.1 (*C*^9^), 114.1 (*C*^11b^), 113.5 (*C*^8^), 105.9 (*C*^11^), 56.4 (−O*C*H_3_), 55.0 (*C*^5^), 54.2 (*C*^2″^), 52.6 (2 × *C*^2′, 6′^), 22.2 (2 × *C*^3′, 5′^), 21.2 (*C*^4′^).

IR *v̅*_max_ (cm^–1^): 3390 (N–H stretch), 2958 (C–H stretch), 1634 (C=N stretch), 1219 (C–O stretch), 1029 (C–O stretch).

HRMS (ESI) *m*/*z*: [M]^+^ calcd for C_23_H_26_ON_3_, 360.2070; found, 360.2081.

### Ditercalinium (**29**)^47^

10-Methoxy-7*H*-pyrido[4,3-*c*]carbazole **9e** (100 mg, 0.40 mmol, 1.0 equiv) and 1,1′-bis(2-chloroethyl)-4,4′-bipiperidine (89 mg, 0.24 mmol, 0.6 equiv) were charged to a 2–5 mL microwave vial that was evacuated and purged with argon twice. DMF (4 mL) was added, and the vial was heated to 80 °C in a DrySyn heating block for 22 h. H_2_O (0.1 mL) was added to further solubilize the starting materials, and the reaction was heated for a further 24 h at 80 °C. DMF (1 mL) was added to redissolve the formation of a yellow precipitate, and the reaction was heated to 120 °C for a further 24 h. The reaction was cooled, and the precipitate was isolated by filtration. The precipitate was washed with cold Et_2_O and then recrystallized from hot EtOH five times to isolate **29** in 33% yield (52 mg, yellow solid).

^1^H NMR ((CD_3_)_2_SO, 400 MHz): δ 12.69 (s, 2H, N*H*), 11.63 (s, 2H, N*H*), 10.51 (s (br), 2H, *H*^1^), 9.00–8.78 (s (br), 2H, *H*^3^), 8.77–8.67 (d, 2H, *J* = 5.8 Hz, *H*^4^), 8.49 (d, 2H, *J* = 8.8 Hz, *H*^6^), 8.45–8.29 (s (br), 2H, *H*^11^), 8.25 (d, 2H, *J* = 8.9 Hz, *H*^5^), 7.75 (d, 2H, *J* = 8.9 Hz, *H*^8^), 7.31 (dd, 2H, *J* = 9.0, 2.2 Hz, *H*^9^), 5.71–5.26 (m, 4H, *H*^1″^), 4.03, 4.00–3.77 (m, 4H, *H*^2″^), 3.73–3.50 (m, 4H, 4 × *H*^2′eq^ or 4 × *H*^2′ax^), 3.11–2.91 (m, 4H, 4 × *H*^2′eq^ or 4 × *H*^2′ax^), 2.06–1.51 (m, 8H, 4 × *H*^3′eq^ and 4 × *H*^3′ax^), 1.48–1.29 (m, 2H, *H*^4′^). ^13^C{^1^H} NMR ((CD_3_)_2_SO, 101 MHz): δ 154.7 (*C*^10^), 143.7 (*C*^1^), 139.4 (*C*^6a^), 134.4 (*C*^7a^), 133.6 (*C*^4a^), 132.6 (*C*^3^), 126.4 (*C*^4^), 124.8 (*C*^6^), 124.3 (*C*^5^), 123.6 (*C*^11c^), 121.3 (*C*^11a^), 116.3 (*C*^9^), 114.1 (*C*^11b^), 113.4 (*C*^8^), 105.7 (*C*^11^), 56.4 (−O*C*H_3_), 55.3 (*C*^5^), 54.3 (*C*^2″^), 52.6 (2 × *C*^2′^), 37.3 (2×*C*^4′^), 25.8 (2 × *C*^3′^).

IR *v̅*_max_ (cm^–1^): 3342 (N–H stretch), 2927 (C–H stretch), 1616 (C=N stretch), 1472 (C=C stretch), 1402 (C=C stretch), 1214 (C–O stretch), 1031 (C–O stretch).

HRMS (ESI) *m*/*z*: [M]^2+^ calcd for C_46_H_50_O_2_N_6_, 359.1992; found, 359.1991.

## Supporting Information Available

Experimental procedures, characterization data, the ^1^H and ^13^C NMR spectra for new compounds, DFT calculations, and crystallographic data (PDF)

## Accession Codes

CCDC 2102051–2102053 contain the supplementary crystallographic data for this paper. These data can be obtained free of charge via www.ccdc.cam.ac.uk/data_request/cif, or by emailing data_request@ccdc.cam.ac.uk, or by contacting The Cambridge Crystallographic Data Centre, 12 Union Road, Cambridge CB2 1EZ, UK; fax: +44 1223 336033.

## Notes

All data underpinning this publication are openly available from the University of Strathclyde KnowledgeBase at https://doi.org/10.15129/f45b6f45-b8f7-435a-b5a3-097e3ff7397c.
