# Supplementary material for: A Chemo- and Regioselective Tandem [3 + 2]Heteroannulation Strategy for Carbazole Synthesis: Combining Two Mechanistically Distinct Bond-Forming Processes
Source: J Org Chem. 2022 Mar 18;87(7):4603–16. doi: 10.1021/acs.joc.1c02943 (PMC8981337; doi:10.1021/acs.joc.1c02943)
Supplement: Supplementary file 1 — jo1c02943_si_001.pdf [file jo1c02943_si_001.pdf]

A Chemo- and Regioselective Tandem [3+2]Heteroannulation Strategy for Carbazole Synthesis: Combining Two Mechanistically Distinct Bond-Forming Processes

Emma Campbell,[a] Andrea Taladriz-Sender,[a] Olivia I. Paisley,[a] Alan R, Kennedy,[a] Jacob T. Bush,[b] Glenn A. Burley[a*]

[a]Department of Pure and Applied Chemistry, University of Strathclyde. Thomas Graham Building, 295 Cathedral Street, Glasgow, United Kingdom. G1 1XL. [b]GlaxoSmithKline, Medicines Research Centre, Gunnels Wood Road, Stevenage, Hertfordshire, SG1 2NY, United Kingdom.

1 General Information S2

1.1 General S2

1.2 Purification of Solvents S2

1.3 Experimental Details S2

1.4 Purification of Products S2

1.5 Spectroscopic Analysis of Products S2

2 Optimisation Procedures S4

2.1 Optimisation Procedure for Microwave Assisted Buchwald-Hartwig Amination S4

2.2 Microwave Assisted Buchwald-Hartwig Amination Optimisation S4

2.3 Optimisation Procedure for Microwave Assisted One-Pot Buchwald-Hartwig Amination/Direct Arylation Optimisation S5

2.4 Microwave Assisted One-Pot Buchwald-Hartwig Amination/Direct Arylation Optimisation S6

3 Experimental Details S7

4 DFT Experimental S14

4.1 Compound 7a S14

4.2 Compound 16 ..... S15

5 Crystallographic Data S16

5.1 Experimental Technique, Single Crystal Diffraction S16

6 References S19

7 NMR, IR and MS Spectra S20

1 General Information

1.1 General

All reagents and solvents were obtained from commercial suppliers and were used without further purification unless otherwise stated. Purification was carried out according to standard laboratory methods. Starting materials were purchase from commercial suppliers and used without further purification unless otherwise stated.

1.2 Purification of Solvents

Dry solvents for reactions were purchased from Sigma-Aldrich and stored under nitrogen. Dichloromethane, chloroform, methanol, ethyl acetate, and petroleum ether (40 – 60 °C) for purification purposes were used as obtained from suppliers, without further purification.

1.3 Experimental Details

Reactions were carried out using conventional glassware for the preparation of starting materials. Microwave reactions were carried out in capped 2 - 5 mL microwave vials purchased from Biotage®. Microwave reactions were carried out at elevated temperatures using a Biotage® Initiator + equipped with a Robot Eight microwave system.

1.4 Purification of Products

Thin layer chromatography was carried out using Merck silica plates coated with fluorescent indicator UV254 and were analyzed under both 254 nm and 375 nm UV light or developed using potassium permanganate solution. Normal phase flash chromatography was carried out using 60Å 40-63 µm silica gel from Fluorochem. Semi-preparative reversed-phase HPLC purification was carried out on a Kinetex 5 µm, 150 × 21.2 mm XB C18 using a DIONEX 3000 series HPLC system equipped with a VWD3400 variable wavelength detector. Preparative purifications of small molecules were performed using a 30 – 90% gradient B (solvent A: 0.1% TFA in water, solvent B: 0.1% TFA in acetonitrile, with a flow rate of 12.0 mL/min. The absorbance UV-active material was detected at 254 nm. Analytical reversed-phase HPLC (RP-HPLC) was carried out on a Shimadzu Prominence instrument equipped with a PDA Detector scanning from 190 to 600 nm using a using a Thermofisher Hypersil GOLD™ column 100 x 4.6 mm, particle size 5 µm.

1.5 Spectroscopic Analysis of Products

Fourier Transformed Infra-Red (FTIR) spectra were obtained on a Shimadzu IR Affinity-1 instrument. Only major absorbance bands are reported. ^{1}H NMR, ^{13}C NMR and ^{19}F NMR spectra were obtained on

a Bruker AV 400 at 400 MHz, 101 MHz and 376 MHz, respectively. Chemical shifts are reported in ppm and coupling constants are reported in Hz with DMSO-d_6 referenced at 2.50 ppm (^{1}H) and 39.52 ppm (^{13}C) and MeOD-d_4 referenced at 3.31 ppm (^{1}H) and 49.0 ppm (^{13}C). Assignment of ^{13}C NMR signals is based on HSQC and HMBC experiments. COSY and NOESY spectra were used to assign unequivocally atom connectivities. High-resolution mass spectra were recorded on a Bruker microTOF II mass spectrometer at the SIRCAMs facility at the University of Edinburgh or on an LTQ Orbitrap xL at the EPSRC National Facility in Swansea.

2 Optimisation Procedures

2.1 Optimisation Procedure for Microwave Assisted Buchwald-Hartwig Amination

Aryl bromide (0.50 mmol, 1.00 equiv), chloroaniline (0.60 mmol, 1.20 equiv), $Pd(OAc)_2$ (5 mol%), ligand (10 mol%) and base (1.50 mmol, 3.0 equiv) were added to a microwave vial (2 – 5 mL). 1,4-Dioxane (5 mL, 0.1 M) was added, and the vial was capped, evacuated and purged with argon 3 times. The reaction was heated at 120 °C for 30 min under microwave irradiation in a Biotage® Microwave. The crude reaction mixture was analysed by Shimadzu analytical HPLC. The crude reaction was sampled by taking a 10 µL aliquot and diluted with 990 µL of DMSO. A 12 minutes method was used, 5 – 95% gradient B (solvent A: 0.1% TFA in water, solvent B: 0.1% TFA in acetonitrile), flow rate 2.0 mL/min. Conversions to products were calculated by integrated the HPLC peak area and plotting against a calibration curve of the appropriate product. All reactions were performed in duplicate.

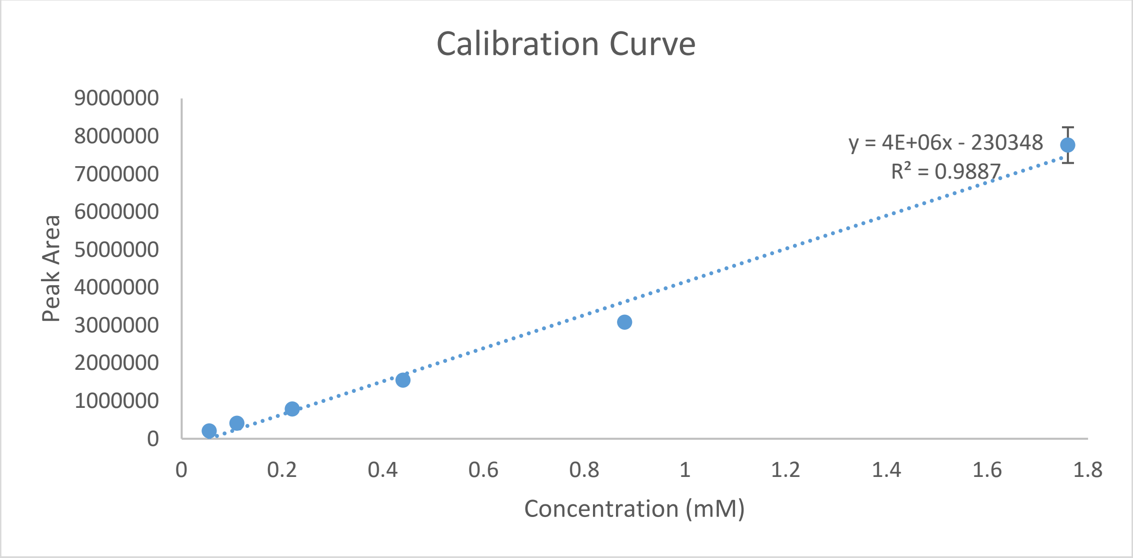

Figure S1. Calibration curve of compound **7a** for Microwave Assisted Buchwald-Hartwig Amination Optimisation. The calibration curve was run in triplicate and error bars correspond to standard deviation.

2.2 Microwave Assisted Buchwald-Hartwig Amination Optimisation

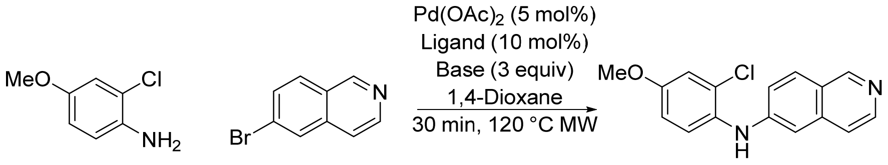

1a **6a** **7a**

Ligand	Base	Conversion (%)
DavePhos	NaO^{t}Bu	12
DavePhos	Cs_2CO_3	74
**DavePhos**	**K_3PO_4**	**85 (87)[a]**
$HPCy_3BF_4$	NaO^{t}Bu	75
$HPCy_3BF_4$	Cs_2CO_3	10
$HPCy_3BF_4$	K_3PO_4	10
$HP^tBu_3BF_4$	NaO^{t}Bu	7
$HP^tBu_3BF_4$	Cs_2CO_3	70
$HP^tBu_3BF_4$	K_3PO_4	65
XPhos	NaO^{t}Bu	47
XPhos	Cs_2CO_3	76
XPhos	K_3PO_4	77

Table S1. Optimisation of Buchwald-Hartwig Amination. All reactions carried out according to general procedure A. Conversions calculated by HPLC. [a] Isolated yield shown in brackets.

2.3 Optimisation Procedure for Microwave Assisted One-Pot Buchwald-Hartwig Amination/Direct Arylation Optimisation

Aryl bromide (0.50 mmol, 1.00 equiv), chloroaniline (0.60 mmol, 1.20 equiv), $Pd(OAc)_2$ (5 mol%), ligand (10 mol%) and base (1.50 mmol, 3.0 equiv) were added to a microwave vial (2 – 5 mL). 1,4-Dioxane (5 mL, 0.1 M) was added, and the vial was capped, evacuated and purged with argon 3 times. The reaction was heated at 120 °C for 30 mins, followed by 160 °C for 8 h under microwave irradiation in a Biotage® Microwave. The crude reaction mixture was analysed by Shimadzu analytical HPLC using a Hypersil Gold Luna Polar column. The crude reaction was sampled by taking a 10 µL aliquot and diluted with 490 µL of DMSO. A 12 minute method was used, 5 – 95% gradient B (solvent A: 0.1% TFA in water, solvent B: 0.1% TFA in acetonitrile) with a flow rate 2.0 mL/min. % Conversions to products were calculated by integrated the peak area by analytical HPLC and plotting against a calibration curve.

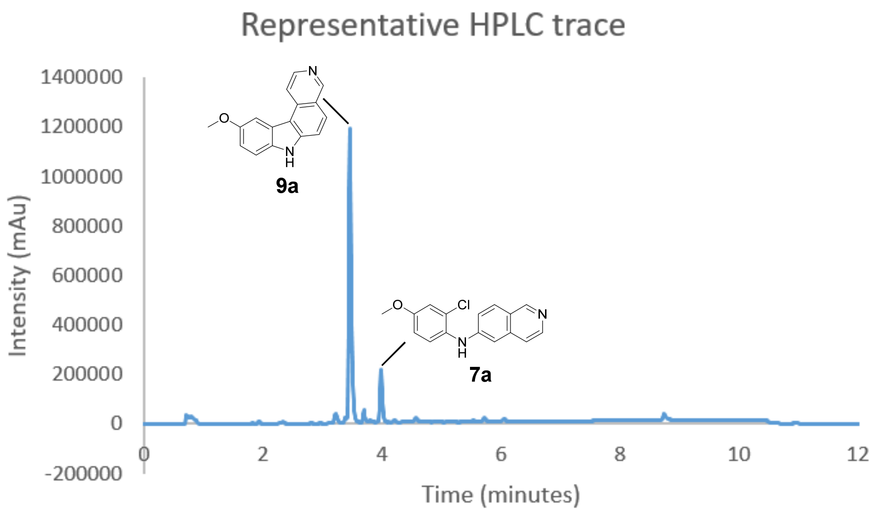

Figure S2. Representative HPLC trace of Microwave Assisted One-Pot Buchwald-Hartwig Amination/Direct Arylation Optimisation for compound **7a** and **9a**.

2.4 Microwave Assisted One-Pot Buchwald-Hartwig Amination/Direct Arylation Optimisation

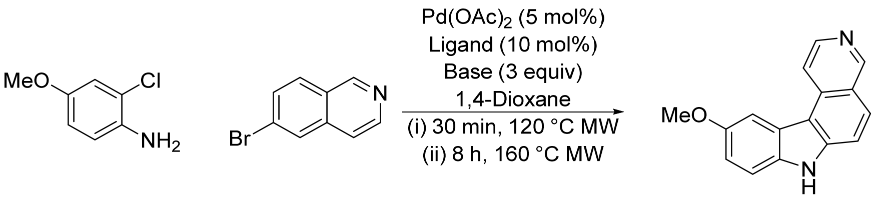

Ligand	Base	Conversion (%)
DavePhos	NaO^tBu	10
DavePhos	Cs_2CO_3	28
DavePhos	K_3PO_4	36
$HPCy_3BF_4$	NaO^tBu	45
$HPCy_3BF_4$	Cs_2CO_3	50
**$HPCy_3BF_4$**	**K_3PO_4**	**78 (82)** [a]
$HP^tBu_3BF_4$	NaO^tBu	3
$HP^tBu_3BF_4$	Cs_2CO_3	25
$HP^tBu_3BF_4$	K_3PO_4	31
XPhos	NaO^tBu	19
XPhos	Cs_2CO_3	18
XPhos	K_3PO_4	22

Table S2. Optimisation of Buchwald-Hartwig Amination/Direct Arylation. All reactions carried out according to general procedure B. Conversions calculated by HPLC. [a] Isolated yield shown in brackets.

3 Experimental Details

_N_-(2-chloro-4-methoxyphenyl)isoquinolin-6-amine (7a) – 1 mmol scale

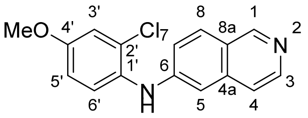

6-Bromoisoquinoline (208 mg, 1.00 mmol, 1.00 equiv), 2-chloro-4-methoxyaniline (188 mg, 1.20 mmol, 1.2 equiv), $Pd(OAc)_2$ (5 mol%), DavePhos (10 mol%) and K_3PO_4 (637 mg, 3.00 mmol, 3 equiv) were added to a microwave vial (10 – 20 mL). 1,4-Dioxane (10 mL, 0.1 M) was added and the vial was capped, evacuated and purged with argon 3 times, then heated at 120 °C for 30 minutes under microwave irradiation in a Biotage® Microwave. The reaction was allowed to cool to rt, diluted with ethyl acetate (50 mL) and the solid filtered under vacuum. The organic phase was washed with water, brine, dried with Na_2SO_4, filtered and the solvent was removed *in vacuo*. The crude residue was purified by silica-gel column chromatography, petroleum ether (40 – 60 °C): EtOAc (7: 3 to 0: 1) to obtain compound **7a** in 68% yield (195 mg, brown solid).

N-(2-chloro-4-methoxyphenyl)-N-(quinoxalin-2-yl)quinoxalin-2-amine (8)

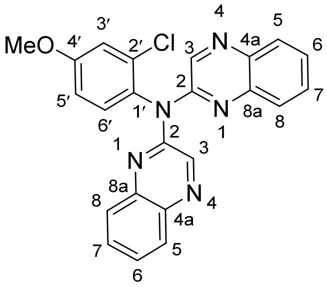

Preparation 1: The reaction was carried out according to general procedure using 2-bromoquinoxaline (105 mg, 0.50 mmol, 1.0 equiv) and 4-methoxy-2-chloroaniline (95 mg, 0.60 mmol, 1.2 equiv) as starting materials. The crude residue was purified by silica-gel column chromatography, petroleum ether (40 – 60 °C): EtOAc (85: 15) to obtained compound **8** in 25% yield (26 mg, yellow solid).

Preparation 2: The reaction was carried out according to general procedure B using 2-bromoquinoxaline (105 mg, 0.50 mmol, 1.0 equiv) and 4-methoxy-2-chloroaniline (95 mg, 0.60 mmol, 1.2 equiv) as starting materials. The crude residue was purified by silica-gel column chromatography, petroleum ether (40 – 60 °C): EtOAc (85: 15) to obtain compound **8** in 61% yield (63 mg, yellow solid). ^{1}H NMR ($(CD_3)_2SO$, 400 MHz): δ 8.83 (s, 2H, 2 × H^{3}), 8.06 – 8.00 (m, 2H, 2 × H^{5}), 7.76 – 7.68 (m, 6H, 2 × $H^{6/7/8}$), 7.58 (d, 1H, J = 8.8 Hz, $H^{6'}$), 7.32 (d, 1H, J = 2.9 Hz, $H^{3'}$), 7.12 (dd, 1H, J = 8.8, 2.9 Hz, $H^{5'}$), 3.88 (s, 3H, -OCH_3). ^{13}C{^{1}H} NMR ($(CD_3)_2SO$,101 MHz): δ 159.6 ($C^{4'}$), 150.4 (2 × C^{2}), 141.5 (2 × C^{3}), 140.0 (2 × C^{8a}), 138.8 (2 × C^{4a}), 133.1 ($C^{2'}$), 132.3 ($C^{6'}$), 130.9 ($C^{1'}$), 130.6 (2 × C^{6} or C^{7} or C^{8}), 128.5 (2 × C^{5}), 128.0 (2 × C^{6} or C^{7} or C^{8}), 127.4 (2 × C^{6} or C^{7} or C^{8}), 115.9 ($C^{3'}$), 115.0 ($C^{5'}$), 55.9 (-OCH_3).

IR $\bar{\nu}_{max}$ (cm^{-1}): 1606 (C=N stretch), 1558 (C=N stretch), 1498 (C=C stretch), 1420 (C=C stretch), 1229 (C-O stretch), 1022 (C-O stretch), 761 (C-Cl stretch).

HRMS (ESI) m/z: $[M+H]^+$ Calcd for $C_{23}H_{17}ON_5Cl$ 414.1116; Found 414.1115

10-methoxy-7*H*-pyrido[3,4-*c*]carbazole (9a) – 1 mmol scale

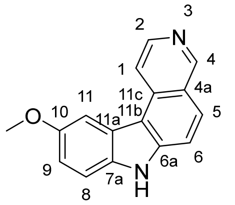

6-Bromoisoquinoline (208 mg, 1.00 mmol, 1 equiv), 2-chloro-4-methoxyaniline (189 mg, 1.20 mmol, 1.2 equiv), $Pd(OAc)_2$ (5 mol%), $P(Cy_3)$ .HBF_4 (10 mol%) and K_3PO_4 (636 mg, 3.00 mmol, 3 equiv) were added to a microwave vial (10 – 20 mL). 1,4-Dioxane (10.0 mL, 0.1 M) was added, and the vial was capped, evacuated and purged with argon 3 times and heated at 120 °C for 30 min followed by 160 °C for 8 h under microwave irradiation in a Biotage® Microwave. The reaction was allowed to cool to rt and diluted with DCM (50 mL). The solvent was removed *in vacuo* and the crude sample dry-loaded onto silica gel without work up. The crude residue was purified by silica gel column chromatography Hexane: EtOAc (3:7) to obtain compound **9a** in 56% yield (168 mg, brown solid).

2,7-dimethoxyphenazine (11)

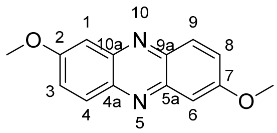

The reaction was carried out according to general procedure B using isoquinoline (65 mg, 0.50 mmol, 1.0 equiv) and 2-chloro-4-methoxyaniline (95 mg, 0.60 mmol, 1.2 equiv) as starting materials. The crude residue was purified by silica-gel column chromatography, hexane: EtOAc (3: 7) to obtain compound **11** in 35% yield (25 mg, brown solid). mp 146-150 °C.

^{1}H NMR ($(CD_3)_2SO$, 400 MHz): δ 8.06 (d, 2H, *J* = 9.4 Hz, $H^{4,9}$), 7.60 (dd, 2H, *J* = 9.4, 2.8 Hz, $H^{3,8}$), 7.50 (d, 2H, *J* = 2.8 Hz, $H^{1,6}$), 4.00 (s, 6H, 2 × -OCH_3). ^{13}C{^{1}H} NMR ($(CD_3)_2SO$,101 MHz): δ 159.8 ($C^{2,7}$), 142.9 ($C^{10a,5a}$), 139.8 ($C^{4a,9a}$), 129.8 ($C^{4,9}$), 125.9 ($C^{3,8}$), 105.1 ($C^{1,6}$), 55.9 (2 × -OCH_3).

IR $\bar{\upsilon}_{max}$ (cm^{-1}): 2975 (C-H stretch), 1633 (C=N stretch), 1491 (C=C stretch), 1437 (C=C stretch), 1217 (C-O stretch).

HRMS (ESI) m/z: $[M+H]^+$ Calcd for $C_{14}H_{13}O_2N_2$ 241.0972; Found 241.0976.

N-(2-chloro-4-methoxyphenyl)-N-(isoquinolin-6-yl)isoquinolin-6-amine (12)

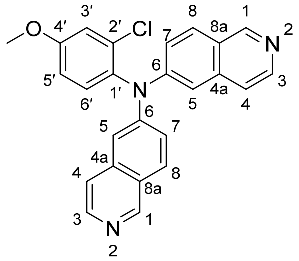

The reaction was carried out according to general procedure B using isoquinoline (65 mg, 0.50 mmol, 1.0 equiv) and 2-chloro-4-methoxyaniline (95 mg, 0.60 mmol, 1.2 equiv) as starting materials. The crude residue was purified by silica-gel column chromatography, hexane: EtOAc (2: 8) to obtain compound **12** in 25% yield (60 mg, brown solid).

IR $\bar{\boldsymbol{\upsilon}}_{\boldsymbol{max}}$ (cm^{-1}): 2975 (C-H stretch), 1633 (C=N stretch), 1491 (C=C stretch), 1437 (C=C stretch), 1217 (C-O stretch), 1016 (C-O stretch), 810 (C-Cl stretch).

^{1}H NMR ($(CD_3)_2SO$, 400 MHz): δ 9.16 (s, 2H, 2 × H^1), 8.36 (d, 2H, *J* = 5.8 Hz, 2 × H^3), 8.04 (d, 2H, *J* = 8.9 Hz, 2 × H^8), 7.64 (d, 2H, *J* = 5.8 Hz, 2 × H^4), 7.43 (d, 1H, *J* = 8.8 Hz, $H^{6'}$), 7.37 (dd, 2H, *J* = 8.9, 2.2 Hz, 2 ×

H^{7}), 7.32 (d, 2H, J = 2.2 Hz, 2 × H^{5}), 7.28 (d, 1H, J = 2.9 Hz, $H^{3'}$), 7.09 (dd, 1H, J = 8.8, 2.9 Hz, $H^{5'}$), 3.86 (s, 3H, -OCH_3). $^{13}C\{^{1}H\}$ NMR ($(CD_3)_2SO$, 101 MHz): δ 159.0 ($C^{4'}$), 151.5 (2 × C^{1}), 147.3 (2 × C^{6}), 143.4 (2 × C^{3}), 136.6 (2 × C^{4a}), 134.6 ($C^{1'}$), 133.3 ($C^{2'}$), 132.4 ($C^{6'}$), 129.2 (2 × C^{8}), 124.8 (2 × C^{8a}), 123.1 (2 × C^{7}), 119.6 (2 × C^{4}), 116.1 ($C^{3'}$), 115.4 ($C^{5'}$), 114.0 (2 × C^{5}), 55.9 (-OCH_3).

HRMS (ESI) m/z: $[M+H]^+$ Calcd for $C_{25}H_{19}ON_3Cl$ 412.1211; Found 412.1222

N-(4-methoxyphenyl)isoquinolin-6-amine (15)

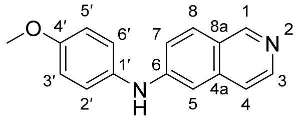

The reaction was carried out according to general procedure B using 6-bromoisoquinoline (104 mg, 0.50 mmol, 1.0 equiv) and 4-methoxyaniline (74 mg, 0.60 mmol, 1.2 equiv) as starting materials. The crude residue was purified by silica-gel column chromatography, hexane: EtOAc (3: 7) to obtain compound **15** in 95% yield (119 mg, brown solid).

^{1}H NMR ($(CD_3)_2SO$, 400 MHz): δ 8.93 (s, 1H, H^{1}), 8.51 (s, 1H, -NH), 8.21 (d, 1H, J = 5.8 Hz, H^{3}), 7.86 (d, 1H, J = 8.9 Hz, H^{8}), 7.43 (d, 1H, J = 5.8 Hz, H^{4}), 7.24 (dd, 1H, J = 8.9, 2.3 Hz, H^{7}), 7.23 – 7.18 (m, 2H, $H^{2',6'}$), 7.08 (d, 1H, J = 2.1 Hz, H^{5}), 6.99 – 6.94 (m, 2H, $H^{3',5'}$), 3.76 (s, 3H, -OCH_3).

$^{13}C\{^{1}H\}$ NMR ($(CD_3)_2SO$, 101 MHz): δ 155.2 ($C^{4'}$), 150.9 (C^{1}), 147.3 (C^{6}), 143.1 (C^{3}), 137.5 (C^{4a}), 134.1 ($C^{1'}$), 128.9 (C^{8}), 122.9 (C^{8a}), 122.7 ($C^{2',6'}$), 119.7 (C^{7}), 118.7 (C^{4}), 114.7 ($C^{3',5'}$), 102.4 (C^{5}), 55.2 (-OCH_3).

IR $\bar{\upsilon}_{max}$ (cm^{-1}): 3263 (N-H stretch), 3040 (C-H stretch), 2833 (C-H stretch), 1618 (C=N stretch), 1510 (C=C stretch), 1498 (C=C stretch), 1238 (C-O stretch), 1035 (C-O stretch).

HRMS (ESI) m/z: $[M+H]^+$ Calcd for $C_{16}H_{15}ON_2$ 251.1179; Found 251.1178

N-(2-chloro-4-methoxyphenyl)-N-(isoquinolin-6-yl)acetamide (16)

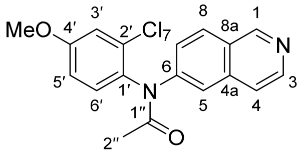

N-(2-chloro-4-methoxyphenyl)isoquinolin-6-amine (100 mg, 0.35 mmol, 1.0 equiv) was dissolved in acetic anhydride (0.5 mL) and DMAP (4 mg, 0.04 mmol, 10 mol%) was dissolved in acetic anhydride (0.5 mL) before being added to the reaction mixture. The reaction was stirred at rt for 19 h before being quenched with H_2O and extracted with EtOAc three times. The combined organic layer was washed with a saturated solution of $NaHCO_3$ four times, washed with brine and then dried over Na_2SO_4. The crude was concentrated *in vacuo* and dry loaded onto silica-gel for purification. The crude residue was purified by silica-gel column chromatography, hexane: EtOAc (3: 7) to obtain compound **16** in 28% yield (32 mg, brown oil).

^{1}H NMR ($(CD_3)_2SO$, 400 MHz): δ 9.26 (s, 1H, H^{1}), 8.46 (d, 1H, J = 5.4 Hz, H^{3}), 8.10 (d, 1H, J = 8.8 Hz, H^{8}), 7.82 – 7.73 overlapping with 7.77 (m (br), 1H, H^{5}), 7.77 (d, 1H, J = 5.4 Hz, H^{4}), 7.69 – 7.58 overlapping with 7.66 (m (br), 1H, $H^{6'}$), 7.66 (dd, 1H, J = 8.8, 2.1 Hz, H^{7}), 7.25 (d, 1H, J = 1.5 Hz, $H^{3'}$), 7.06 (dd, 1H, J

= 8.5, 1.5 Hz, $H^{5'}$), 3.82 (s, 3H, -OCH_3), 1.98 (s, 3H, -CH_3). ^{13}C{^{1}H} NMR ((CD_3)$_2$SO,101 MHz): δ 169.8 ($C^{1''}$), 159.7 ($C^{4'}$), 151.8 (C^1), 143.4 (C^3), 143.3 (C^6), 135.4 (C^{4a}), 133.1 ($C^{1'}$), 132.1 ($C^{6'}$), 128.2 (C^8), 126.2 (C^7), 126.1 ($C^{2'}$), 121.4 (C^{8a}), 120.2 ($C^{4,5}$), 115.4 ($C^{3'}$), 114.7 ($C^{5'}$), 55.9 (-OCH_3), 23.2 (-CH_3).

IR $\bar{\upsilon}_{max}$ (cm^{-1}): 2932 (C-H stretch), 2851 (C-H stretch), 1687 (C=O stretch), 1631 (C=N stretch), 1603 (C=C stretch), 1497 (C=C stretch), 1214 (C-O stretch), 1039 (C-O stretch).

HRMS (ESI) m/z: [M+H]$^+$ Calcd for $C_{18}H_{16}O_2N_2Cl$ 327.0895; Found 327.0895

N-(2-chloro-4-methoxyphenyl)-N-(quinolin-2-yl)quinolin-2-amine (22)

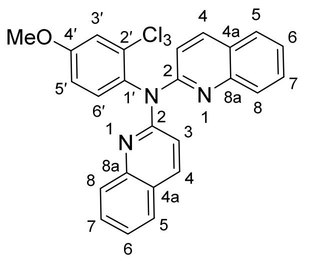

The reaction was carried out according to general procedure B using 2-bromoquinoline (104 mg, 0.50 mmol, 1.0 equiv) and 2-chloro-4-methoxyaniline (95 mg, 0.60 mmol, 1.2 equiv) as starting materials. The crude residue was purified by silica-gel column chromatography, hexane: EtOAc (3: 7) to obtain compound **22** in 52% yield (54 mg, brown solid).

^{1}H NMR ((CD_3)$_2$SO, 400 MHz): δ 8.22 (d, 2H, J = 9.0 Hz, 2 × H^4), 7.88 (d, 2H, J = 8.0 Hz, 2 × H^5), 7.66 – 7.59 (m, 4H, 2 × $H^{7,8}$), 7.45 (ddd, 2H, J = 8.1, 5.5, 2.8 Hz, 2 × H^6), 7.38 (d, 1H, J = 8.8 Hz, $H^{6'}$), 7.28 (d, 2H, J = 8.9 Hz, 2 × H^3), 7.24 (d, 1H, J = 2.9 Hz, $H^{3'}$), 7.07 (dd, 1H, J = 8.8, 2.9 Hz, $H^{5'}$), 3.87 (s, 3H, -OCH_3). ^{13}C{^{1}H} NMR ((CD_3)$_2$SO,101 MHz): δ 158.9 ($C^{4'}$), 155.3 (2 × C^2), 146.4 (2 × C^{8a}), 137.3 (2 × C^4), 133.5 ($C^{1',2'}$), 132.6 ($C^{6'}$), 129.7 (2 × C^7), 127.6 (2 × C^5), 127.2 (2 × C^8), 125.2 (2 × C^{4a}), 124.7 (2 × C^6), 116.6 (2 × C^3), 115.5 ($C^{3'}$), 114.6 ($C^{5'}$), 55.8 (-OCH_3).

IR $\bar{\upsilon}_{max}$ (cm^{-1}): 1597 (C=N stretch), 1575 (C=N stretch), 1502 (C=C stretch), 1428 (C=C stretch), 1230 (C-O stretch), 1040 (C-O stretch).

HRMS (ESI) m/z: [M+H]$^+$ Calcd for $C_{25}H_{19}ON_3Cl$ 412.1211; Found 412.1230

2-(2-chloro-4-hydroxyphenyl)isoindoline-1,3-dione

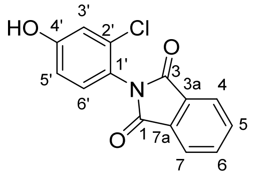

4-Amino-3-chlorophenol (10.0 g, 69.7 mmol) and 1, 2-benzenedicarboxylic anhydride (13.4 g, 90.6 mmol) were heated in acetic acid (400 mL) to 120 °C in a DrySyn® heating block for 19 h. The reaction mixture was quenched with water and the precipitate isolated by filtration to obtain 2-(2-chloro-4-hydroxyphenyl)isoindoline-1,3-dione in 93% yield (17.8 g, purple solid).

^{1}H NMR ((CD_3)$_2$SO, 400 MHz): δ 10.33 (s, 1H, -OH), 7.99 – 7.90 (m, 4H, 4 × H^{4-7}) 7.39 (d, 1H, J = 8.7 Hz, $H^{6'}$) 7.03 (d, 1H, J = 2.6 Hz, $H^{3'}$), 6.88 (dd, 1H, J = 8.7, 2.7 Hz, $H^{5'}$). ^{13}C{^{1}H} NMR ((CD_3)$_2$SO,101 MHz): δ

166.8 (2 × $C^{1,3}$), 159.1 ($C^{4'}$), 135.0 (2 × $C^{5,6}$), 132.6 ($C^{1'}$), 132.0 ($C^{6'}$), 131.4 (2 × $C^{3a,7a}$), 123.6 (2 × $C^{4,7}$), 120.3 ($C^{2'}$), 116.1 ($C^{3'}$), 115.0 ($C^{5'}$).

IR $\bar{v}_{max}$ (cm^{-1}): 3505 (O-H stretch), 2889 (C-H stretch), 2777 (C-H stretch), 1721 (C=O stretch), 1694 (C=O stretch), 1384 (C-H bend), 1096 (C-O stretch).

HRMS (ESI) m/z: $[M+H]^+$ Calcd for $C_{14}H_8ClNO_3$ 274.0265; found 274.0269

2-Chloro-4-[2-(dimethylamino)ethoxy]aniline

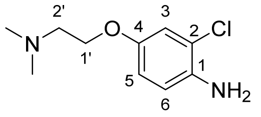

2-Chloro-N,N-dimethylethylamine hydrochloride (10.0 g, 69.43 mmol) was stirred vigorously in a mixture of water (43 mL), toluene (15 mL) and NaOH aq (30% w/v, 8 mL) for 1 h. The organic layer was separated for use in the next step. To a solution of 2-(2-chloro-4-hydroxyphenyl)isoindoline-1,3-dione (2.00g, 7.31 mmol), K_2CO_3 (3.03 g, 21.9 mmol) and TBAB (25 mg, 0.08 mmol) in EtOAc (30 mL), 2-Chloro-N,N-dimethylethylamine in toluene (8 mL) was added and the reaction heated to 80 °C in a DrySyn® heating block for 4 h. The reaction mixture was concentrated *in vacuo* and filtered through a silica plug to obtain the alkylated intermediate without further purification. A portion of the crude residue (1.2 g, 3.48 mmol) obtained was re-dissolved in a solution of EtOH (20 mL) and DCM (4 mL) before hydrazine monohydrate (0.33 mL) was added. The reaction was stirred at rt for 2 h and the reaction was filtered. The filtrate was concentrated *in vacuo* to obtain 2-chloro-4-[2-(dimethylamino)ethoxy]aniline in 75% overall yield (0.71 g, brown oil).

^{1}H NMR (400 MHz, DMSO-d_6) δ 6.83 (d, 1H, J = 2.8 Hz, H^3), 6.74 (d, 1H, J = 8.5 Hz, H^6), 6.69 (dd, 1H, J = 8.5, 2.6 Hz, H^5), 4.83 (s, 2H, -NH_2), 3.91 (t, 2H, J = 5.9 Hz, $H^{1'}$), 2.55 (t, 2H, J = 5.9 Hz, $H^{2'}$), 2.33 (s, 6H, 2 × -CH_3). ^{13}C{^{1}H} NMR ($(CD_3)_2SO$,101 MHz): δ 153.0 (C^4), 139.1 (C^1), 120.8 (C^2), 118.3 (C^6), 116.5 (C^5), 116.0 (C^3), 67.5 ($C^{1'}$), 59.2 ($C^{2''}$), 44.4 (2 × -CH_3).

IR $\bar{v}_{max}$ (cm^{-1}): 3334 (N-H stretch), 3198 (N-H stretch), 2947 (C-H stretch), 2778 (C-H stretch), 1605 (N-H stretch), 1504 (N-H stretch), 1212 (C-O stretch), 1039 (C-O stretch).

HRMS (ESI) m/z: $[M+H]^+$ Calcd for $C_{10}H_{15}ClN_2O$ 215.0946; found 215.0947

11-methyl-7*H*-pyrido[3,4-*c*]carbazole and 10-methylellipticine

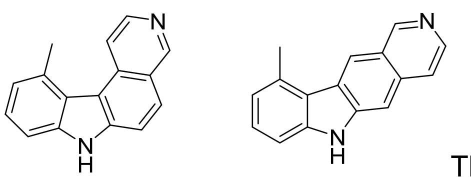

The reaction was carried out according to general procedure B using 6-bromoisoquinoline (104 mg, 0.50 mmol, 1.0 equiv) and 2-chloro-3-methylaniline (85 mg, 0.60 mmol, 1.2 equiv) as starting materials. The crude residue was

purified by silica-gel column chromatography, DCM: MeOH (0% to 2% MeOH) to obtain compound a mix of regioisomers in a 1:2 ratio in 16% yield (19 mg, grey solid).

^{1}H NMR (($(CD_3)_2SO$, 400 MHz): δ 12.19 (s, 1H, -N*H*), 11.96 (s, 1H, -N*H*), 9.31 (s, 1H), 9.28 (s, 1H), 8.83 (d, 1H, *J* = 6.0), 8.63-8.54 (m,1H), 8.54-8.43 (m, 1H, 1H), 8.06 (d,1H, J= 8.7 Hz), 8.03 (d, 1H, *J* = 8.8 Hz), 7.87 (dd, 1H *J* = 8.8, 5.8 Hz), 7.53-7.43 (m, 2H, 1H),7.35 (dd, 2H *J*= 7.7 Hz), 7.19 (d, *J* = 8.1 Hz, 1H), 7.12 (d, *J* = 7.2 Hz, 2H), 3.20 (s, 3H), 2.53 (s, 3H).

Synthesis of alkylated 7H-pyrido[4,3-c]carbazole Ditercalinium.

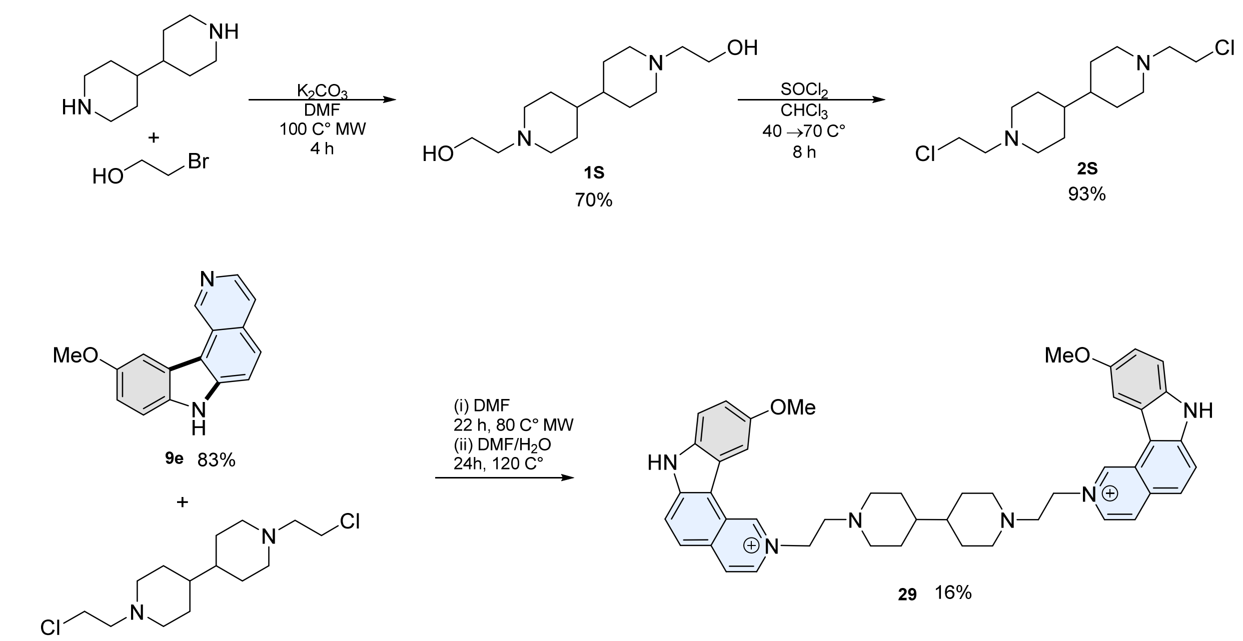

Scheme S1. Synthesis of Ditercalinium **29**.

2,2'-([4,4'-bipiperidine]-1,1'-diyl)bis(ethan-1-ol) (1S)[7]

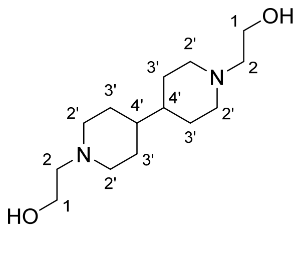

4,4′-Bipiperidine (300 mg, 1.24 mmol, 1.0 equiv) and K_2CO_3 (1.03 g, 7.47 mmol, 6.0 equiv) was charged to a 10 – 20 mL microwave vial before being sealed, evacuated and purged with argon three times. DMF (10 mL) was added followed by bromoethanol (528 mg, 4.23 mmol, 3.4 equiv) before being heated for 4 h at 100 °C using microwave irradiation. The crude residue was purified by silica column chromatography, MeOH: NH_4OH (100: 5) to obtain 2,2'-([4,4'-bipiperidine]-1,1'-diyl)bis(ethan-1-ol) in 70% yield (223 mg, white solid).

^{1}H NMR (CD_3OD, 400 MHz): δ 3.80 (t, 4H, *J* = 5.6 Hz, H^1), 3.40 – 3.33 (m, 4H, 4 × $H^{2'eq}$ or 4 × $H^{2'ax}$), 2.90 (t, 4H, *J* = 5.6 Hz, H^2), 2.56 (t,4H, *J* = 11.8 Hz, 4 × $H^{2'eq}$ or 4 × $H^{2'ax}$), 1.90 (d, 4H, *J* = 13.4 Hz, 4 × $H^{3'eq}$ or 4

× $H^{3'ax}$), 1.54 – 1.41 (m, 4H, 4 × $H^{3'eq}$ or 4 × $H^{3'ax}$), 1.39 (m, 2H, $H^{4'}$).$^{13}C\{^1H\}$ NMR (CD_3OD,101 MHz): δ 60.5 (C^2), 58.1 (C^1), 54.7 (2 × $C^{2'}$), 40.2 ($C^{4'}$), 28.6 (2 × $C^{3'}$).

IR $\bar{\nu}_{max}$ (cm^{-1}): 3345 (O-H stretch), 2936 (C-H stretch), 1266 (C-N stretch), 1052 (C-O stretch).

LC-MS (ESI +ve mode): m/z = 257.3

1,1'-bis(2-chloroethyl)-4,4'-bipiperidine (2S)[7]

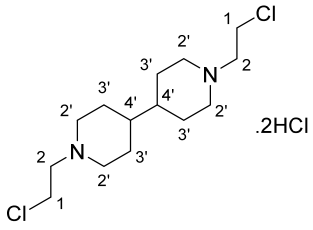

2,2'-([4,4'-bipiperidine]-1,1'-diyl)bis(ethan-1-ol) (163 mg, 0.64 mmol, 1.0 equiv) was dissolved in $CHCl_3$ (20 mL) under argon atmosphere. The reaction was heated to 40 °C and stirred for 30 minutes to fully dissolved the starting material. $SOCl_2$ (303 mg, 2.54 mmol, 4.0 equiv) was added dropwise and the reaction heated to 70 °C in a DrySyn® heating block for 8 h. The reaction was cooled to rt and the precipitate isolated through filtration to obtain crude 1,1'-bis(2-chloroethyl)-4,4'-bipiperidine in 93% yield (216 mg, white solid) to be used directly in the next step without purification.

4 DFT Experimental

All the calculations are carried out with Gaussian 09 program and visualized in Gaussview v05. All calculations were carried out with the DFT B3LYP method with the 6-31G+G(d,p) basis set and optimized to a minimum.

4.1 Compound 7a

E = -1262.49949229 a.u.

HOMO = -0.20496 eV **LUMO =** -0.05912 eV

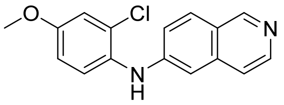

HOMO calculations for **7a**

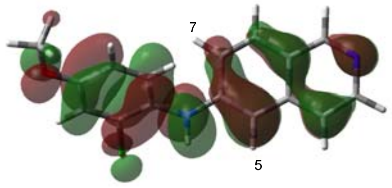

7a

Row	Symbol	X	Y	Z
1	C	1.670868	-0.93267	0.486367
2	C	1.416851	0.408982	0.166901
3	C	2.53866	1.206679	-0.14782
4	C	3.829314	0.697554	-0.15984
5	C	4.048663	-0.65599	0.128385
6	C	2.959309	-1.47128	0.454537
7	H	0.843682	-1.56767	0.782962
8	H	4.67236	1.33442	-0.40047
9	H	3.09454	-2.51611	0.705869
10	N	0.136562	0.972618	0.204509
11	H	0.097455	1.952115	0.44821
12	C	-1.09291	0.342981	0.013535
13	C	-2.23548	0.891807	0.58484
14	C	-1.21284	-0.81598	-0.81541
15	C	-3.50986	0.319757	0.359823
16	H	-2.1528	1.770184	1.220596
17	C	-2.44105	-1.39222	-1.03314
18	H	-0.33014	-1.22011	-1.29722
19	C	-4.71634	0.828073	0.914101
20	C	-3.61906	-0.85058	-0.45315
21	H	-2.52128	-2.2665	-1.67426
22	C	-5.90672	0.187833	0.649345
23	H	-4.69806	1.71403	1.542787
24	C	-4.90858	-1.41073	-0.64906
25	H	-6.83818	0.562711	1.066209
26	H	-5.00794	-2.30142	-1.27037
27	N	-6.02066	-0.92661	-0.12781
28	Cl	2.309373	2.916655	-0.50783
29	O	5.350167	-1.06979	0.069698
30	C	5.639431	-2.4328	0.35648
31	H	5.132655	-3.10519	-0.34726
32	H	6.71892	-2.53714	0.242766

33	H	5.355122	-2.69474	1.383425

Table S3. DFT atom list compound **9a**.

4.2 Compound 16

E= -1415.15310016 a.u.

HOMO = -0.23190 eV **LUMO =** -0.07160 eV

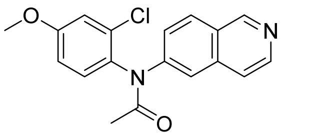

	Atom	X	Y	Z
1	C	2.087934	-0.02024	1.335721
2	C	1.381352	0.504475	0.252491
3	C	1.835685	0.189781	-1.03967
4	C	2.932943	-0.6387	-1.23894
5	C	3.622557	-1.1594	-0.13503
6	C	3.200476	-0.84403	1.163213
7	H	1.764458	0.239047	2.338704
8	H	3.274739	-0.87691	-2.23908
9	H	3.721309	-1.2224	2.03382
10	N	0.237845	1.345086	0.472083
11	C	-1.06133	0.796111	0.212024
12	C	-1.97166	1.493718	-0.63163
13	C	-1.4128	-0.4319	0.740856
14	C	-3.21741	0.973492	-0.89475
15	H	-1.66028	2.427875	-1.08475
16	C	-2.68546	-0.99733	0.469597
17	H	-0.71151	-0.97399	1.36725
18	C	-3.60638	-0.27903	-0.35196
19	H	-3.90663	1.508523	-1.54266
20	C	0.465027	2.624827	0.991
21	O	4.688404	-1.95407	-0.43322
22	C	5.462273	-2.4879	0.636923
23	H	4.860341	-3.14762	1.273818
24	H	6.257337	-3.06643	0.165838
25	H	5.903171	-1.68917	1.245462
26	N	-5.2628	-2.03273	-0.11125
27	C	-3.11625	-2.24944	0.983824
28	H	-2.45556	-2.83745	1.614615
29	C	-4.37717	-2.70888	0.674703
30	H	-4.72747	-3.66374	1.057803
31	C	-4.87726	-0.86925	-0.59684
32	H	-5.5935	-0.33939	-1.22516
33	O	1.603665	3.055943	1.110761
34	Cl	1.033825	0.856597	-2.44926
35	C	-0.73275	3.445815	1.436573
36	H	-1.07757	4.084886	0.617479
37	H	-1.57606	2.842863	1.778266
38	H	-0.38843	4.097469	2.241273

Table S4. DFT atom list compound **16**.

HOMO calculations for **16**

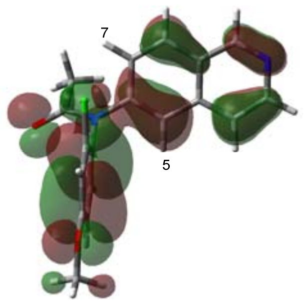

16

5 Crystallographic Data

5.1 Experimental Technique, Single Crystal Diffraction

Data for **7k**, **7n** and **11** were measured with an Oxford Diffraction Synergy-i instrument using Cu Kα(λ = 1.54184 Å) radiation. In all cases, data collection and processing used CrysalisPro software.[8] All structures were refined to convergence on F^2 using all independent reflections and SHELXL-2018 as implemented within WinGX.[9,10] In all structures the non-hydrogen atoms were refined anisotropically and any hydrogen atoms bound to nitrogen were refined freely and isotropically. All other hydrogen atoms were placed in idealised positions and refined in riding modes. Samples of **7n** were found to be twinned by a 180° rotation about the 100 direction. This structure was thus refined against a hklf 5 formatted dataset and the twin ratio refined to 0.781(3):0.219(3). Selected crystallographic data and refinement parameters are presented in Table **S5**. Structural figures showing displacement ellipsoids are given in Figures **S3**, **S4** and **S5**. CCDC deposition numbers 2102051 to 2102053 contain the full supplementary crystallographic data for this paper in cif format. These data are provided free of charge by the joint Cambridge Crystallographic Data Centre and Fachinformationszentrum Karlsruhe Access Structures service www.ccdc.cam.ac.uk/structures.

**Compound**	**7k**	**7n**	**11**
**CCDC**	2102051	2102052	2102053
**Formula**	$C_{17}H_{12}ClNO_3$	$C_{17}H_9ClF_3NO_2$	$C_{14}H_{12}N_2O_2$
**Form. Wt.**	313.73	351.70	240.26
**Space Group**	P1	P-1	$P2_1/n$
**Crystal system**	Triclinic	Triclinic	Monoclinic
**Temp. (K)**	100(2)	100(2)	100(2)
**a (Å)**	3.8010(3)	7.6416(1)	3.8270(1)
**b (Å)**	7.9128(6)	15.3687(1)	18.3658(7)
**c (Å)**	11.9303(6)	24.7412(2)	7.9201(3)
**α (°)**	73.367(6)	97.874(1)	90
**β (°)**	81.808(6)	94.672(1)	98.847(3)
**γ (°)**	83.153(6)	97.008(1)	90
**Volume (Å^3)**	339.13(4)	2842.47(5)	550.05(3)
**Z**	1	8	2
**Z'**	1	4	0.5
**Measured Reflections**	5624	43157	5007
**Unique Reflections**	2301	43157	1067
**2θmax (°)**	143.10	143.48	142.68
**R_{int}**	0.0607	0.0435*	0.0510
**Observed Reflections [I>2σI]**	2251	35901	906
**No. Parameters**	205	882	83
**S**	1.082	1.047	1.049
**R [on *F*, obs refs only]**	0.0689	0.0538	0.0446

**ωR [on F^2, all data]**	0.1760	0.1590	0.1307
**Largest diff. peak /hole (eÅ^{-3})**	0.860/-0.554	0.822/-0.419	0.194/-0.296
**Flack parameter**	0.02(3)	n.a.	n.a.

**Value prior to twin treatment.*

Table S5. Selected crystallographic data and refinement parameters.

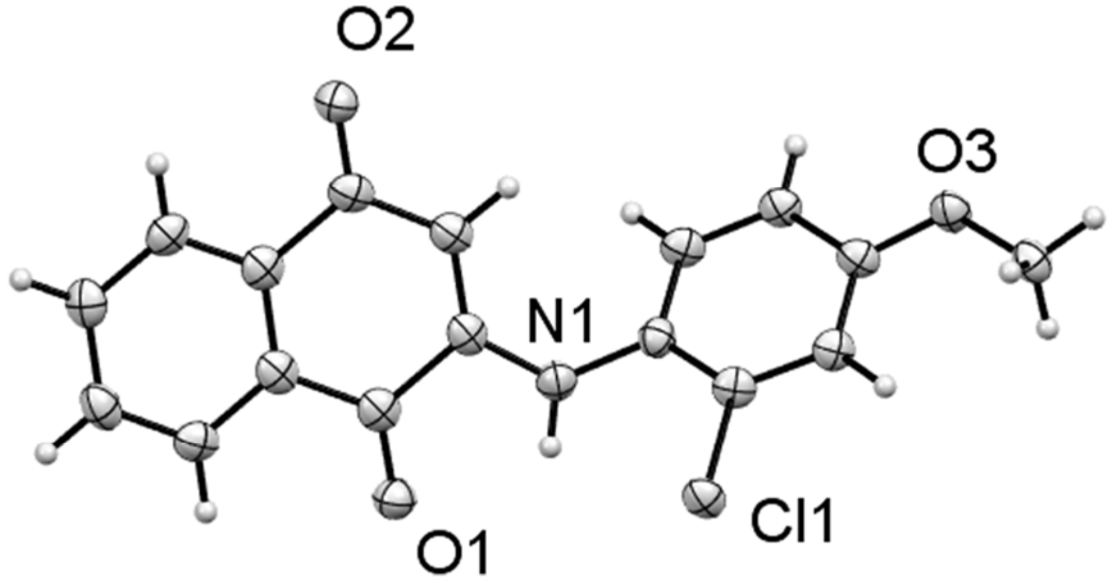

Figure S3. ORTEP representation of the structure of 7k.[3] Here and in other figures, the non-H atoms are shown as 50 % probability ellipsoids and the H atoms are shown as small spheres of arbitrary size.

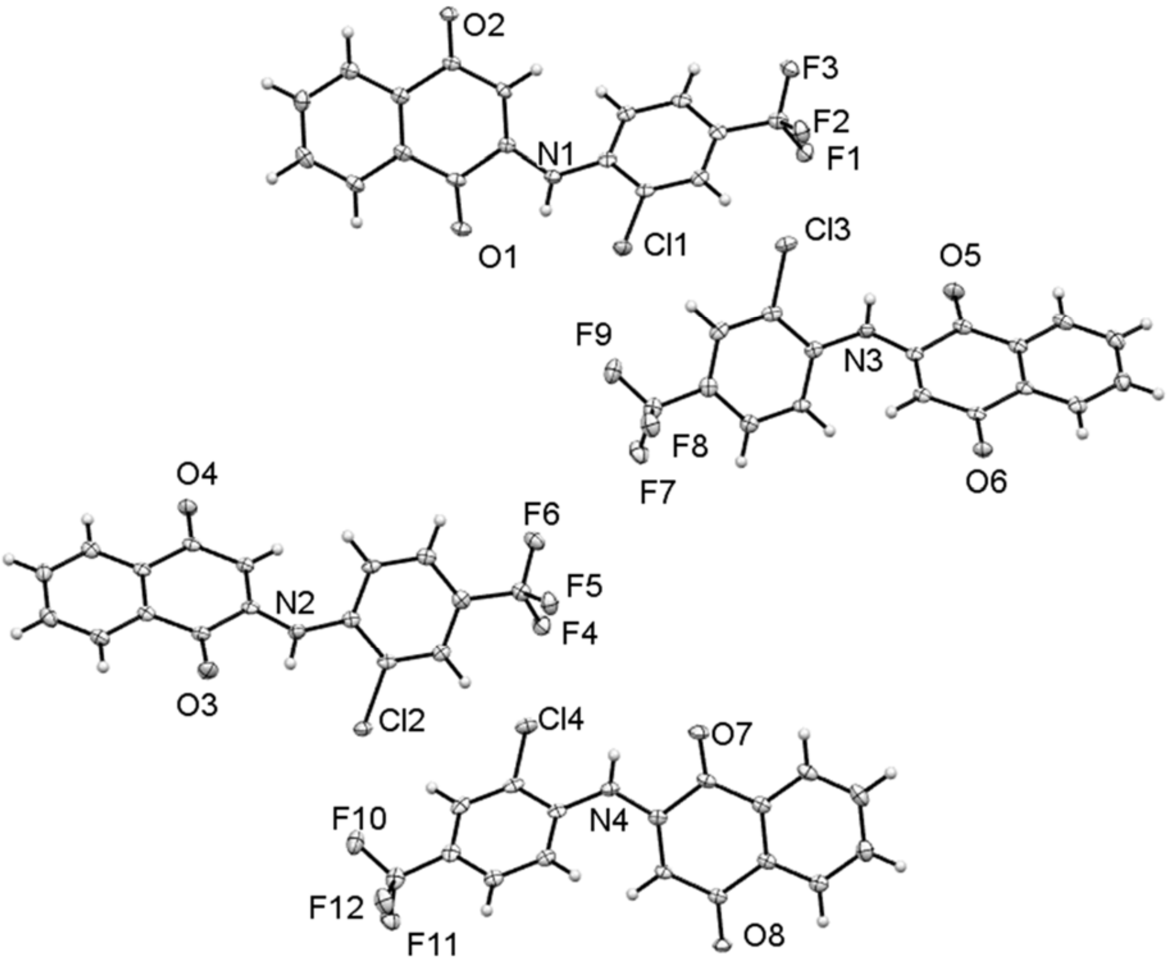

Figure S4. ORTEP representation of the asymmetric unit contents of 7n.[3] Note that this structure has Z' = 4. The non-H atoms are shown as 50 % probability ellipsoids and the H atoms are shown as small spheres of arbitrary size.

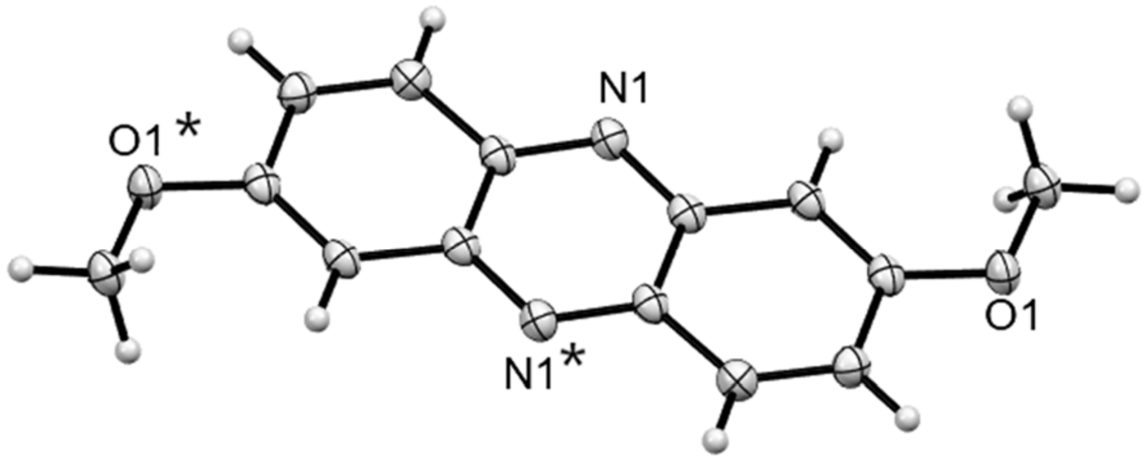

Figure S5. ORTEP representation of the structure of 11.[3] The molecule has a crystallographic centre of symmetry, where * represents the operation -x, 2-y, 2-z. The non-H atoms are shown as 50 % probability ellipsoids and the H atoms are shown as small spheres of arbitrary size.

6 References

1. Q. Yan, E. Gin, M. Wasinska-Kalwa, M. G. Banwell and P. D. Carr, *J. Org. Chem.*, 2017, **82**, 4148-4159.
2. Z. Zhao, Y. Sun, L. Wang, X. Chen, Y. Sun, L. Lin, Y. Tang, F. Li and D. Chen, *Tetrahedron Lett.*, 2019, **60**, 800-804.
3. H.-J. Knölker and K. R. Reddy, in *The Alkaloids: Chemistry and Biology*, ed. G. A. Cordell, Academic Press, 2008, vol. 65, pp. 3-158.
4. A. A. Haddach, A. Kelleman and M. V. Deaton-Rewolinski, *Tetrahedron Lett.*, 2002, **43**, 399-402.
5. C. M. Miller, E. C. O'Sullivan, K. J. Devine and F. O. McCarthy, *Org. Biomol. Chem.,* 2012, **10**, 7912-7921.
6. D. Pelaprat, R. Oberlin, I. L. Guen, J. B. Le Pecq and B. P. Roques, *J. Med. Chem.*, 1980, **23**, 1330-1335.
7. P. Leon, C. Garbay-Jaureguiberry, B. Lambert, J. B. Le Pecq and B. P. Roques, *J. Med. Chem.*, 1988, **31**, 1021-1026.
8. *CrysalisPro*. (Rigaku Oxford Diffraction, 2019).
9. G. Sheldrick, *Acta Cryst. Section C*, 2015, **71**, 3-8.
10. L. Farrugia, *J. Appl. Cryst.*, 2012, **45**, 849-854.

7 ^{1}H and ^{13}C{^{1}H} NMR, IR and HRMS Spectra for each compound

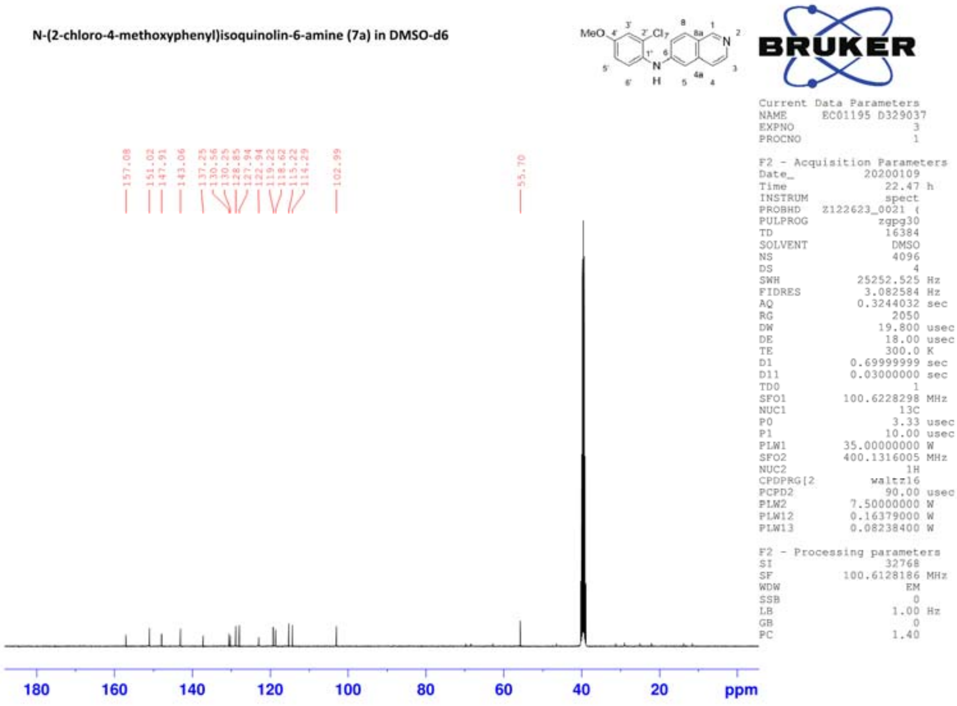

Figure S6. ^{13}C{^{1}H} NMR spectrum of N-(2-chloro-4-methoxyphenyl)isoquinolin-6-amine (**7a**).

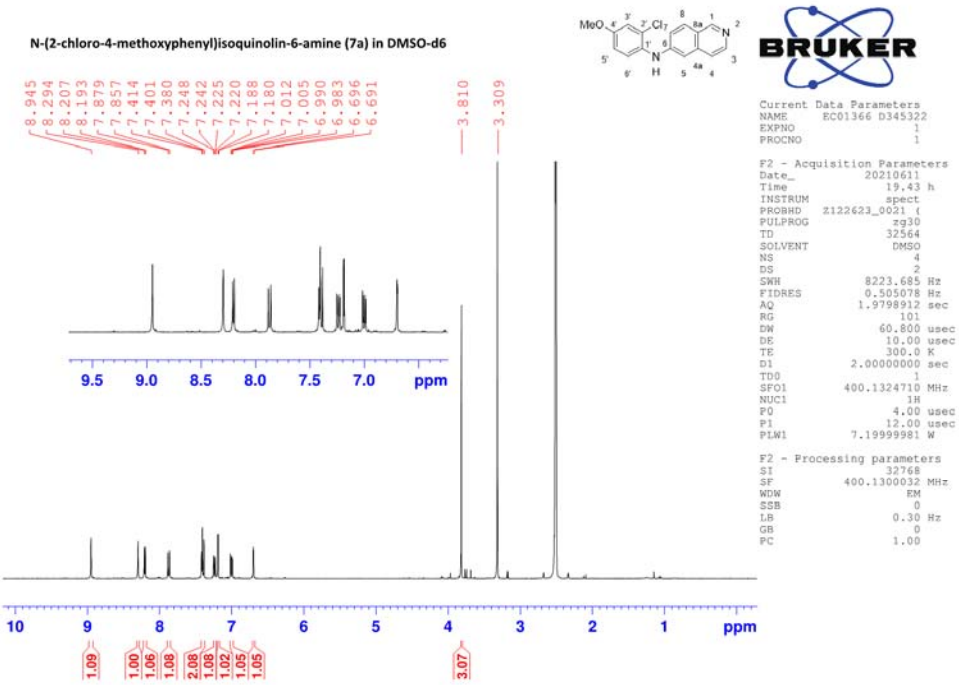

Figure S7. ^{1}H NMR spectrum of N-(2-chloro-4-methoxyphenyl)isoquinolin-6-amine (**7a**).

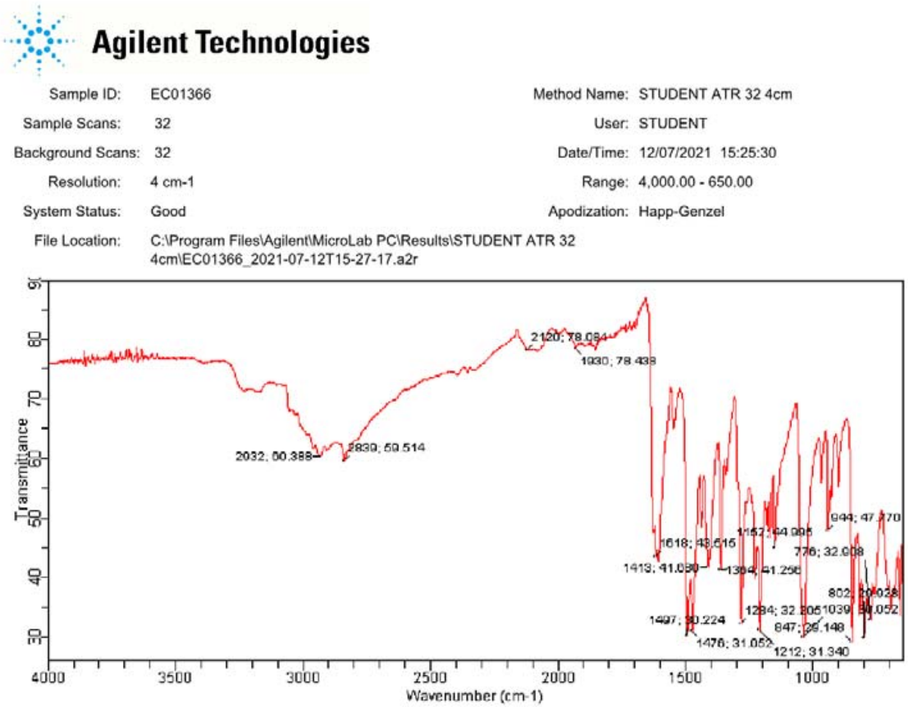

Figure S8. FT-IR spectrum of N-(2-chloro-4-methoxyphenyl)isoquinolin-6-amine (**7a**).

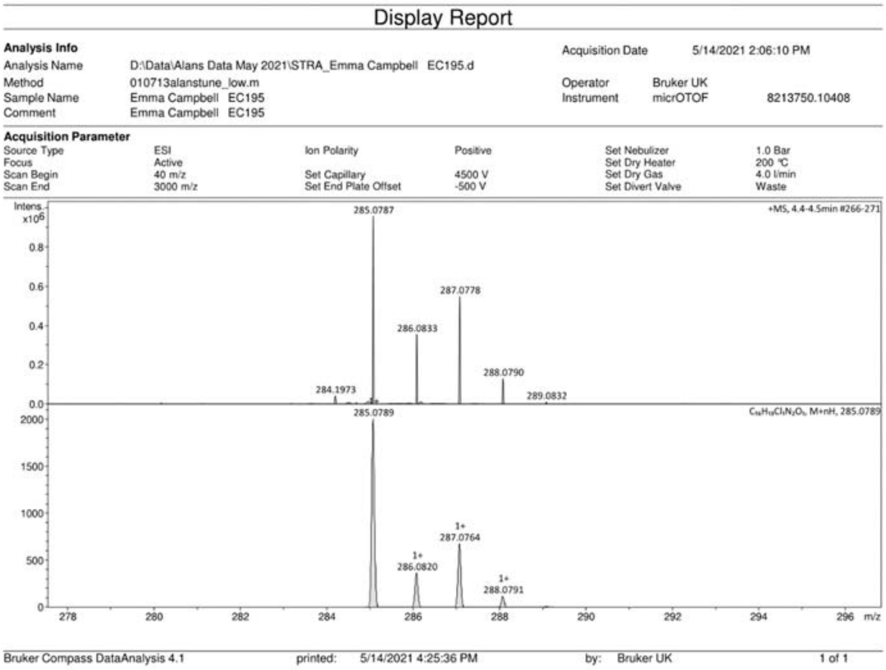

Figure S9. HRMS analysis of N-(2-chloro-4-methoxyphenyl)isoquinolin-6-amine (**7a**).

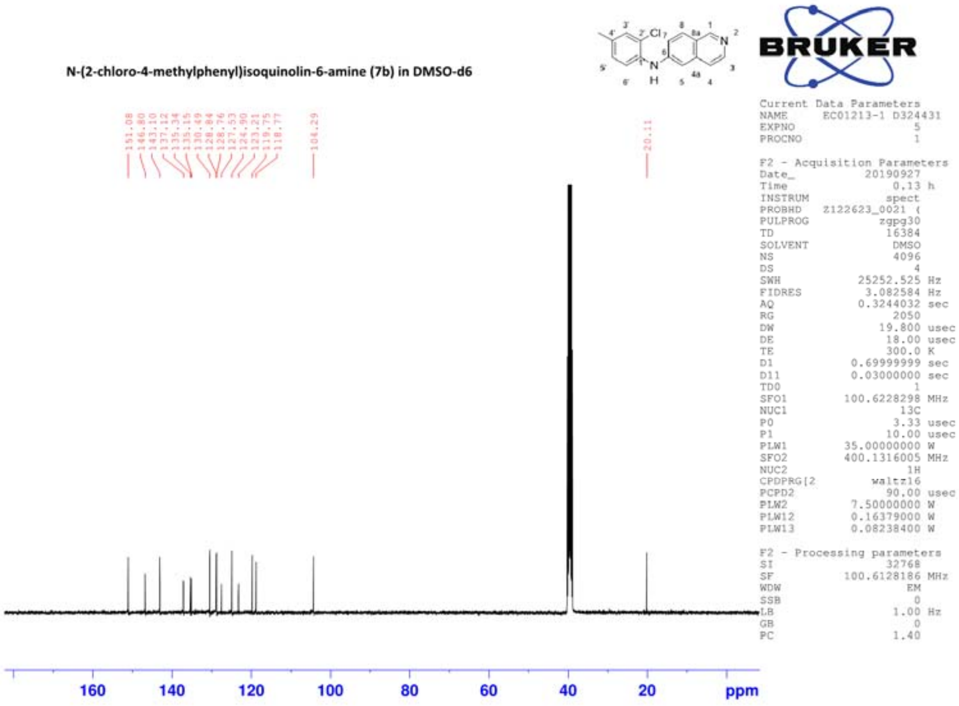

Figure S10. $^{13}C\{^{1}H\}$ NMR spectrum of N-(2-chloro-4-methylphenyl)isoquinolin-6-amine (**7b**).

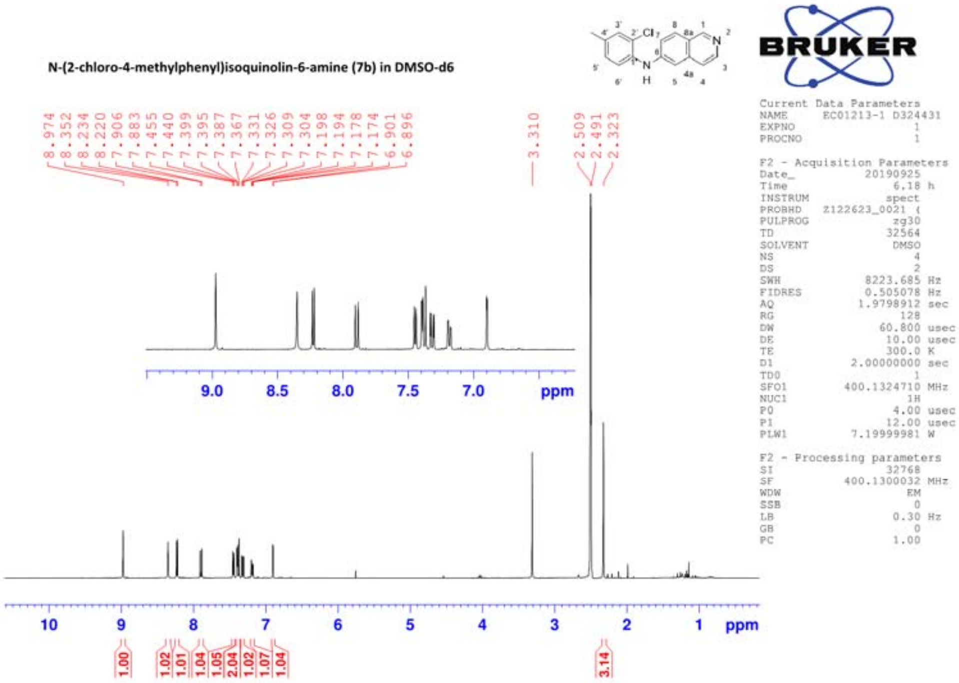

Figure S11. ^{1}H NMR spectrum of N-(2-chloro-4-methylphenyl)isoquinolin-6-amine (**7b**)

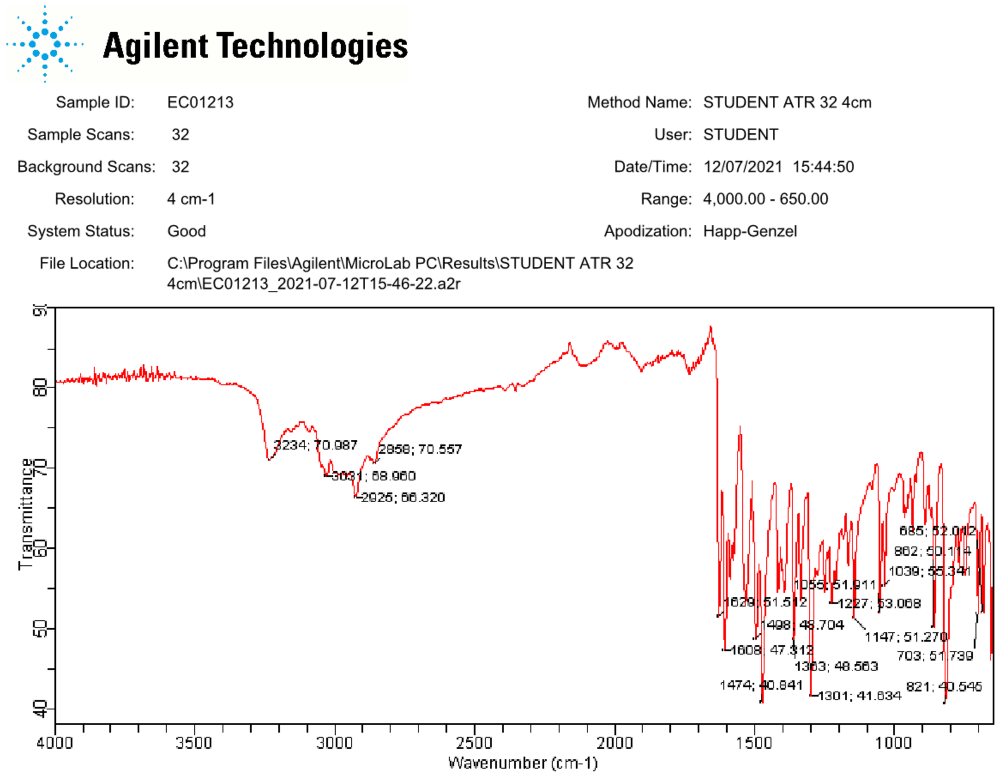

Figure S12. FT-IR spectrum of N-(2-chloro-4-methylphenyl)isoquinolin-6-amine (**7b**).

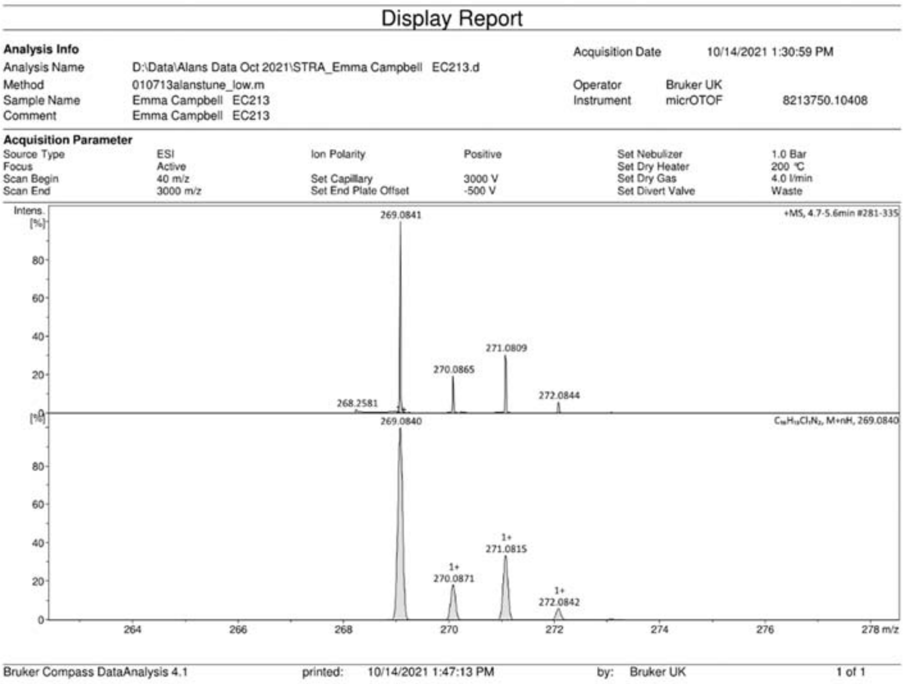

Figure S13. HRMS analysis of N-(2-chloro-4-methylphenyl)isoquinolin-6-amine (**7b**).

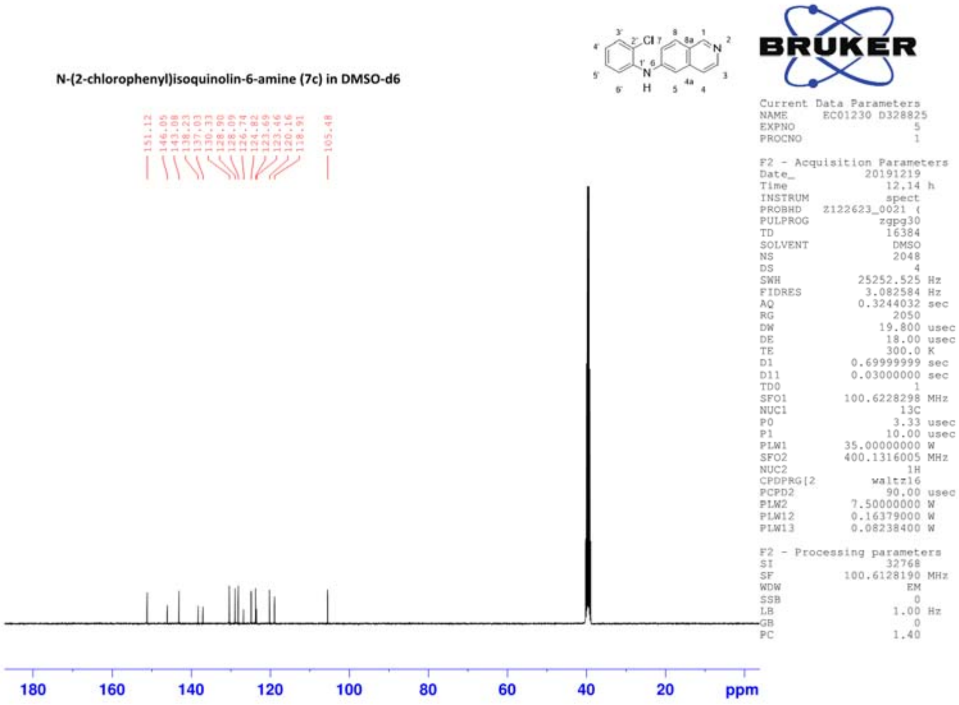

Figure S14. $^{13}C\{^{1}H\}$ NMR spectrum of N-(2-chlorophenyl)isoquinolin-6-amine (**7c**).

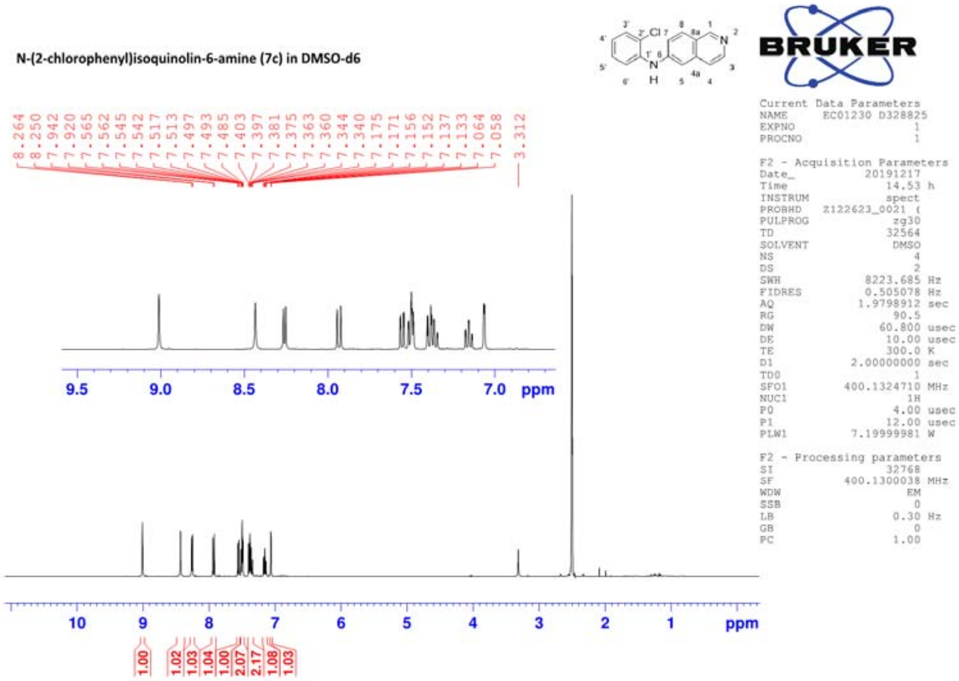

Figure S15. ^{1}H NMR spectrum of N-(2-chlorophenyl)isoquinolin-6-amine (**7c**).

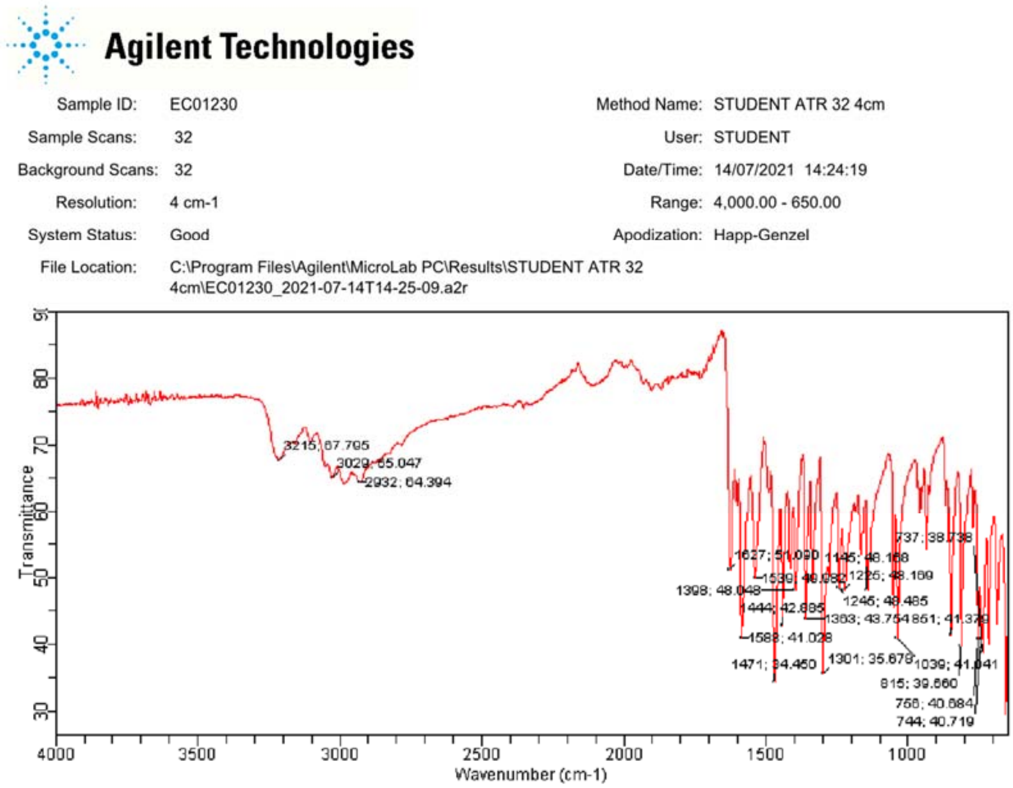

Figure S16. FT-IR spectrum of N-(2-chlorophenyl)isoquinolin-6-amine (**7c**).

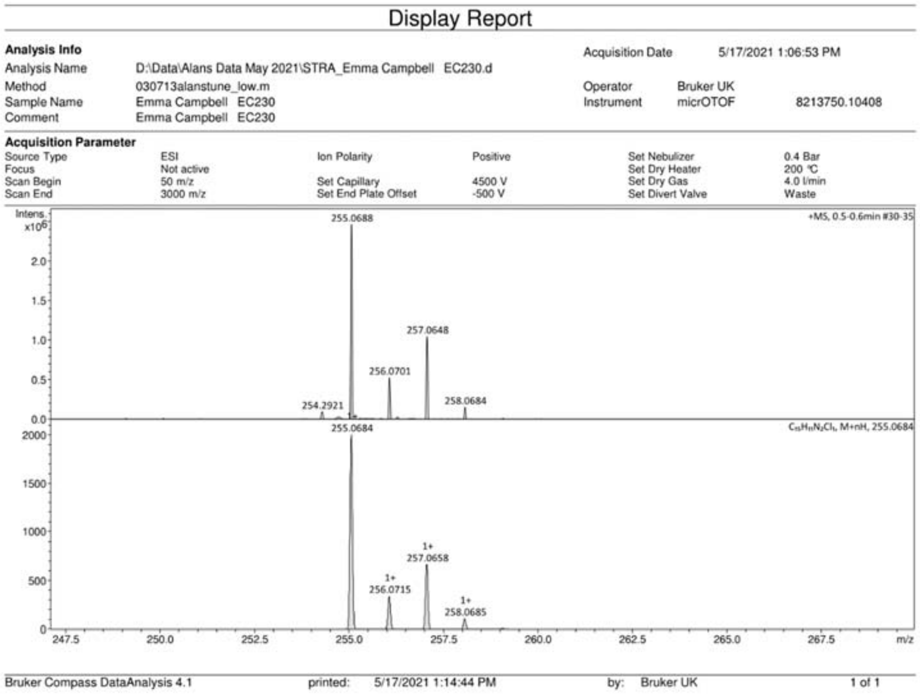

Figure S17. HRMS analysis of N-(2-chlorophenyl)isoquinolin-6-amine (**7c**).

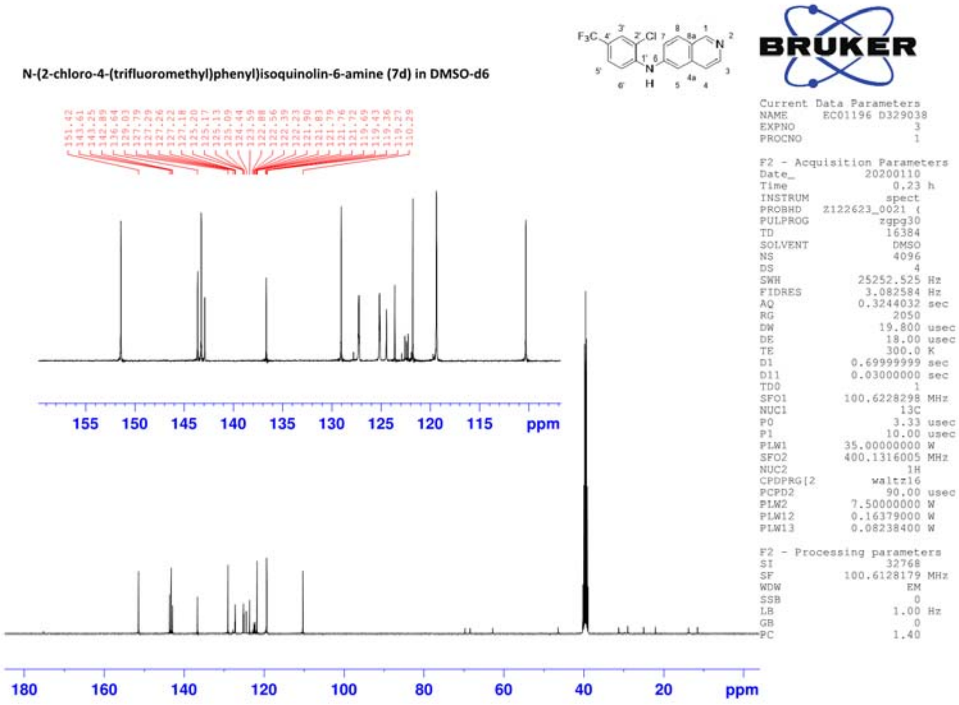

Figure S18. $^{13}C\{^1H\}$ NMR spectrum of N-(2-chloro-4-(trifluoromethyl)phenyl)isoquinolin-6-amine (**7d**).

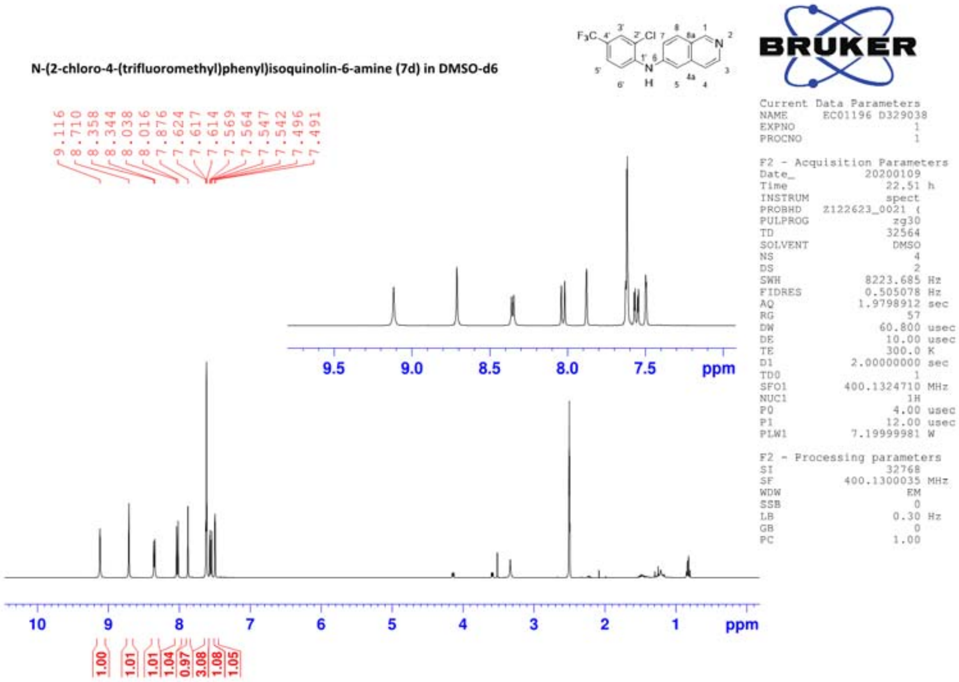

Figure S19. ^{1}H NMR spectrum of N-(2-chloro-4-(trifluoromethyl)phenyl)isoquinolin-6-amine (**7d**).

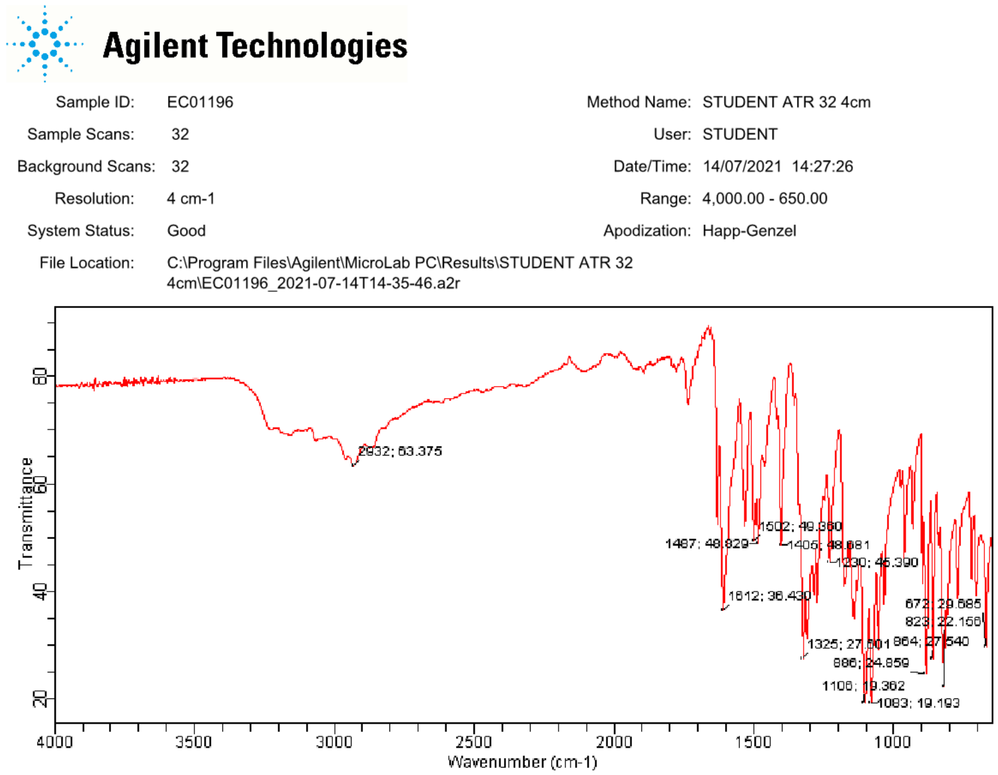

Figure S20. FT-IR spectrum of N-(2-chloro-4-(trifluoromethyl)phenyl)isoquinolin-6-amine (**7d**).

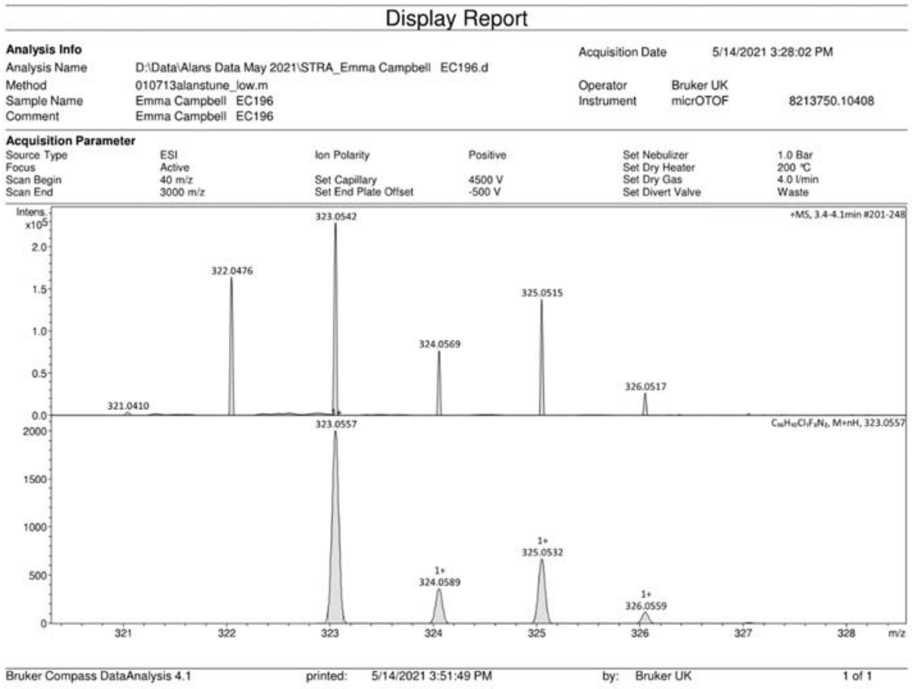

Figure S21. HRMS analysis of N-(2-chloro-4-(trifluoromethyl)phenyl)isoquinolin-6-amine (**7d**).

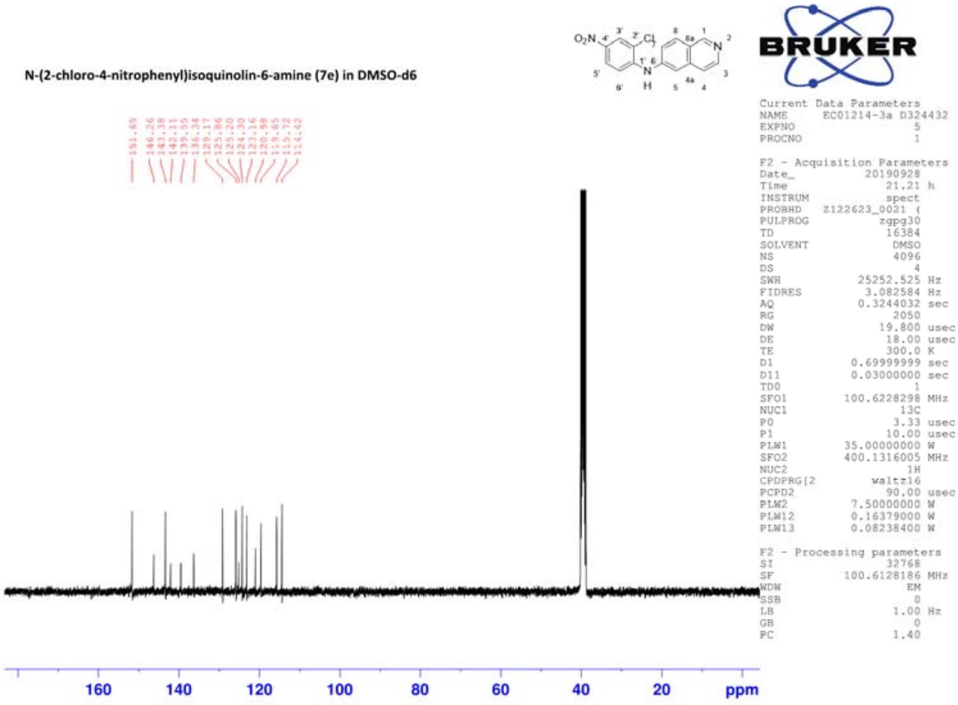

Figure S22. $^{13}C\{^{1}H\}$ NMR spectrum of N-(2-chloro-4-nitrophenyl)isoquinolin-6-amine (**7e**).

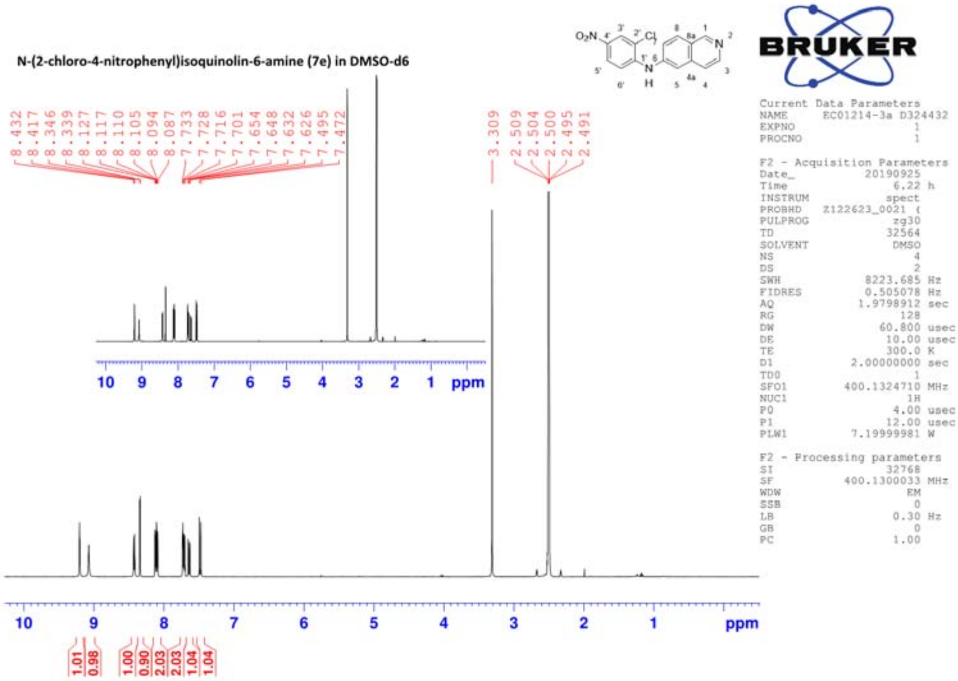

Figure S23. ^{1}H NMR spectrum of N-(2-chloro-4-nitrophenyl)isoquinolin-6-amine (**7e**).

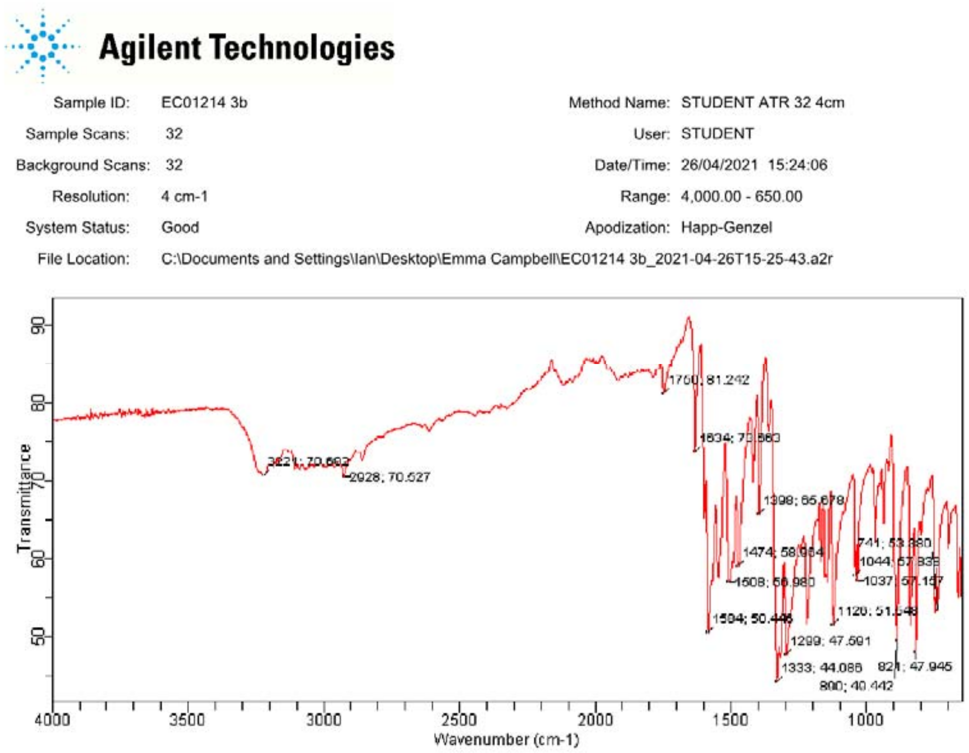

Figure S24. FT-IR spectrum of N-(2-chloro-4-nitrophenyl)isoquinolin-6-amine (**7e**).

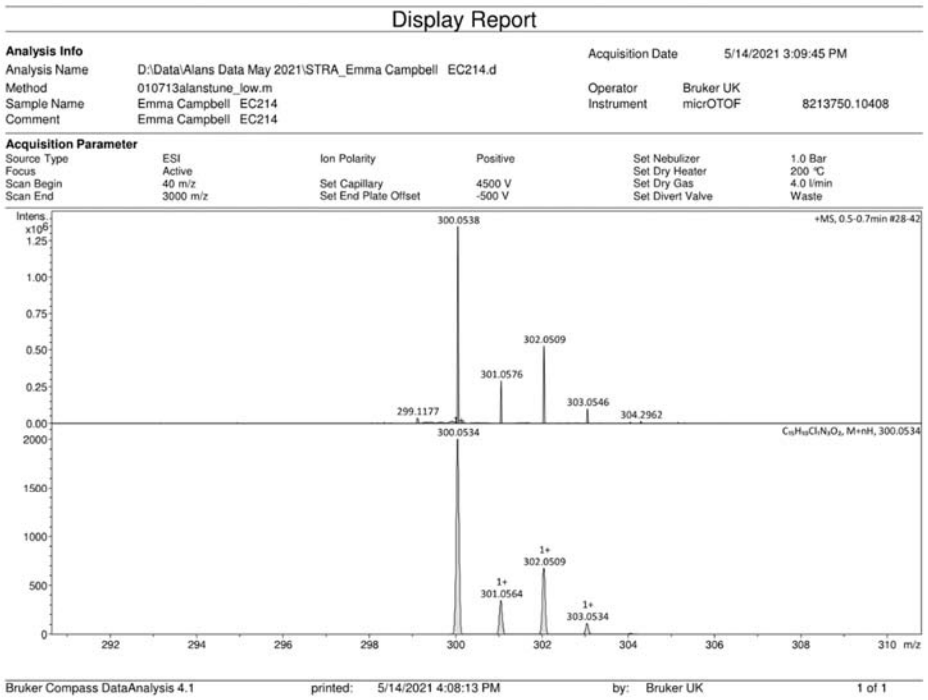

Figure S25. HRMS analysis of N-(2-chloro-4-nitrophenyl)isoquinolin-6-amine (**7e**).

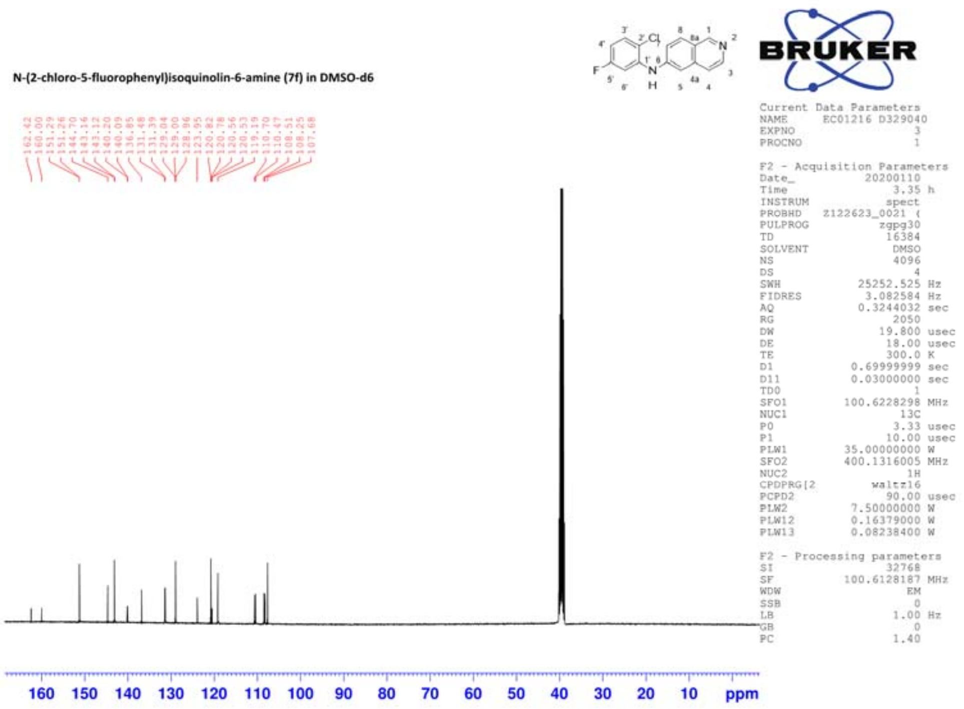

Figure S26. $^{13}C\{^{1}H\}$ NMR spectrum of N-(2-chloro-5-fluorophenyl)isoquinolin-6-amine (**7f**).

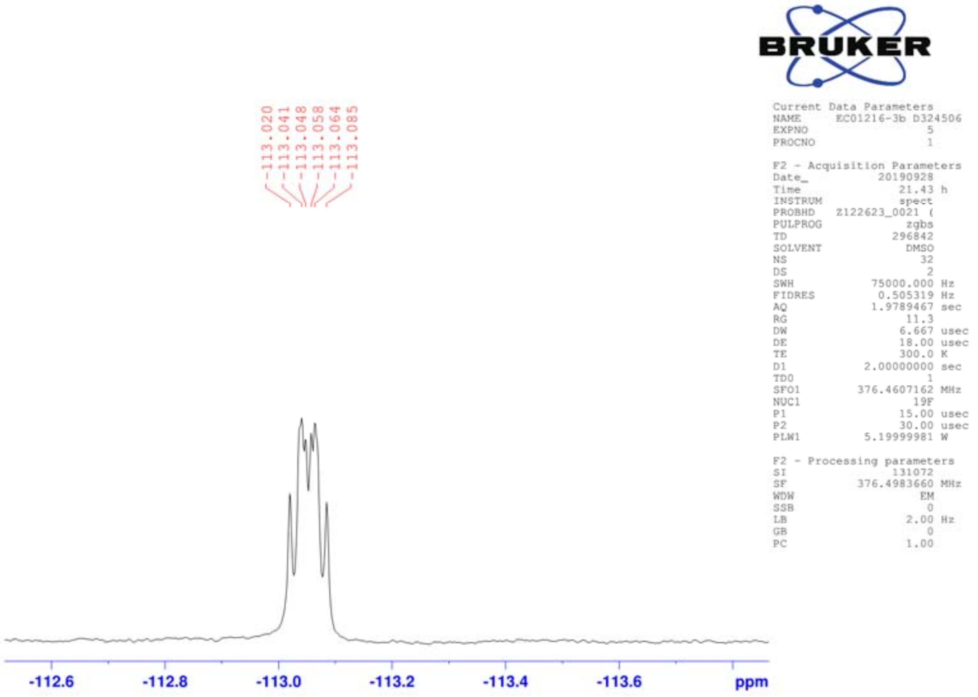

Figure S27. ^{19}F NMR spectrum of N-(2-chloro-5-fluorophenyl)isoquinolin-6-amine (**7f**).

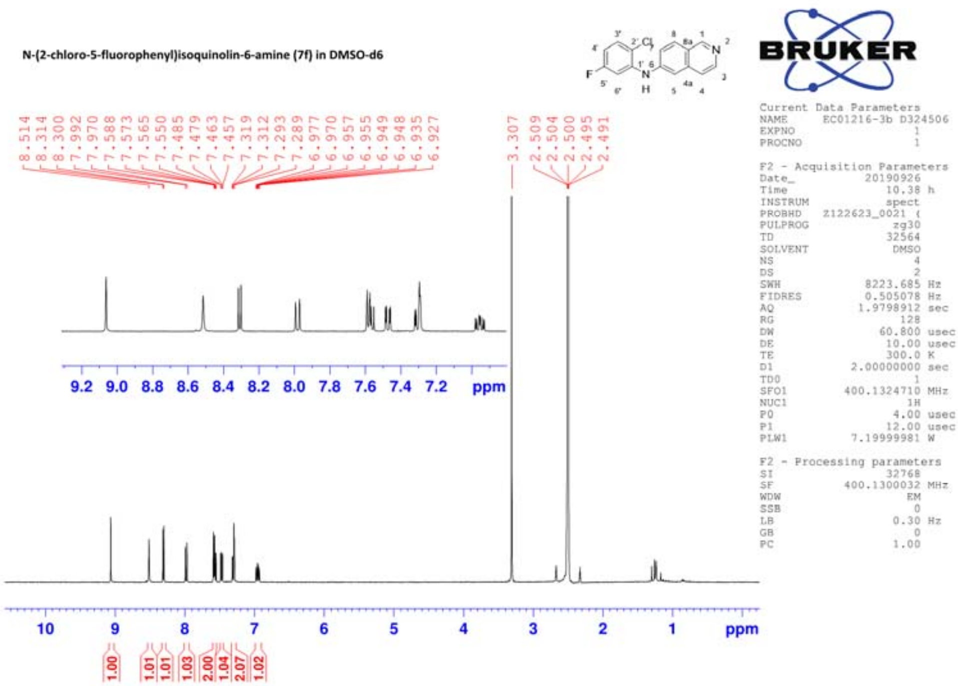

Figure S28. ^{1}H NMR spectrum of N-(2-chloro-5-fluorophenyl)isoquinolin-6-amine (**7f**).

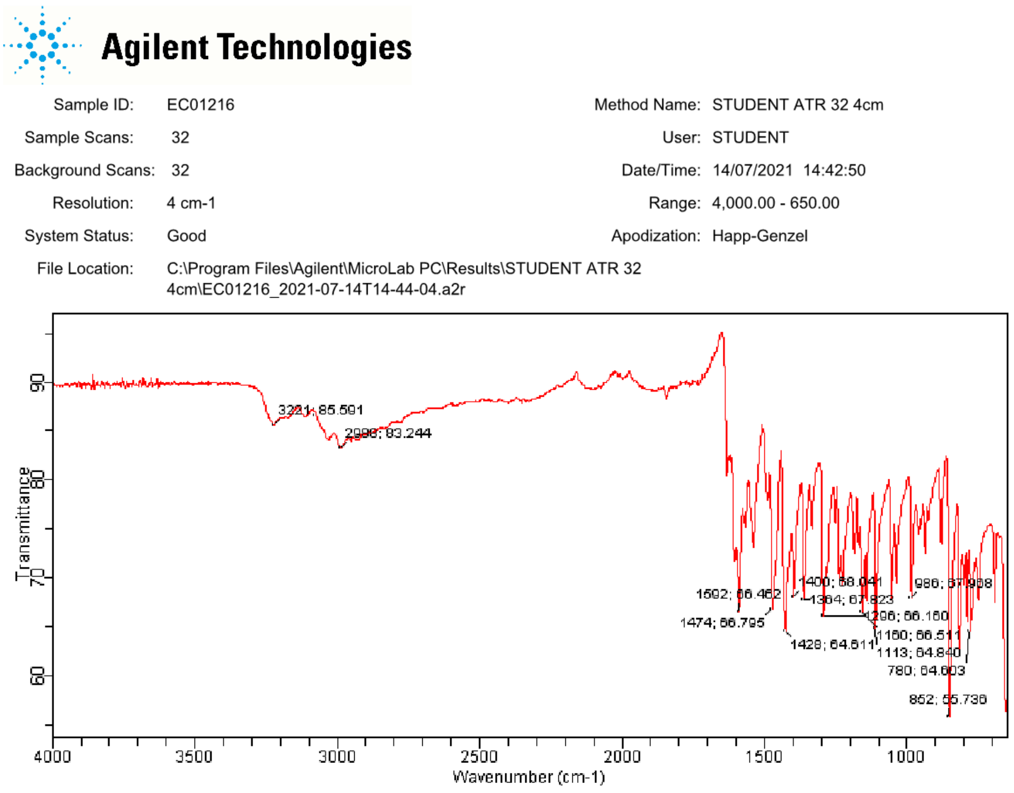

Figure S29. FT-IR spectrum of N-(2-chloro-5-fluorophenyl)isoquinolin-6-amine (**7f**).

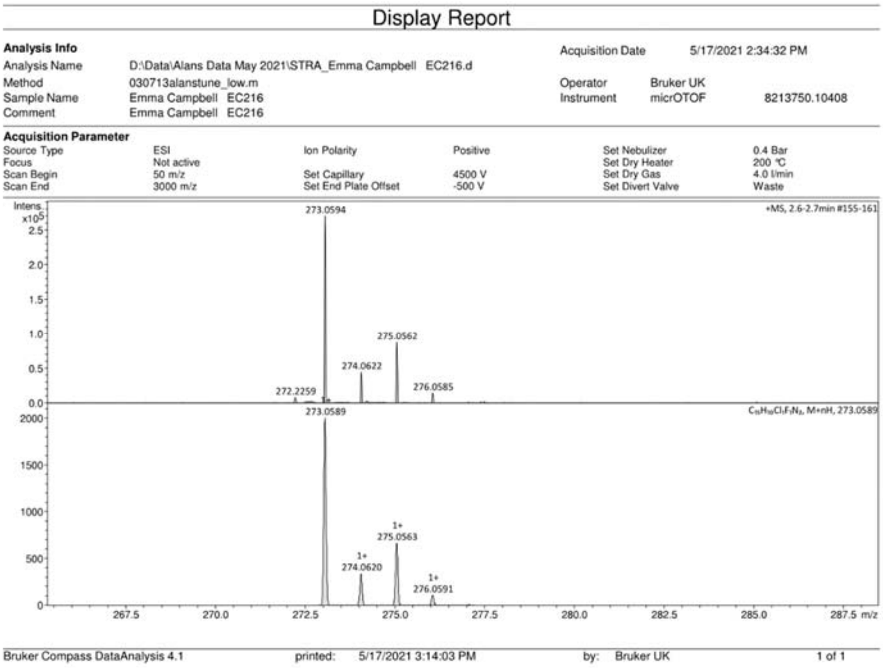

Figure S30. HRMS analysis of N-(2-chloro-5-fluorophenyl)isoquinolin-6-amine (**7f**).

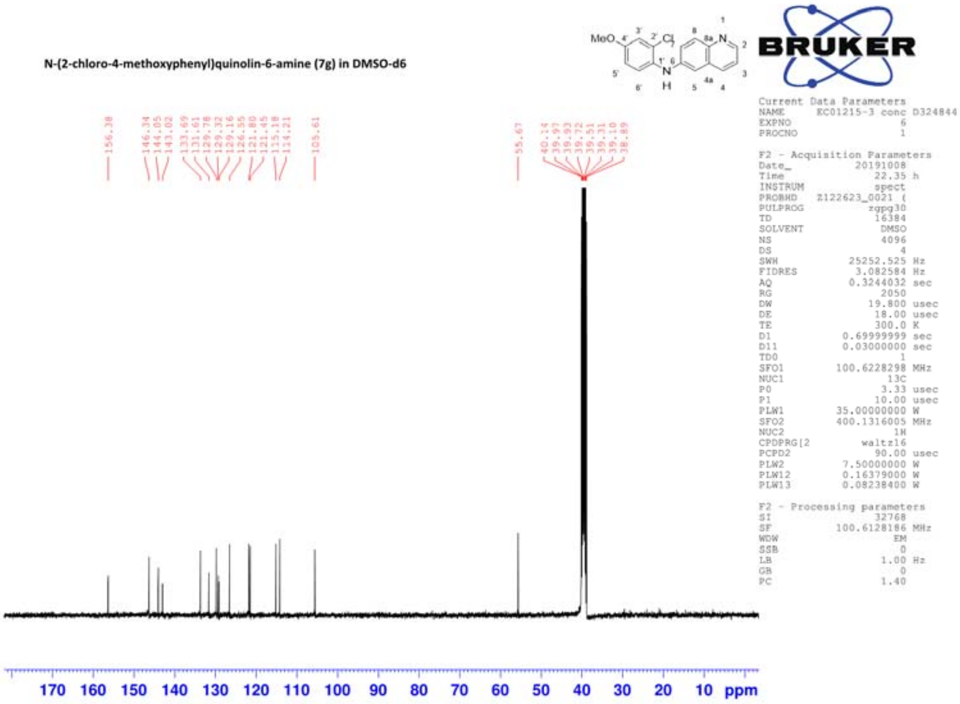

Figure S31. $^{13}C\{^{1}H\}$ NMR spectrum of N-(2-chloro-4-methoxyphenyl)quinolin-6-amine (**7g**).

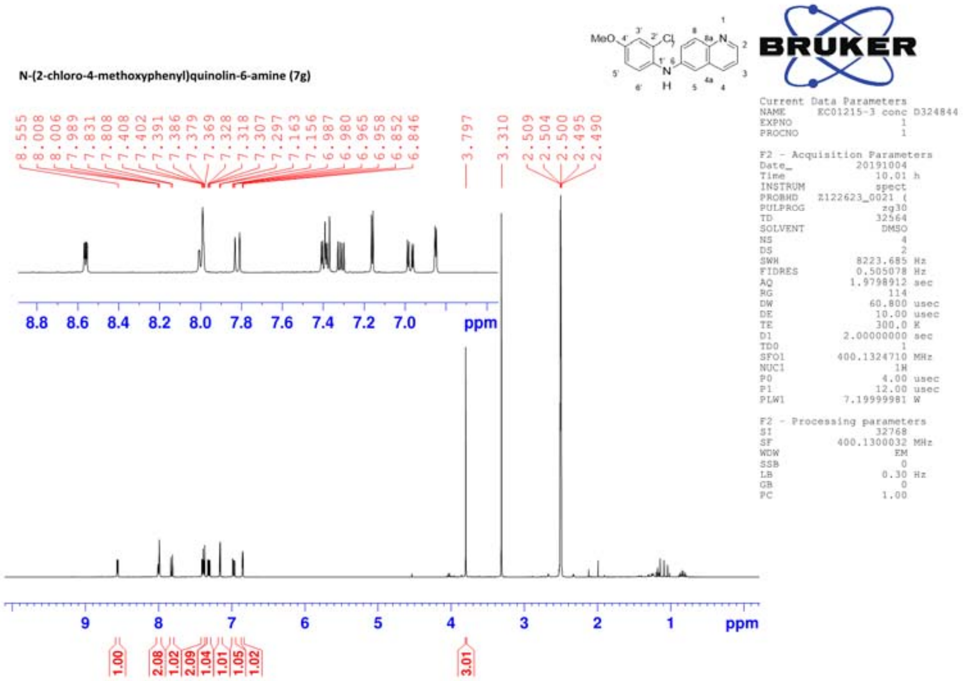

Figure S32. ¹H NMR spectrum of N-(2-chloro-4-methoxyphenyl)quinolin-6-amine (**7g**).

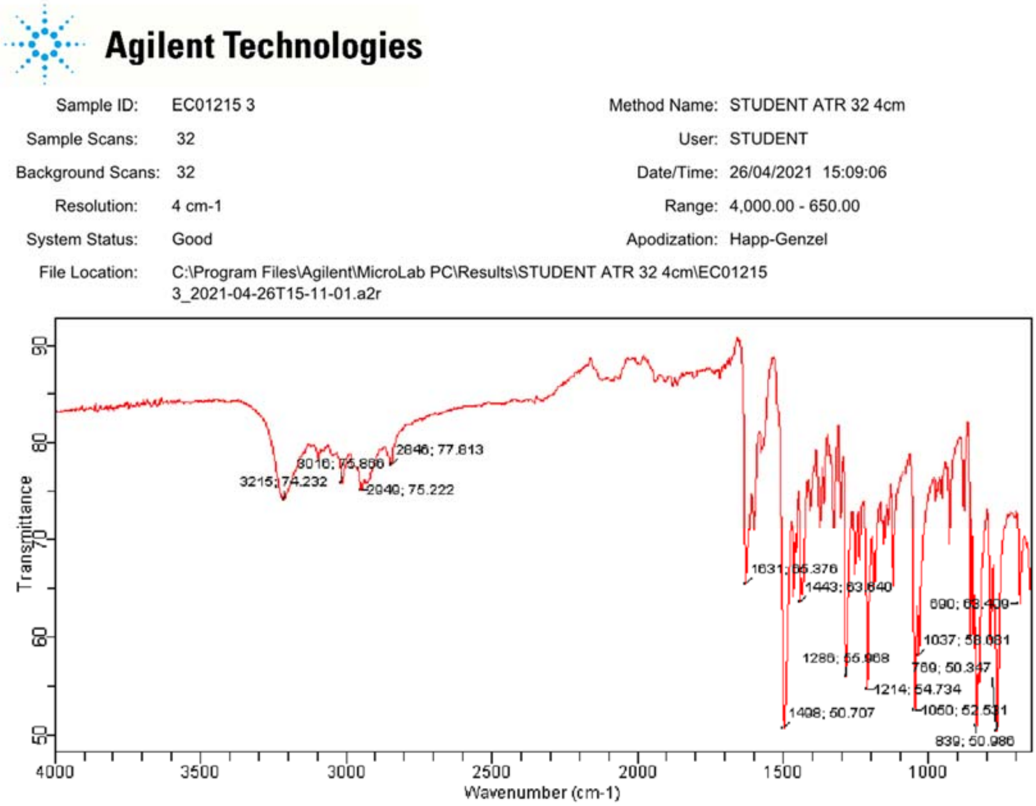

Figure S33. FT-IR spectrum of N-(2-chloro-4-methoxyphenyl)quinolin-6-amine (**7g**).

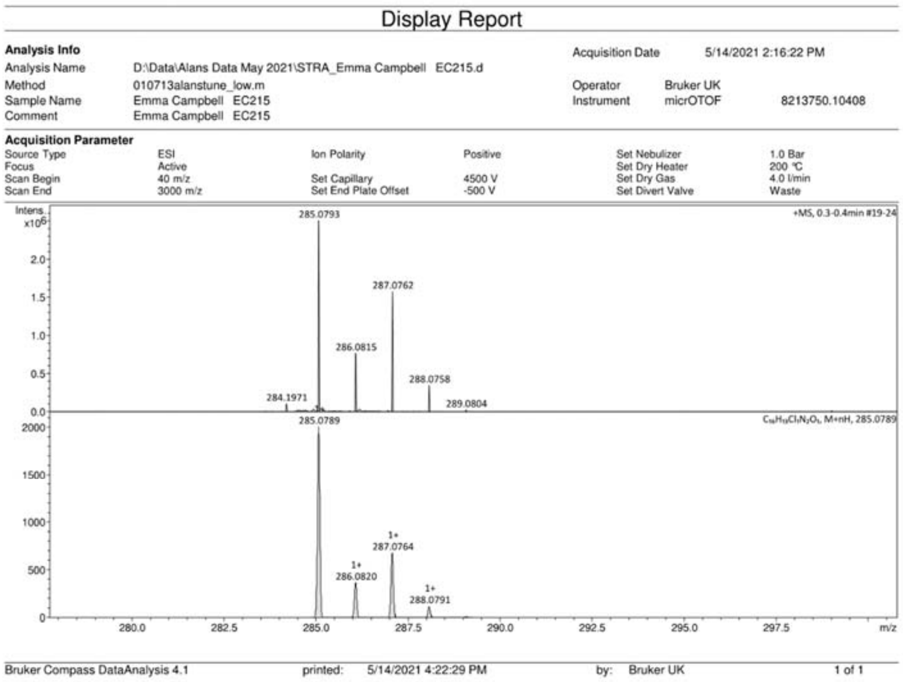

Figure S34. HRMS analysis of N-(2-chloro-4-methoxyphenyl)quinolin-6-amine (**7g**).

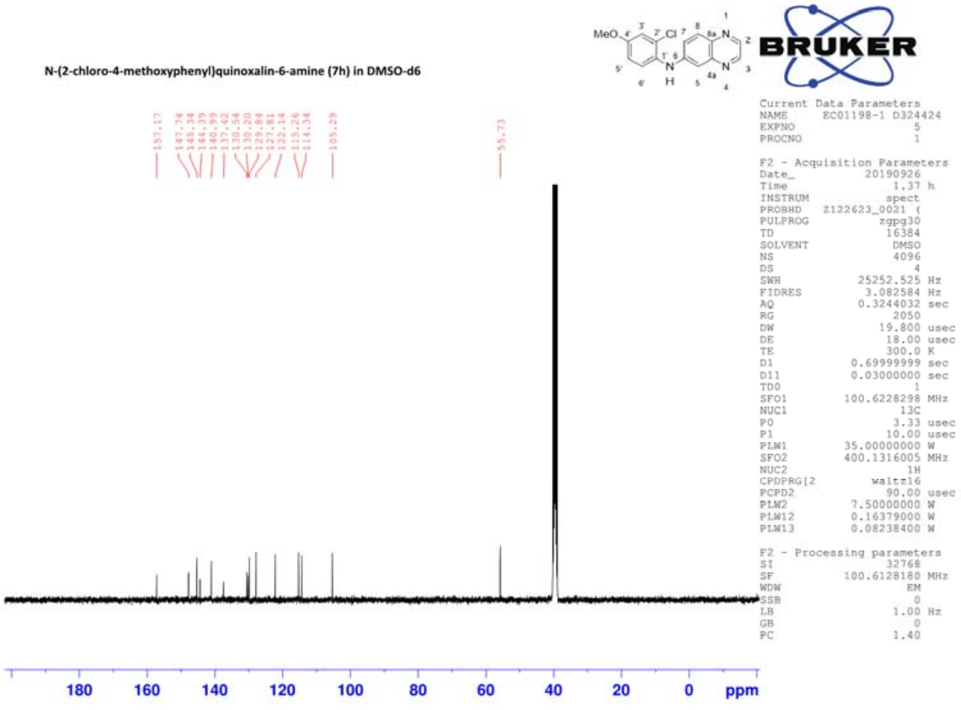

Figure S35. $^{13}C\{^{1}H\}$ NMR spectrum of N-(2-chloro-4-methoxyphenyl)quinoxalin-6-amine (**7h**).

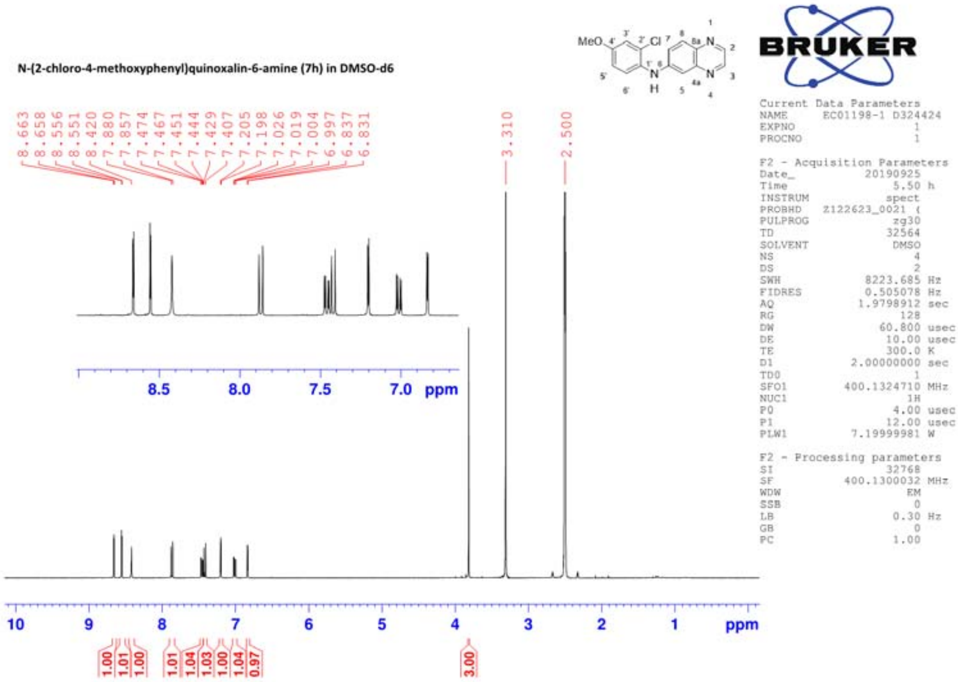

Figure S36. ^{1}H NMR spectrum of N-(2-chloro-4-methoxyphenyl)quinoxalin-6-amine (**7h**).

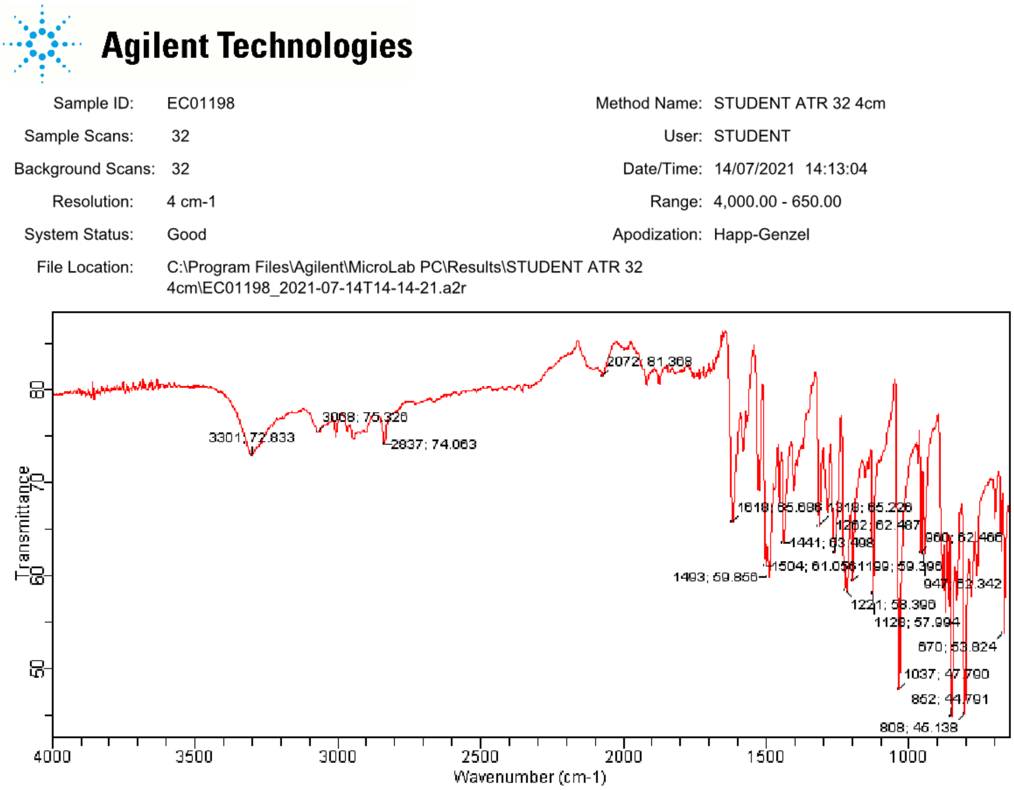

Figure S37. FT-IR spectrum of N-(2-chloro-4-methoxyphenyl)quinoxalin-6-amine (**7h**).

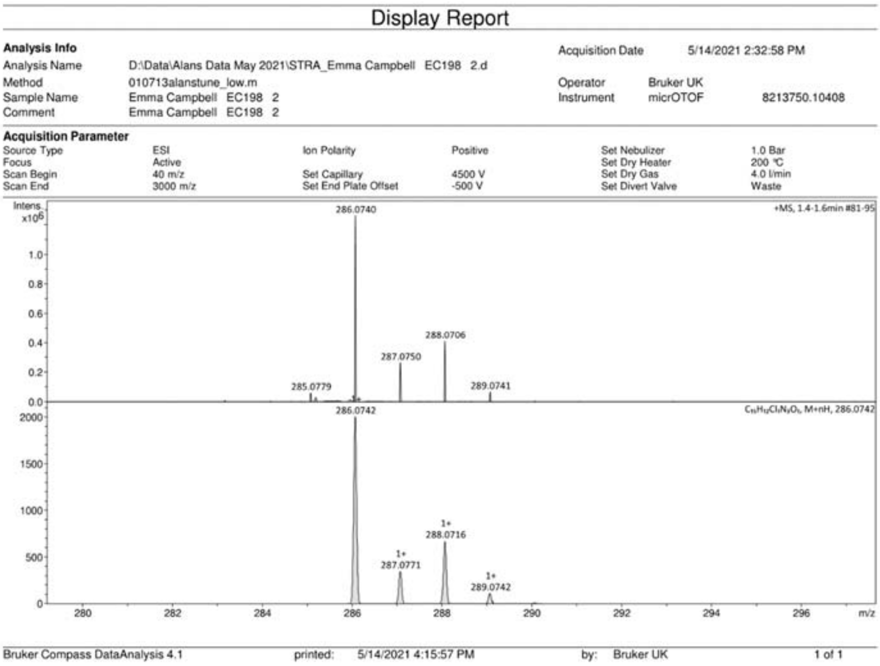

Figure S38. HRMS analysis of N-(2-chloro-4-methoxyphenyl)quinoxalin-6-amine (**7h**).

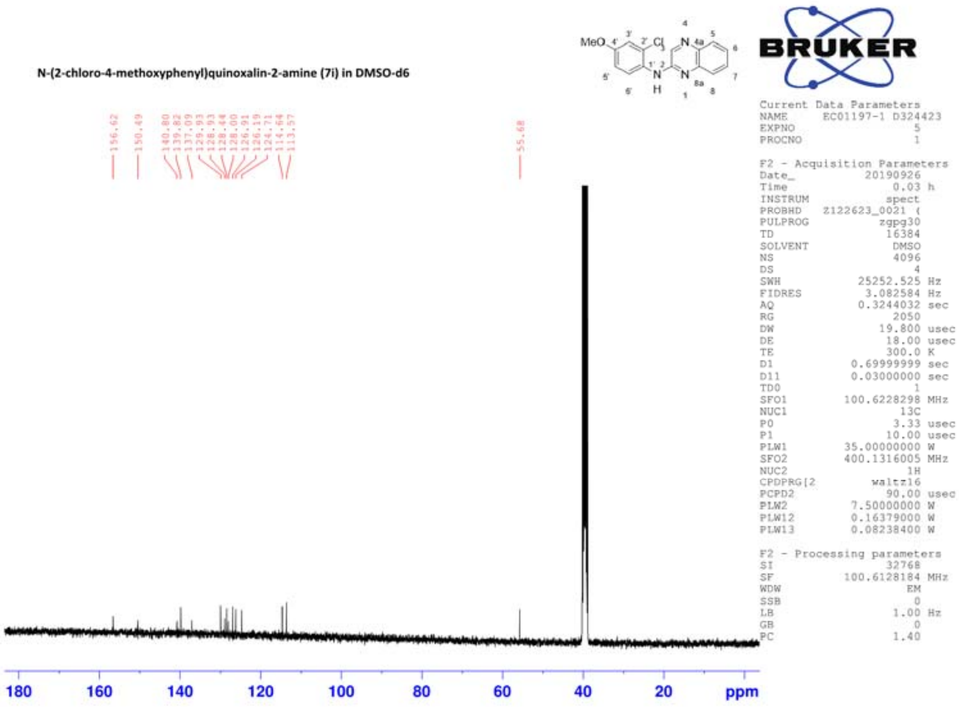

Figure S39. $^{13}C\{^{1}H\}$ NMR spectrum of N-(2-chloro-4-methoxyphenyl)quinoxalin-2-amine (**7i**).

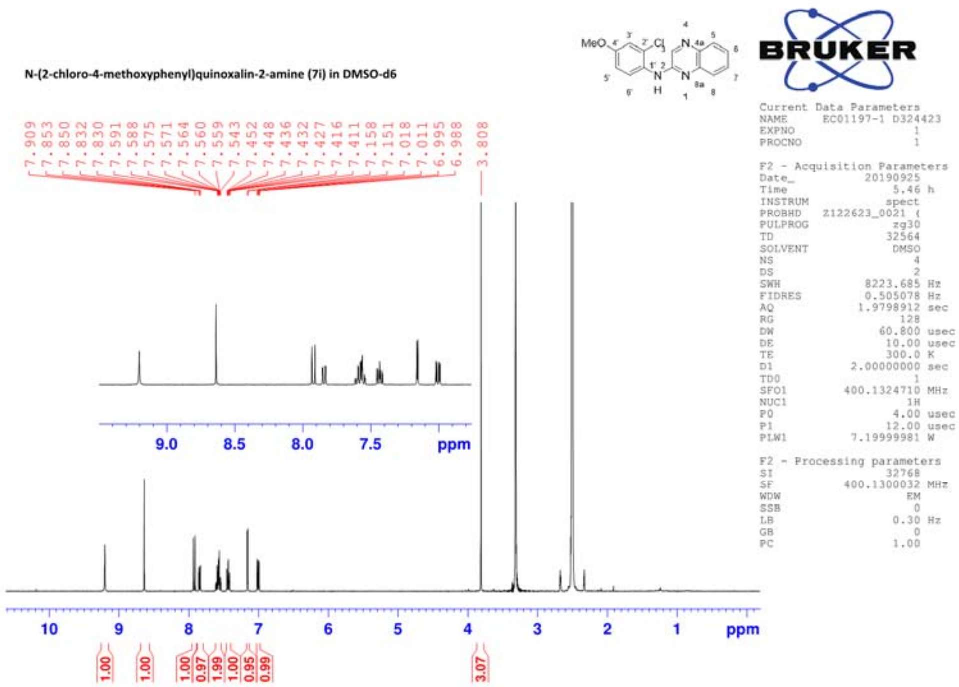

Figure S40. ^{1}H NMR spectrum of N-(2-chloro-4-methoxyphenyl)quinoxalin-2-amine (**7i**) .

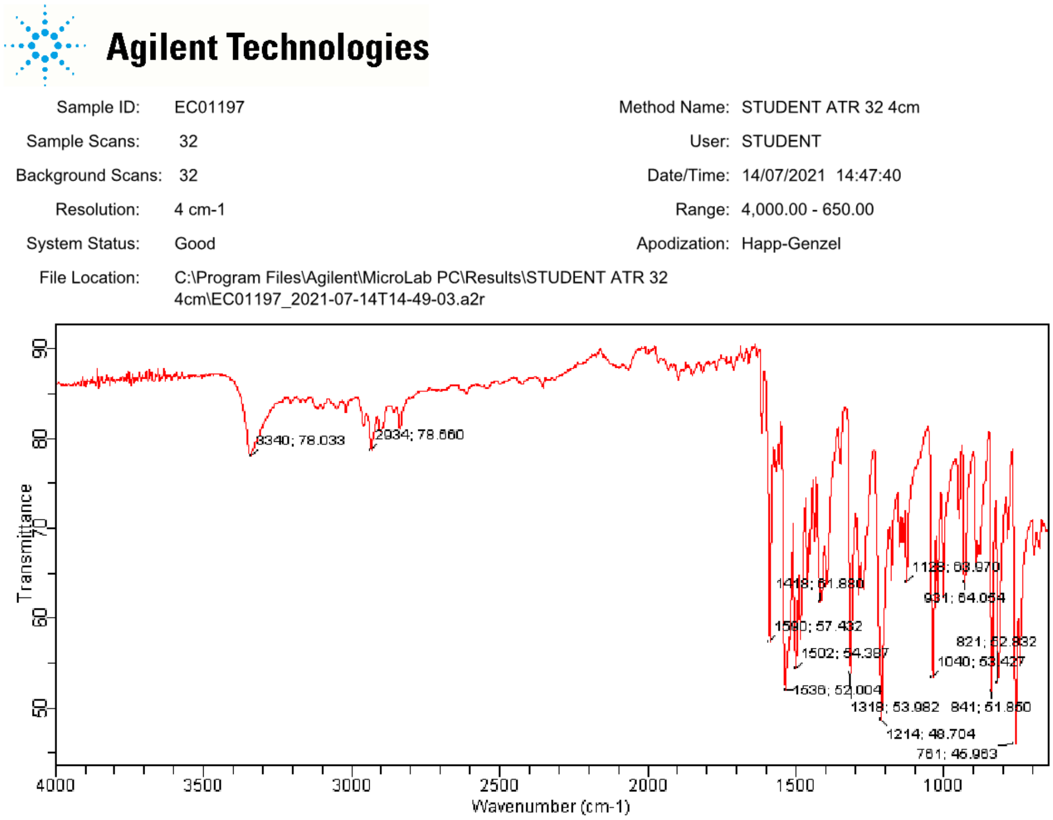

Figure S41. FT-IR spectrum of N-(2-chloro-4-methoxyphenyl)quinoxalin-2-amine (**7i**).

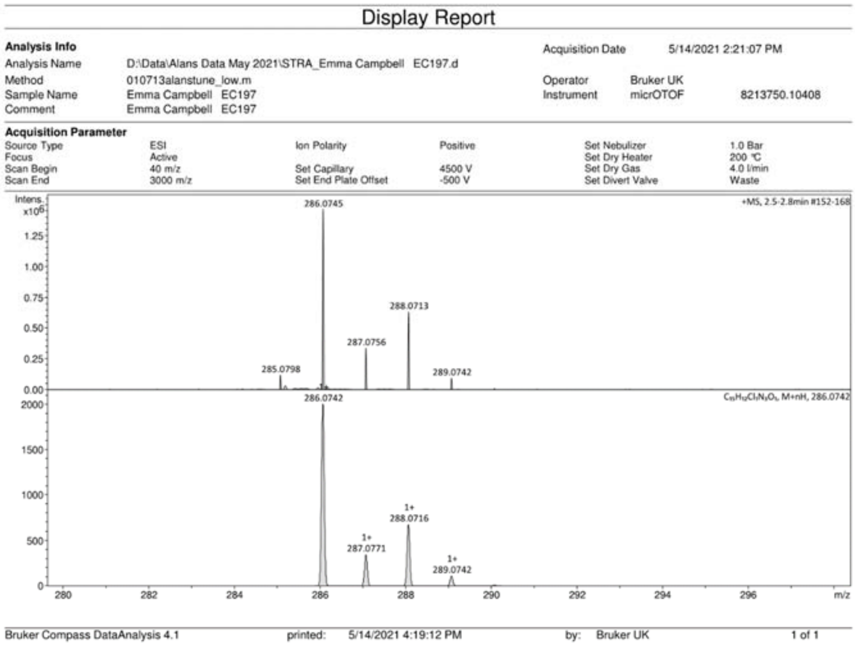

Figure S42. HRMS analysis of N-(2-chloro-4-methoxyphenyl)quinoxalin-2-amine (**7i**).

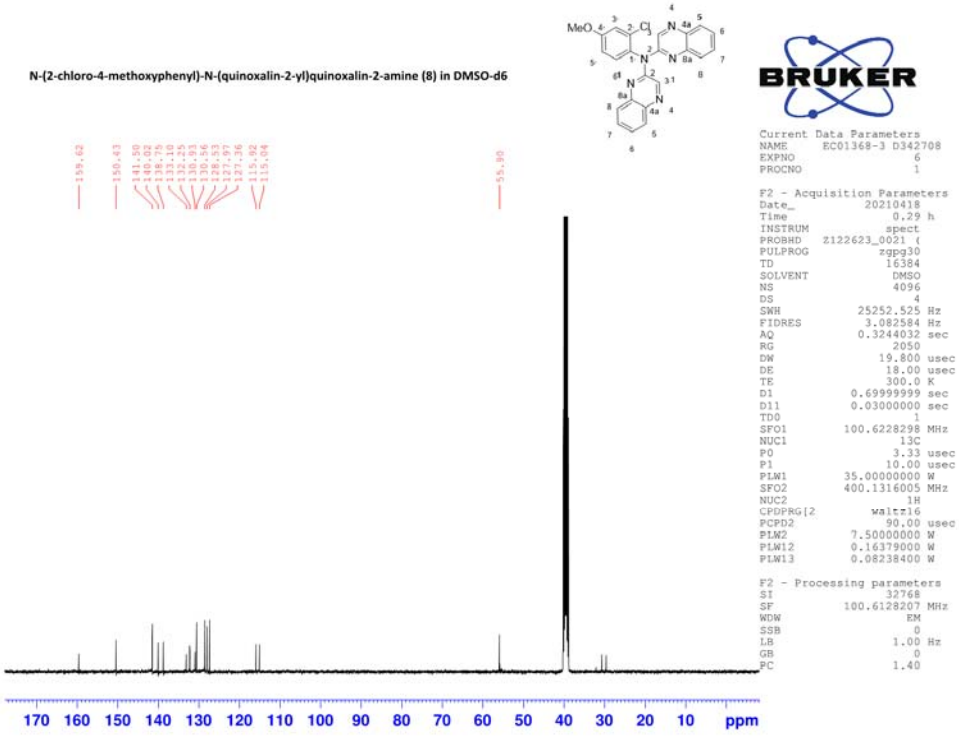

Figure S43. $^{13}C\{^1H\}$ NMR spectrum of N-(2-chloro-4-methoxyphenyl)-N-(quinoxalin-2-yl)quinoxalin-2-amine (**8**).

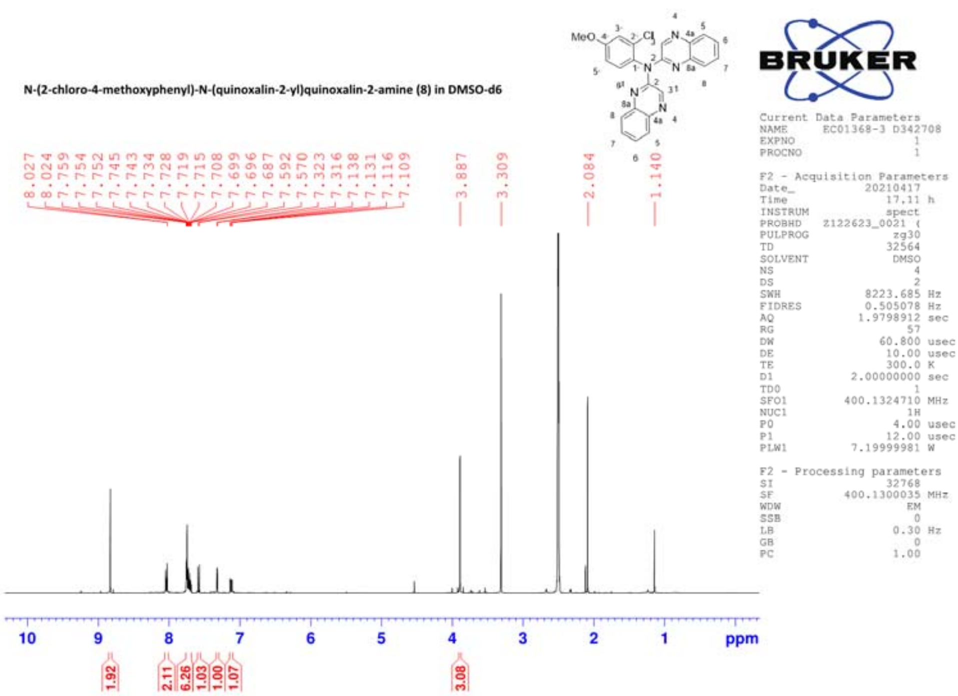

Figure S44. ^{1}H NMR spectrum of N-(2-chloro-4-methoxyphenyl)-N-(quinoxalin-2-yl)quinoxalin-2-amine (**8**).

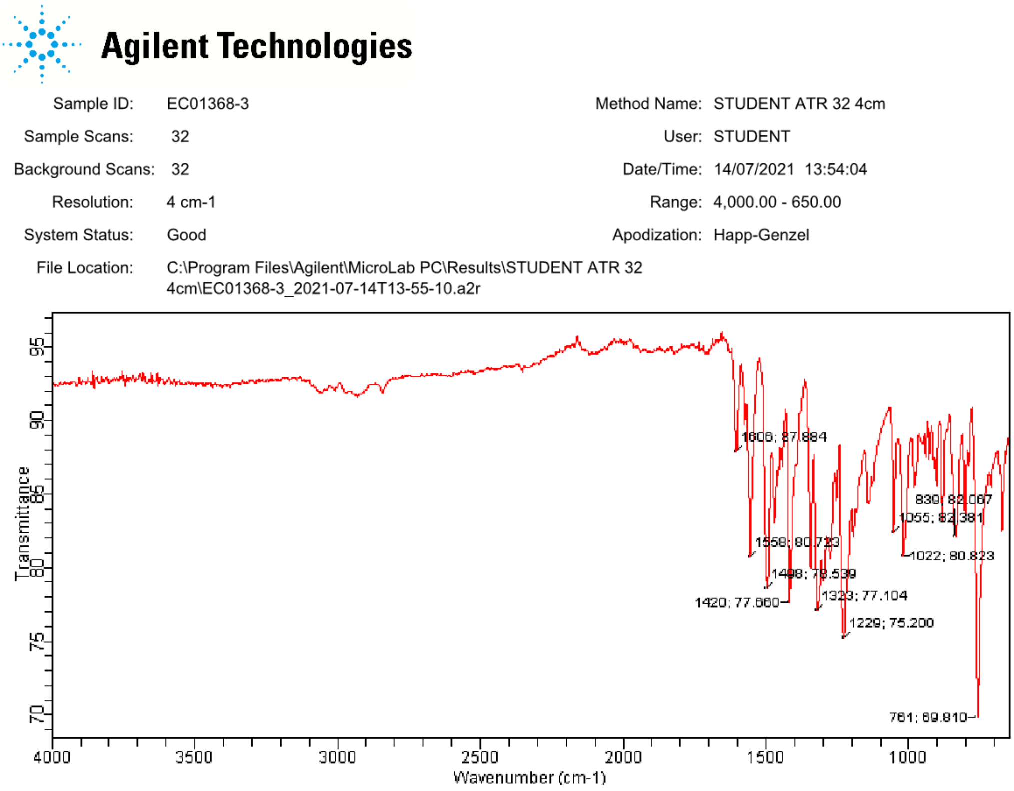

Figure S45. FT-IR spectrum of N-(2-chloro-4-methoxyphenyl)-N-(quinoxalin-2-yl)quinoxalin-2-amine (**8**).

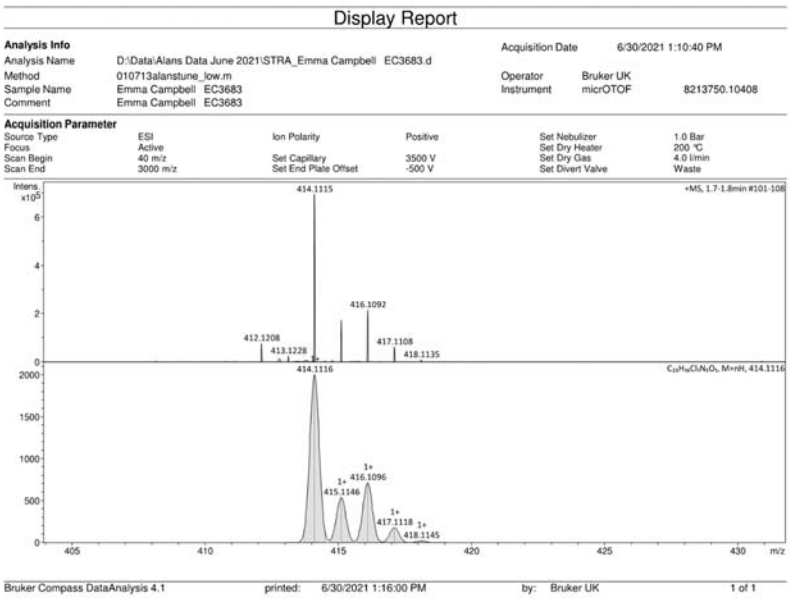

Figure S46. HRMS analysis of N-(2-chloro-4-methoxyphenyl)-N-(quinoxalin-2-yl)quinoxalin-2-amine (**8**).

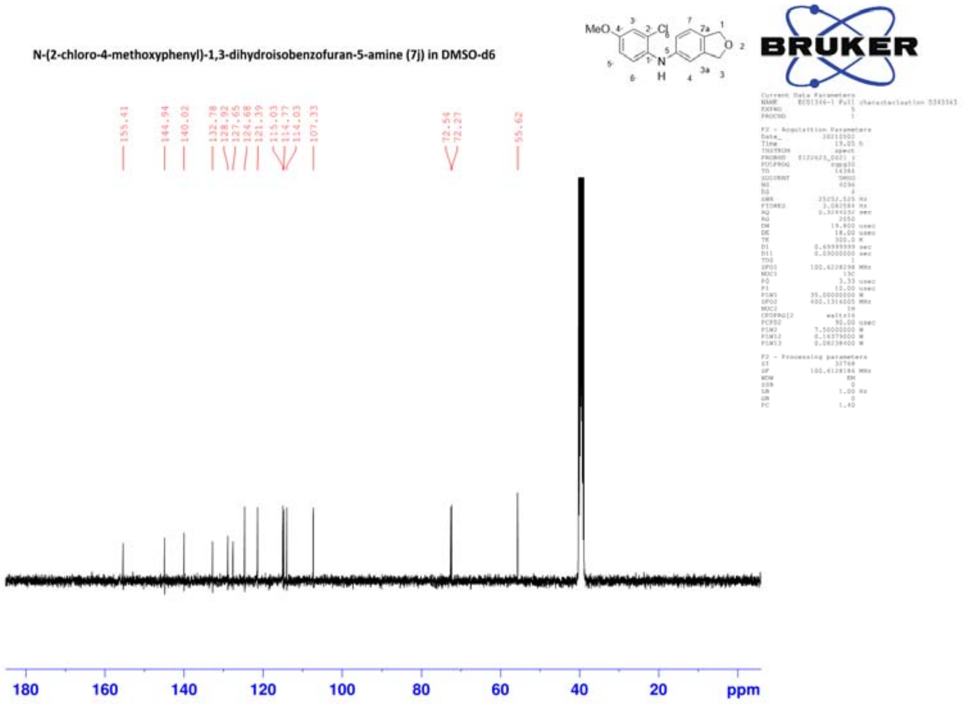

Figure S47. $^{13}C\{^{1}H\}$ NMR spectrum of N-(2-chloro-4-methoxyphenyl)-1,3-dihydroisobenzofuran-5-amine (**7j**).

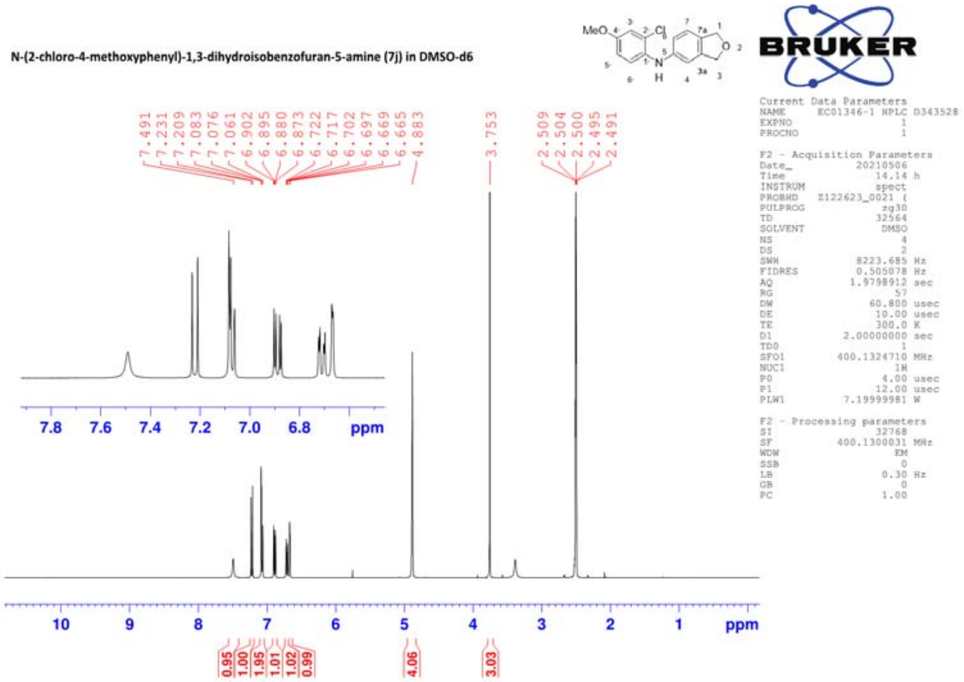

Figure S48. ^1H NMR spectrum of N-(2-chloro-4-methoxyphenyl)-1,3-dihydroisobenzofuran-5-amine (**7j**).

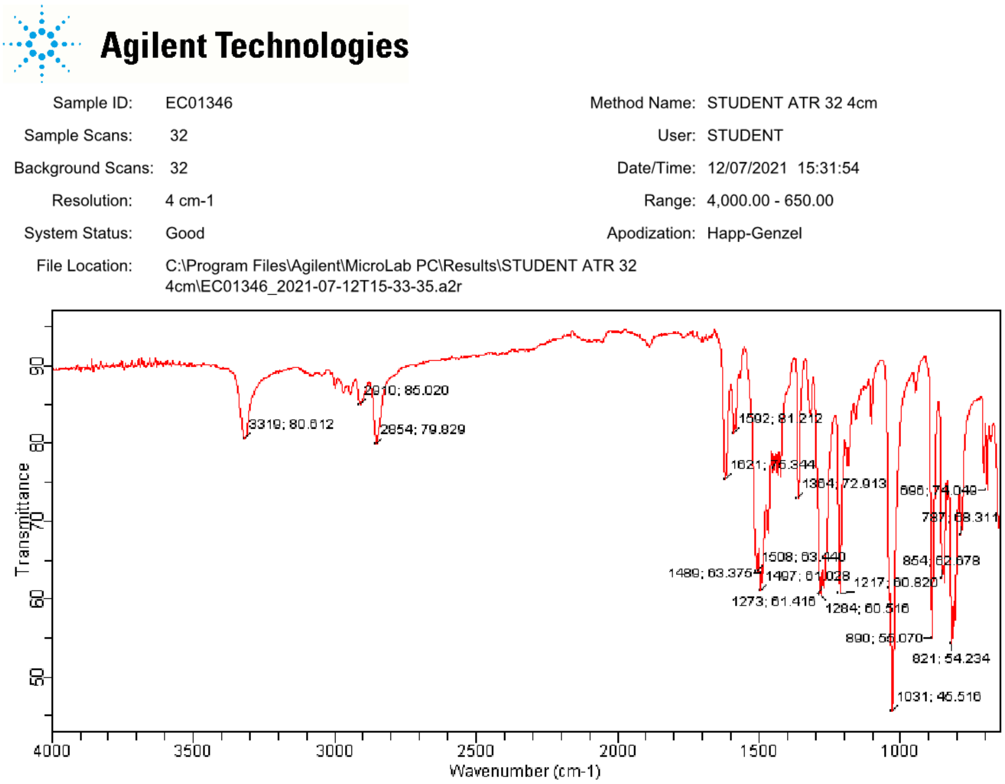

Figure S49. FT-IR spectrum of N-(2-chloro-4-methoxyphenyl)-1,3-dihydroisobenzofuran-5-amine (**7j**).

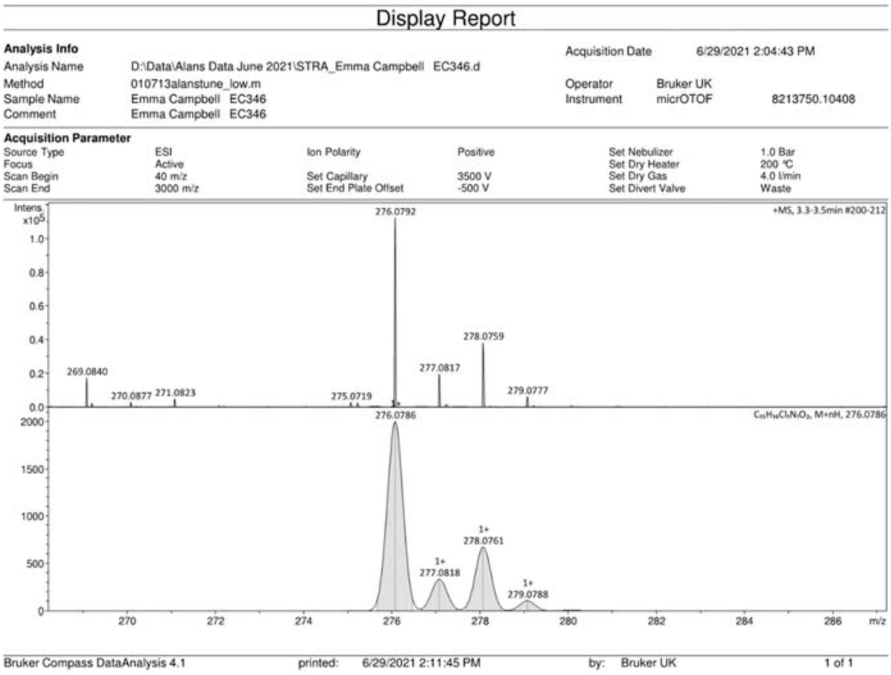

Figure S50. HRMS analysis of N-(2-chloro-4-methoxyphenyl)-1,3-dihydroisobenzofuran-5-amine (**7j**).

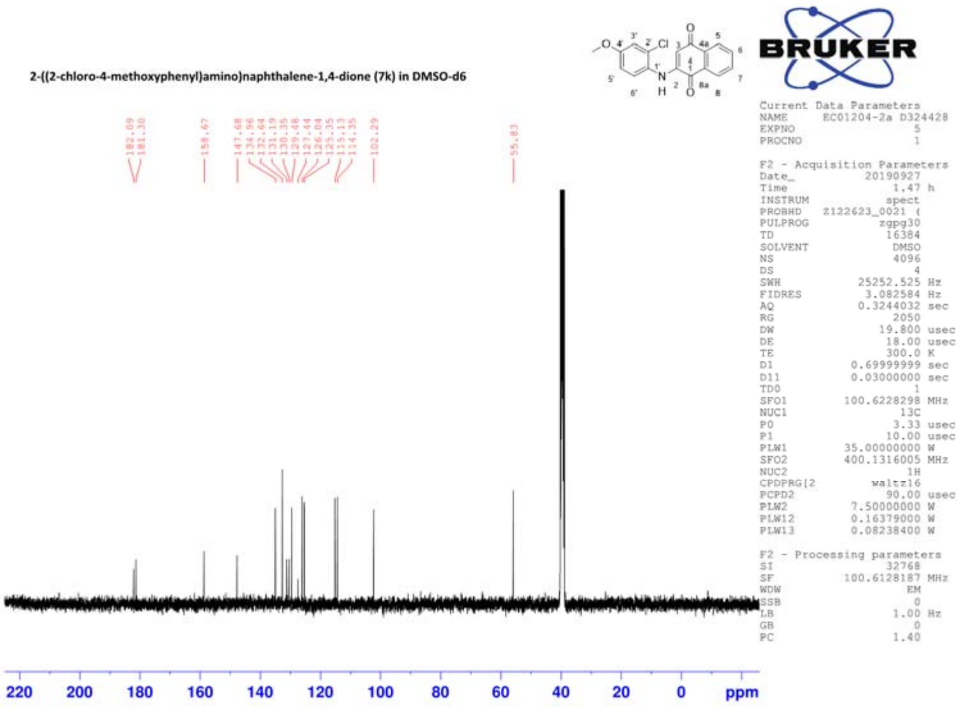

Figure S51. $^{13}C\{^1H\}$ NMR spectrum of 2-((2-chloro-4-methoxyphenyl)amino)naphthalene-1,4-dione (**7k**).

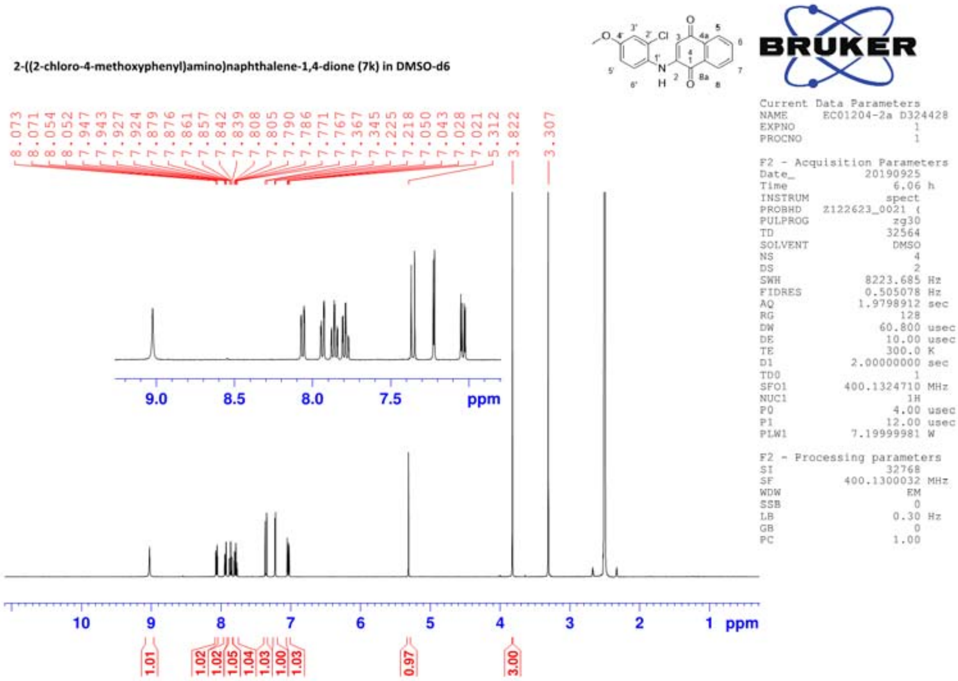

Figure S52. ^{1}H NMR spectrum of 2-((2-chloro-4-methoxyphenyl)amino)naphthalene-1,4-dione (**7k**).

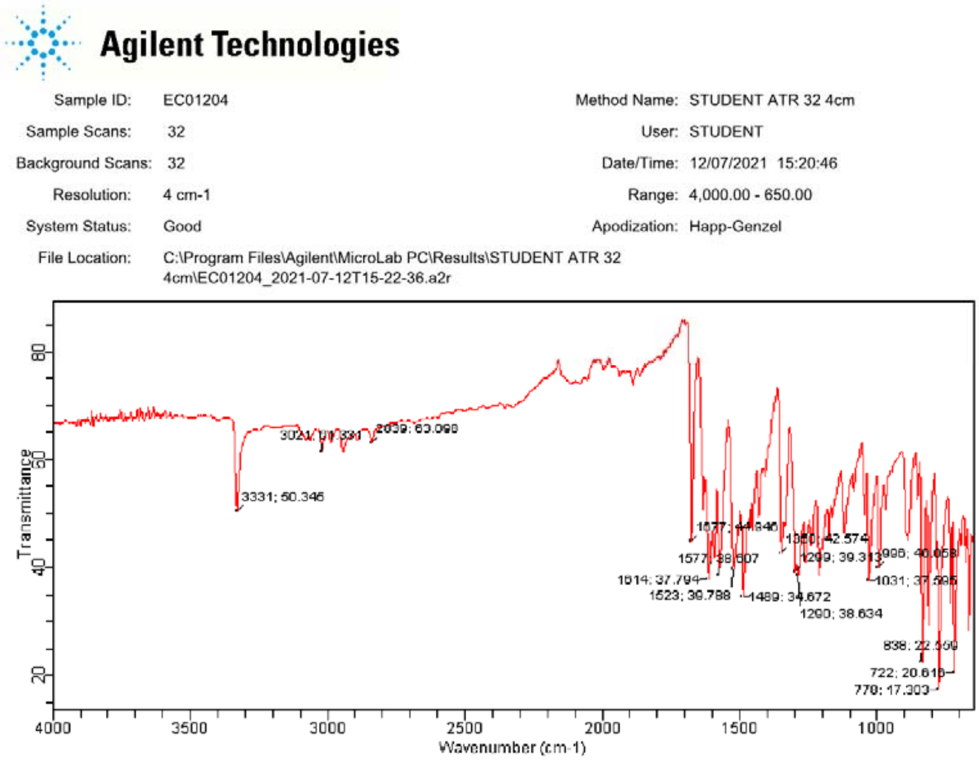

Figure S53. FT-IR spectrum of 2-((2-chloro-4-methoxyphenyl)amino)naphthalene-1,4-dione (**7k**).

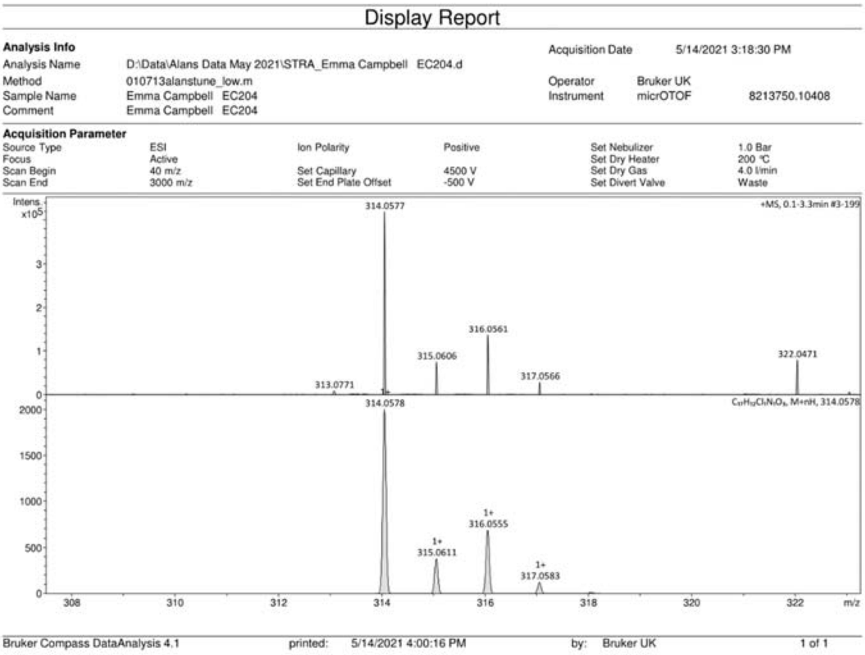

Figure S54. HRMS analysis of 2-((2-chloro-4-methoxyphenyl)amino)naphthalene-1,4-dione (**7k**).

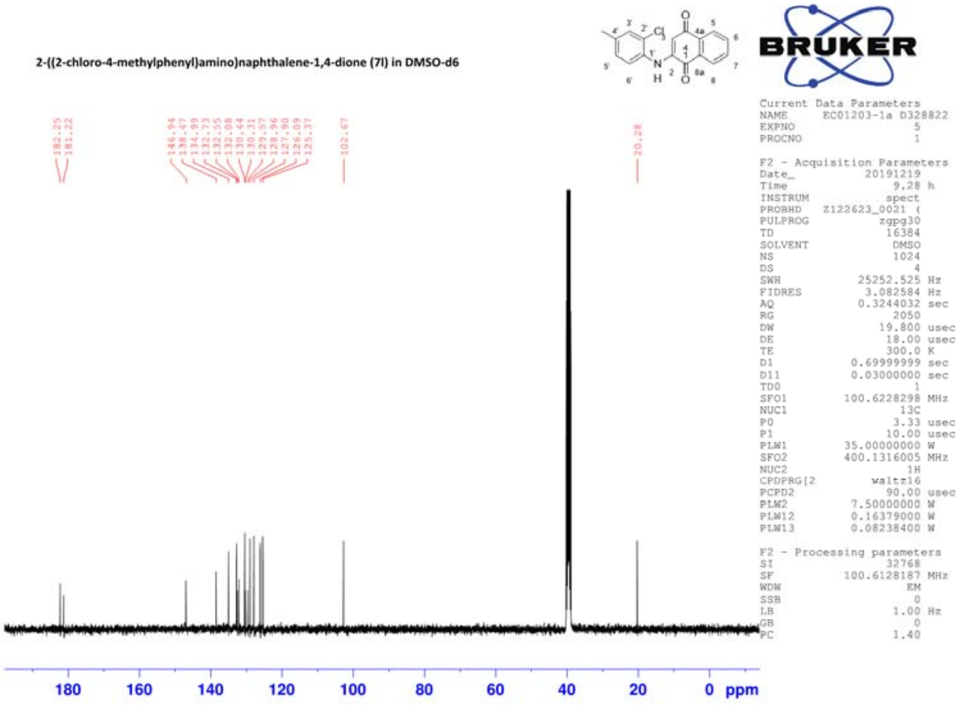

Figure S55. $^{13}C\{^{1}H\}$ NMR spectrum of 2-((2-chloro-4-methylphenyl)amino)naphthalene-1,4-dione (**7l**).

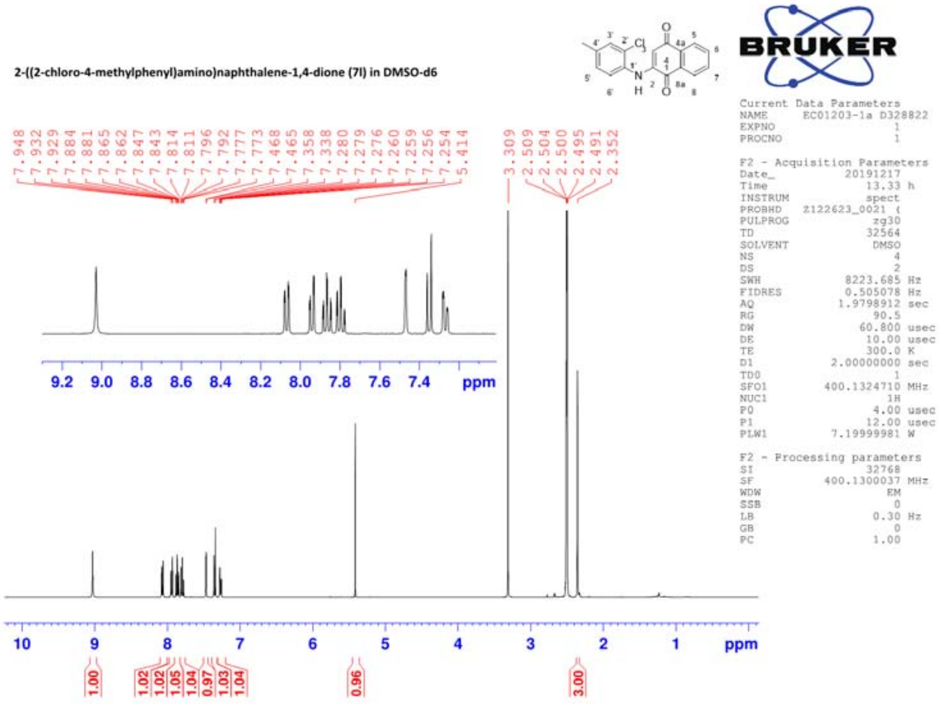

Figure S56. ^{1}H NMR spectrum of 2-((2-chloro-4-methylphenyl)amino)naphthalene-1,4-dione (**7l**).

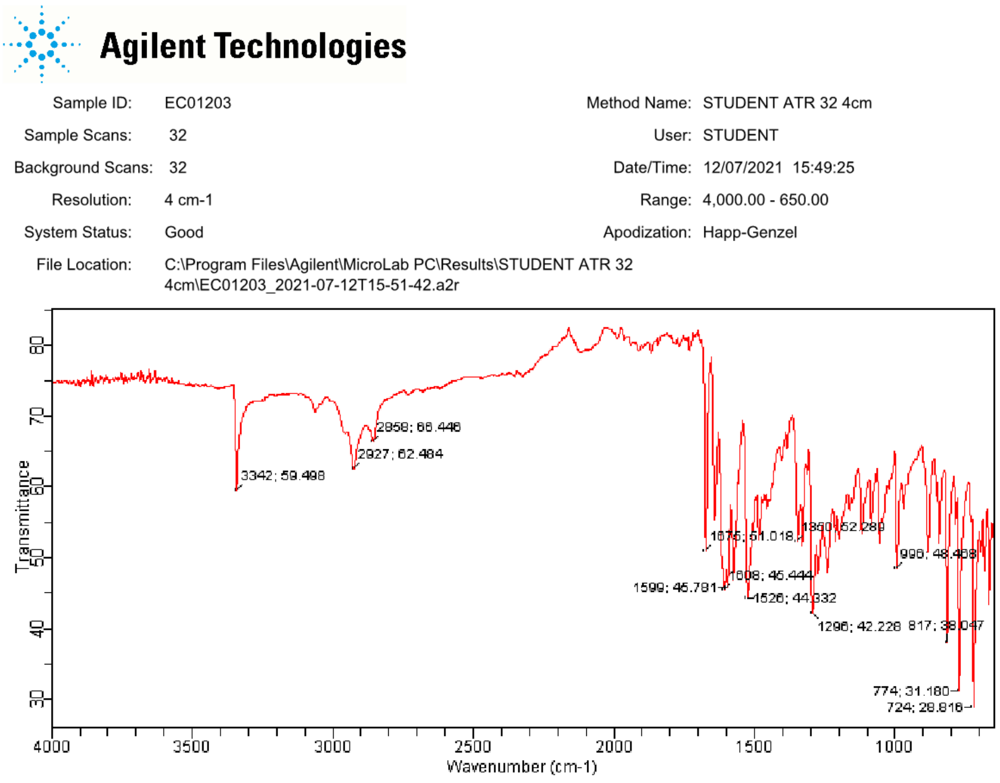

Figure S57. FT-IR spectrum of 2-((2-chloro-4-methylphenyl)amino)naphthalene-1,4-dione (**7l**).

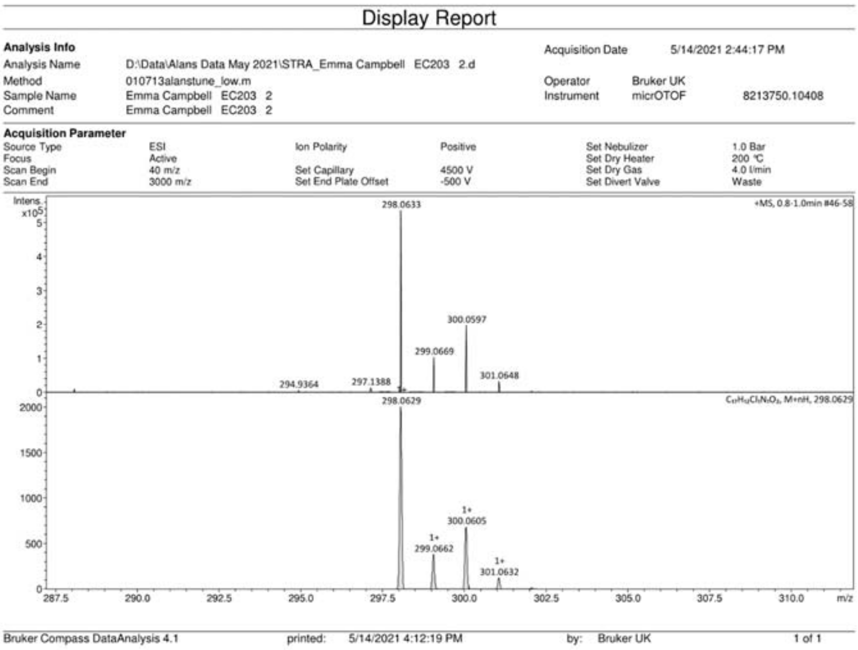

Figure S58. HRMS analysis of 2-((2-chloro-4-methylphenyl)amino)naphthalene-1,4-dione (**7l**).

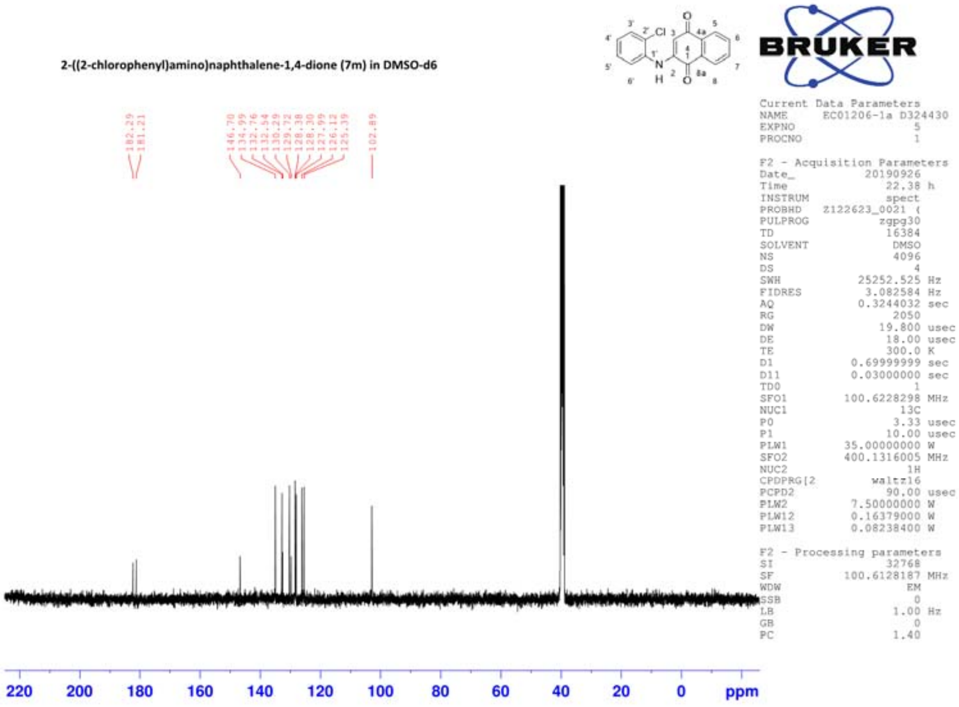

Figure S59. $^{13}C\{^{1}H\}$ NMR spectrum of 2-((2-chlorophenyl)amino)naphthalene-1,4-dione (**7m**).

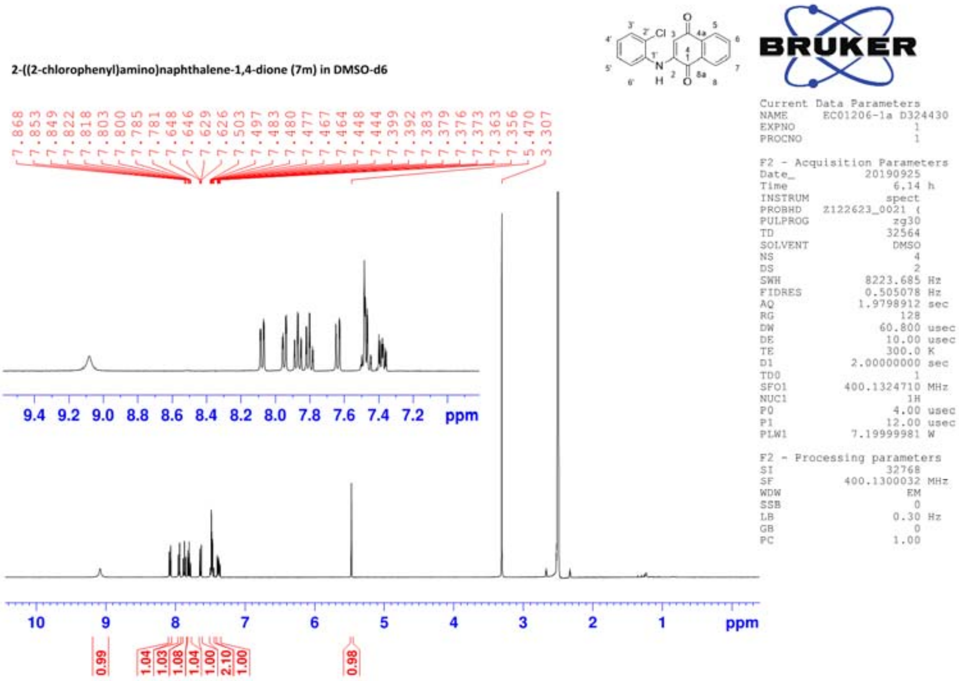

Figure S60. ^{1}H NMR spectrum of2-((2-chlorophenyl)amino)naphthalene-1,4-dione (**7m**).

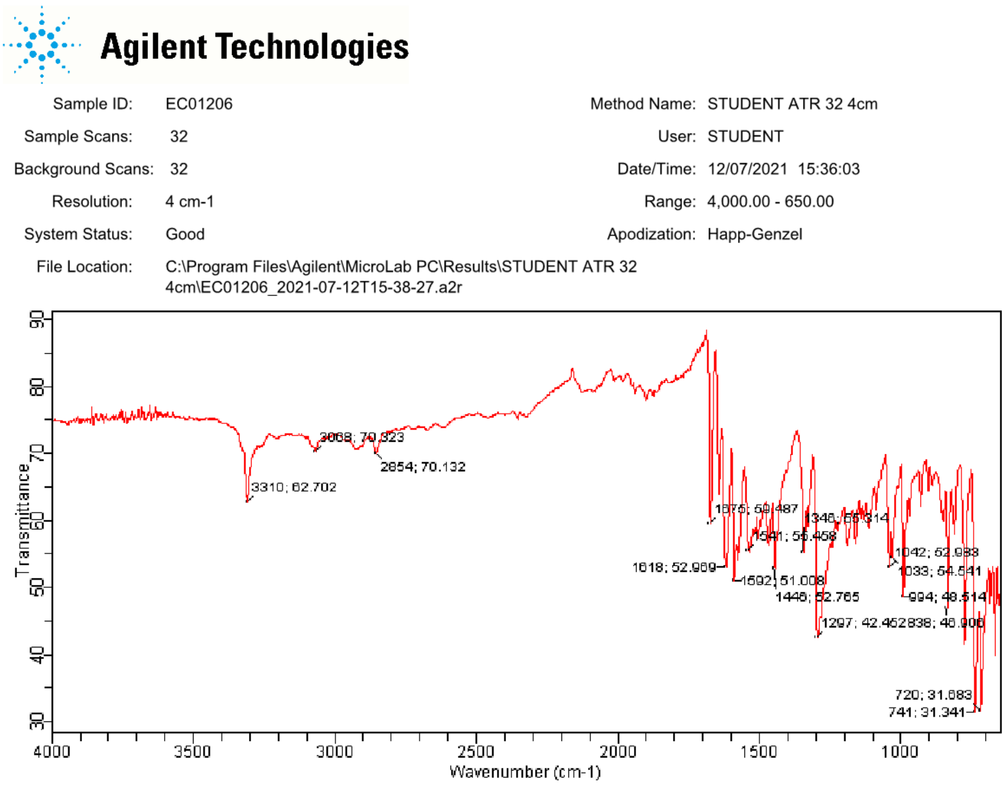

Figure S61. FT-IR spectrum of 2-((2-chlorophenyl)amino)naphthalene-1,4-dione (**7m**).

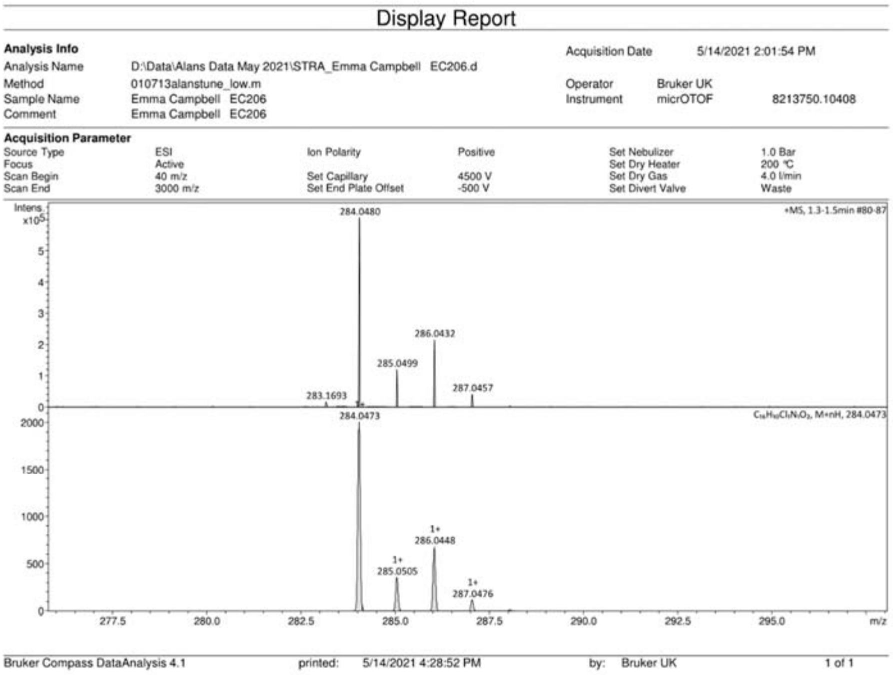

Figure S62. HRMS analysis of 2-((2-chlorophenyl)amino)naphthalene-1,4-dione (**7m**).

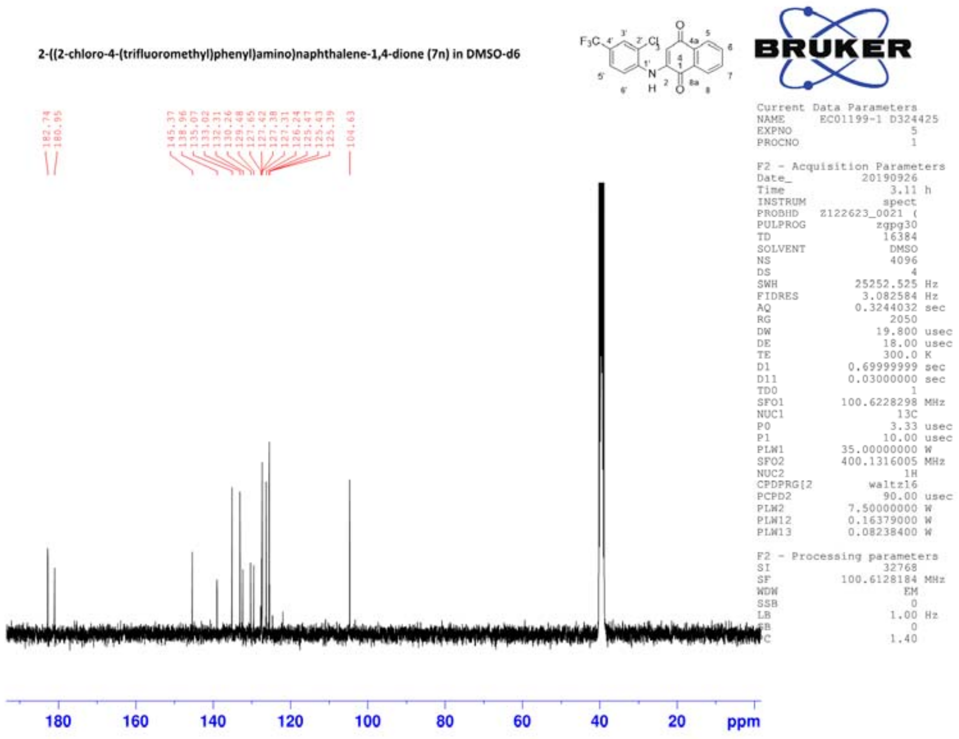

Figure S63. $^{13}C\{^1H\}$ NMR spectrum of 2-((2-chloro-4-(trifluoromethyl)phenyl)amino)naphthalene-1,4-dione (**7n**).

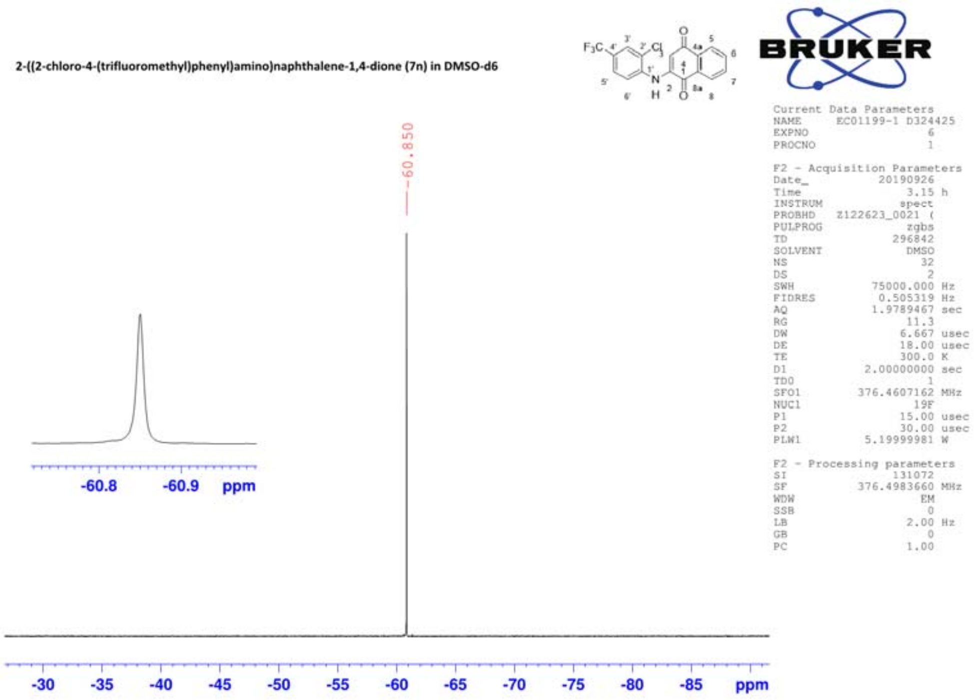

Figure S64. ^{19}F NMR spectrum of 2-((2-chloro-4-(trifluoromethyl)phenyl)amino)naphthalene-1,4-dione (**7n**).

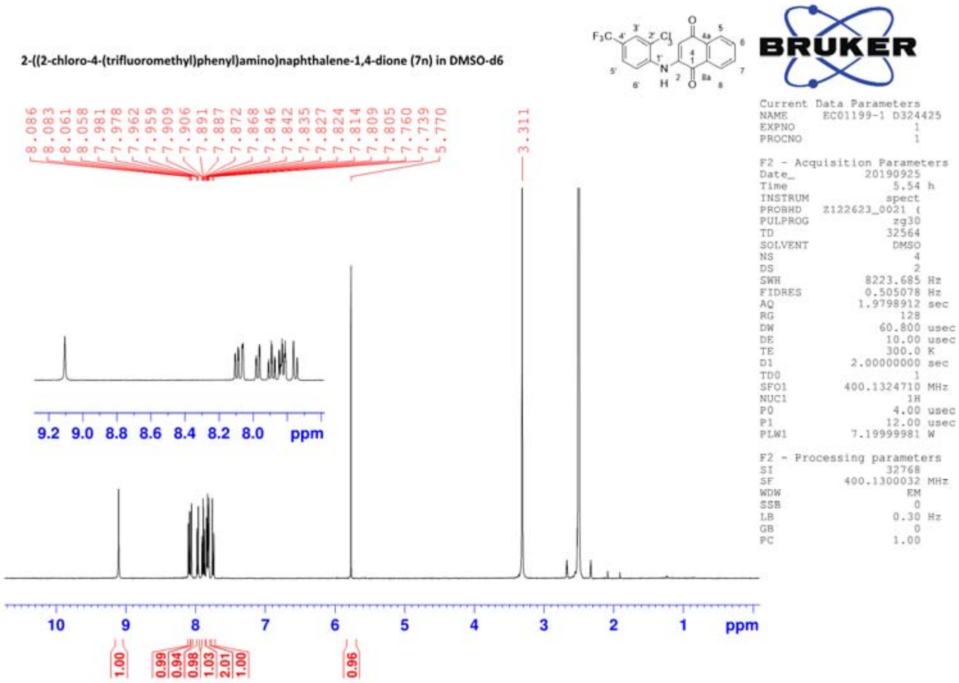

Figure S65. ^{1}H NMR spectrum of 2-((2-chloro-4-(trifluoromethyl)phenyl)amino)naphthalene-1,4-dione (**7n**).

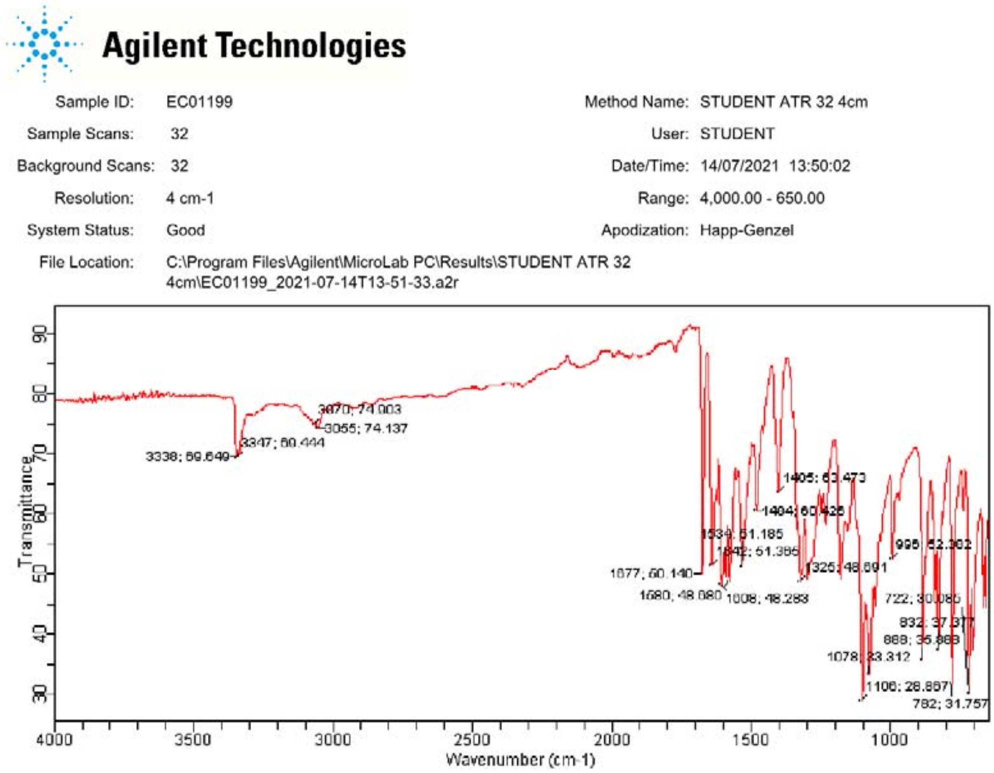

Figure S66. FT-IR spectrum of 2-((2-chloro-4-(trifluoromethyl)phenyl)amino)naphthalene-1,4-dione

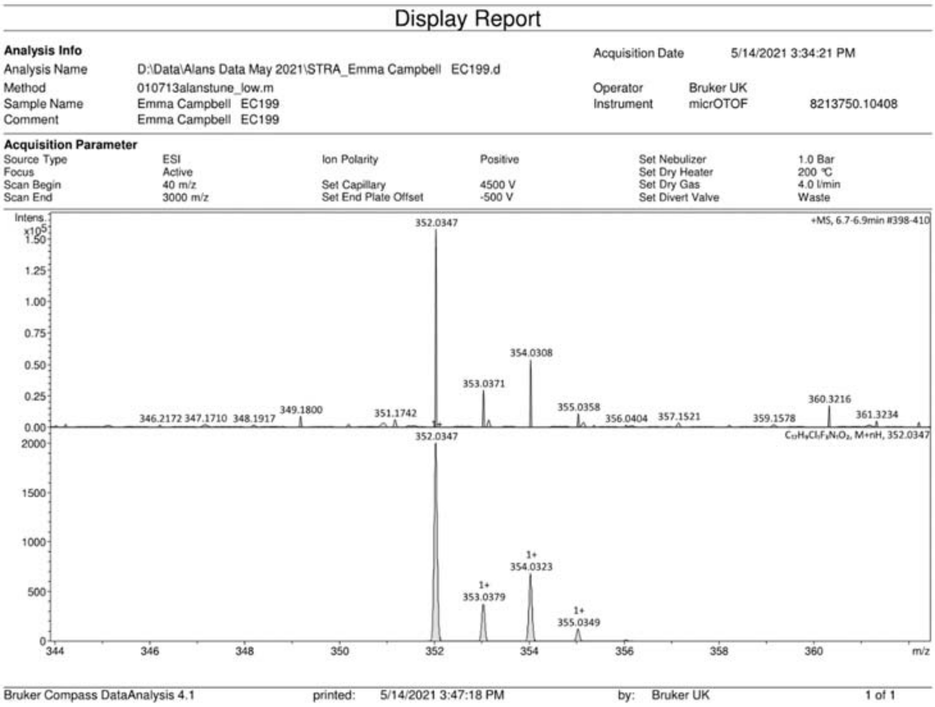

(**7n**).

Figure S67. HRMS analysis of 2-((2-chloro-4-(trifluoromethyl)phenyl)amino)naphthalene-1,4-dione (**7n**).

(**7o**).

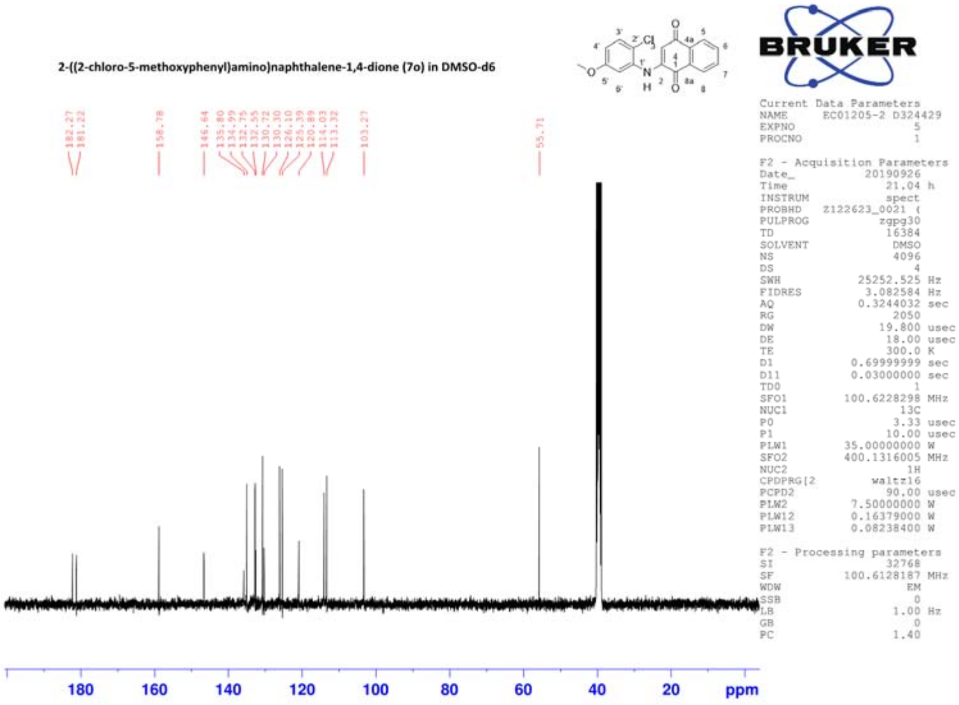

Figure S68. $^{13}C\{^1H\}$ NMR spectrum of 2-((2-chloro-5-methoxyphenyl)amino)naphthalene-1,4-dione

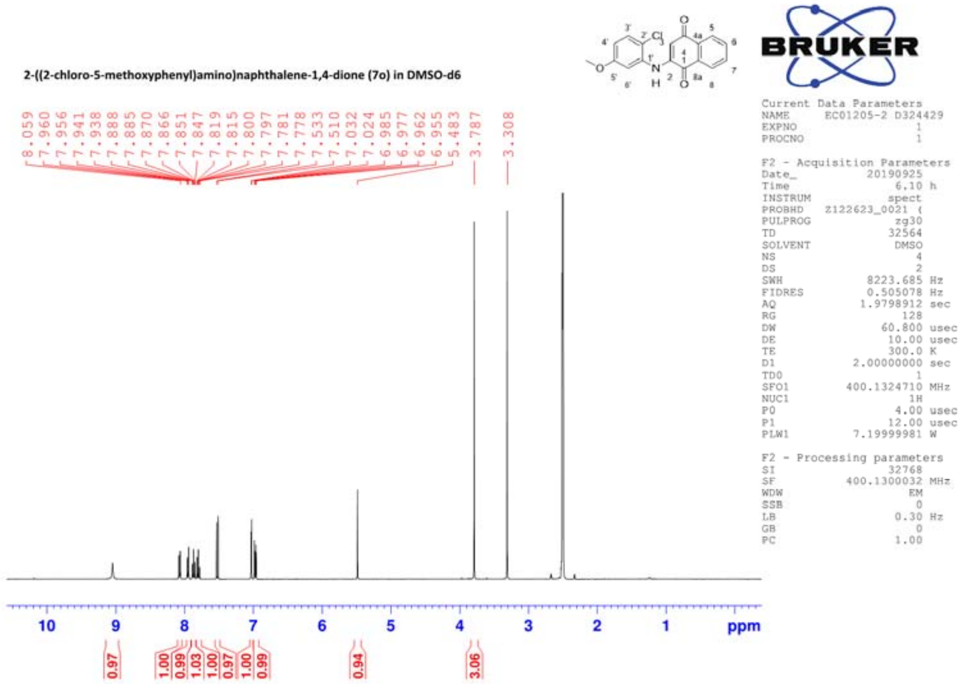

(**7o**).

Figure S69. ^1H NMR spectrum of 2-((2-chloro-5-methoxyphenyl)amino)naphthalene-1,4-dione (**7o**).

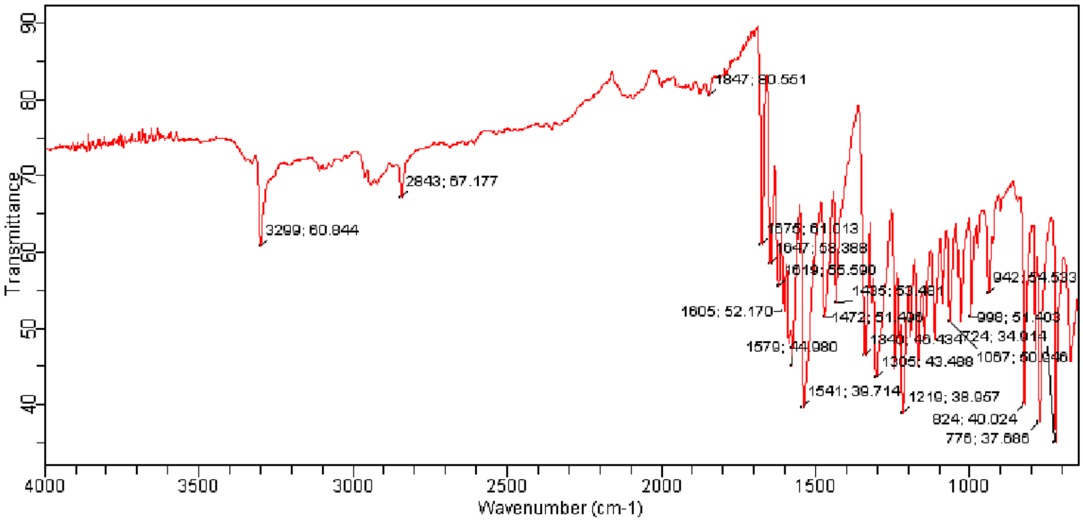

Figure S70. FT-IR spectrum of 2-((2-chloro-5-methoxyphenyl)amino)naphthalene-1,4-dione (**7o**)

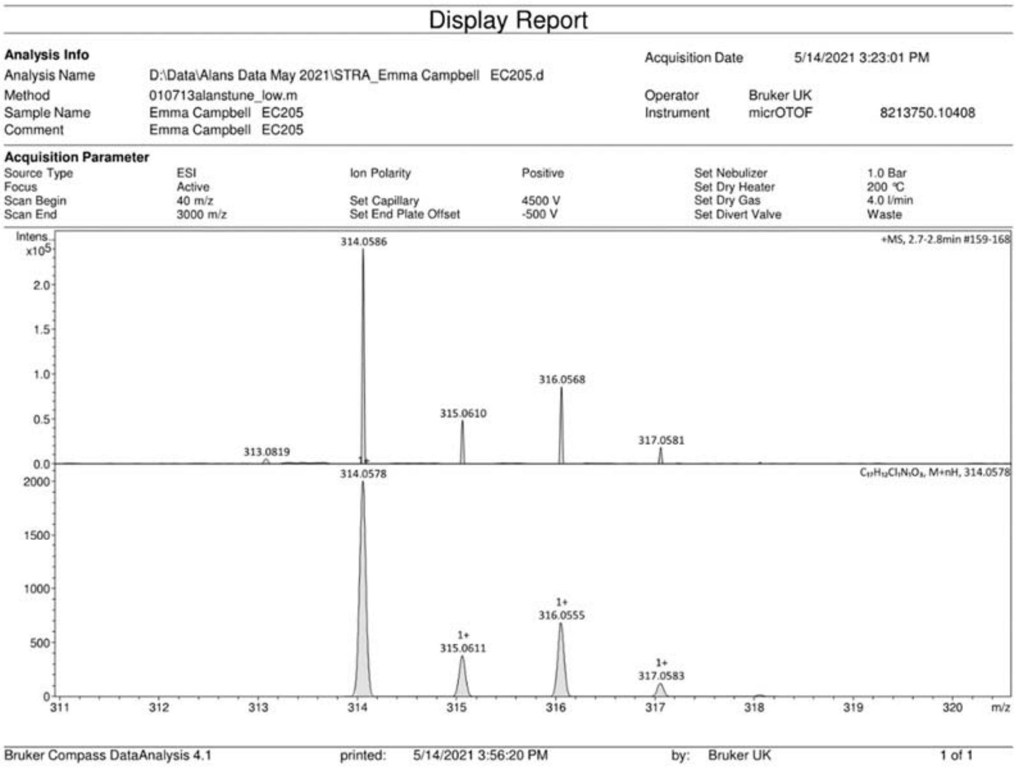

Figure S71. HRMS analysis of 2-((2-chloro-5-methoxyphenyl)amino)naphthalene-1,4-dione (**7o**).

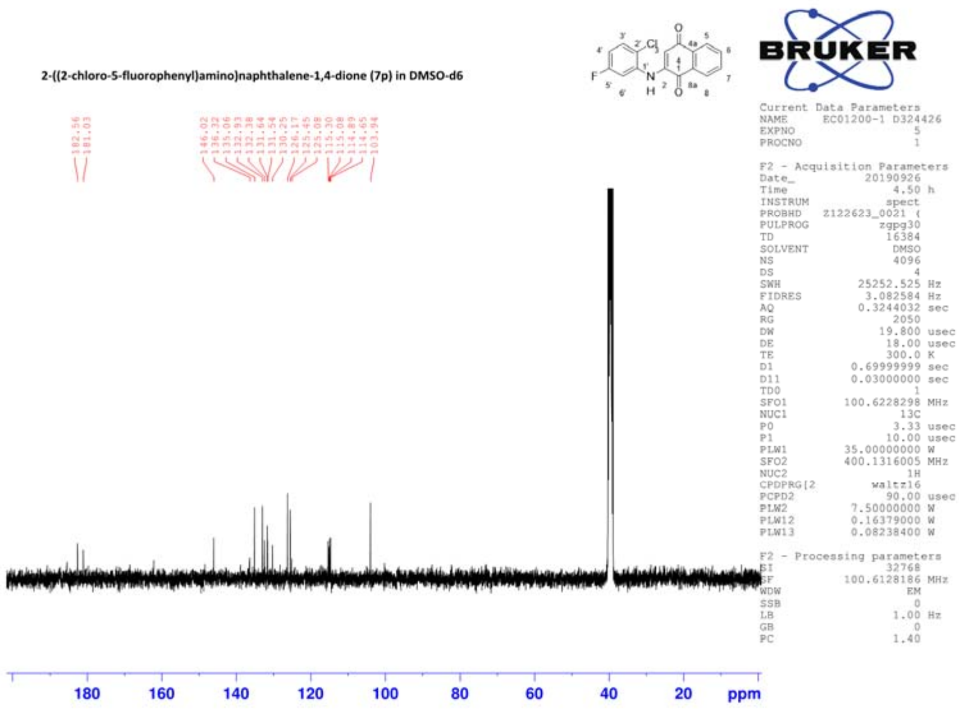

Figure S72. $^{13}C\{^1H\}$ NMR spectrum of 2-((2-chloro-5-fluorophenyl)amino)naphthalene-1,4-dione (**7p**).

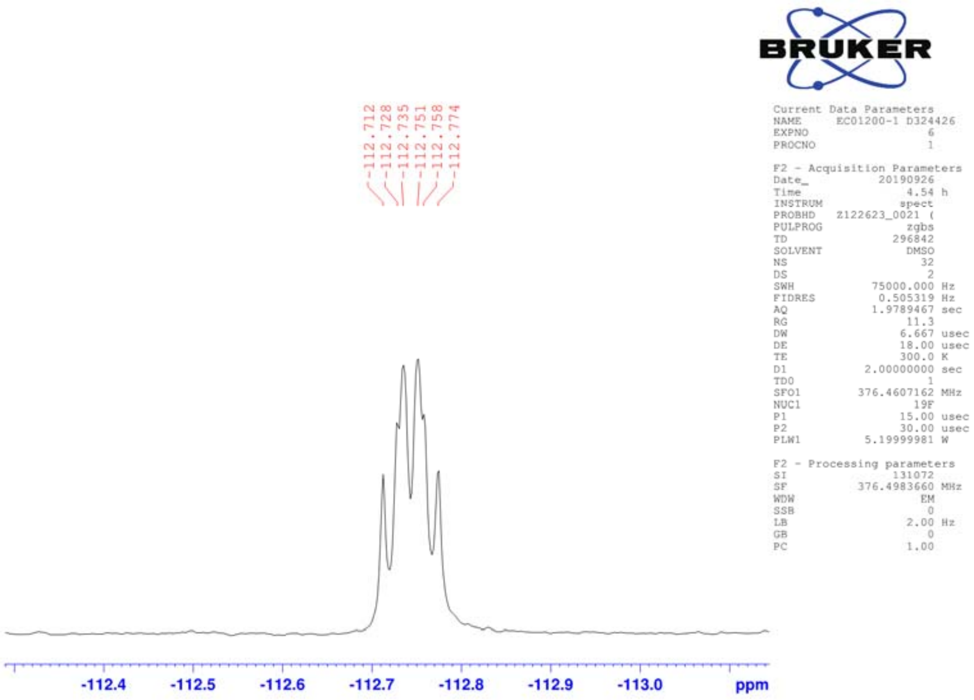

Figure S73. ^{19}F NMR spectrum of 2-((2-chloro-5-fluorophenyl)amino)naphthalene-1,4-dione (**7p**).

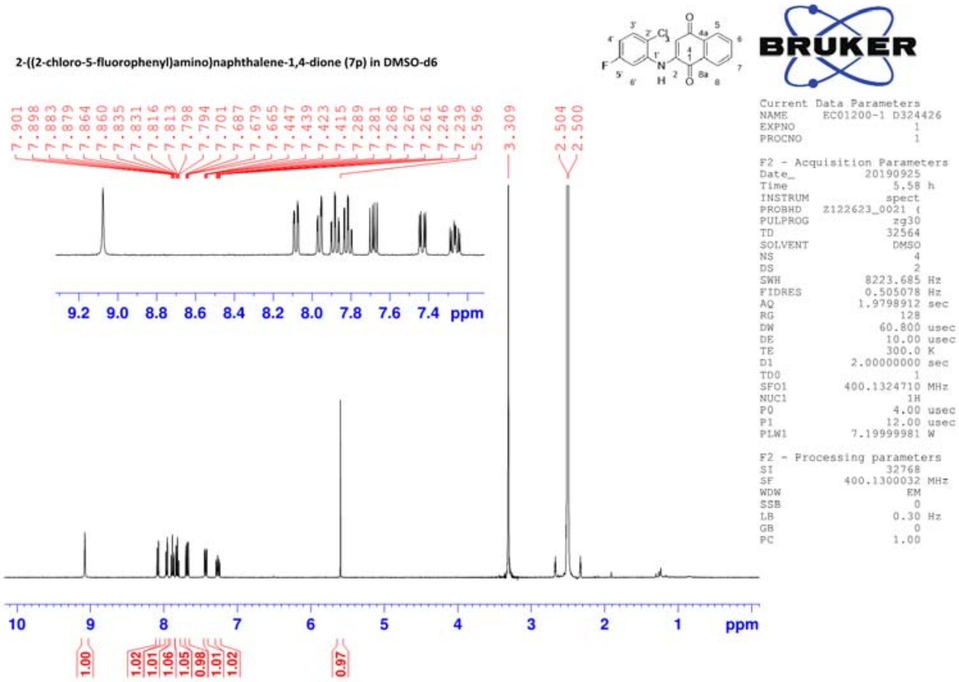

Figure S74. ^{1}H NMR spectrum of 2-((2-chloro-5-fluorophenyl)amino)naphthalene-1,4-dione (**7p**).

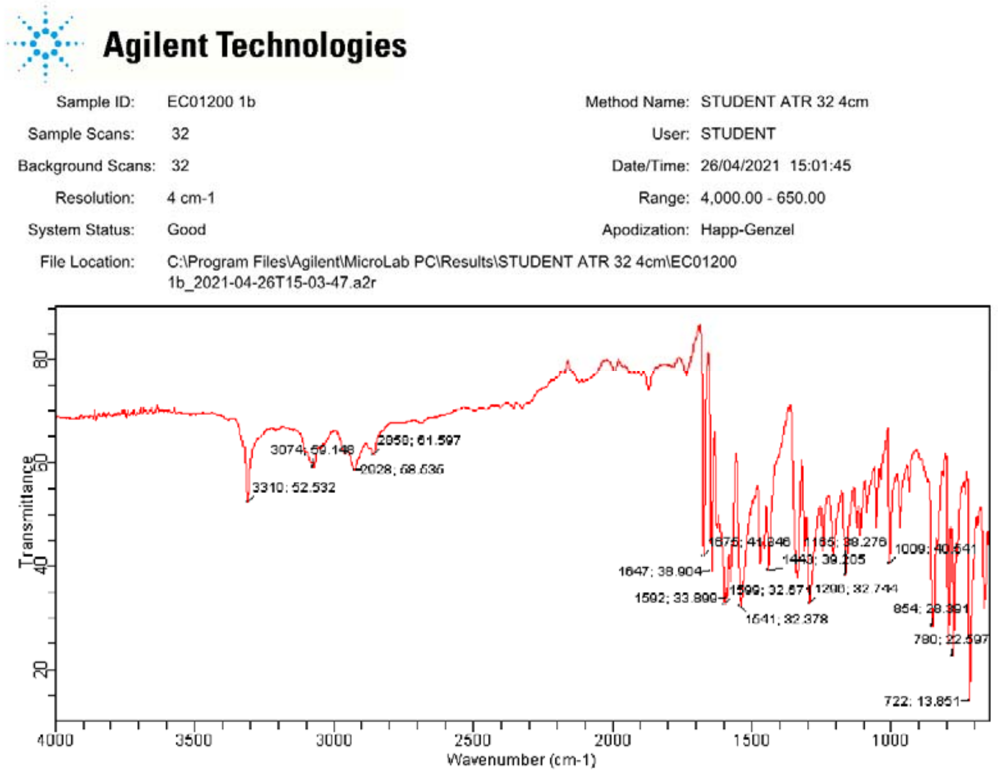

Figure S75. FT-IR spectrum of 2-((2-chloro-5-fluorophenyl)amino)naphthalene-1,4-dione (**7p**).

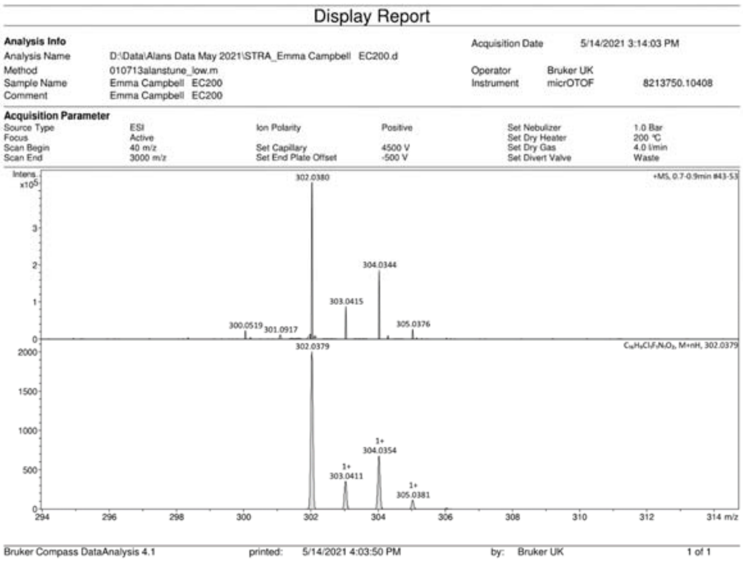

Figure S76. HRMS analysis of 2-((2-chloro-5-fluorophenyl)amino)naphthalene-1,4-dione (**7p**).

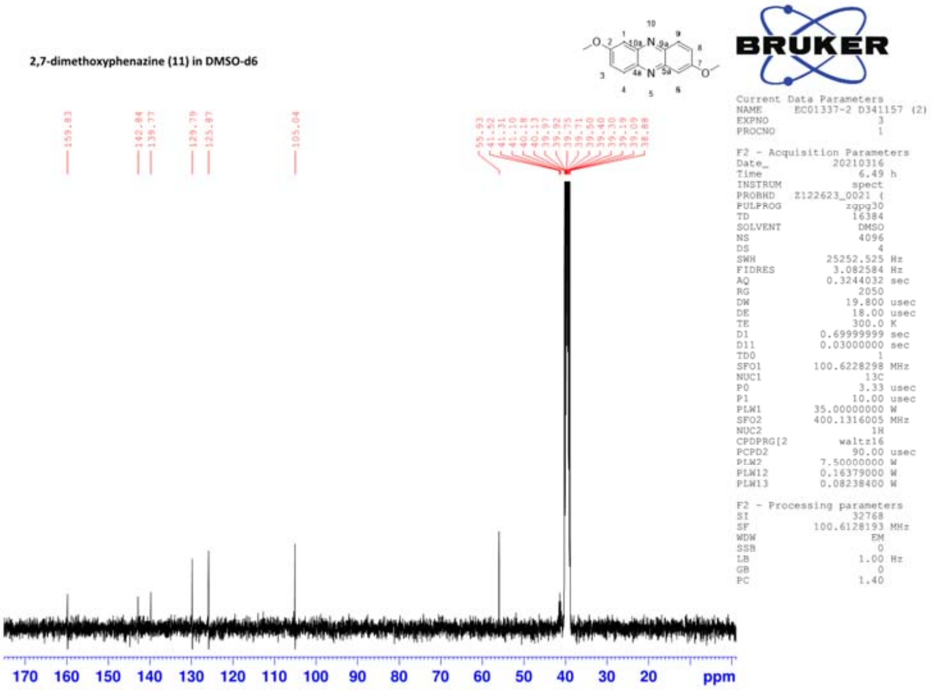

Figure S77. $^{13}C\{^{1}H\}$ NMR spectrum of 2,7-dimethoxyphenazine (**11**).

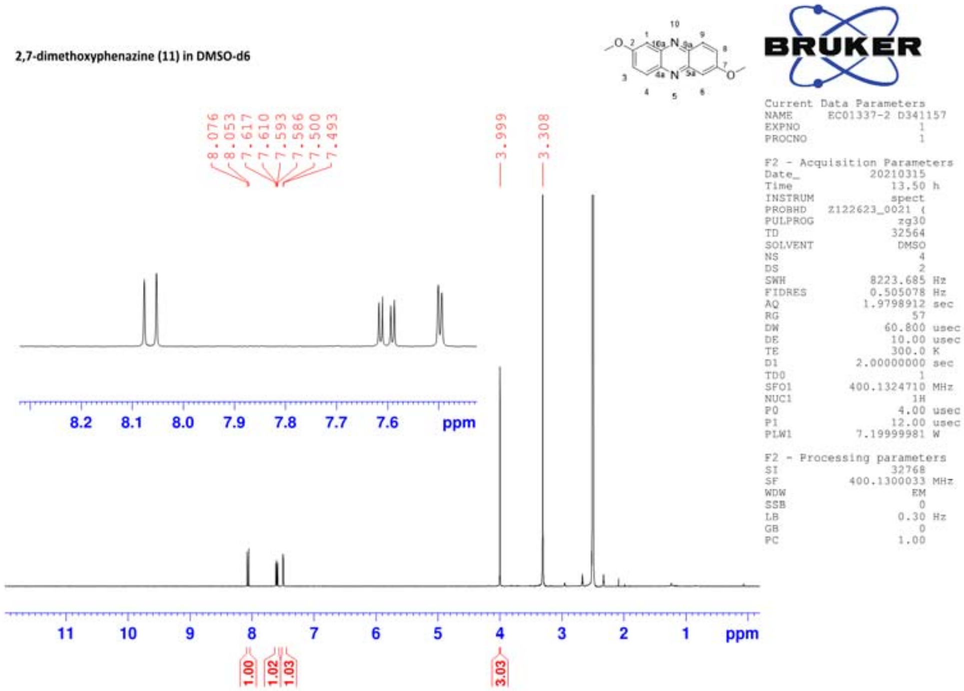

Figure S78. ^{1}H NMR spectrum of 2,7-dimethoxyphenazine (**11**).

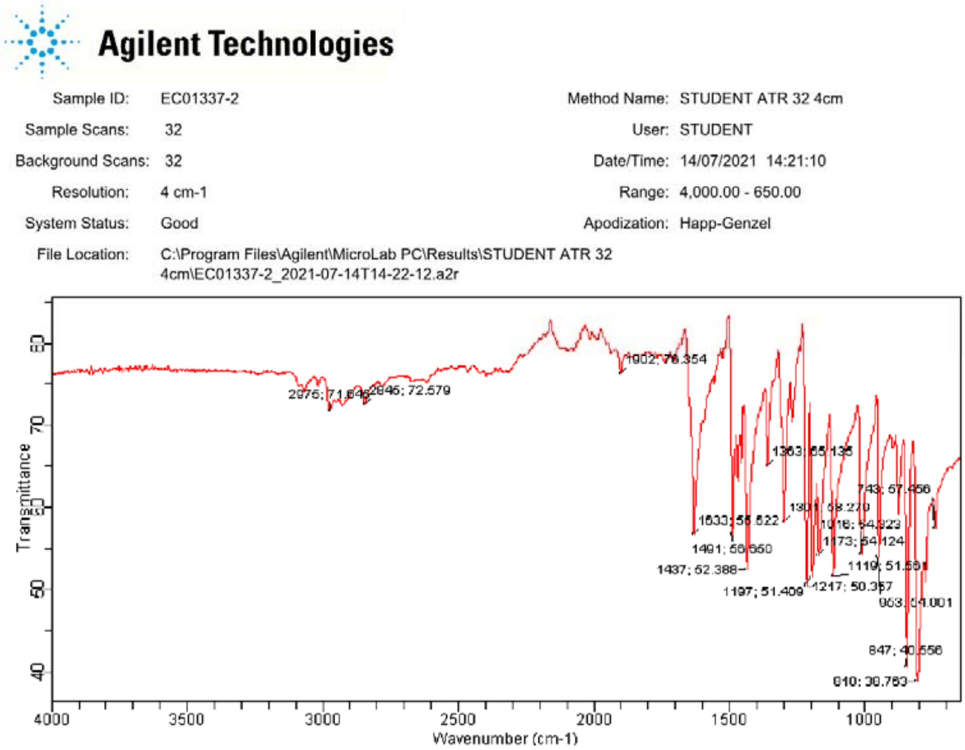

Figure S79. FT-IR spectrum of 2,7-dimethoxyphenazine (**11**).

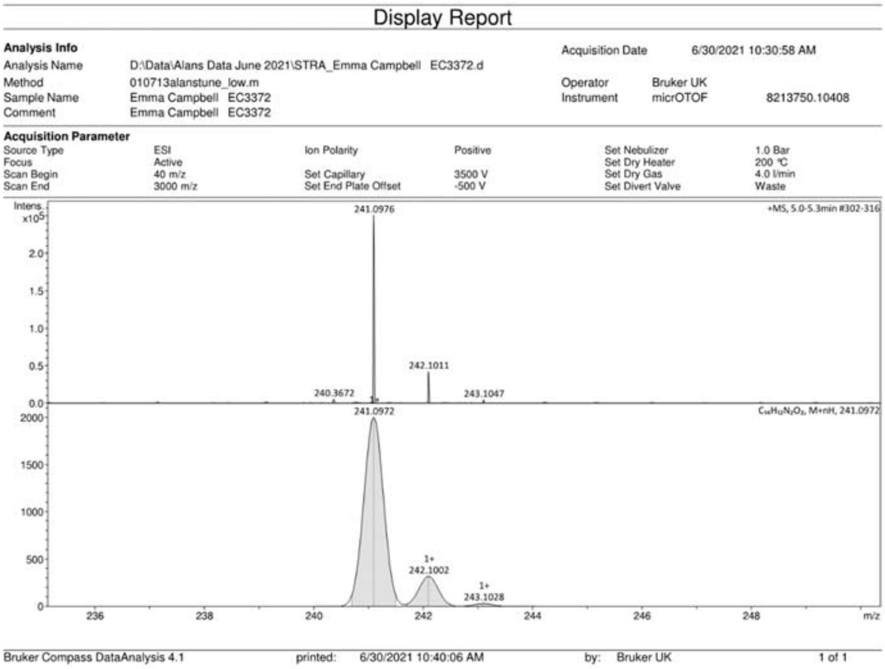

Figure S80. HRMS analysis of 2,7-dimethoxyphenazine (**11**).

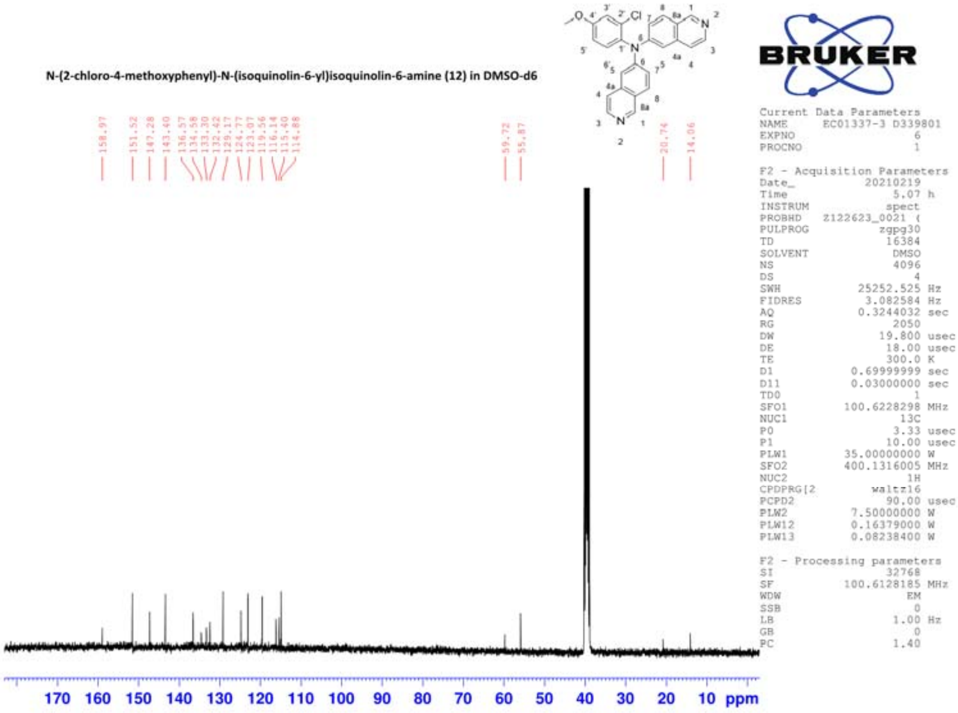

Figure S81. $^{13}C\{^{1}H\}$ NMR spectrum of N-(2-chloro-4-methoxyphenyl)-N-(isoquinolin-6-yl)isoquinolin-6-amine (**12**).

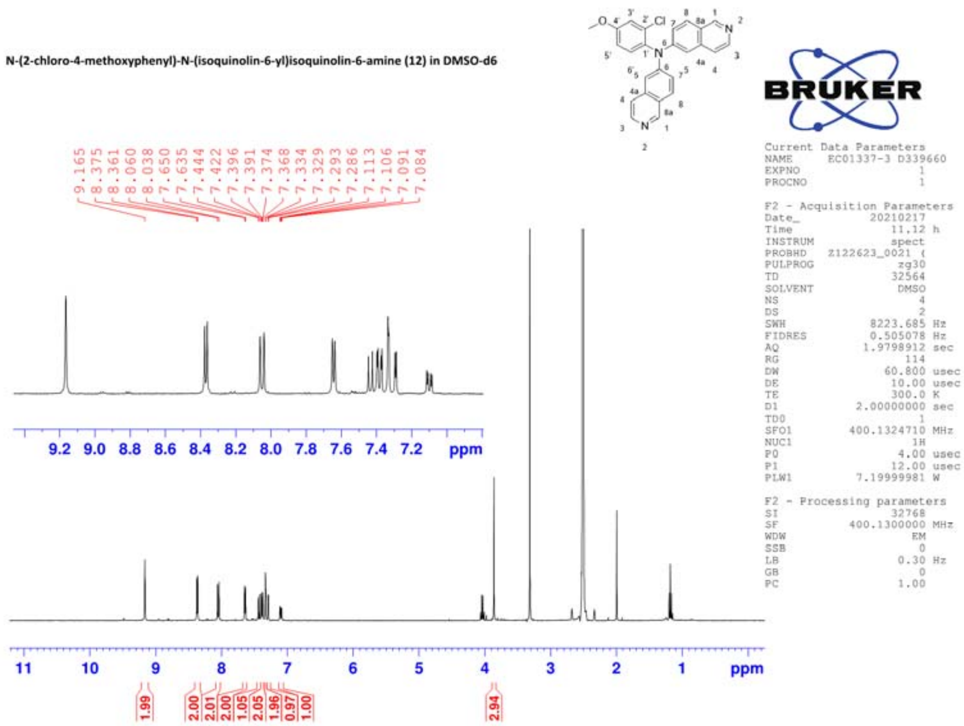

Figure S82. ^{1}H NMR spectrum of N-(2-chloro-4-methoxyphenyl)-N-(isoquinolin-6-yl)isoquinolin-6-amine (**12**).

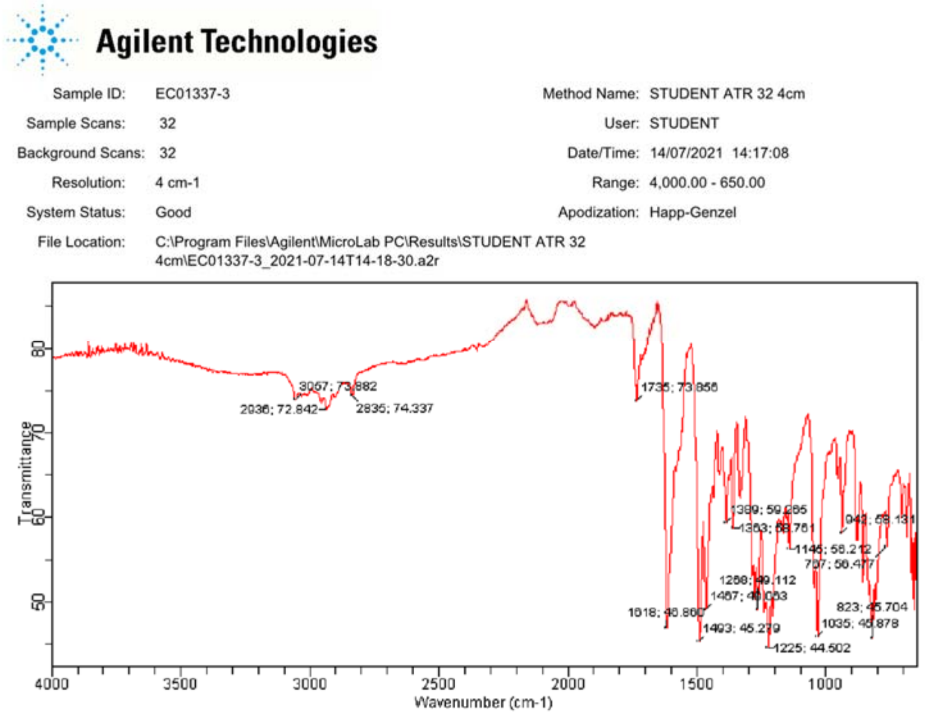

Figure S83. FT-IR spectrum of N-(2-chloro-4-methoxyphenyl)-N-(isoquinolin-6-yl)isoquinolin-6-amine (**12**).

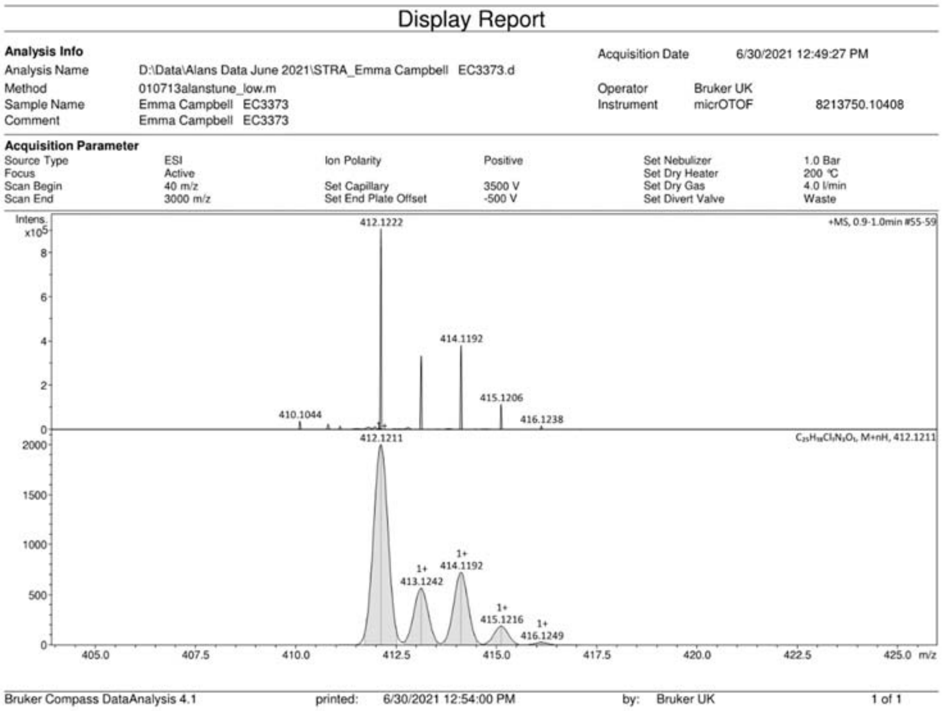

Figure S84. HRMS analysis of N-(2-chloro-4-methoxyphenyl)-N-(isoquinolin-6-yl)isoquinolin-6-amine (**12**).

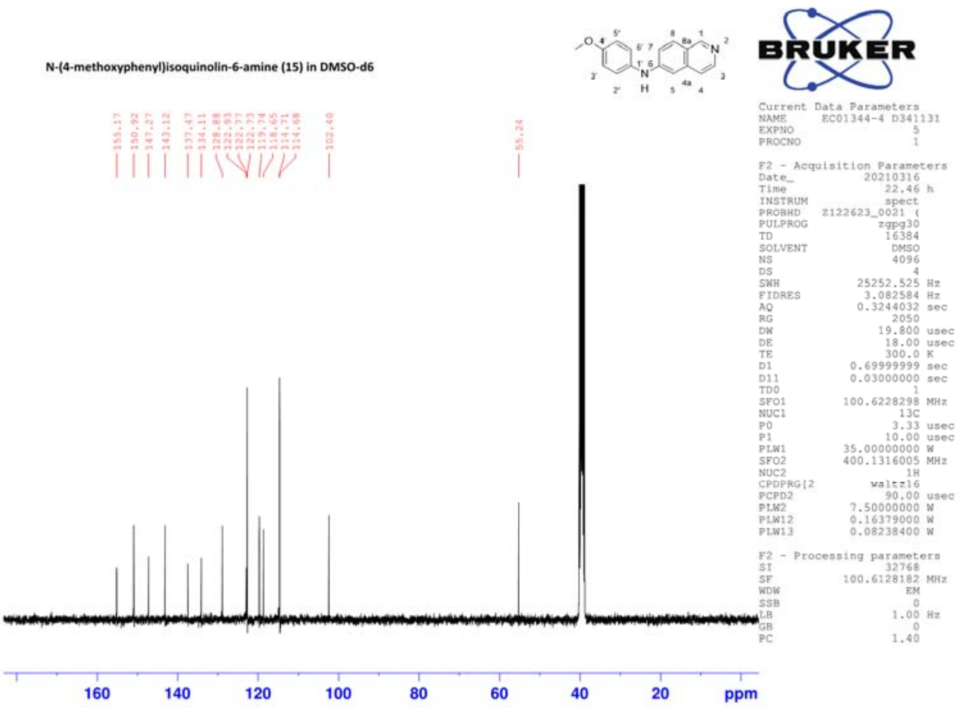

Figure S85. $^{13}C\{^{1}H\}$ NMR spectrum of N-(4-methoxyphenyl)isoquinolin-6-amine (**15**).

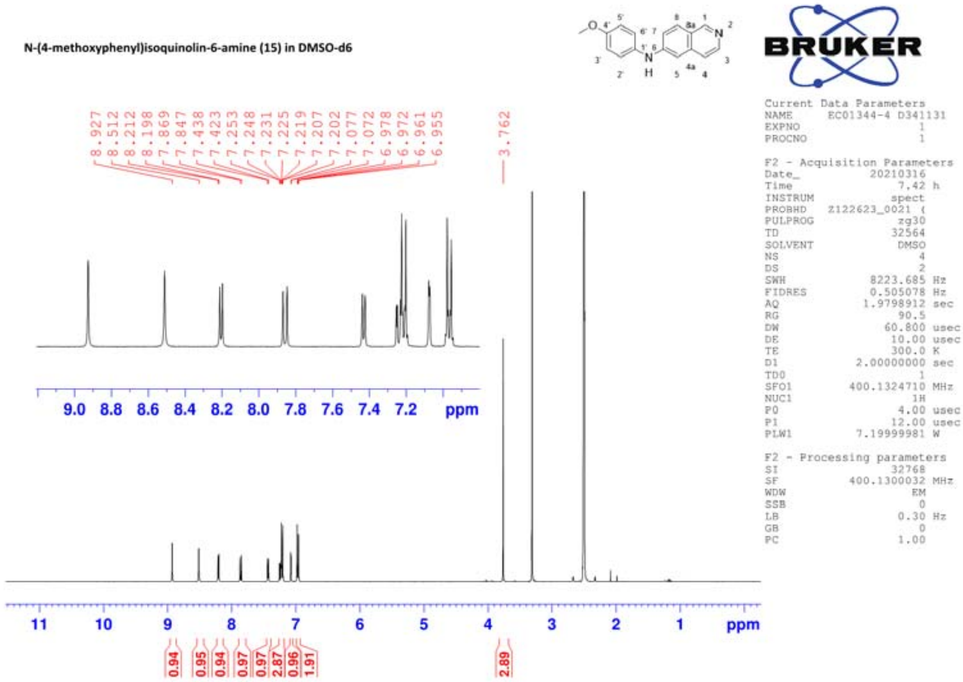

Figure S86. ^{1}H NMR spectrum of N-(4-methoxyphenyl)isoquinolin-6-amine (**15**).

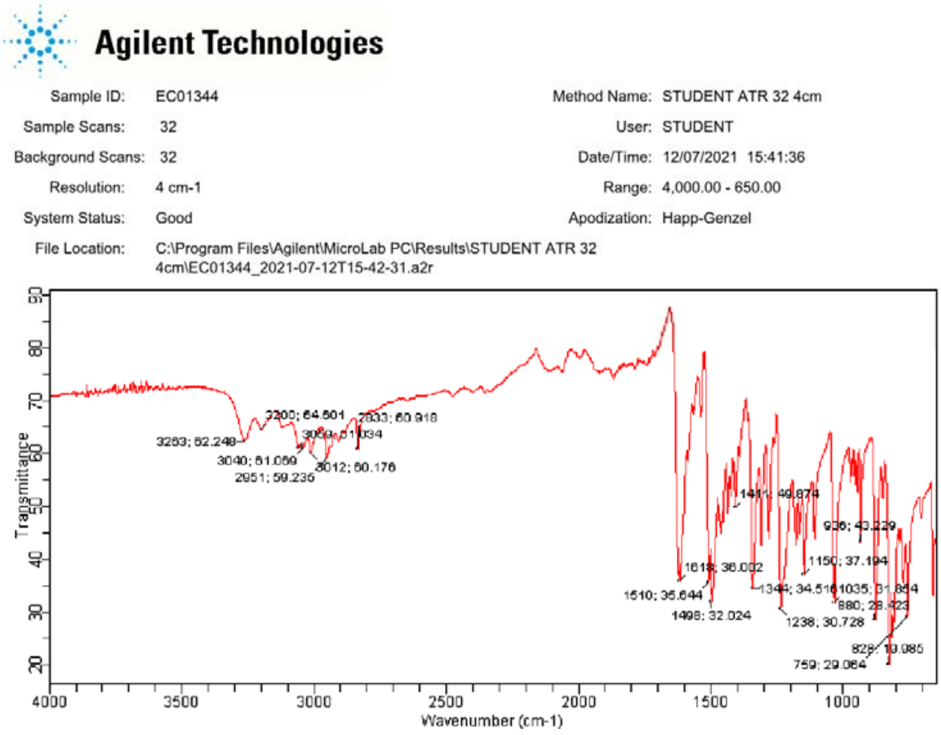

Figure S87. FT-IR spectrum of N-(4-methoxyphenyl)isoquinolin-6-amine (**15**).

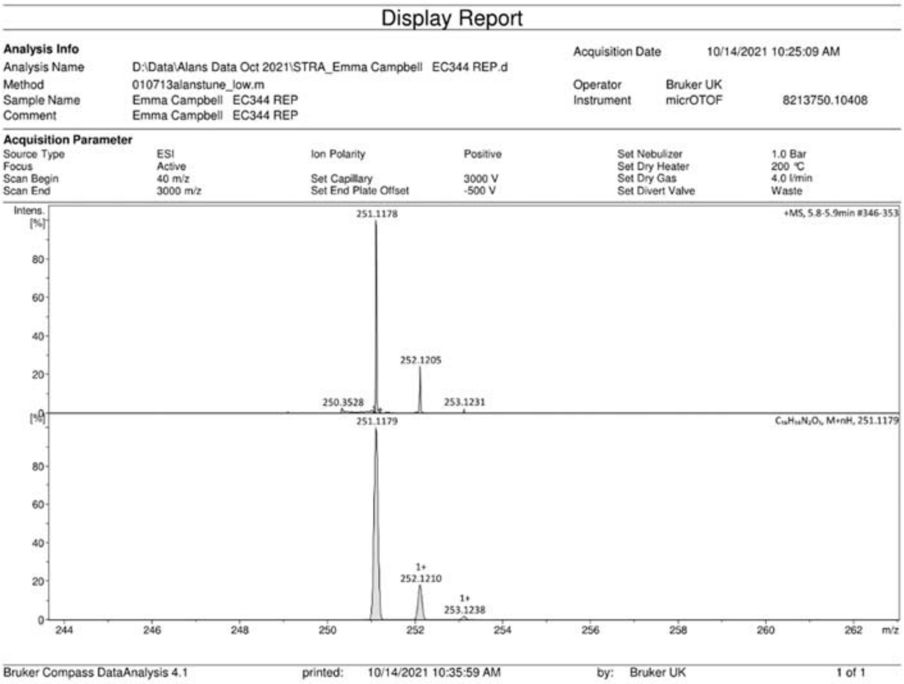

Figure S88. HRMS analysis of N-(4-methoxyphenyl)isoquinolin-6-amine (**15**).

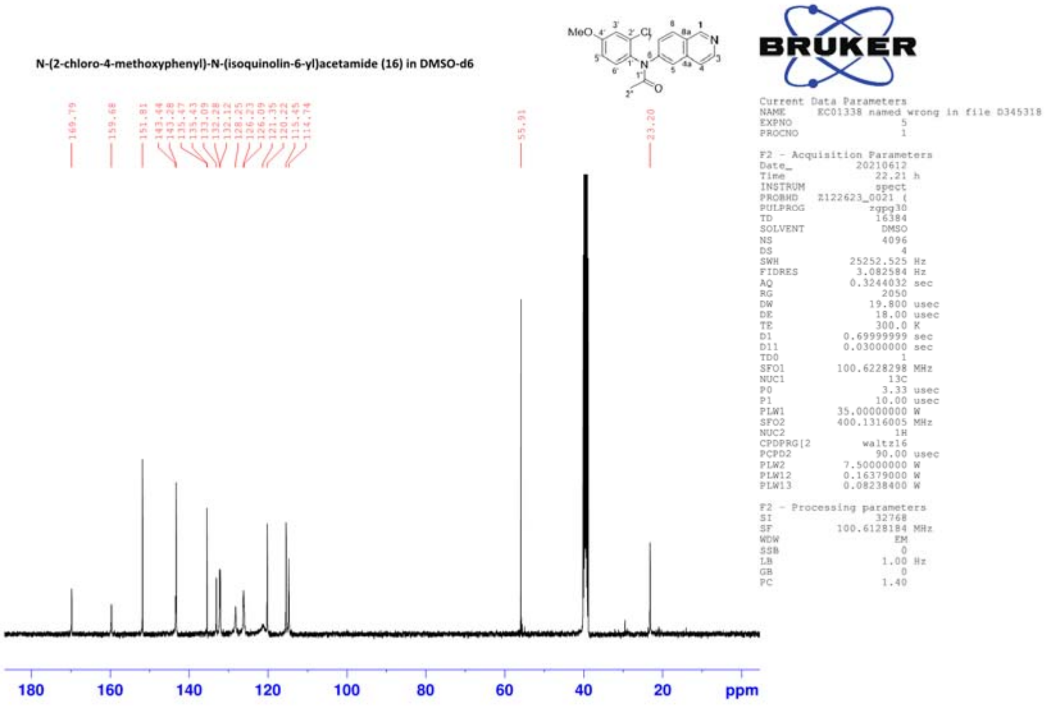

Figure S89. $^{13}C\{^{1}H\}$ NMR spectrum of N-(2-chloro-4-methoxyphenyl)-N-(isoquinolin-6-yl)acetamide (**16**).

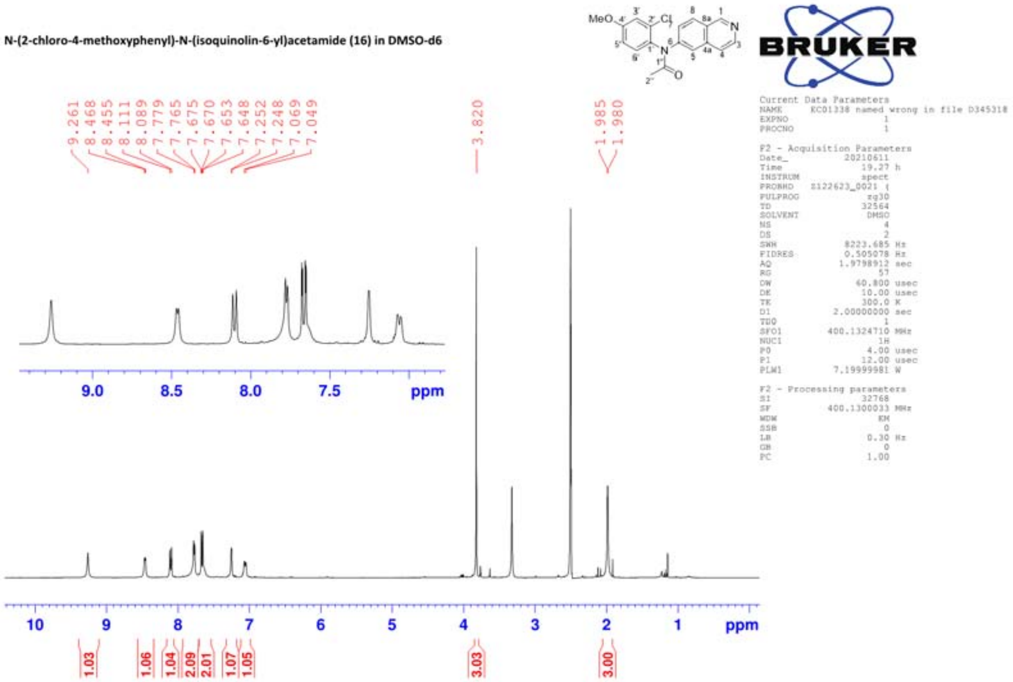

Figure S90. ^{1}H NMR spectrum of N-(2-chloro-4-methoxyphenyl)-N-(isoquinolin-6-yl)acetamide (**16**).

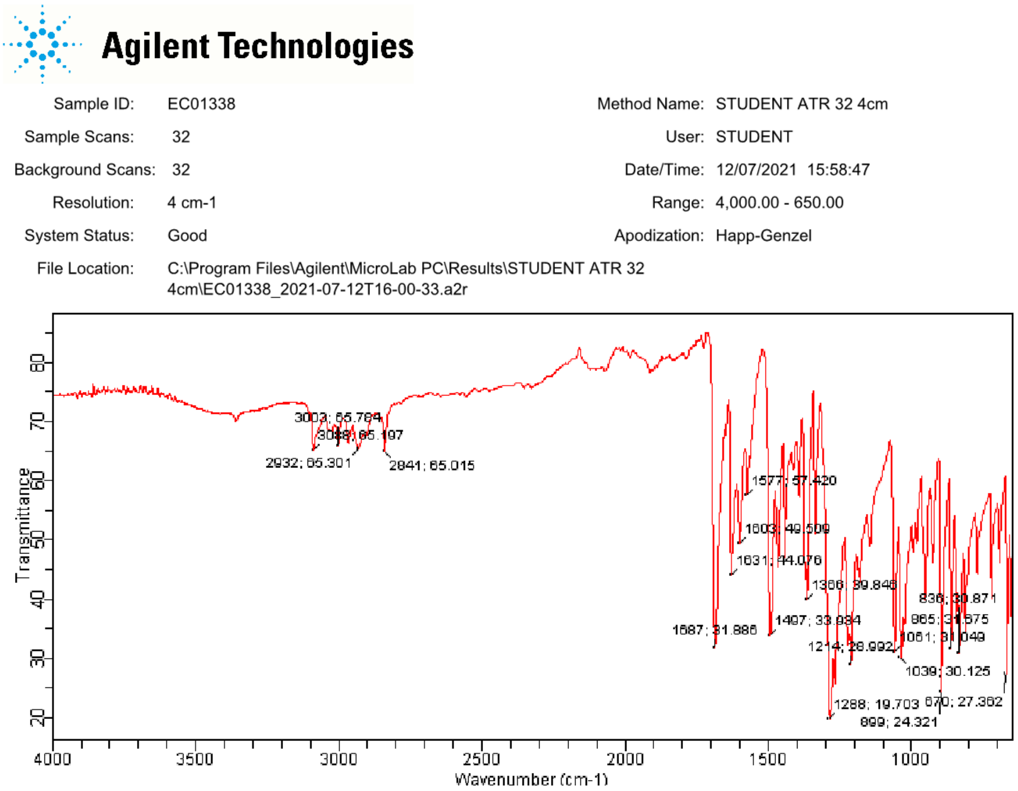

Figure S91. FT-IR spectrum of N-(2-chloro-4-methoxyphenyl)-N-(isoquinolin-6-yl)acetamide (**16**).

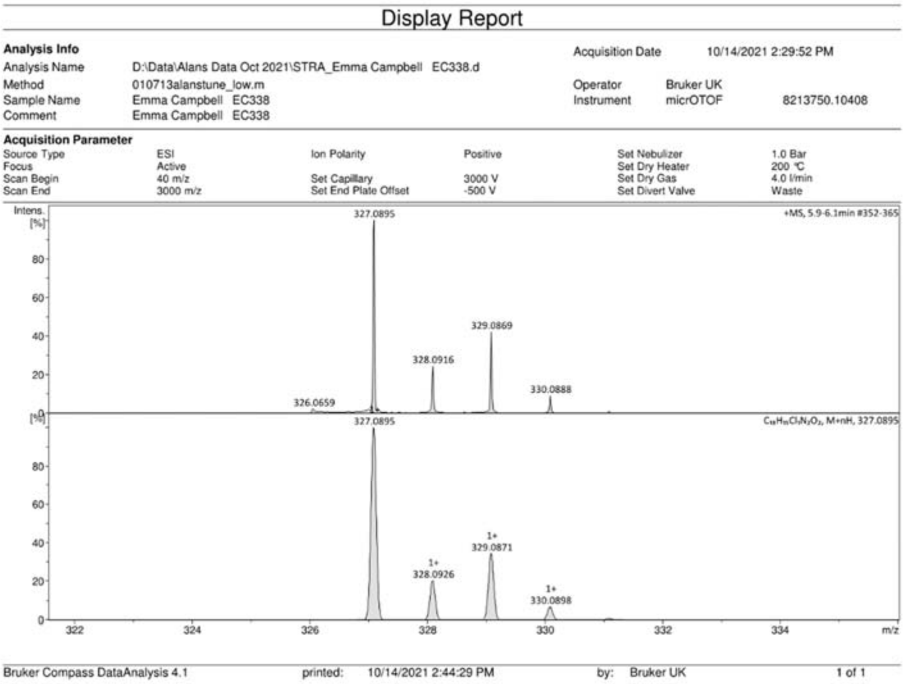

Figure S92. HRMS analysis of N-(2-chloro-4-methoxyphenyl)-N-(isoquinolin-6-yl)acetamide (**16**).

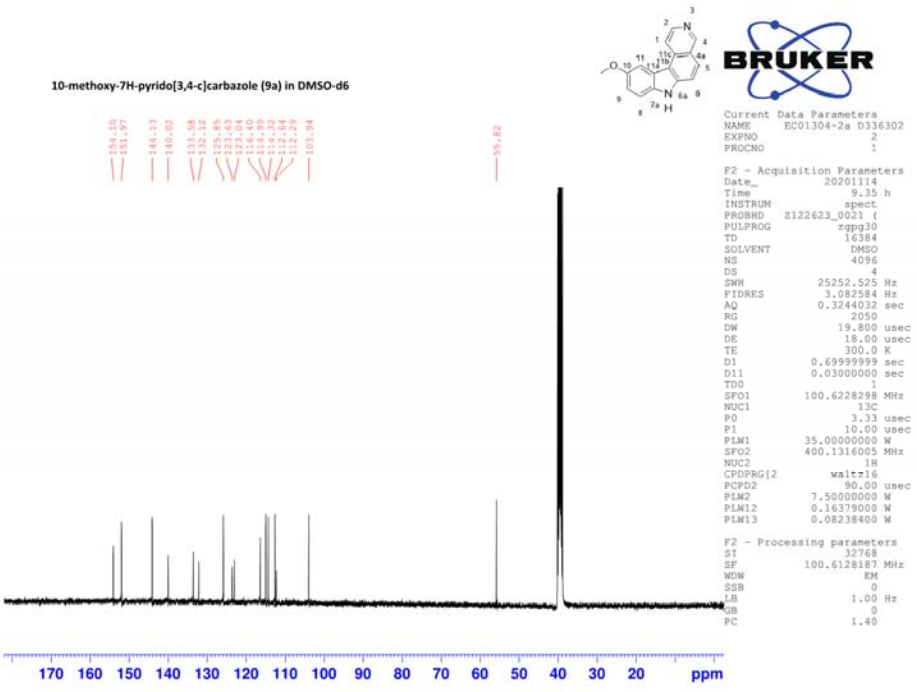

Figure S93. $^{13}C\{^{1}H\}$ NMR spectrum of 10-methoxy-*7H*-pyrido[3,4-*c*]carbazole (**9a**).

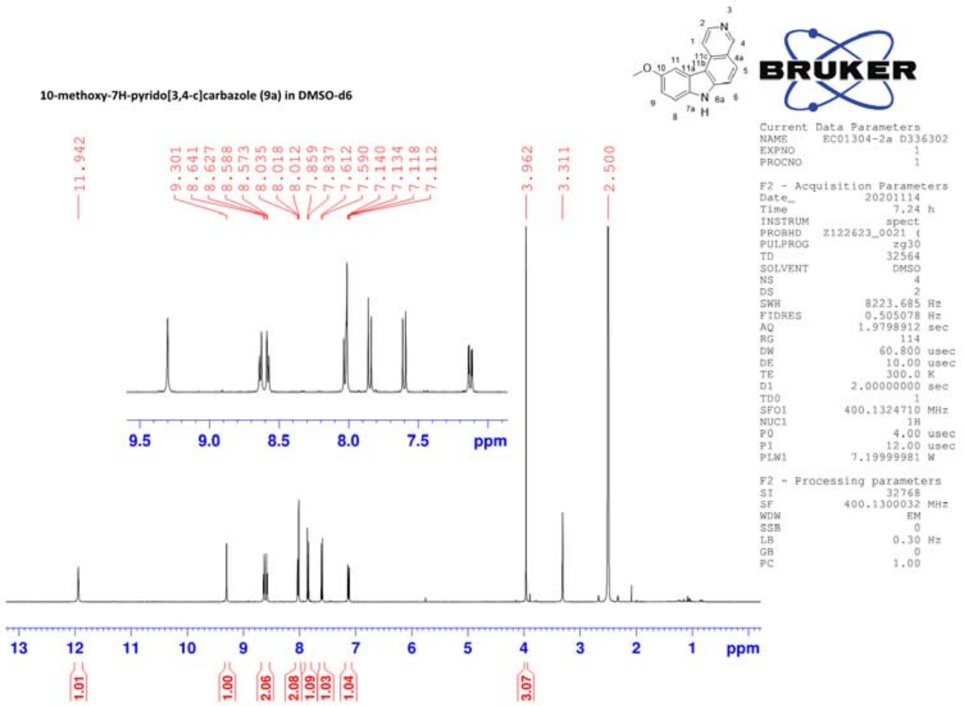

Figure S94. ^{1}H NMR spectrum of 10-methoxy-*7H*-pyrido[3,4-*c*]carbazole (**9a**).

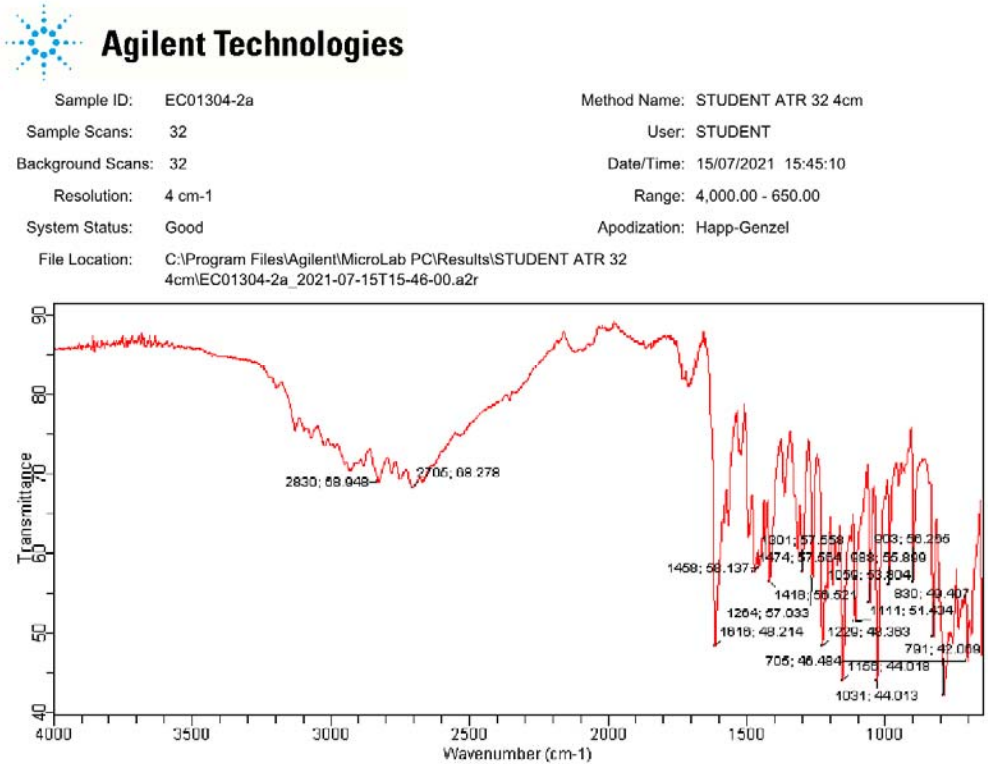

Figure S95. FT-IR spectrum of 10-methoxy-*7H*-pyrido[3,4-*c*]carbazole (**9a**).

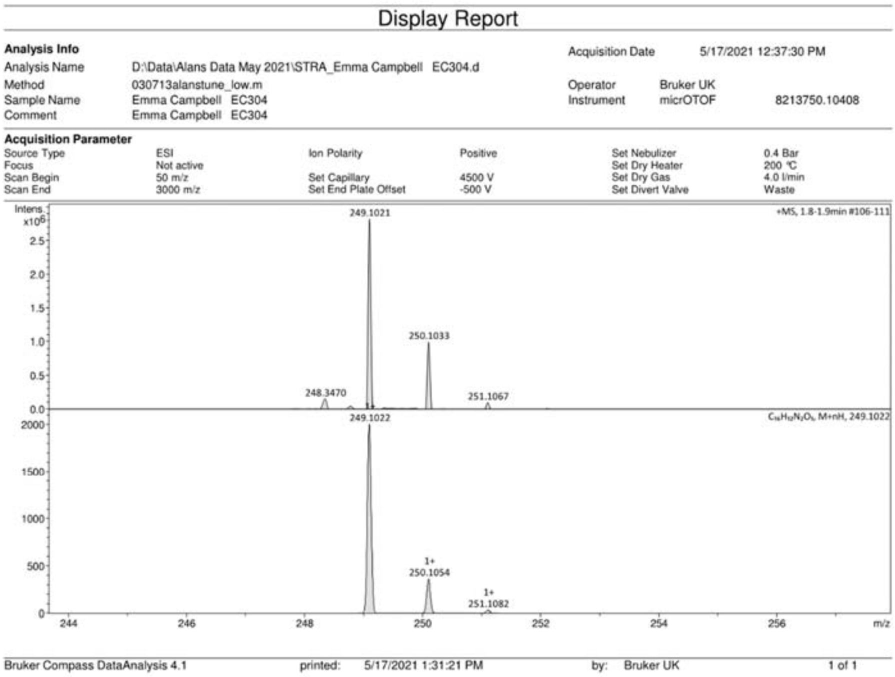

Figure S96. HRMS analysis of 10-methoxy-*7H*-pyrido[3,4-*c*]carbazole (**9a**).

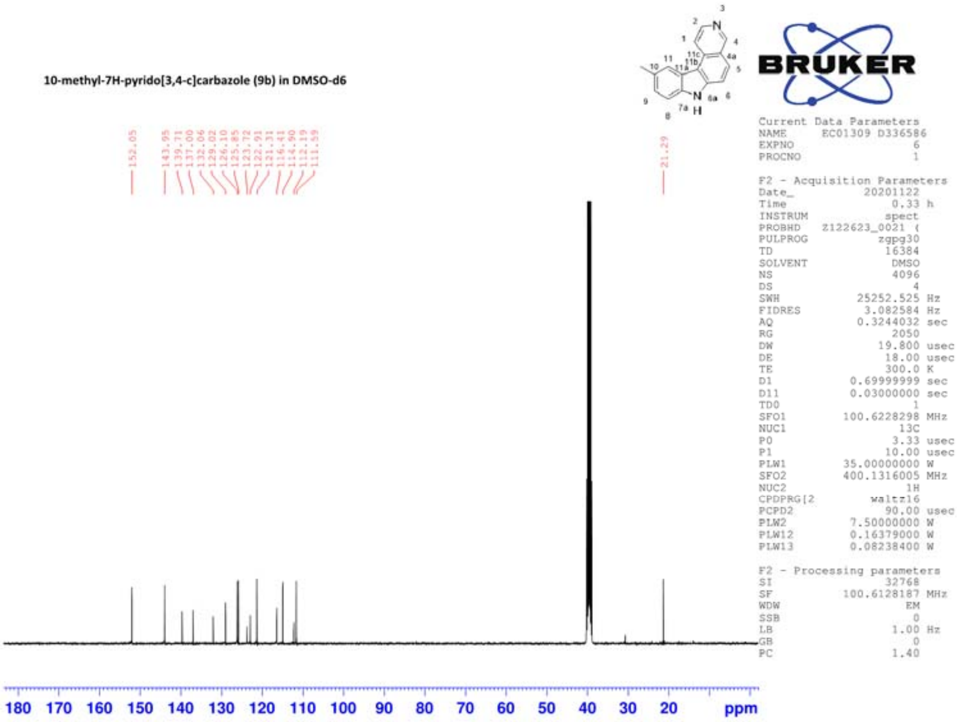

Figure S97. $^{13}C\{^{1}H\}$ NMR spectrum of 10-methyl-*7H*-pyrido[3,4-*c*]carbazole (**9b**).

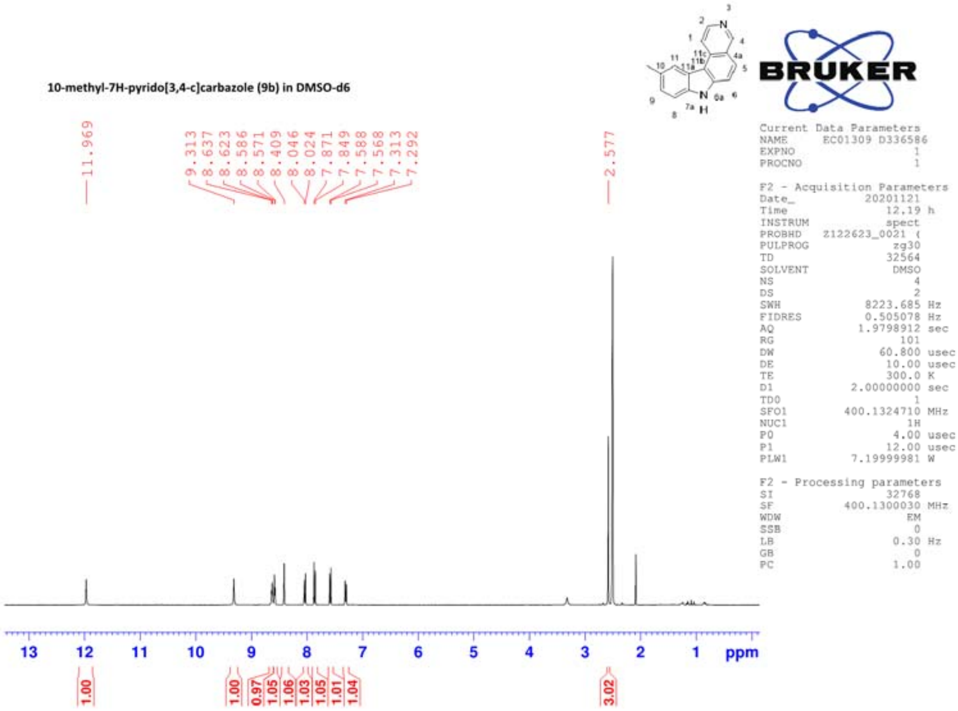

Figure S98. ^{1}H NMR spectrum of 10-methyl-*7H*-pyrido[3,4-*c*]carbazole (**9b**).

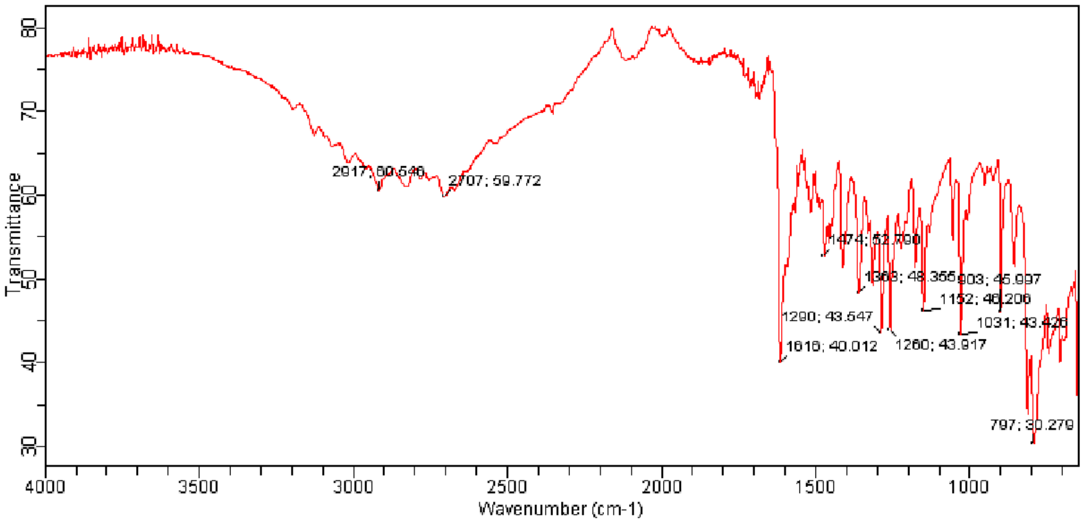

Figure S99. FT-IR spectrum of 10-methyl-*7H*-pyrido[3,4-*c*]carbazole (**9b**).

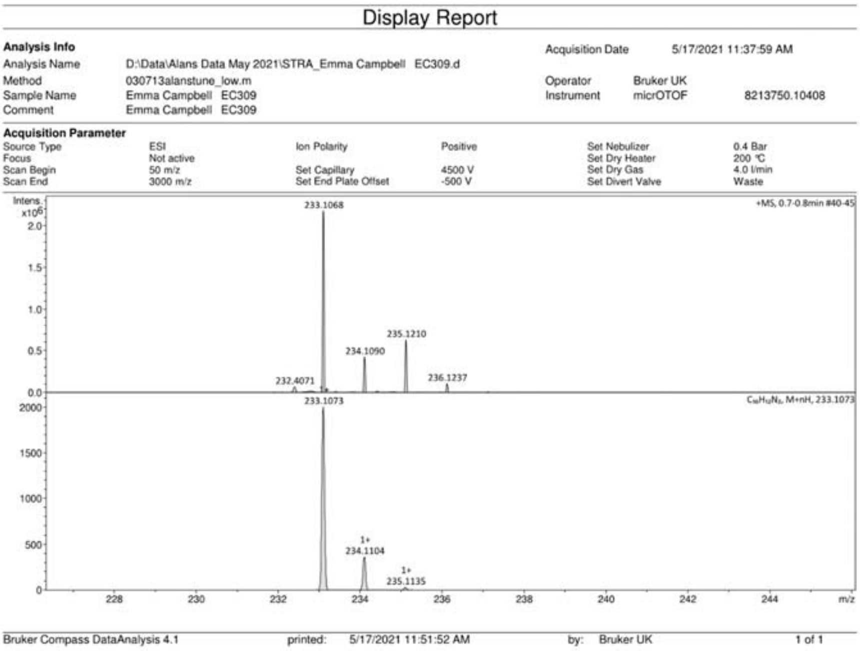

Figure S100. HRMS analysis of 10-methyl-*7H*-pyrido[3,4-*c*]carbazole (**9b**).

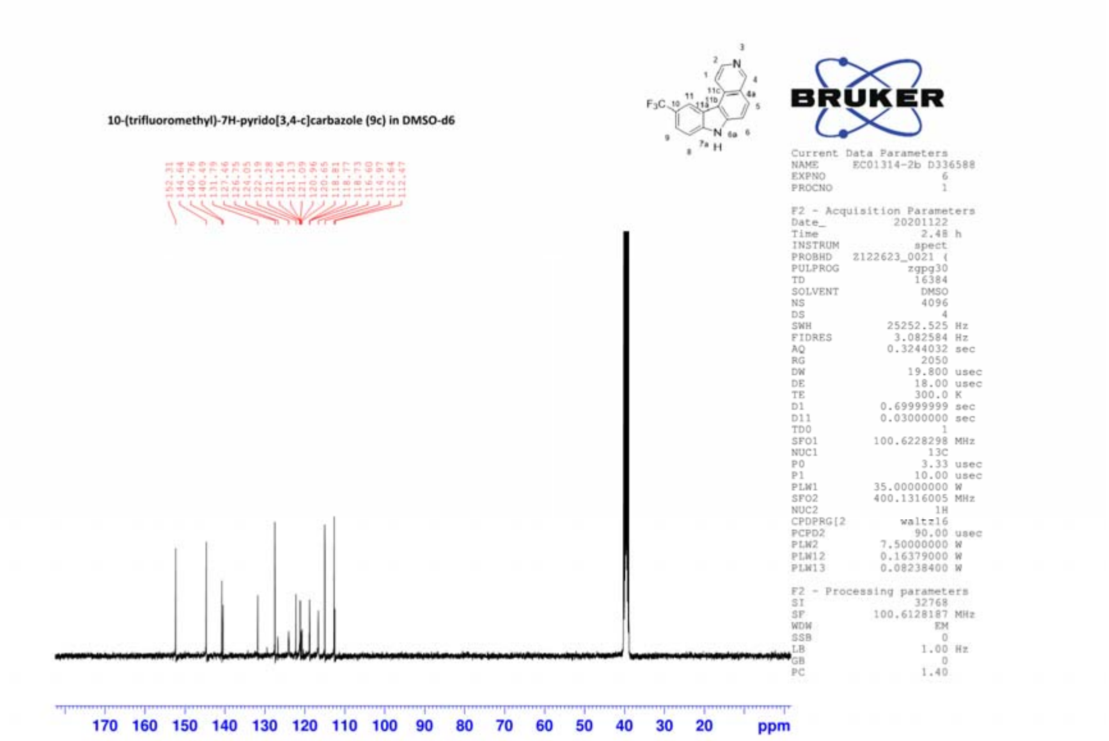

Figure S101. $^{13}C\{^1H\}$ NMR spectrum of 10-(trifluoromethyl)-*7H*-pyrido[3,4-*c*]carbazole (**9c**).

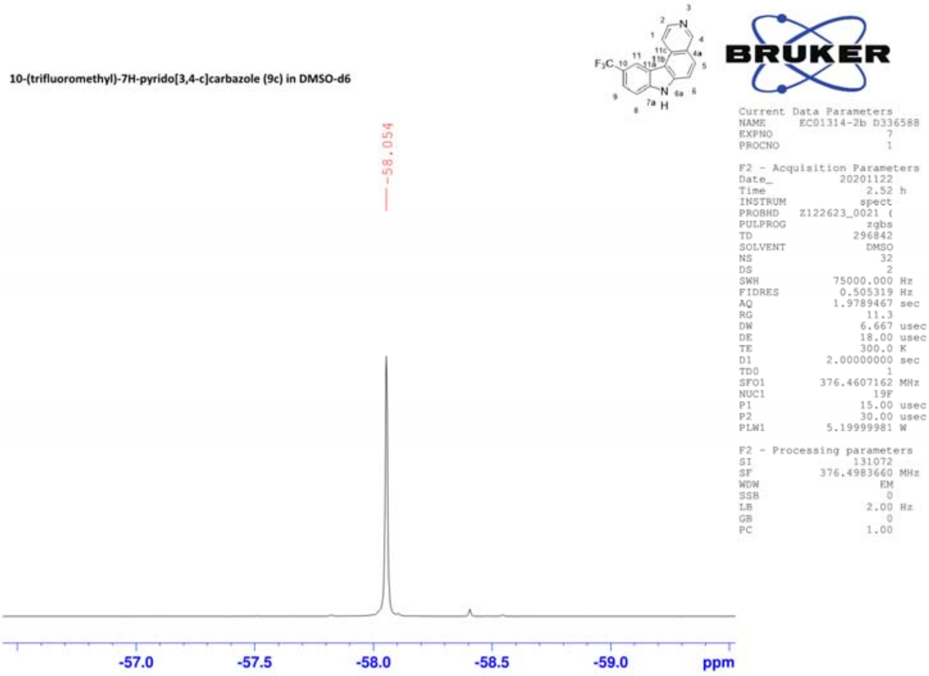

Figure S102. ^{19}F NMR spectrum of 10-(trifluoromethyl)-*7H*-pyrido[3,4-*c*]carbazole (**9c**).

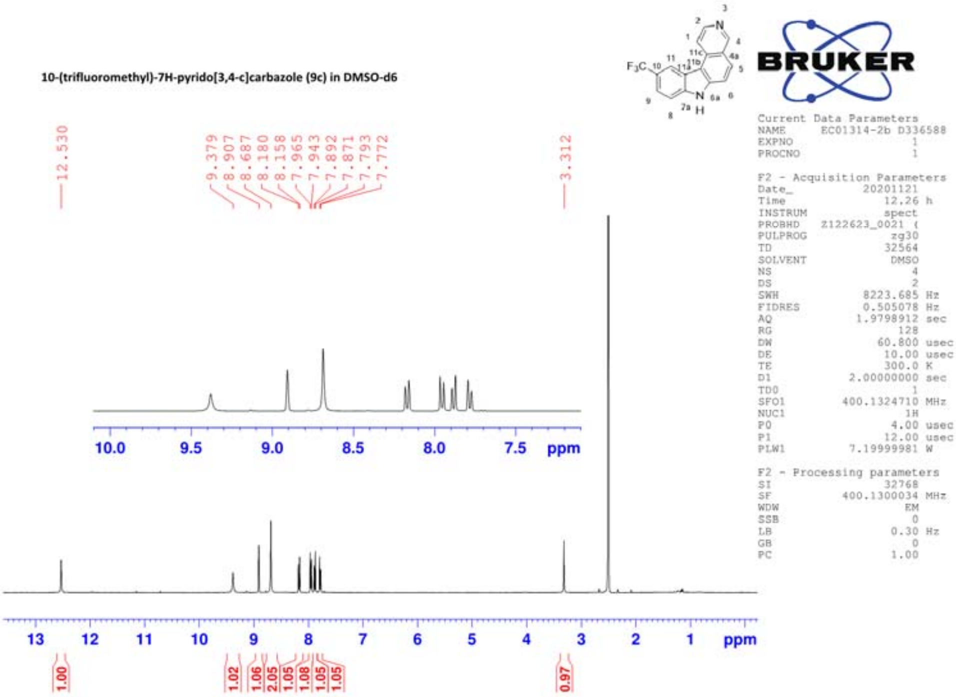

Figure S103. ^{1}H NMR spectrum of 10-(trifluoromethyl)-*7H*-pyrido[3,4-*c*]carbazole (**9c**).

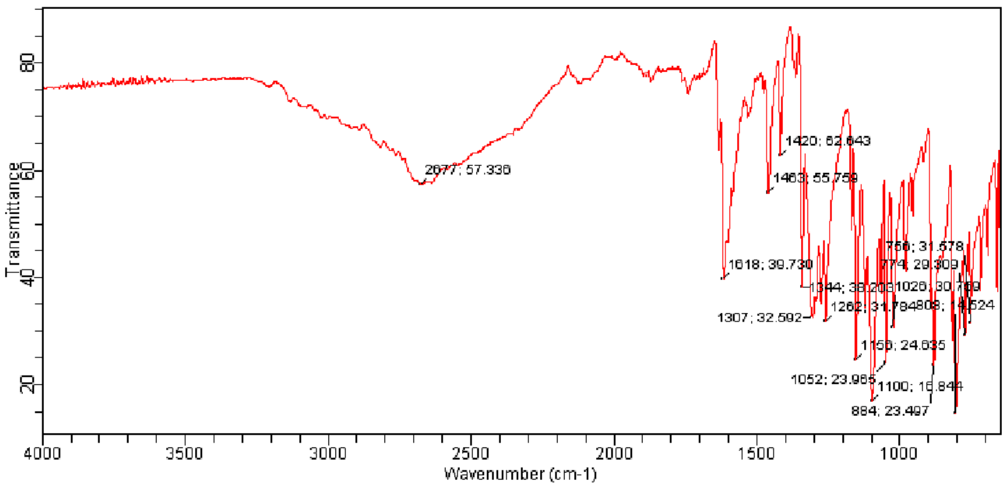

Figure S104. FT-IR spectrum of 10-(trifluoromethyl)-*7H*-pyrido[3,4-*c*]carbazole (**9c**).

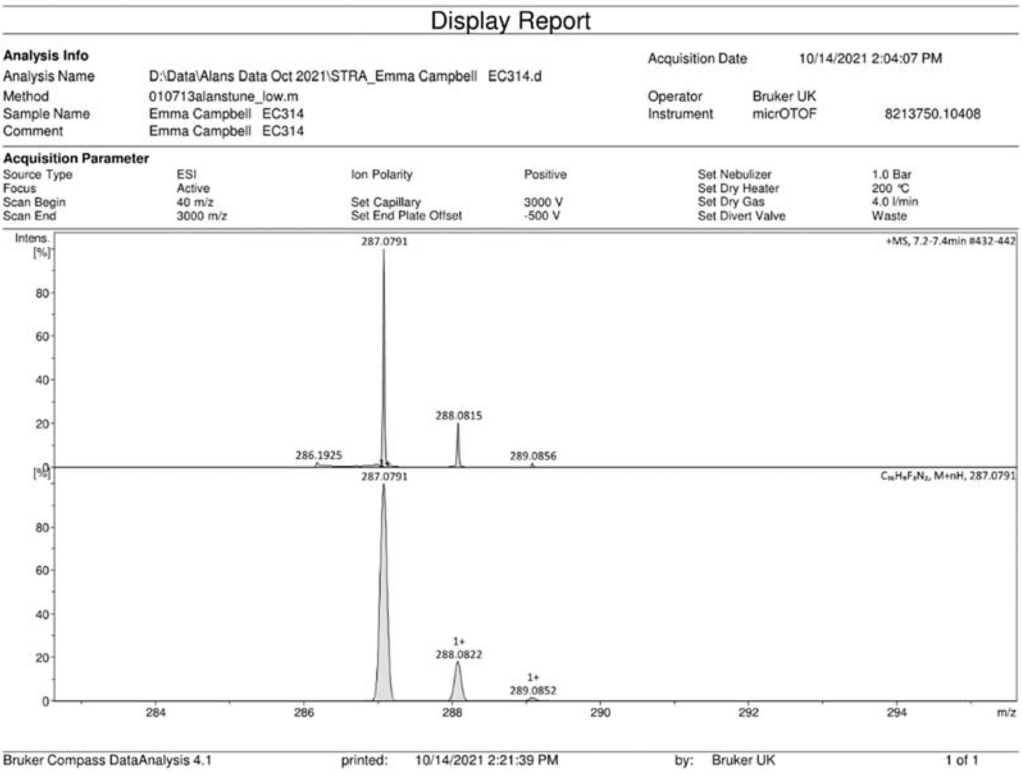

Figure S105. HRMS analysis of 10-(trifluoromethyl)-*7H*-pyrido[3,4-*c*]carbazole (**9c**).

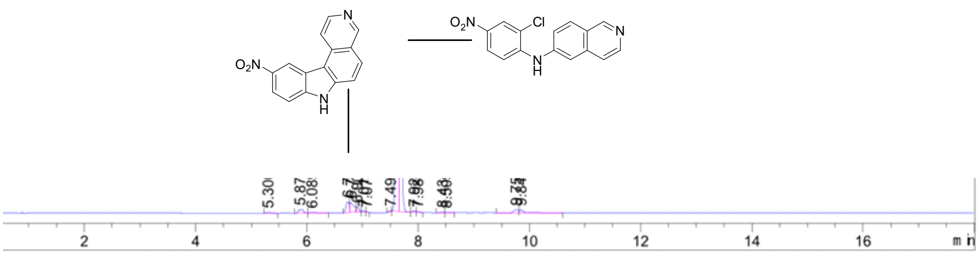

Figure S106. LC-MS analysis of crude reaction mixture for trace 10-nitro-7H-pyrido[3,4-c]carbazole (**9d**) and N-(2-chloro-4-nitrophenyl)isoquinolin-6-amine (**7e**).

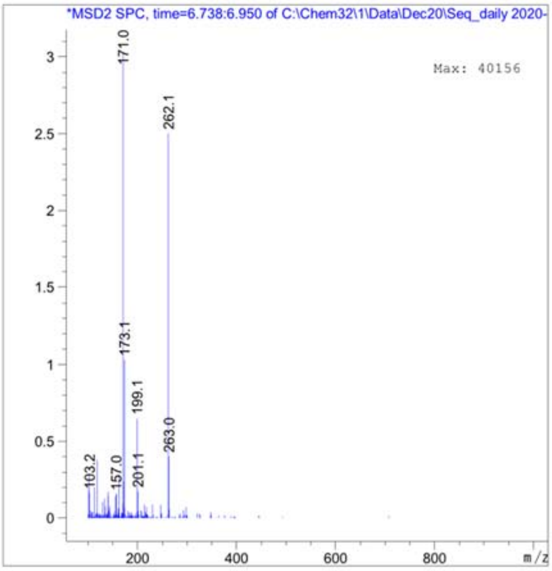

Figure S107. ESI MS of 10-nitro-7H-pyrido[3,4-c]carbazole (**9d**) retention time = 6.74 min.

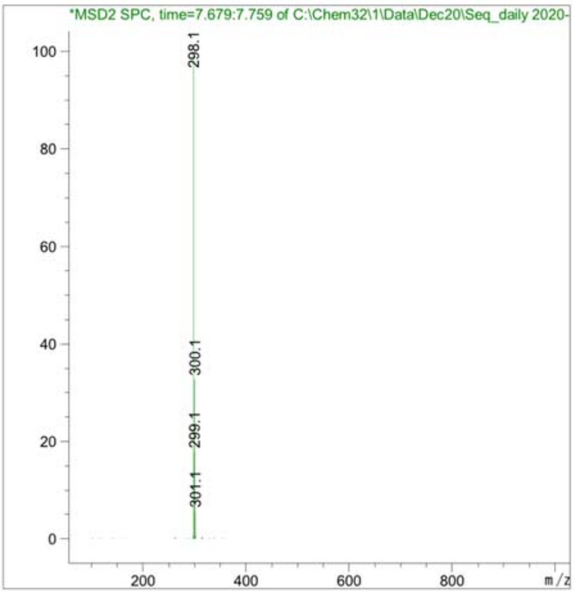

Figure S108. ESI MS of N-(2-chloro-4-nitrophenyl)isoquinolin-6-amine (**7e**) retention time = 7.68 min.

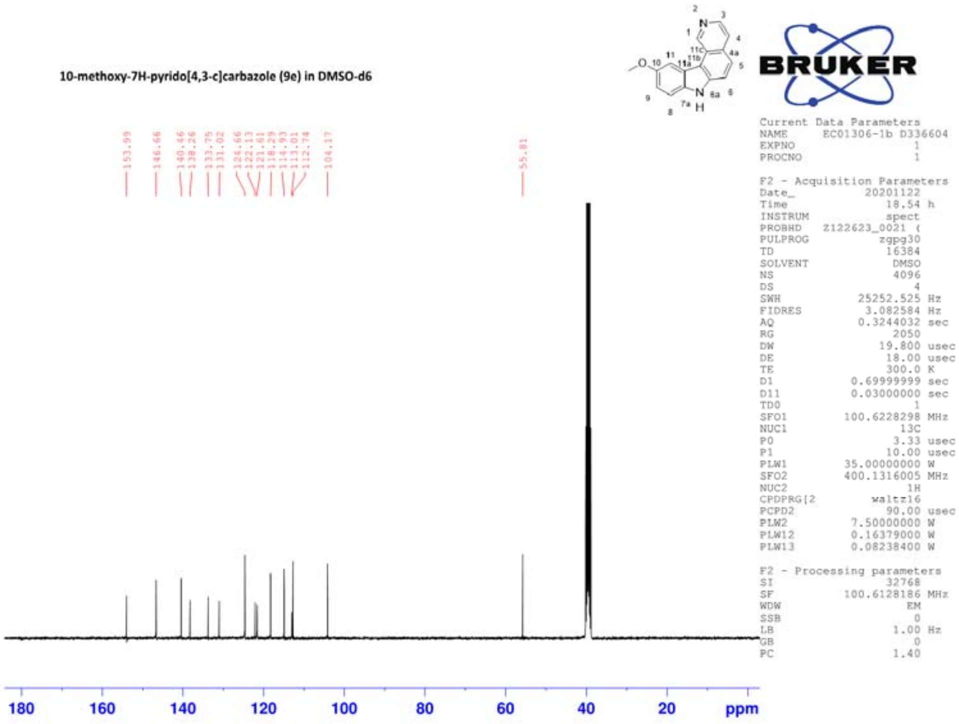

Figure S109. $^{13}C\{^{1}H\}$ NMR spectrum of 10-methoxy-*7H*-pyrido[4,3-*c*]carbazole (**9e**).

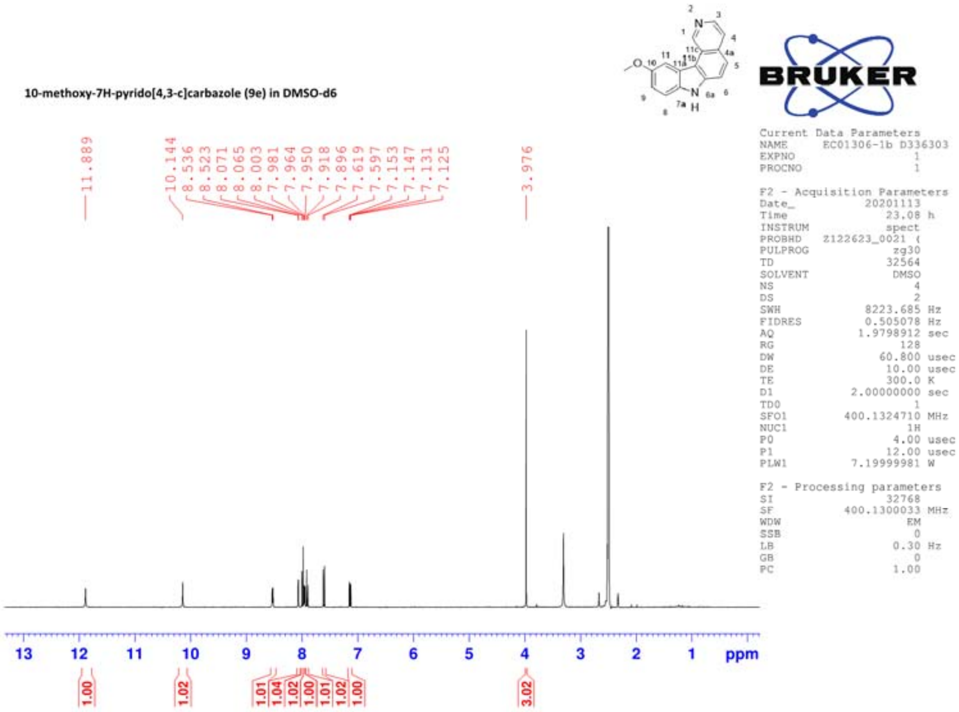

Figure S110. ^{1}H NMR spectrum of 10-methoxy-*7H*-pyrido[4,3-*c*]carbazole (**9e**).

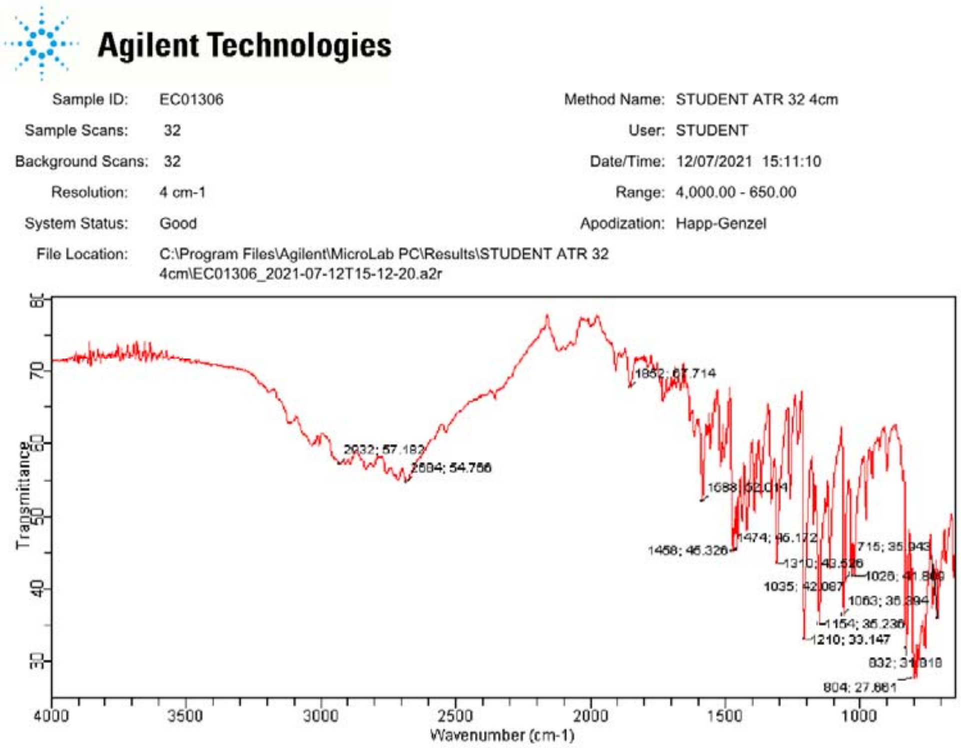

Figure S111. FT-IR spectrum of 10-methoxy-*7H*-pyrido[4,3-*c*]carbazole (**9e**).

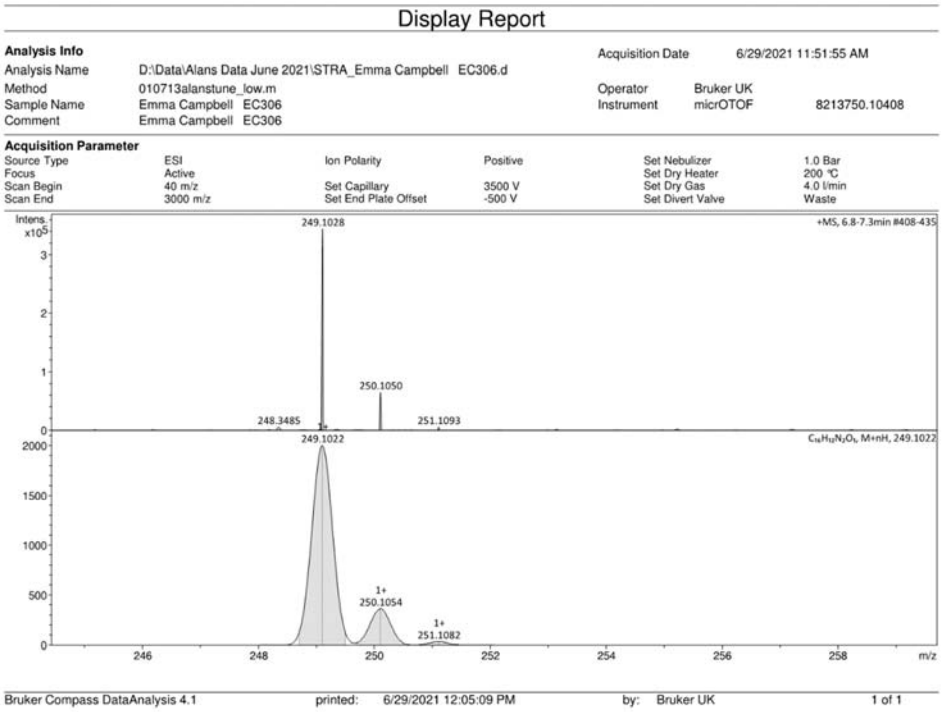

Figure S112. HRMS analysis of 10-methoxy-*7H*-pyrido[4,3-*c*]carbazole (**9e**).

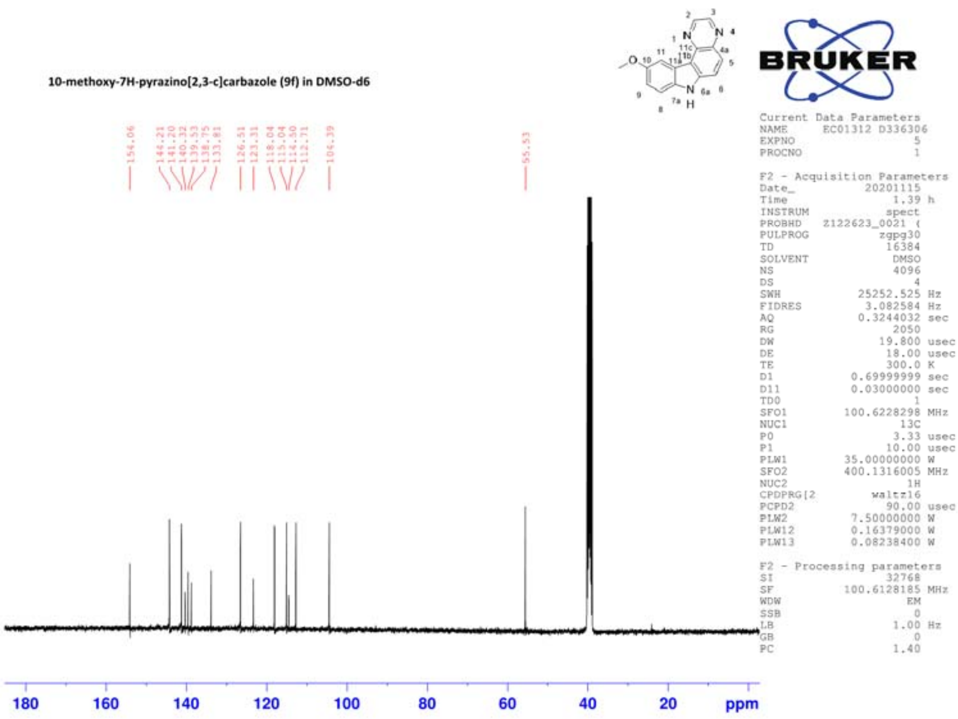

Figure S113. $^{13}C\{^{1}H\}$ NMR spectrum of 10-methoxy-*7H*-pyrazino[2,3-*c*]carbazole (**9f**).

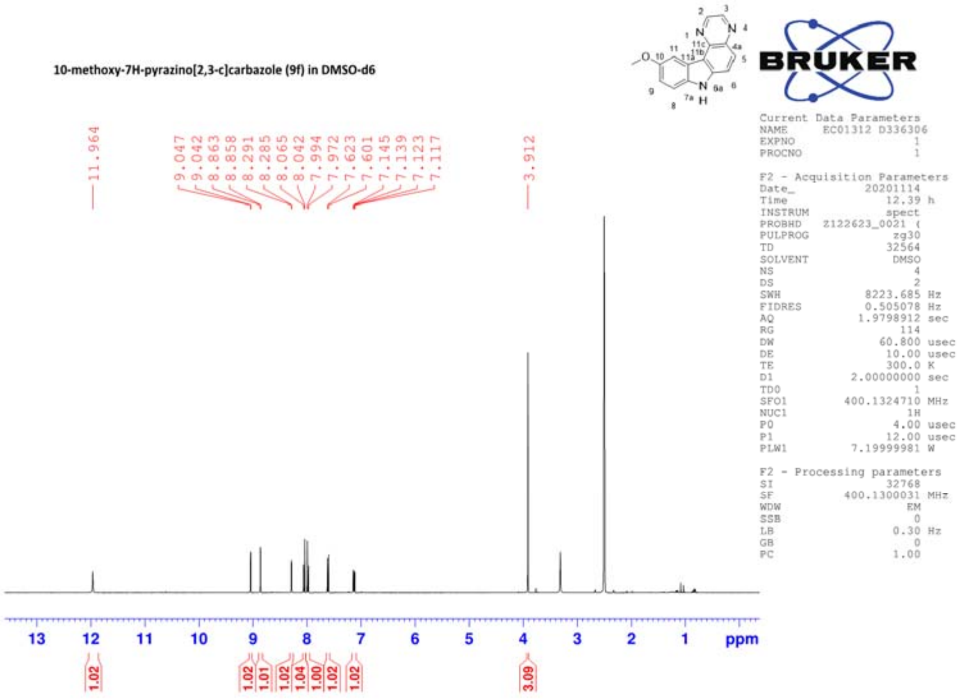

Figure S114. ^{1}H NMR spectrum of 10-methoxy-*7H*-pyrazino[2,3-*c*]carbazole (**9f**).

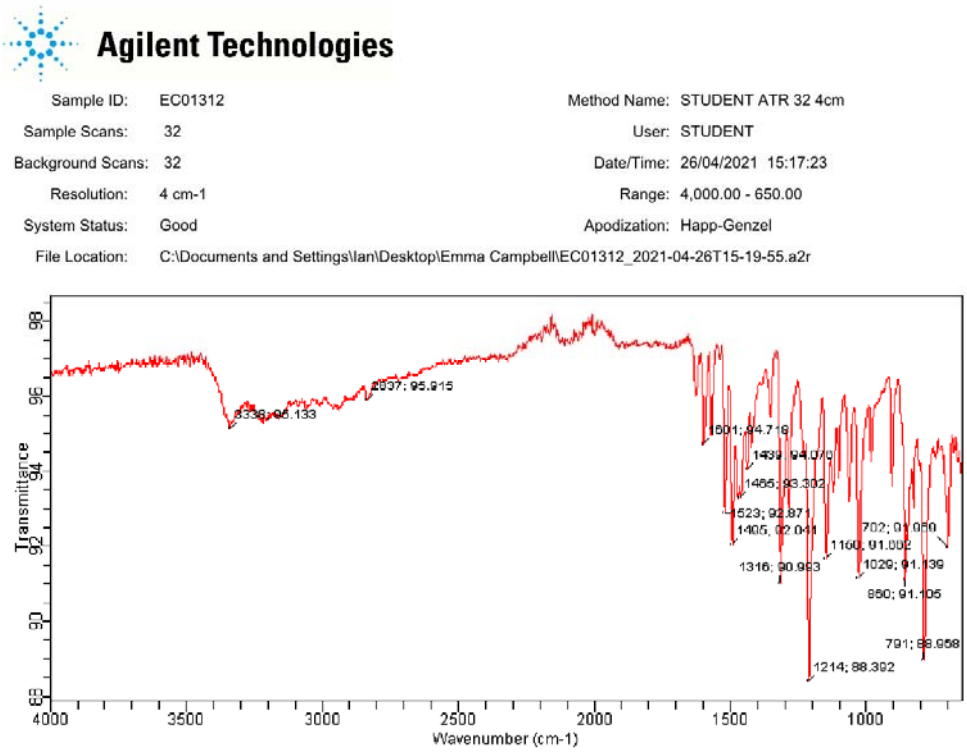

Figure S115. FT-IR spectrum of 10-methoxy-*7H*-pyrazino[2,3-*c*]carbazole (**9f**).

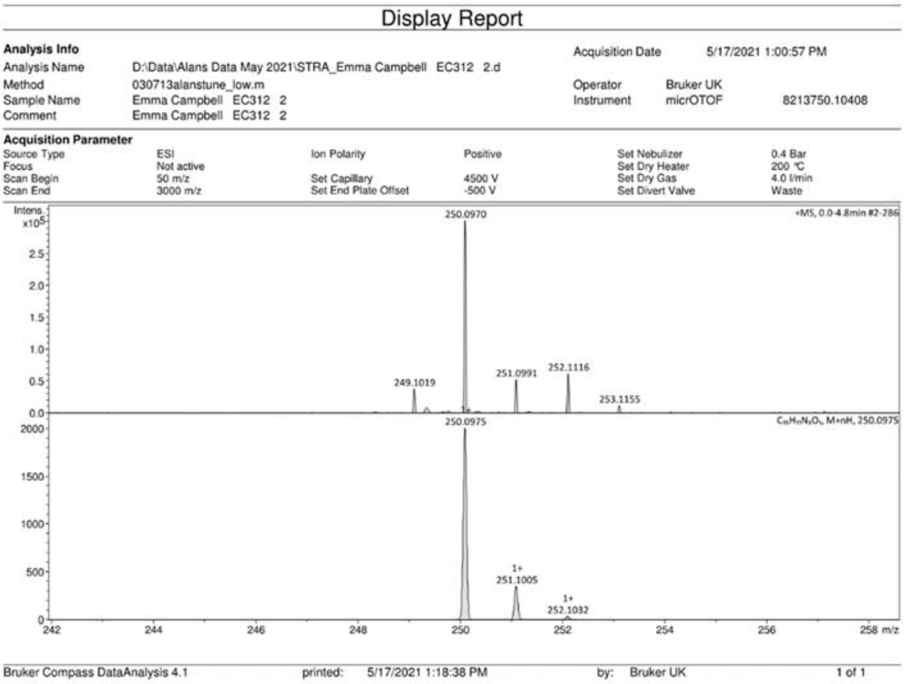

Figure S116. HRMS analysis of 10-methoxy-*7H*-pyrazino[2,3-*c*]carbazole (**9f**).

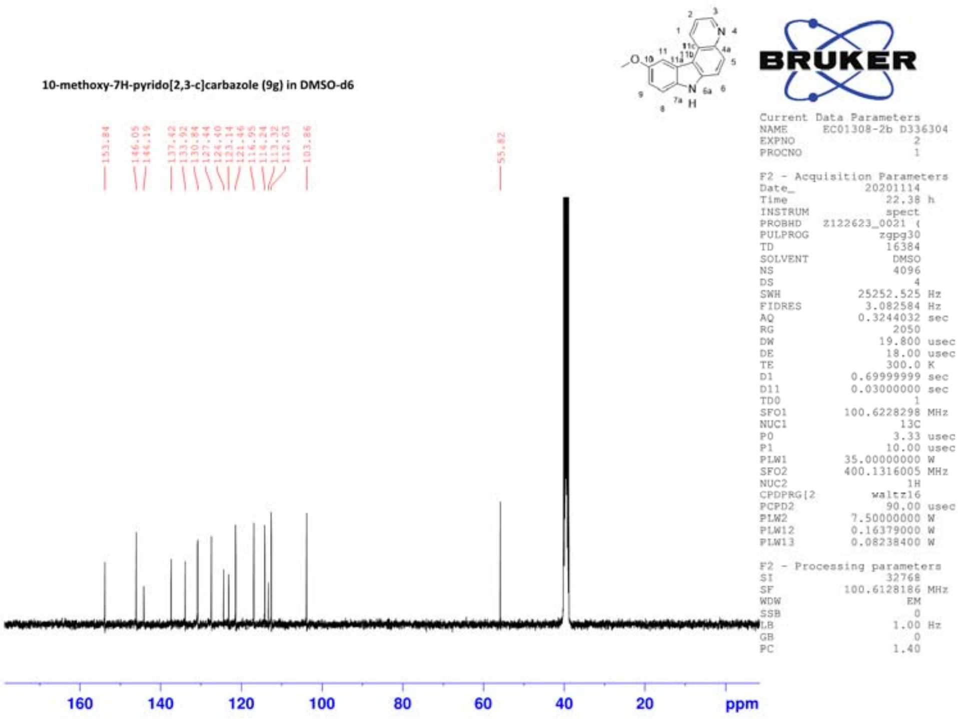

Figure S117. $^{13}C\{^{1}H\}$ NMR spectrum of 10-methoxy-*7H*-pyrido[2,3-*c*]carbazole (**9g**).

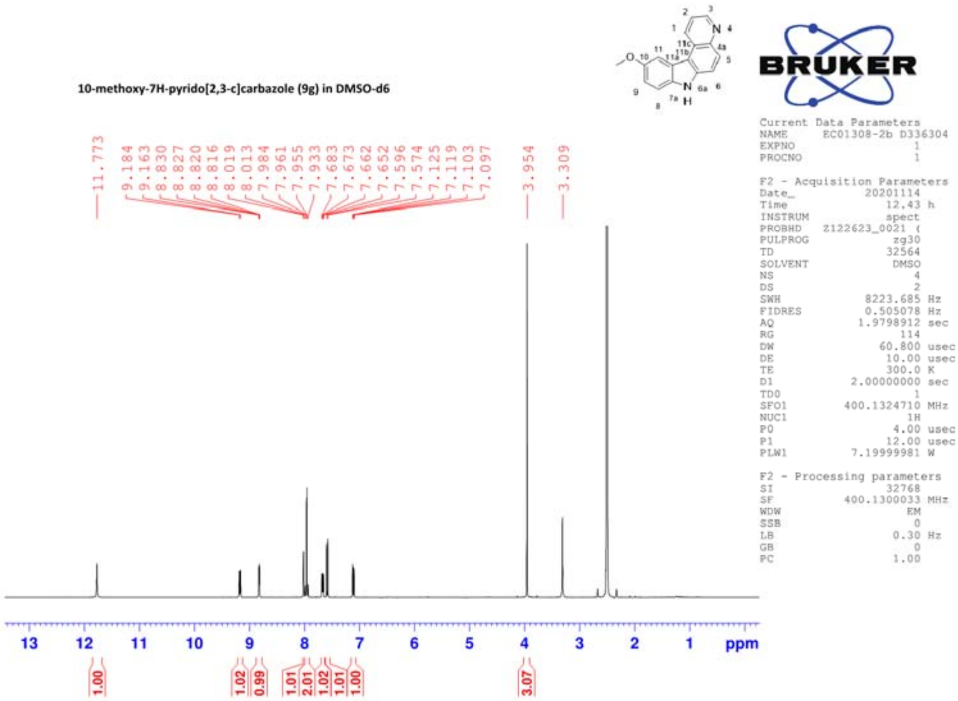

Figure S118. ^{1}H NMR spectrum of 10-methoxy-*7H*-pyrido[2,3-*c*]carbazole (**9g**).

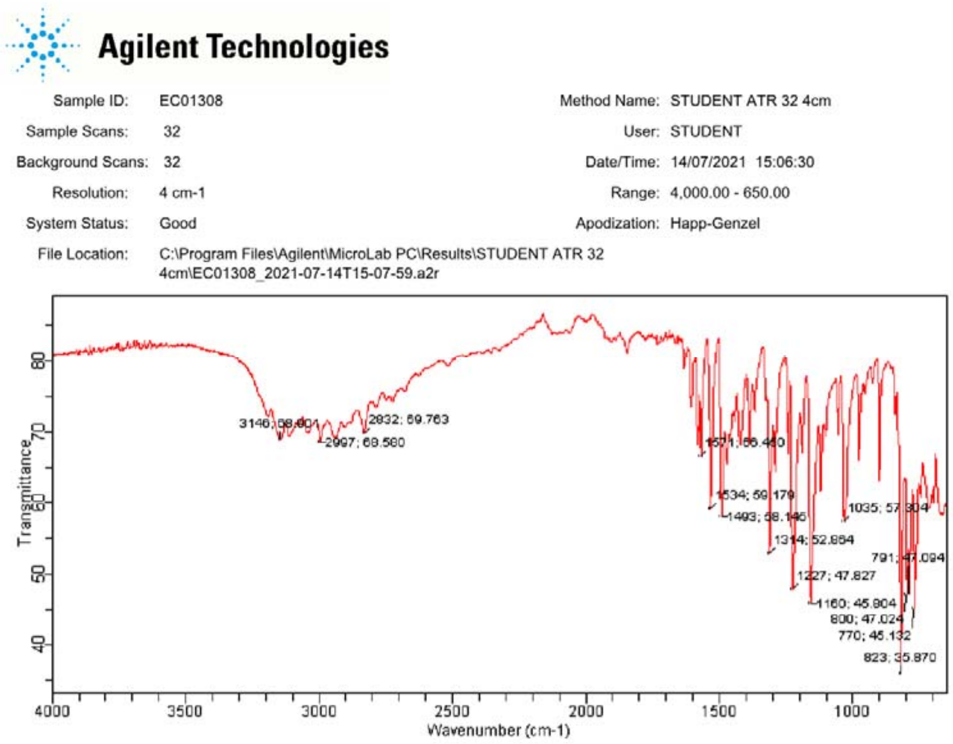

Figure S119. FT-IR spectrum of 10-methoxy-*7H*-pyrido[2,3-*c*]carbazole (**9g**).

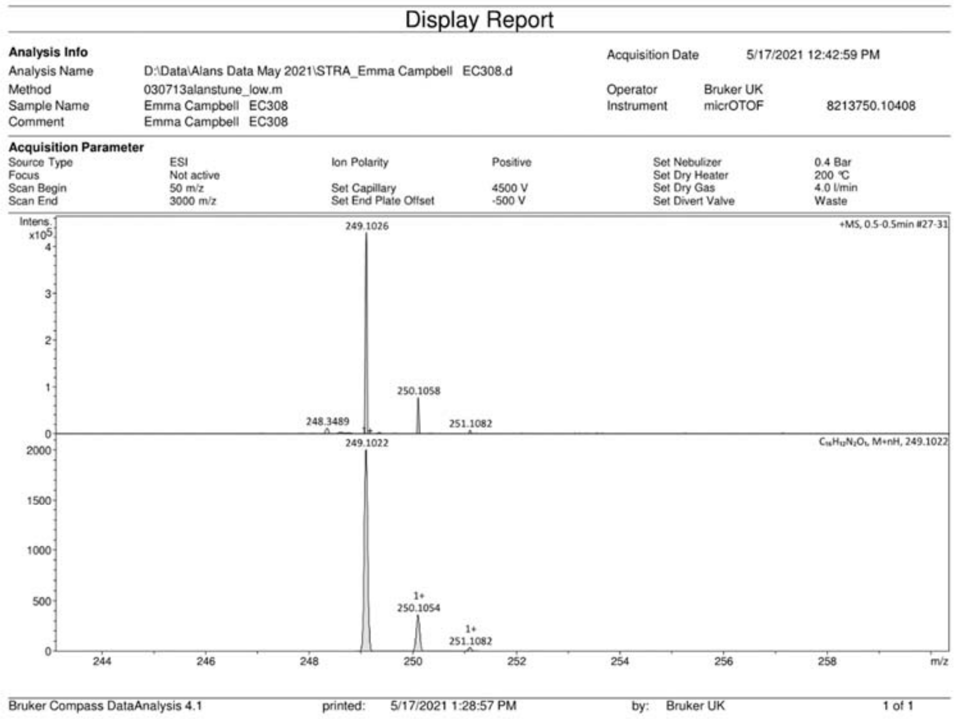

Figure S120. HRMS analysis of 10-methoxy-*7H*-pyrido[2,3-*c*]carbazole (**9g**).

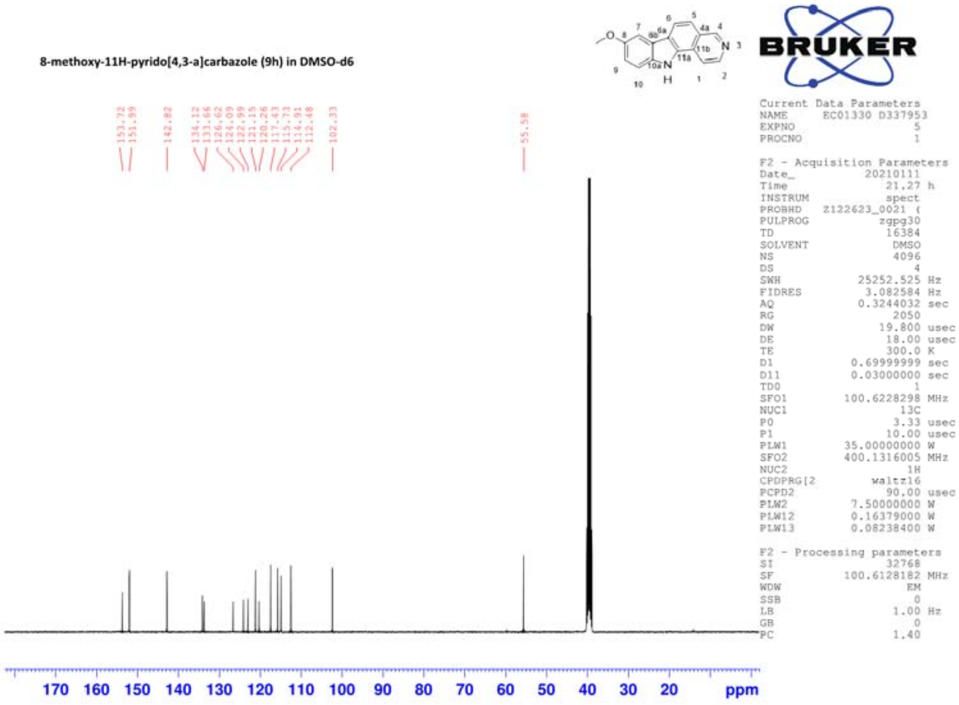

Figure S121. $^{13}C\{^{1}H\}$ NMR spectrum of 8-methoxy-11*H*-pyrido[4,3-*a*]carbazole (**9h**).

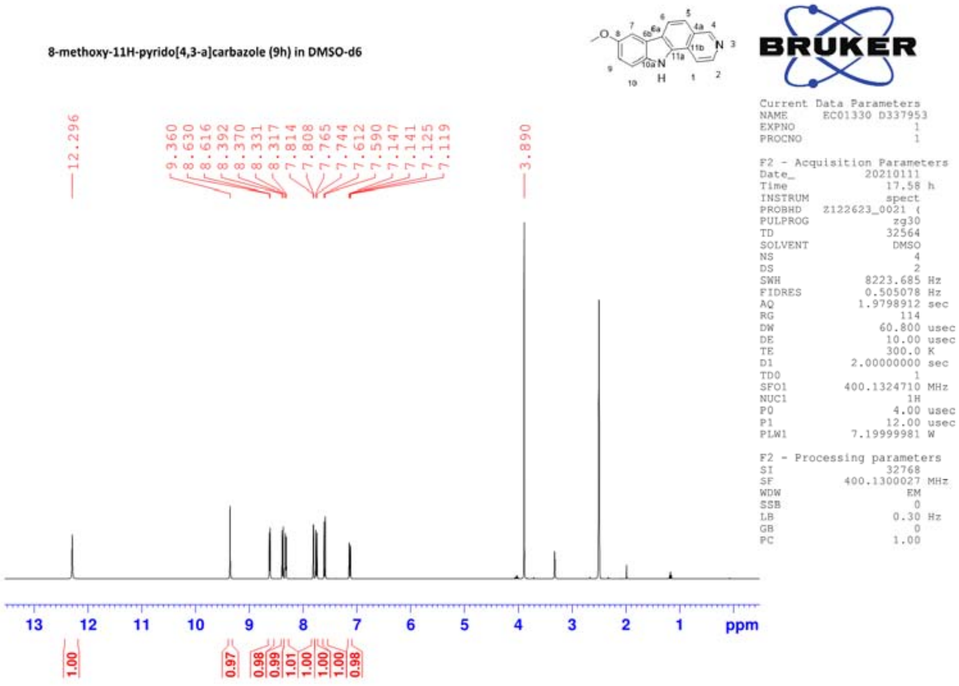

Figure S122. ^{1}H NMR spectrum of 8-methoxy-11*H*-pyrido[4,3-*a*]carbazole (**9h**).

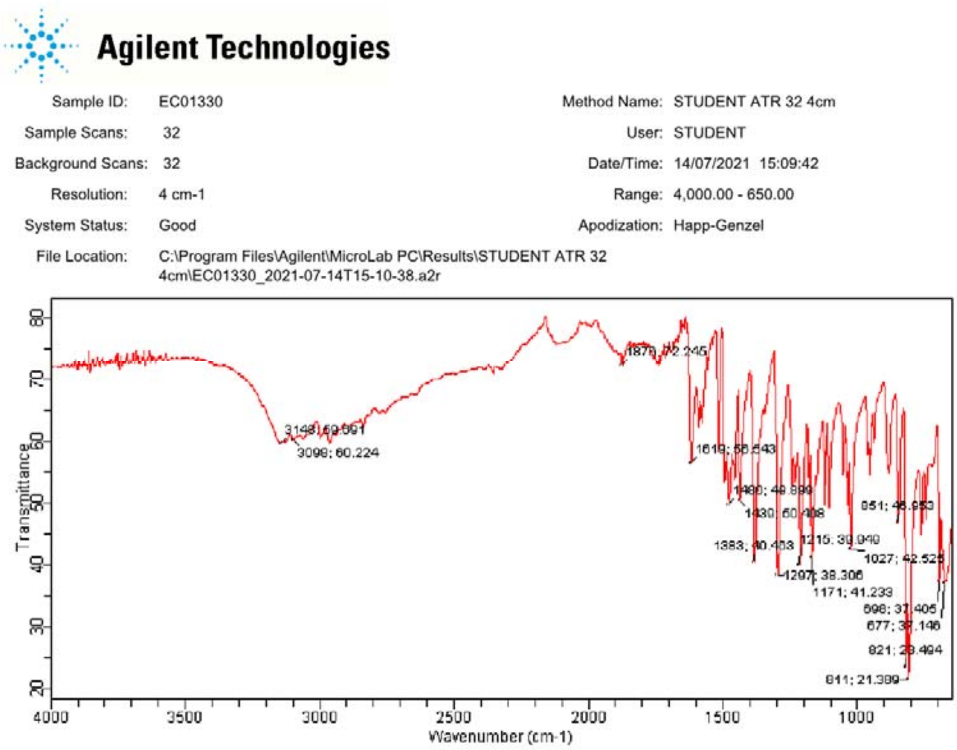

Figure S123. FT-IR spectrum of 8-methoxy-11*H*-pyrido[4,3-*a*]carbazole (**9h**).

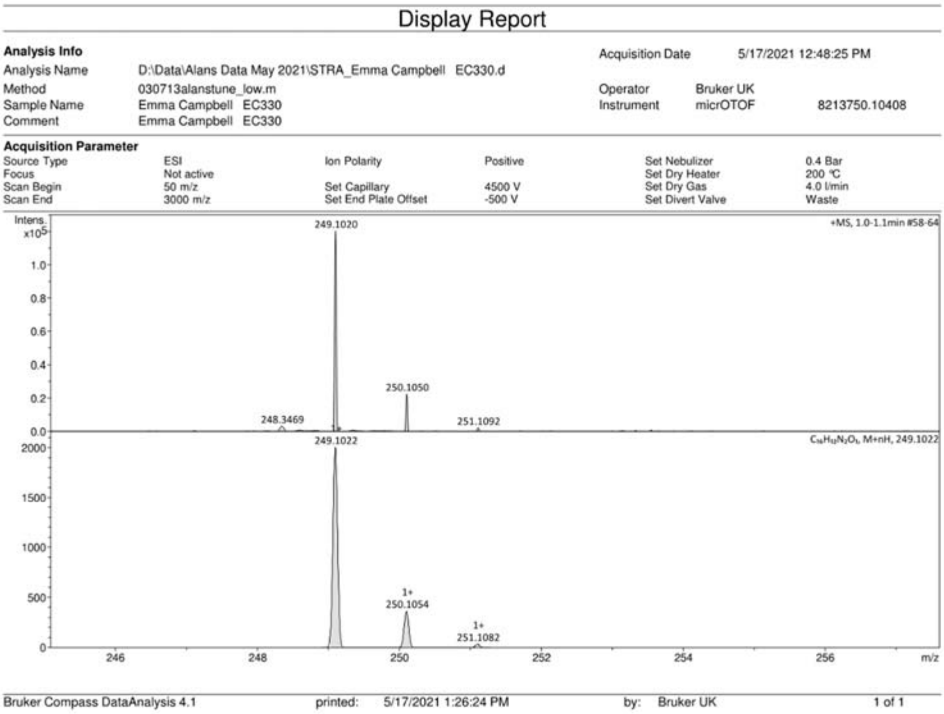

Figure S124. HRMS analysis of 8-methoxy-11*H*-pyrido[4,3-*a*]carbazole (**9h**).

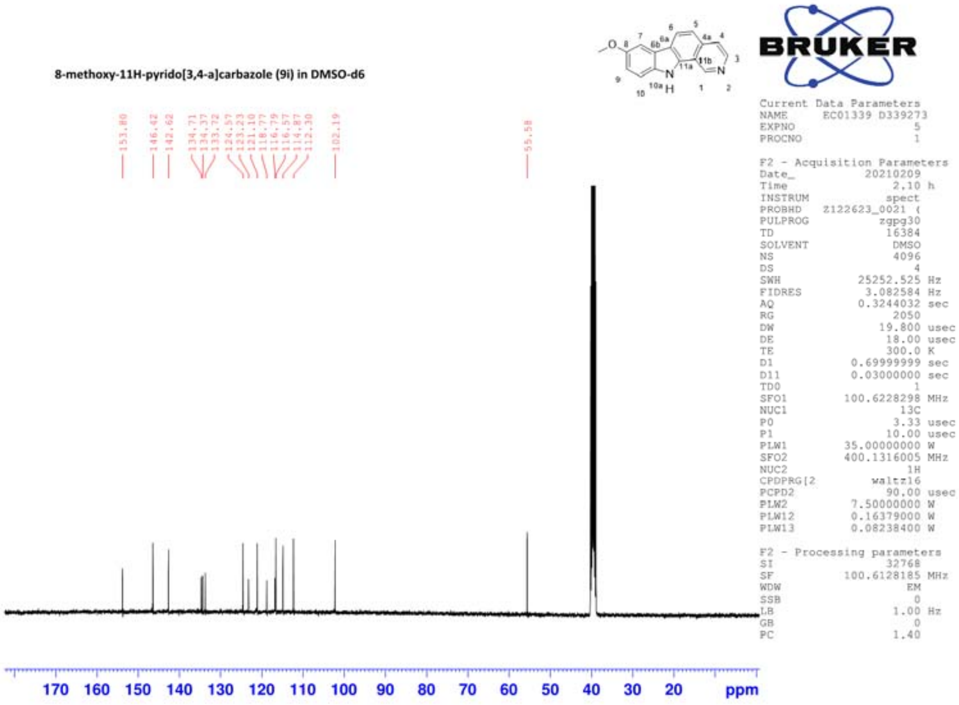

Figure S125. $^{13}C\{^1H\}$ NMR spectrum of 8-methoxy-11*H*-pyrido[3,4-*a*]carbazole (**9i**).

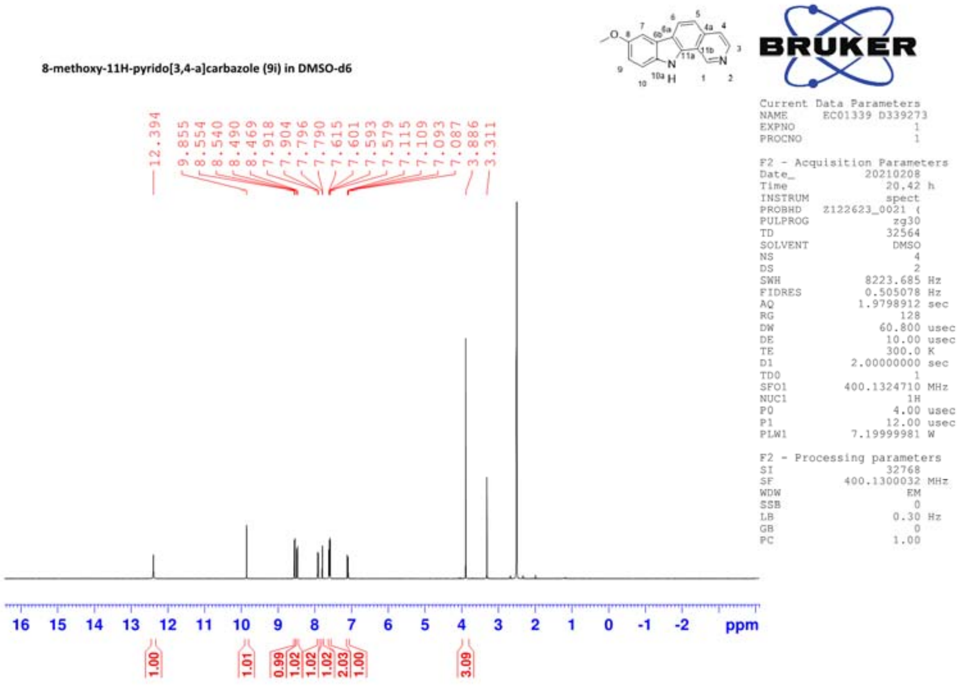

Figure S126. ^1H NMR spectrum of 8-methoxy-11*H*-pyrido[3,4-*a*]carbazole (**9i**).

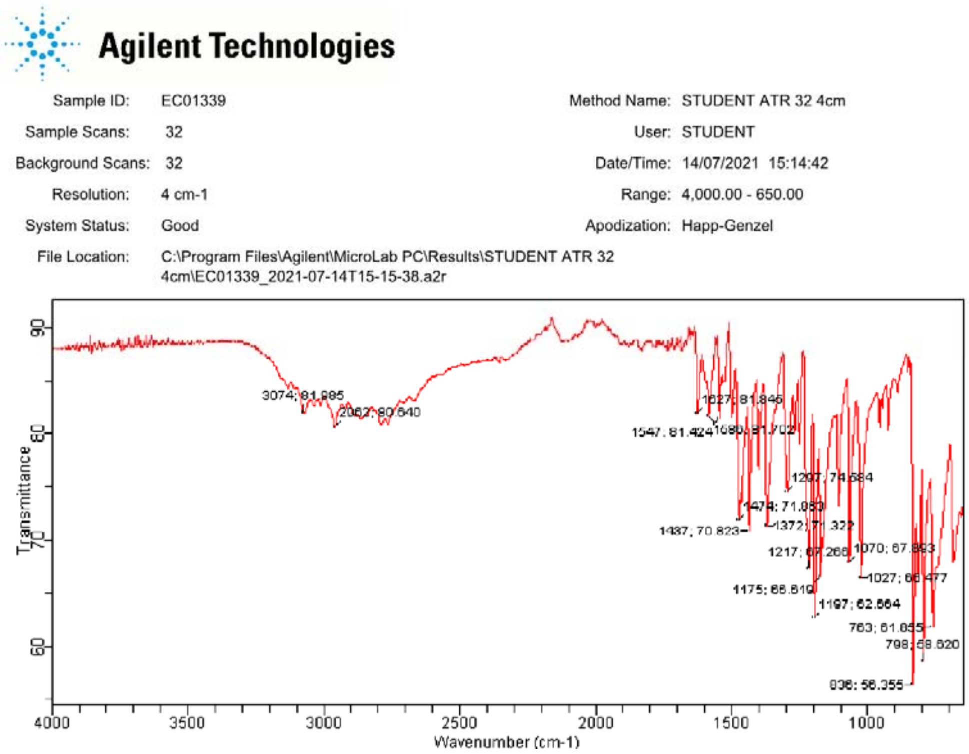

Figure S127. FT-IR spectrum of 8-methoxy-11*H*-pyrido[3,4-*a*]carbazole (**9i**).

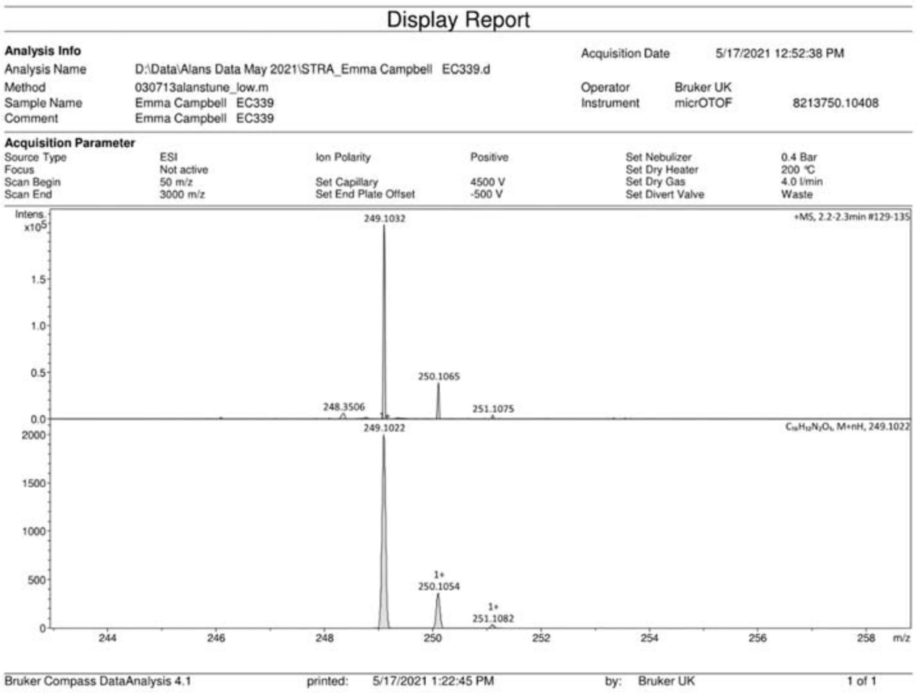

Figure S128. HRMS analysis of 8-methoxy-11*H*-pyrido[3,4-*a*]carbazole (**9i**).

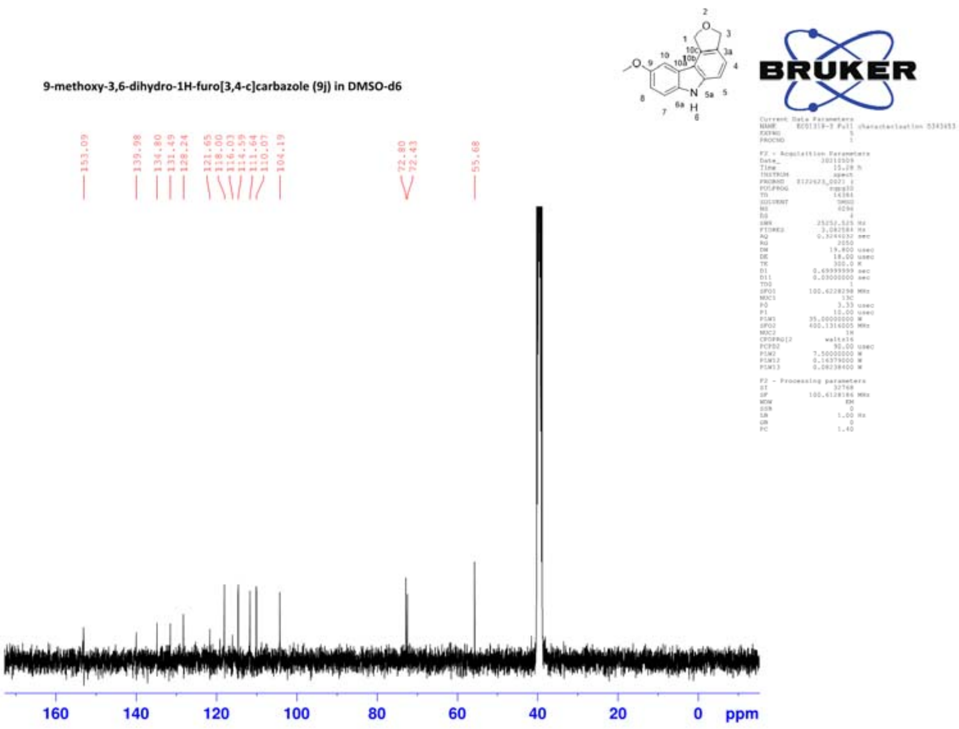

Figure S129. $^{13}C\{^{1}H\}$ NMR spectrum of 9-methoxy-3,6-dihydro-1H-furo[3,4-*c*]carbazole (**9j**).

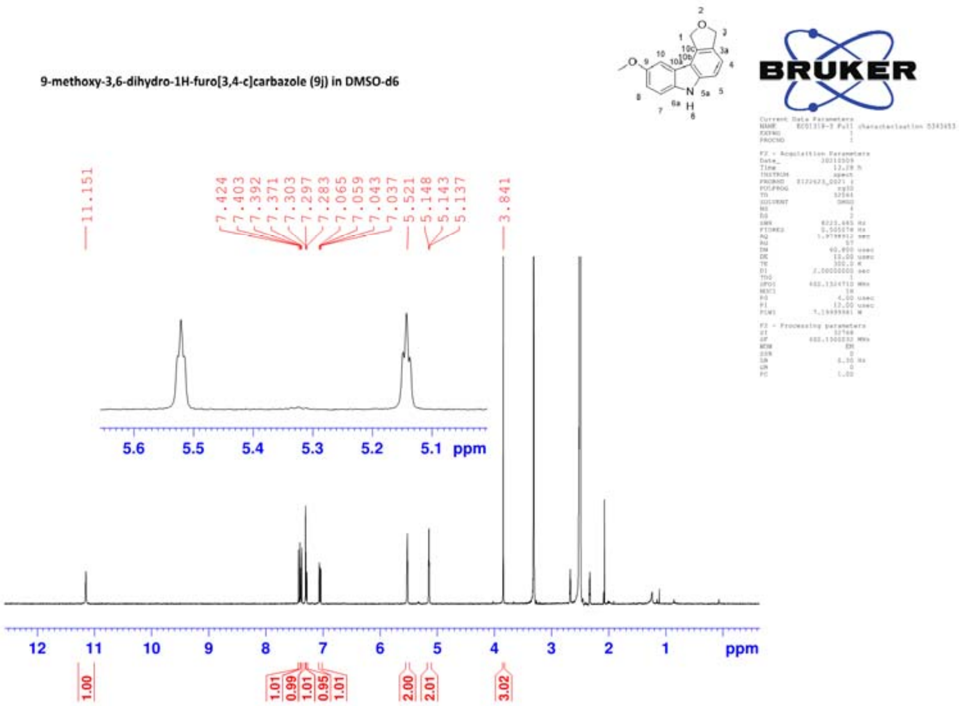

Figure S130. ^{1}H NMR spectrum of 9-methoxy-3,6-dihydro-1H-furo[3,4-*c*]carbazole (**9j**).

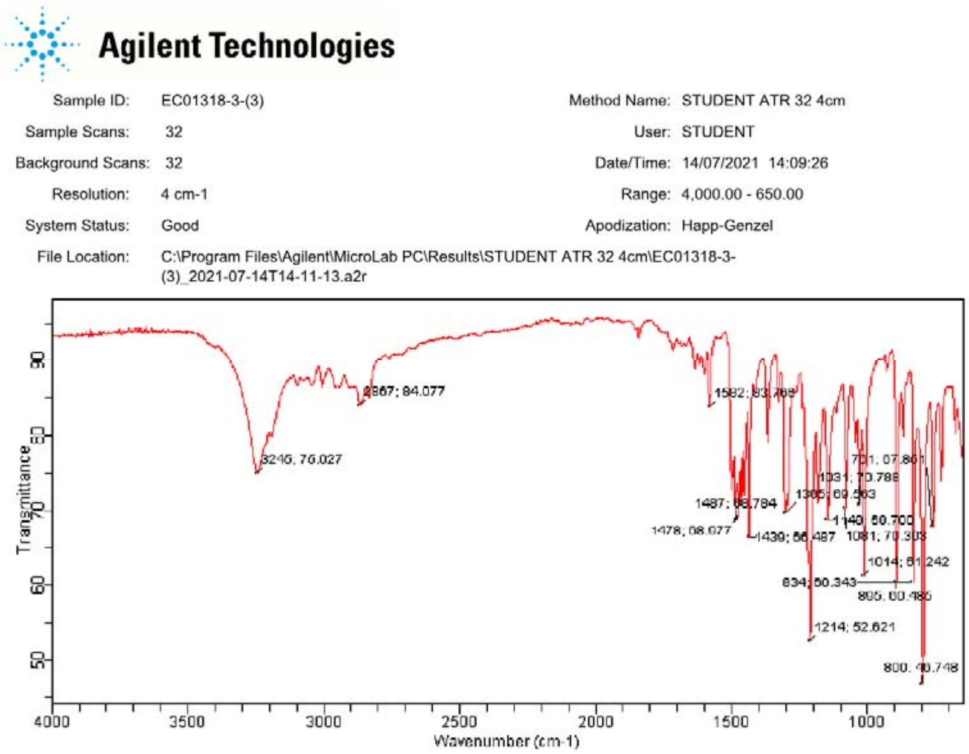

Figure S131. FT-IR spectrum of 9-methoxy-3,6-dihydro-1H-furo[3,4-*c*]carbazole (**9j**).

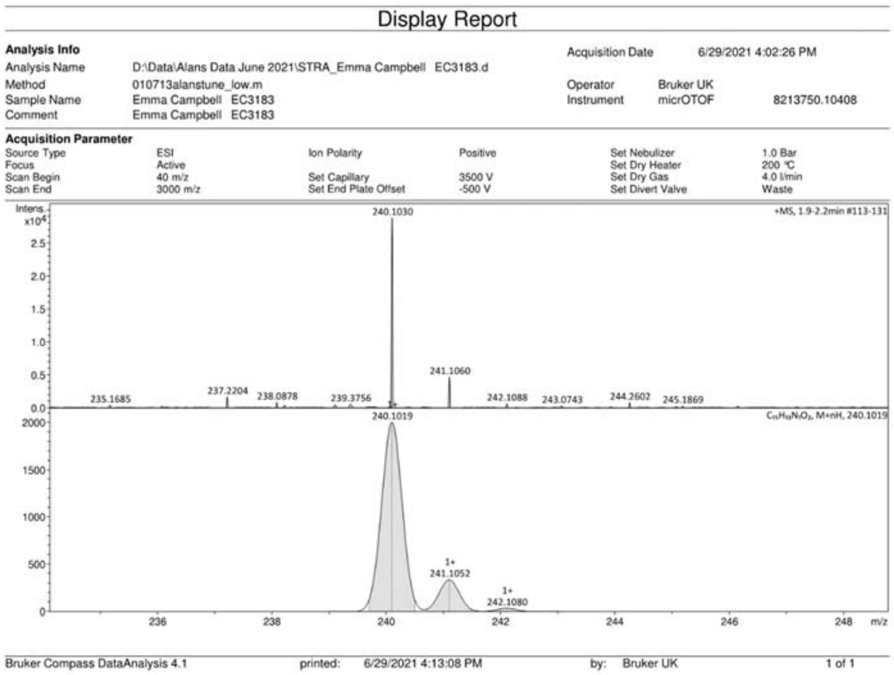

Figure S132. HRMS analysis of 9-methoxy-3,6-dihydro-1H-furo[3,4-*c*]carbazole (**9j**).

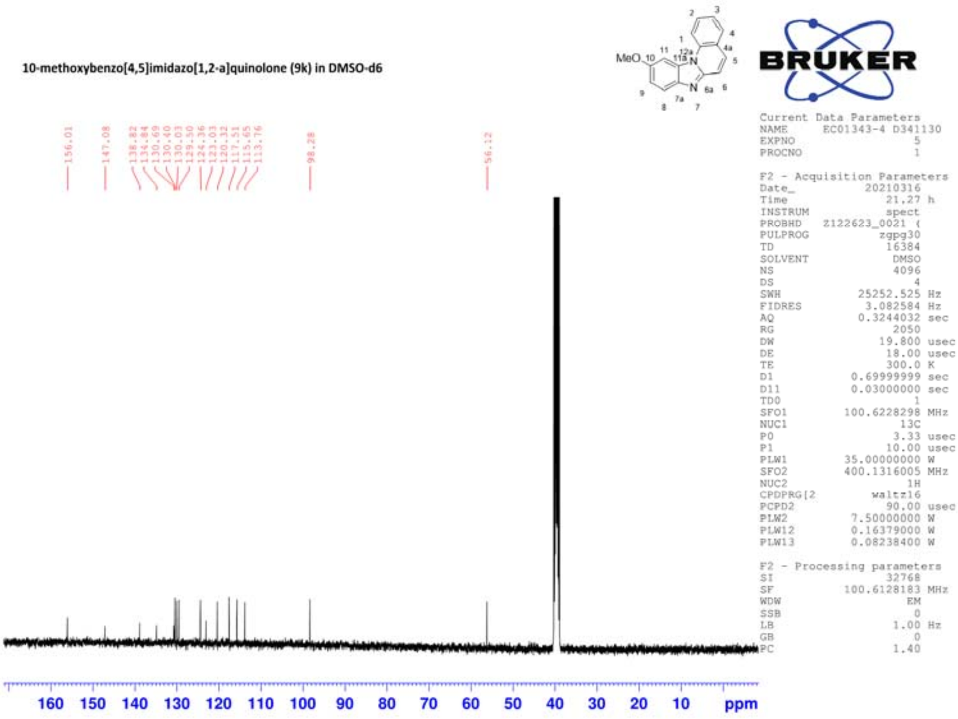

Figure S133. $^{13}C\{^{1}H\}$ NMR spectrum of 10-methoxybenzo[4,5]imidazo[1,2-*a*]quinolone (**9k**).

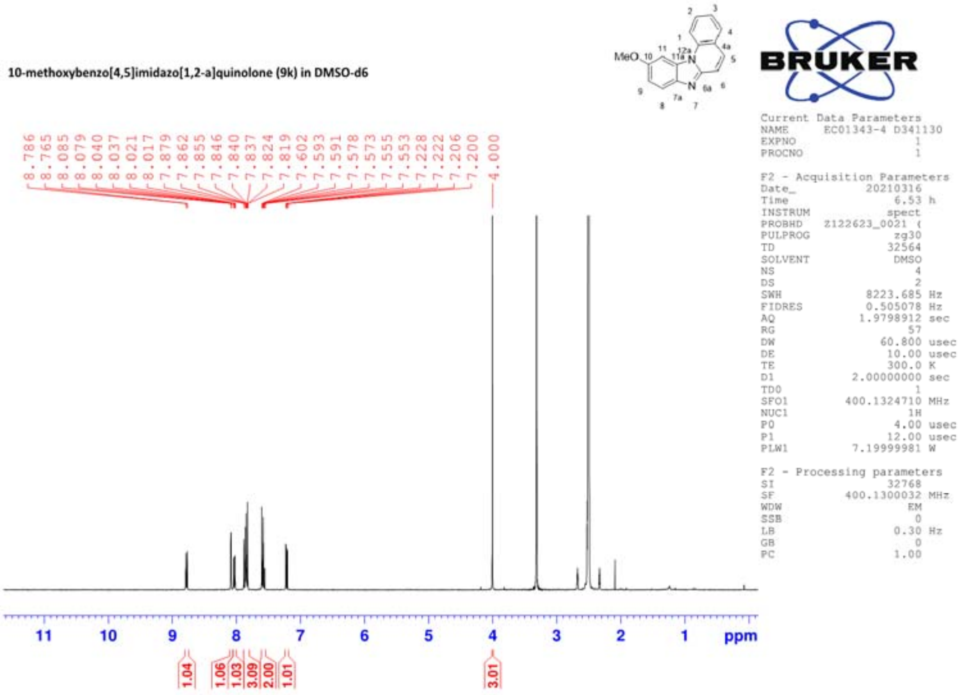

Figure S134. ^{1}H NMR spectrum of 10-methoxybenzo[4,5]imidazo[1,2-*a*]quinolone (**9k**).

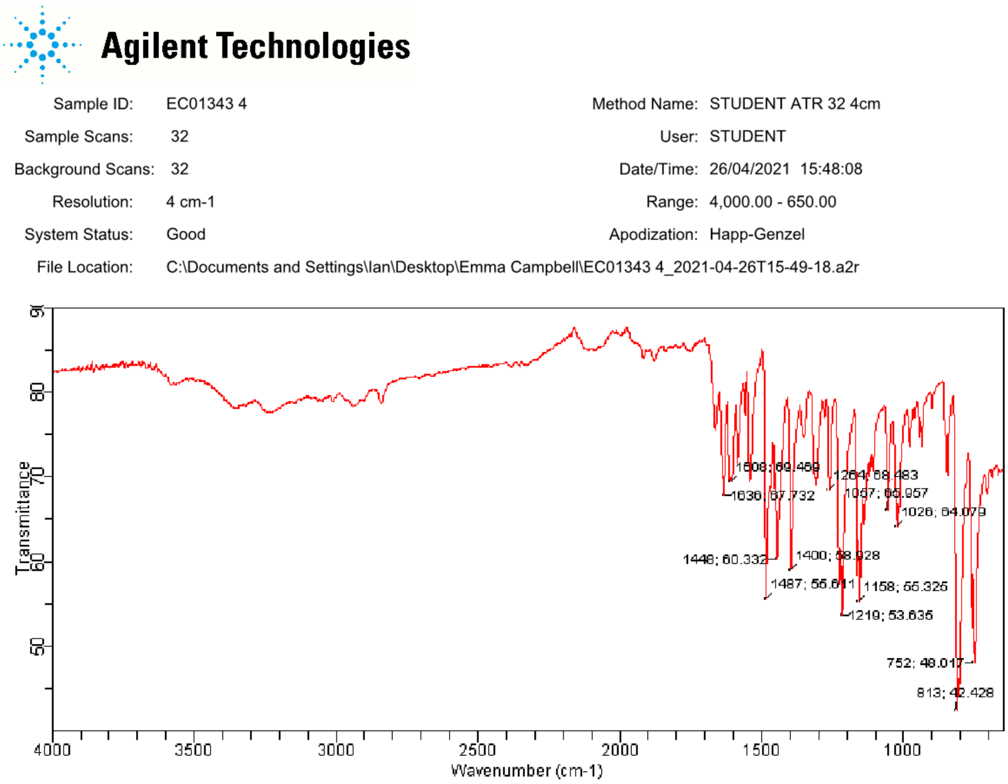

Figure S135. FT-IR spectrum of 10-methoxybenzo[4,5]imidazo[1,2-*a*]quinolone (**9k**).

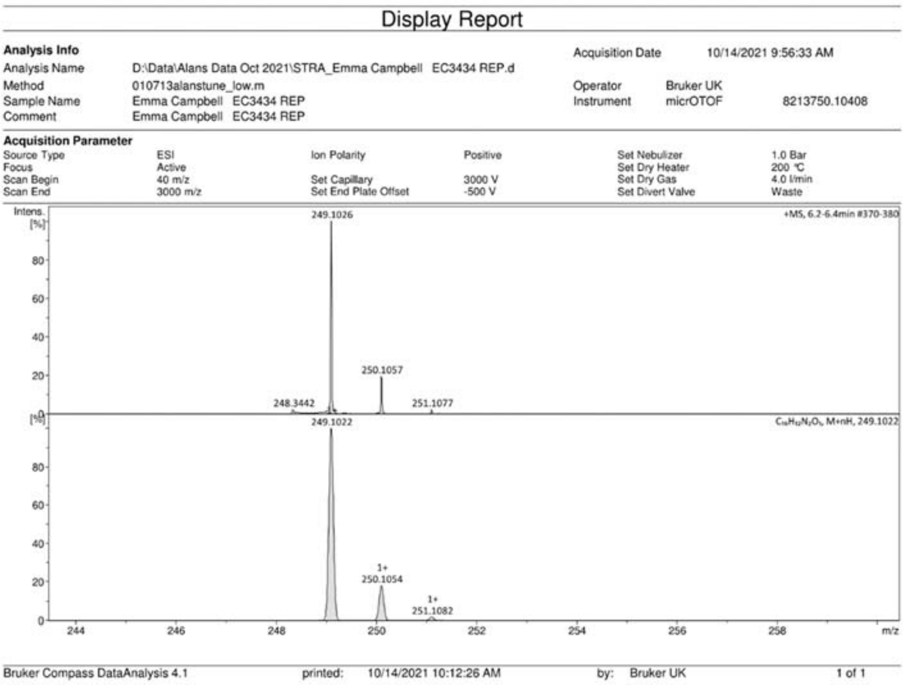

Figure S136. HRMS analysis of 10-methoxybenzo[4,5]imidazo[1,2-*a*]quinolone (**9k**).

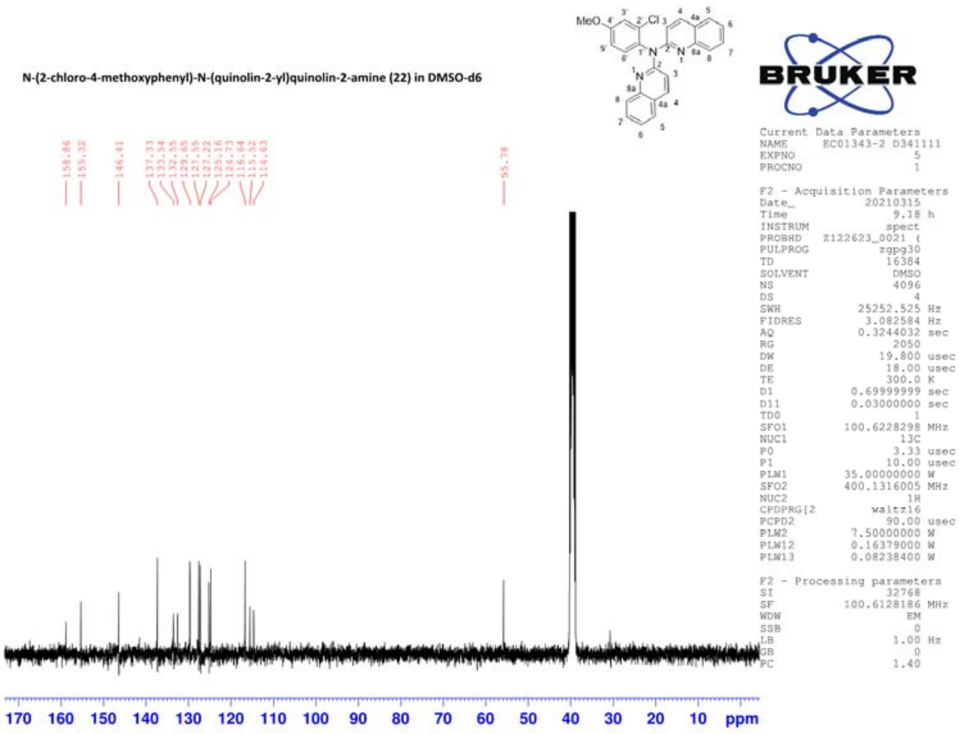

Figure S137. $^{13}C\{^{1}H\}$ NMR spectrum of N-(2-chloro-4-methoxyphenyl)-N-(quinolin-2-yl)quinolin-2-amine (**22**).

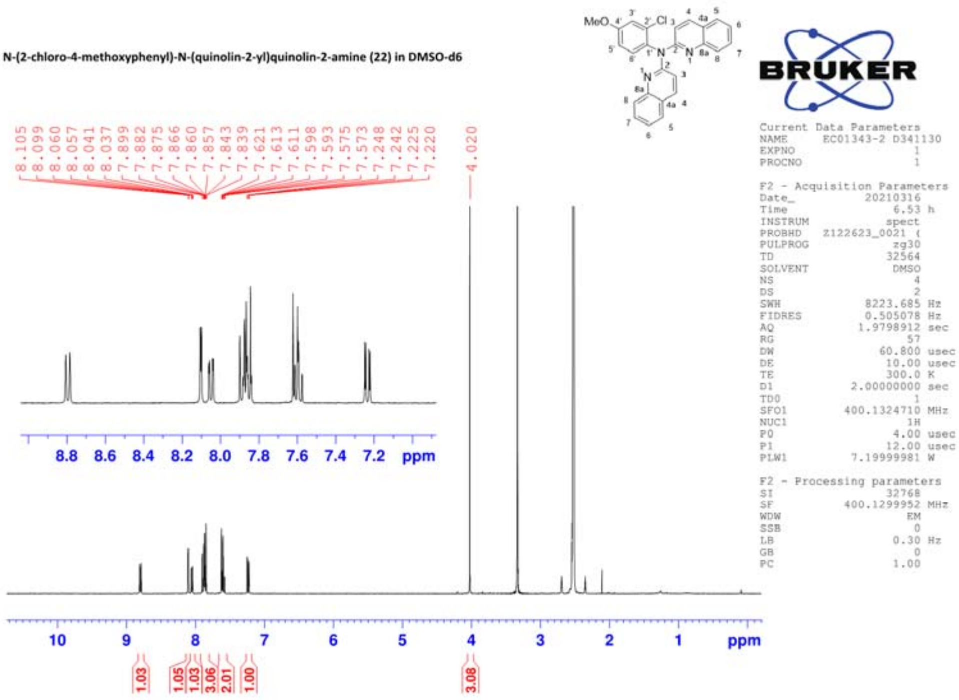

Figure S138. ^{1}H NMR spectrum of N-(2-chloro-4-methoxyphenyl)-N-(quinolin-2-yl)quinolin-2-amine (**22**).

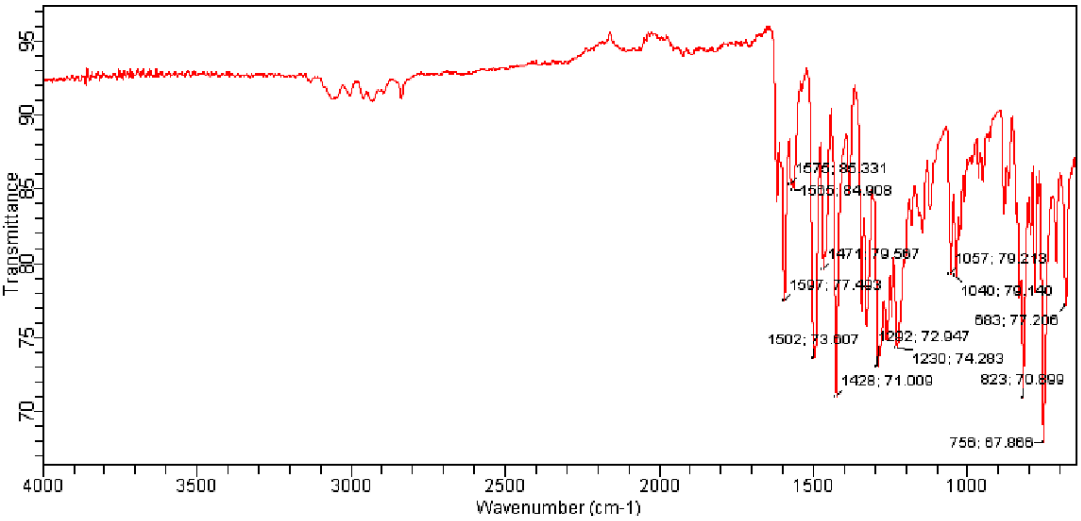

Figure S139. FT-IR spectrum of N-(2-chloro-4-methoxyphenyl)-N-(quinolin-2-yl)quinolin-2-amine (**22**).

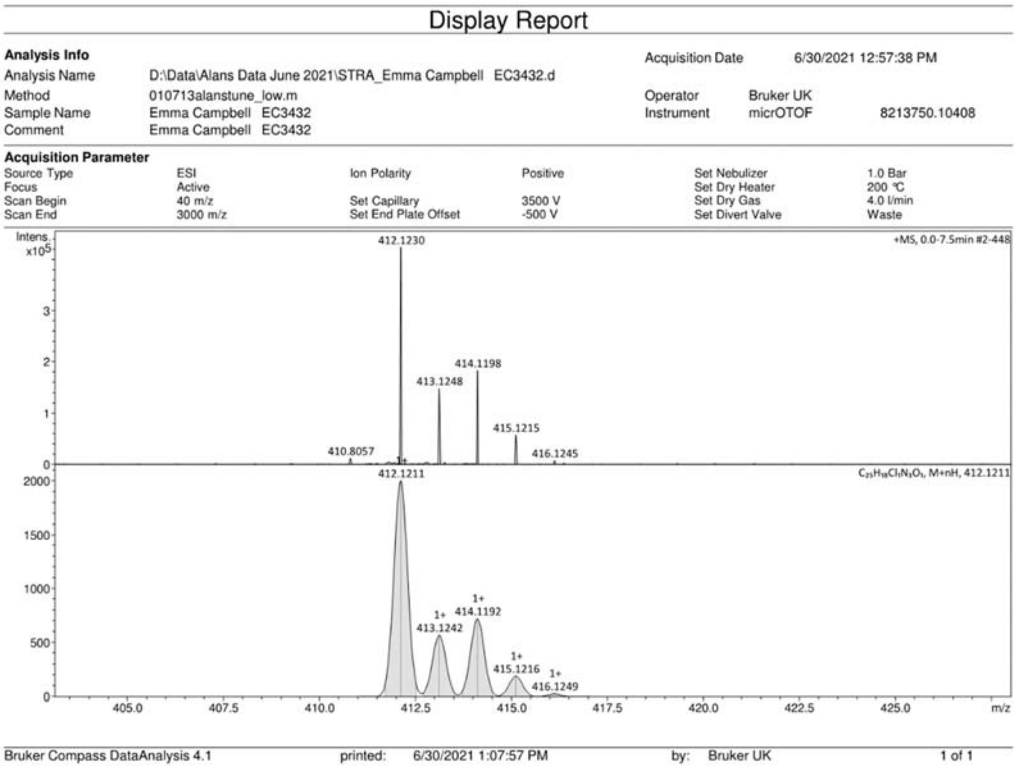

Figure S140. HRMS analysis of N-(2-chloro-4-methoxyphenyl)-N-(quinolin-2-yl)quinolin-2-amine (**22**).

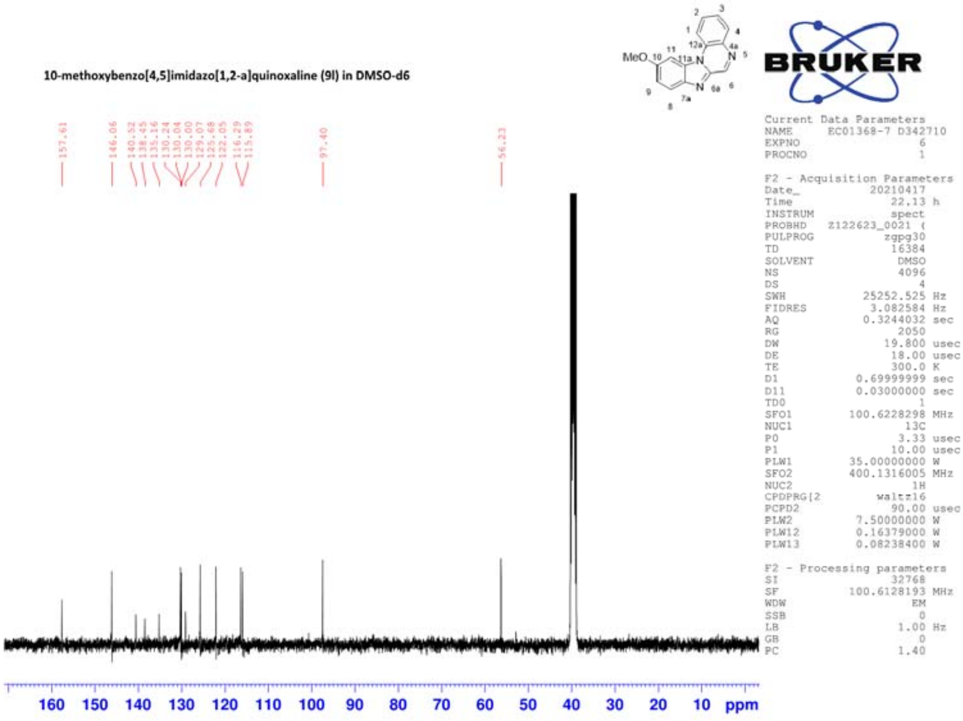

Figure S141. $^{13}C\{^{1}H\}$ NMR spectrum of 10-methoxybenzo[4,5]imidazo[1,2-a]quinoxaline (**9l**).

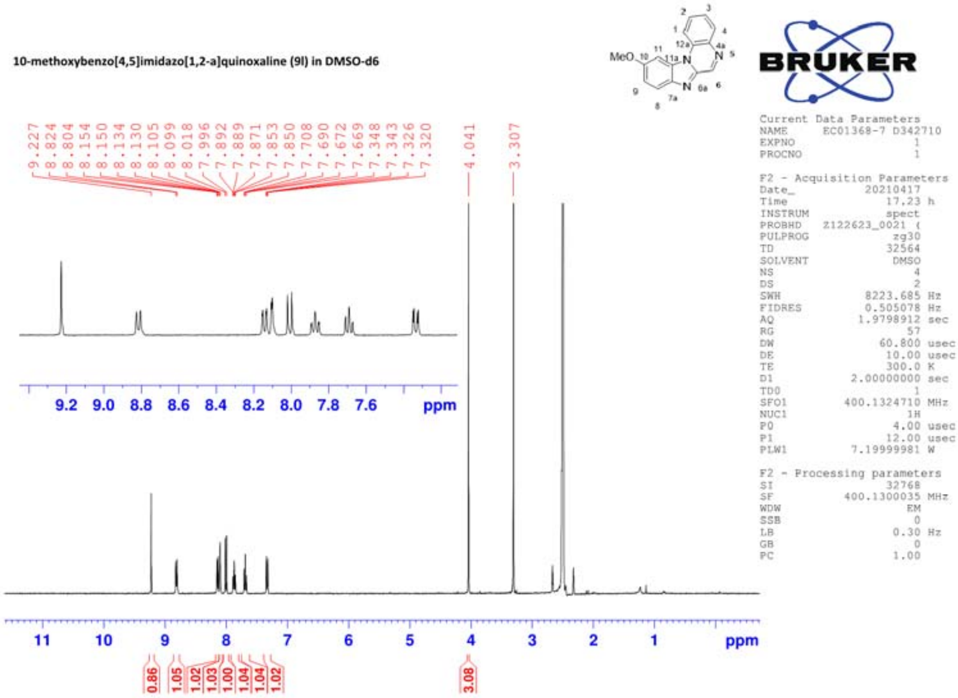

Figure S142. ^{1}H NMR spectrum of 10-methoxybenzo[4,5]imidazo[1,2-*a*]quinoxaline (**9l**).

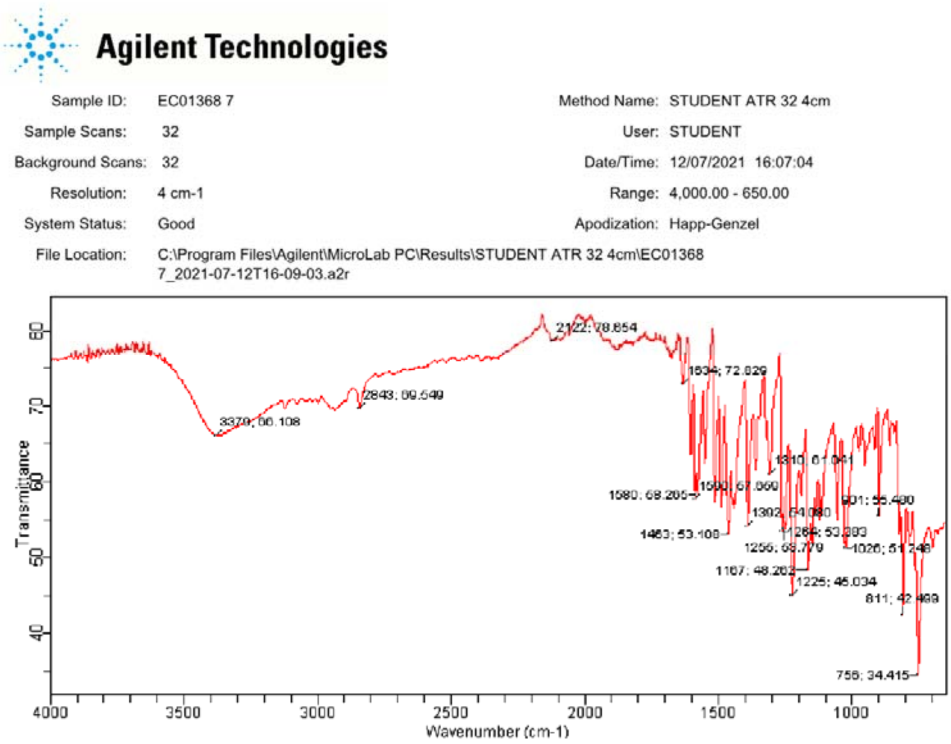

Figure S143. FT-IR spectrum of 10-methoxybenzo[4,5]imidazo[1,2-*a*]quinoxaline (**9l**).

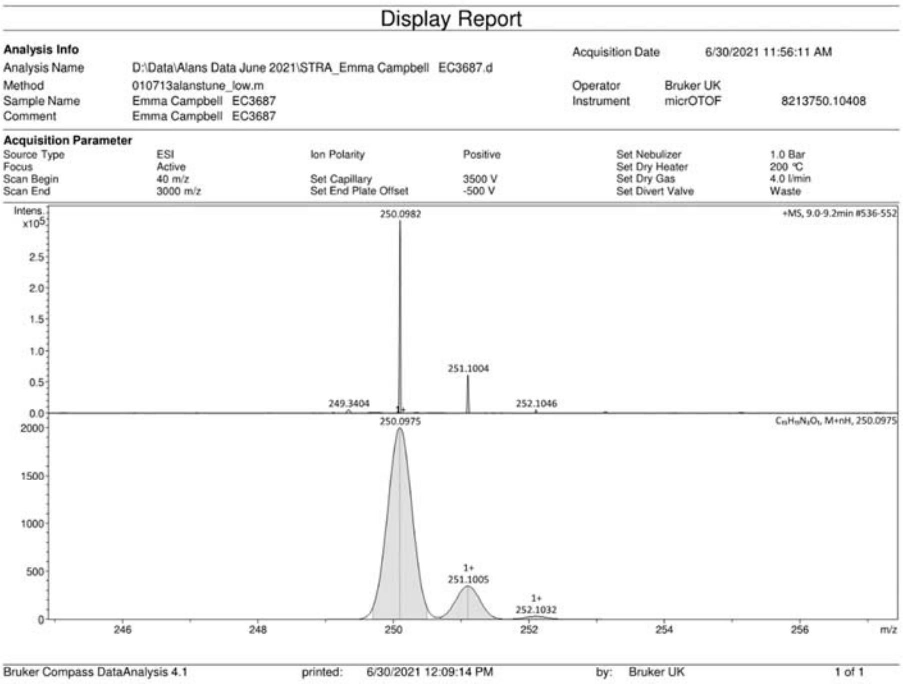

Figure S144. HRMS analysis of 10-methoxybenzo[4,5]imidazo[1,2-*a*]quinoxaline (**9l**).

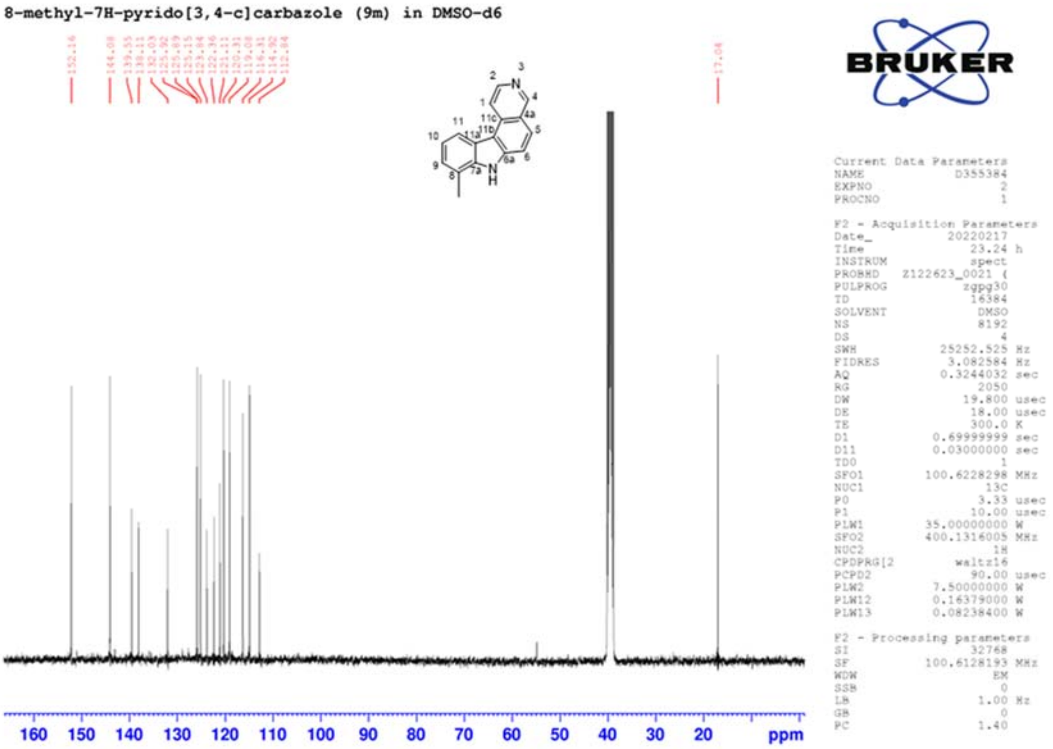

Figure S145. $^{13}C\{^1H\}$ NMR spectrum of 8-methyl-7H-pyrido[3,4-c]carbazole (**9m**).

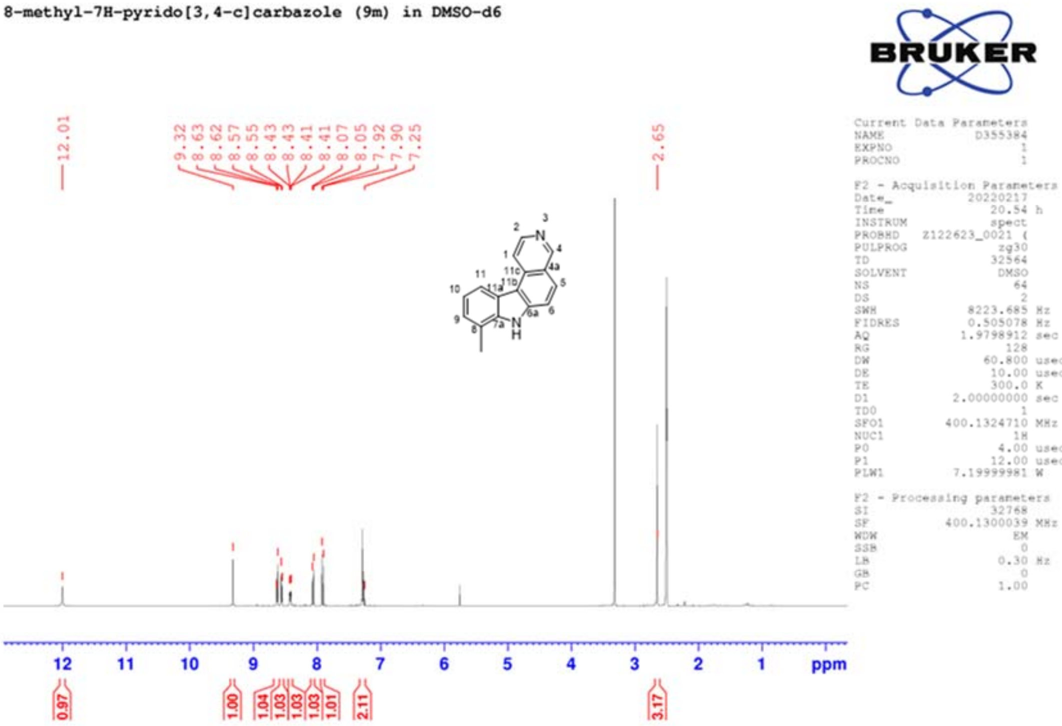

Figure S146. ^1H NMR spectrum of 8-methyl-7H-pyrido[3,4-c]carbazole (**9m**).

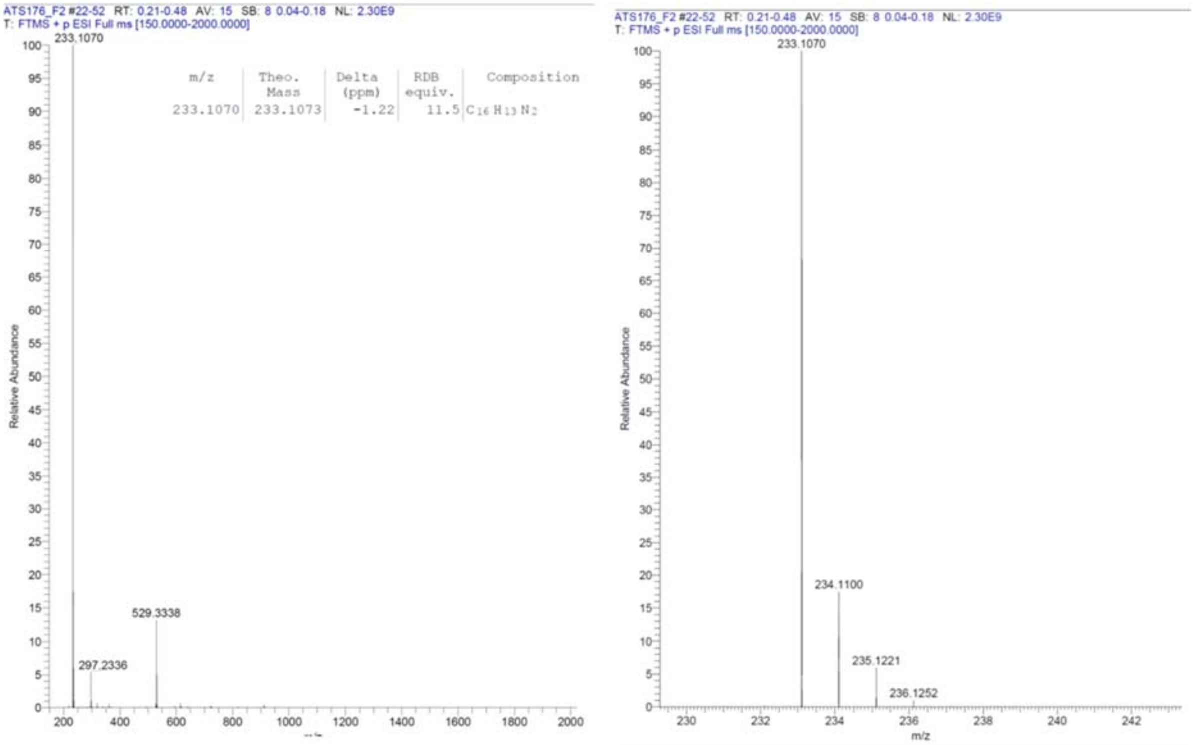

Figure S147. HRMS analysis of 8-methyl-7H-pyrido[3,4-c]carbazole (**9m**).

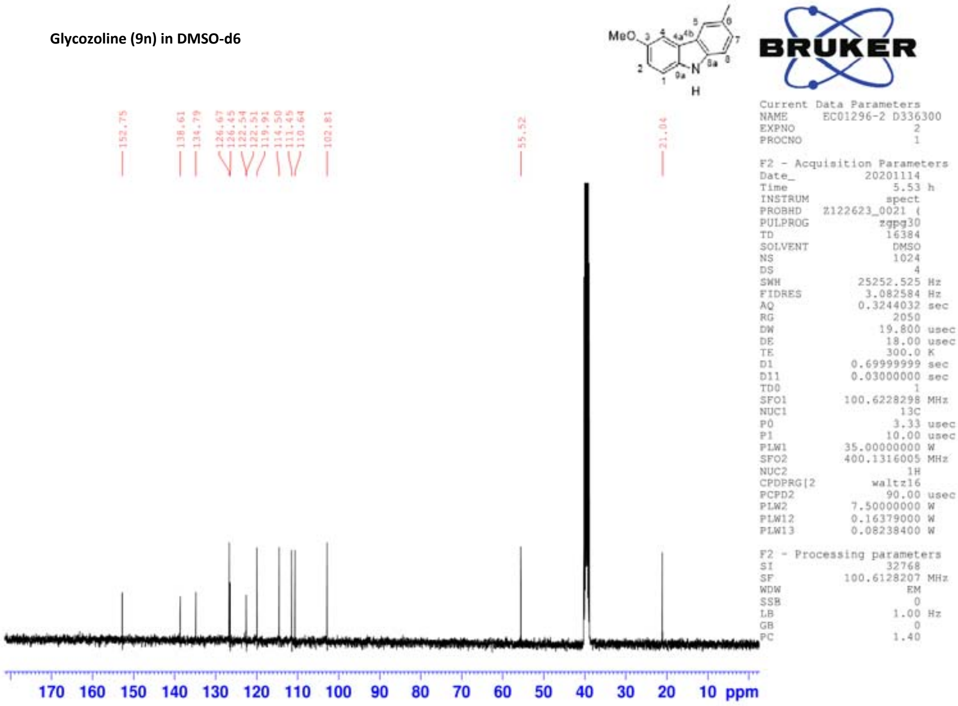

Figure S148. $^{13}C\{^{1}H\}$ NMR spectrum of Glycozoline (**9n**).

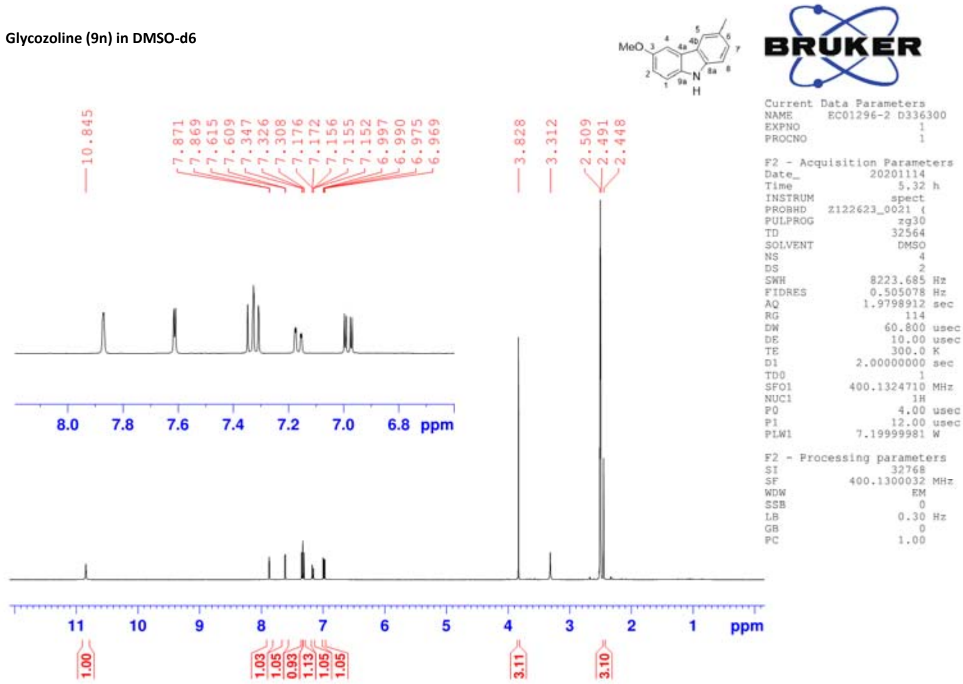

Figure S149. ^{1}H NMR spectrum of Glycozoline (**9n**).

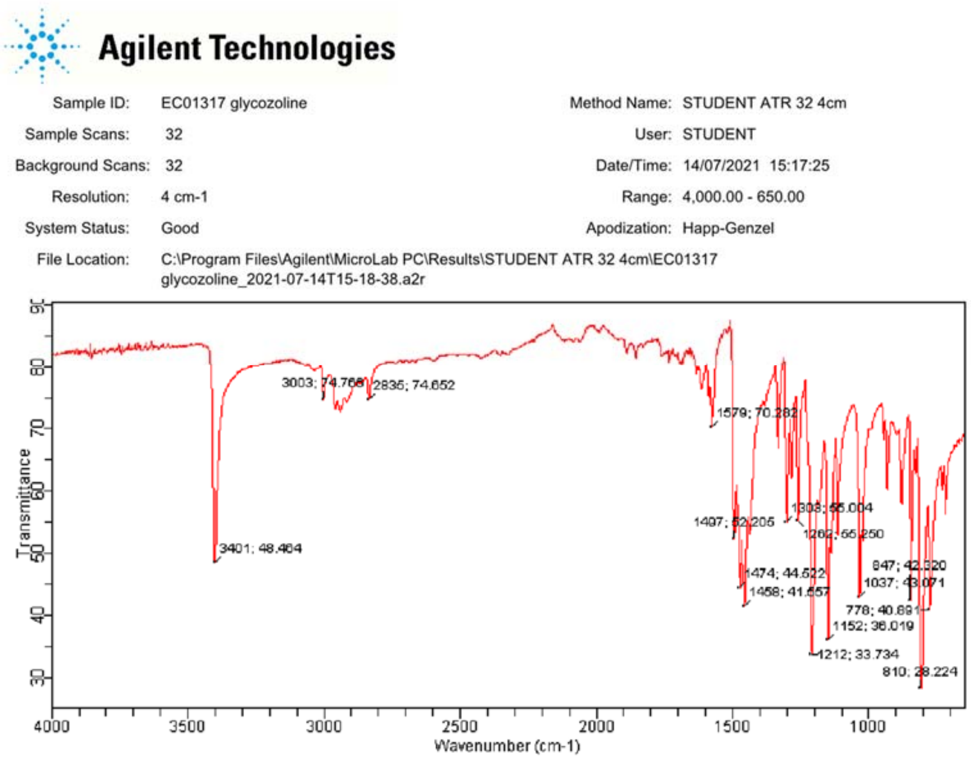

Figure S150. FT-IR spectrum of Glycozoline (**9n**).

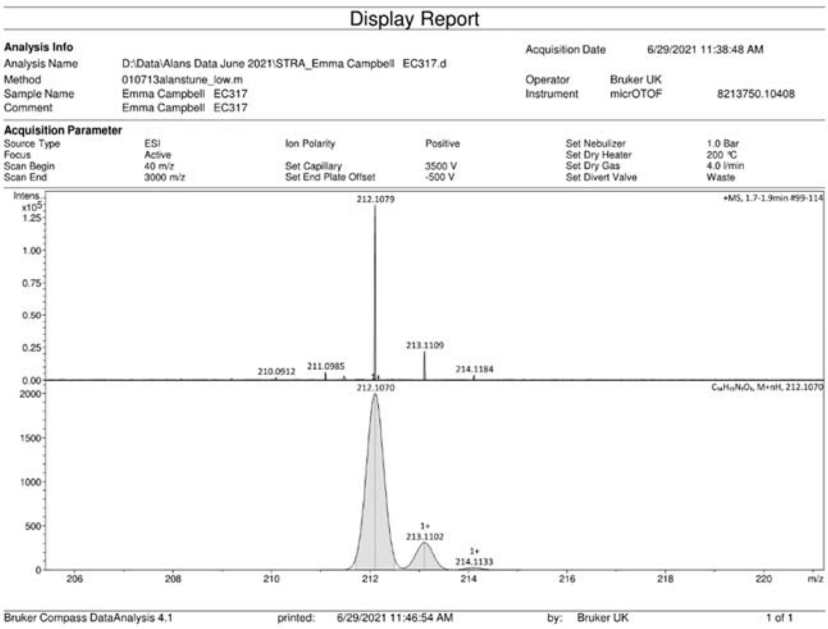

Figure S151. HRMS analysis of Glycozoline (**9n**).

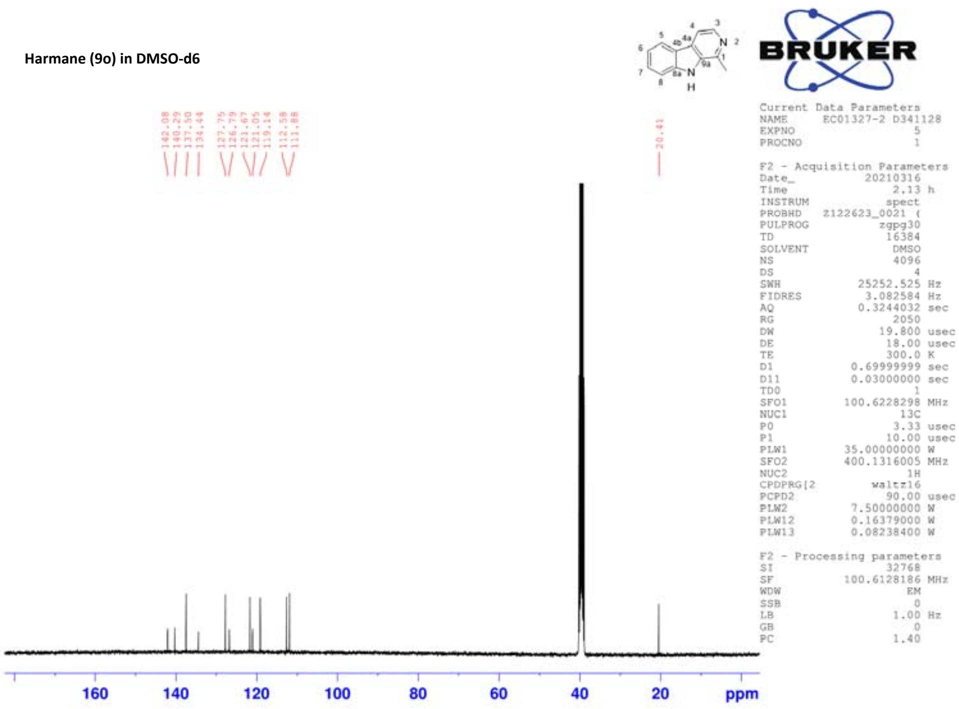

Figure S152. $^{13}C\{^{1}H\}$ NMR spectrum of Harmane (**9o**).

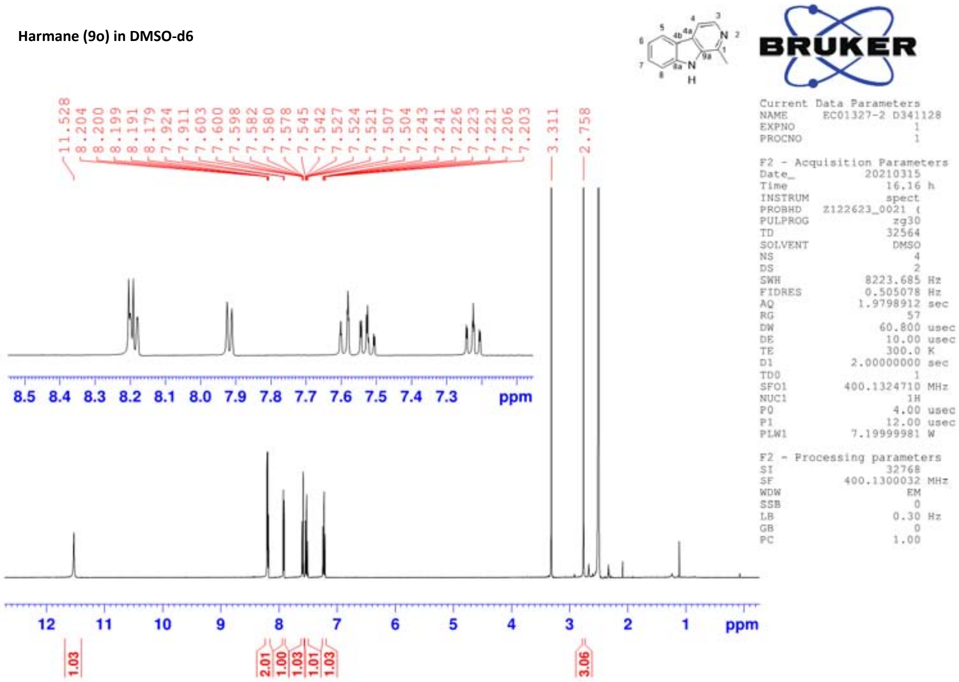

Figure S153. ^{1}H NMR spectrum of Harmane (**9o**).

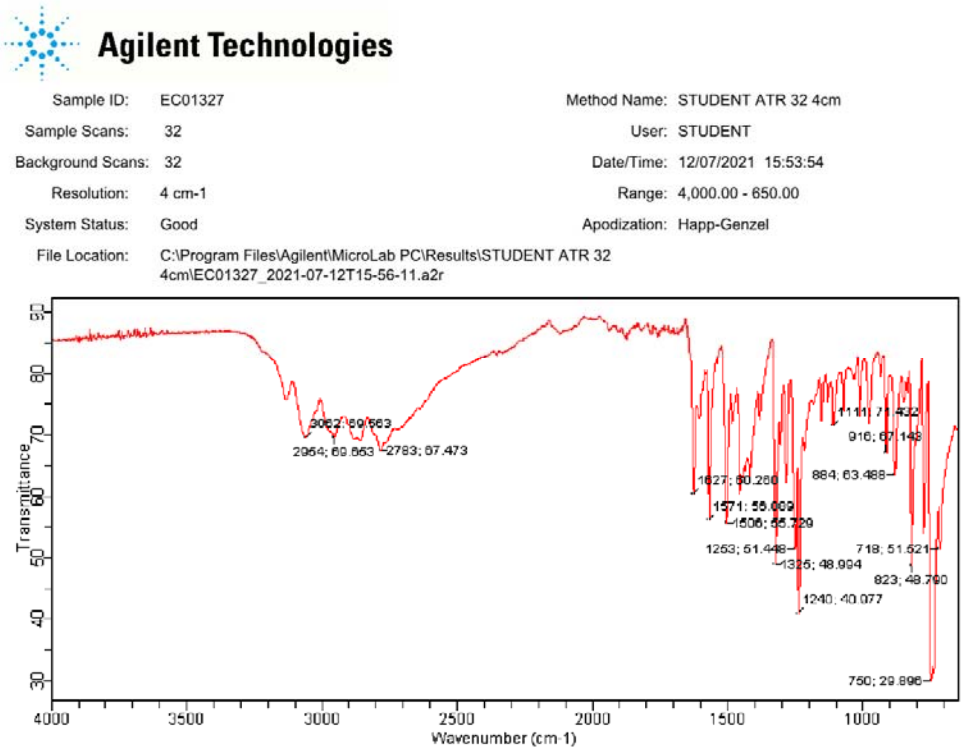

Figure S154. FT-IR spectrum of Harmane (**9o**).

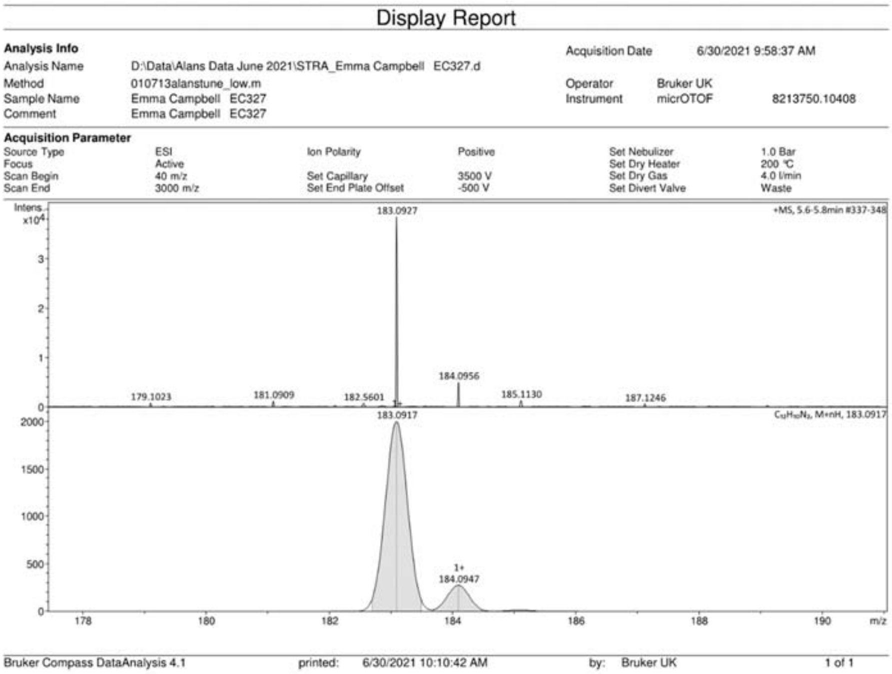

Figure S155. HRMS analysis of Harmane (**9o**).

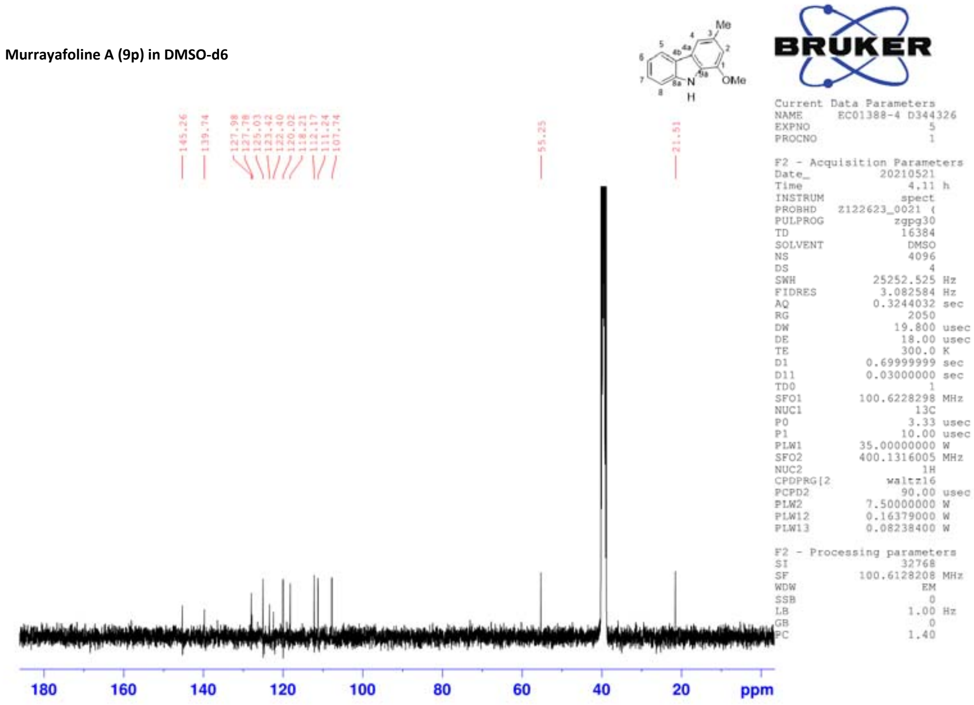

Figure S156. $^{13}C\{^{1}H\}$ NMR spectrum of Murrayafoline A (**9p**).

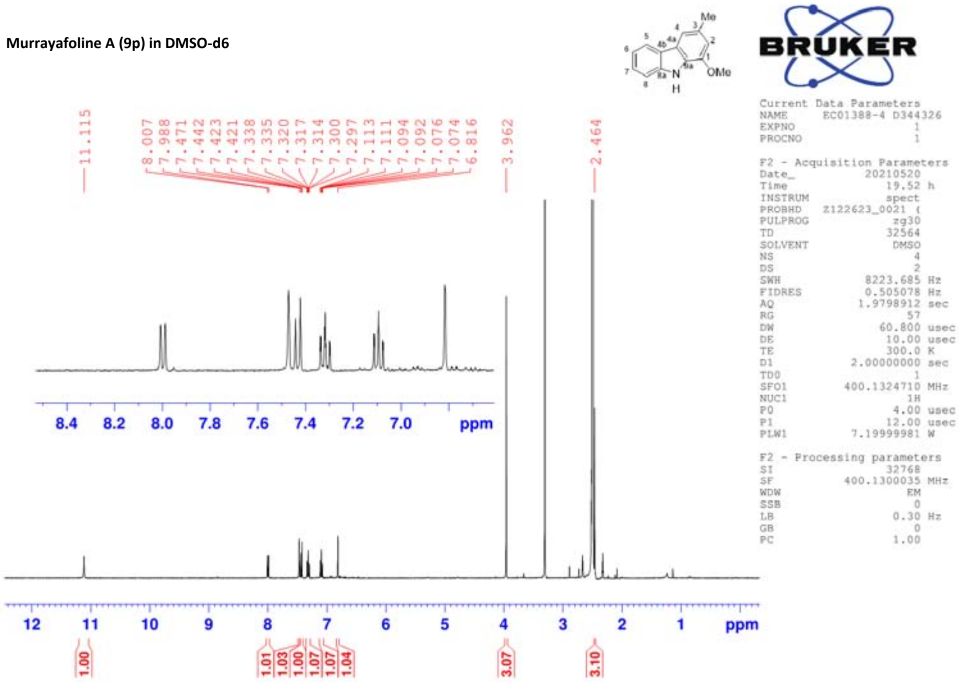

Figure S157. ^{1}H NMR spectrum of Murrayafoline A (**9p**).

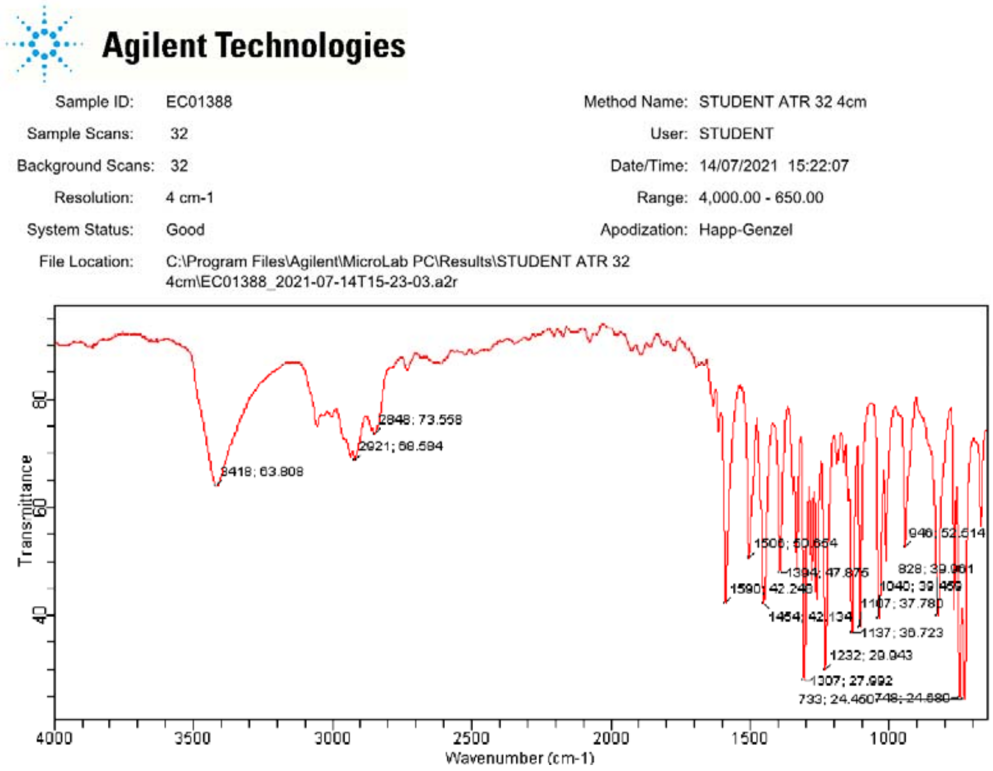

Figure S158. FT-IR spectrum of Murrayafoline A (**9p**).

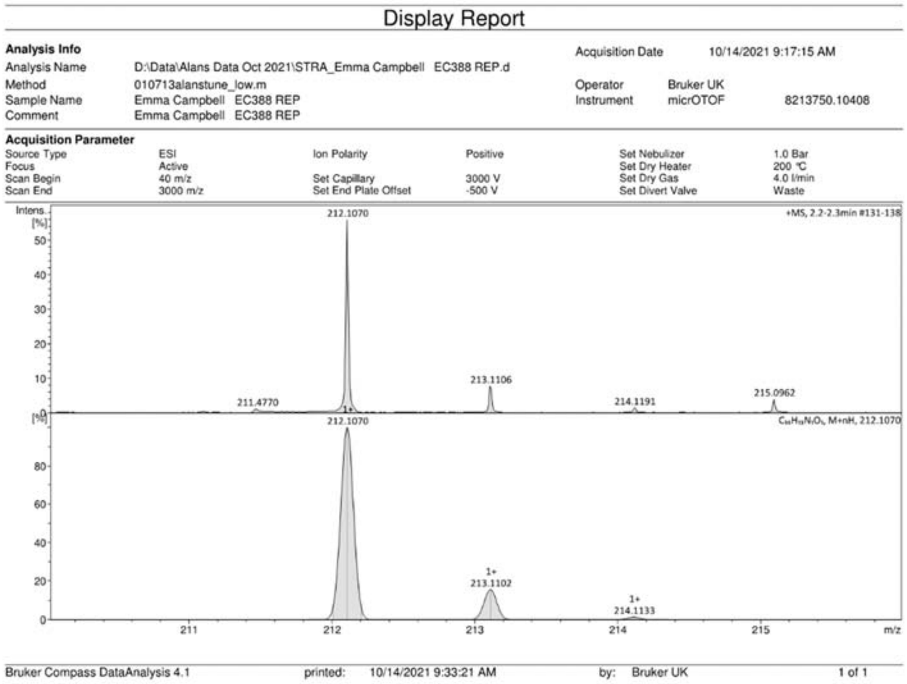

Figure S159. HRMS analysis of Murrayafoline A (**9p**).

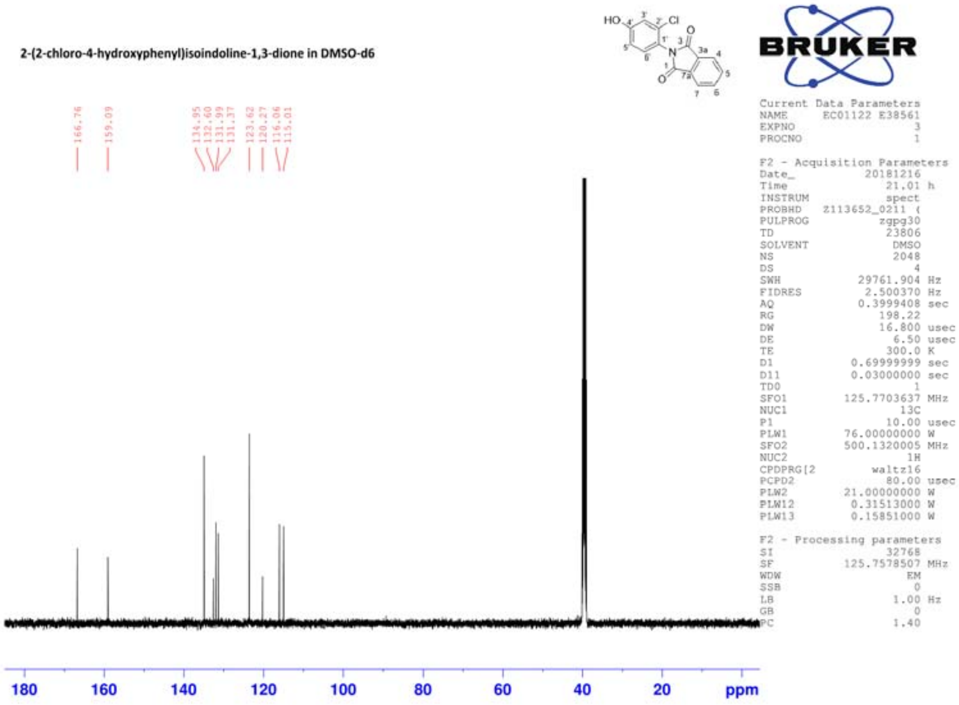

Figure S160. $^{13}C\{^{1}H\}$ NMR spectrum of 2-(2-chloro-4-hydroxyphenyl)isoindoline-1,3-dione.

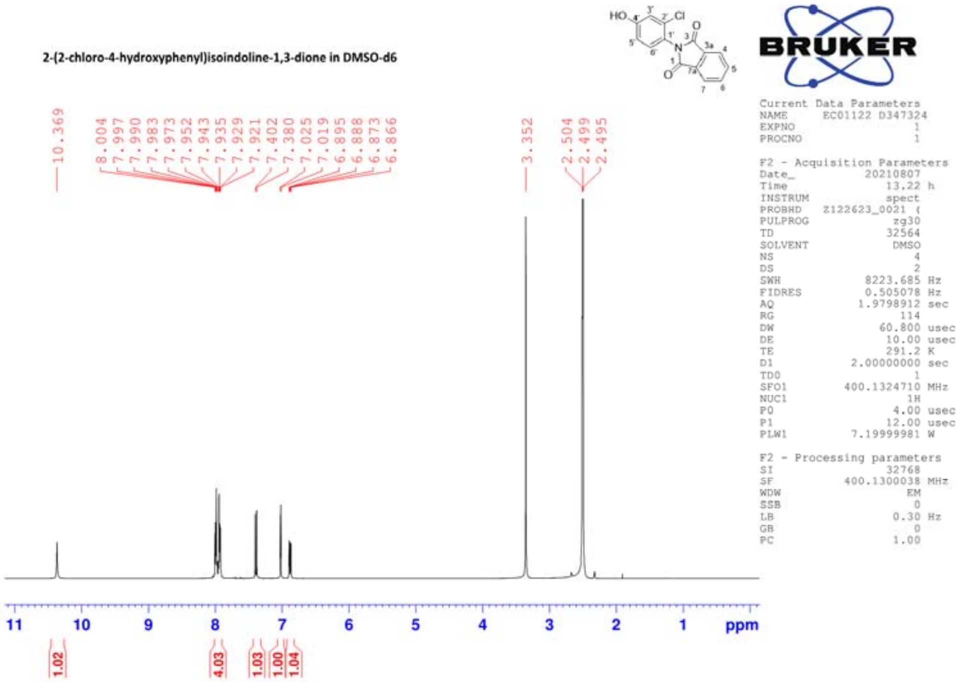

Figure S161. ^{1}H NMR spectrum of 2-(2-chloro-4-hydroxyphenyl)isoindoline-1,3-dione.

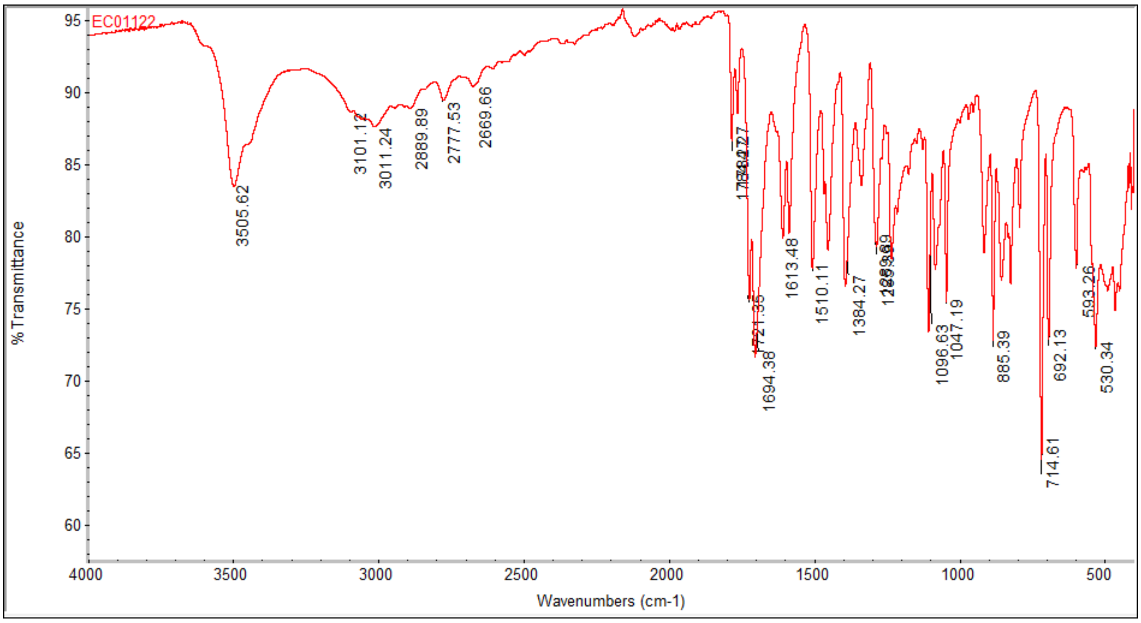

Figure S162. FT-IR spectrum of 2-(2-chloro-4-hydroxyphenyl)isoindoline-1,3-dione.

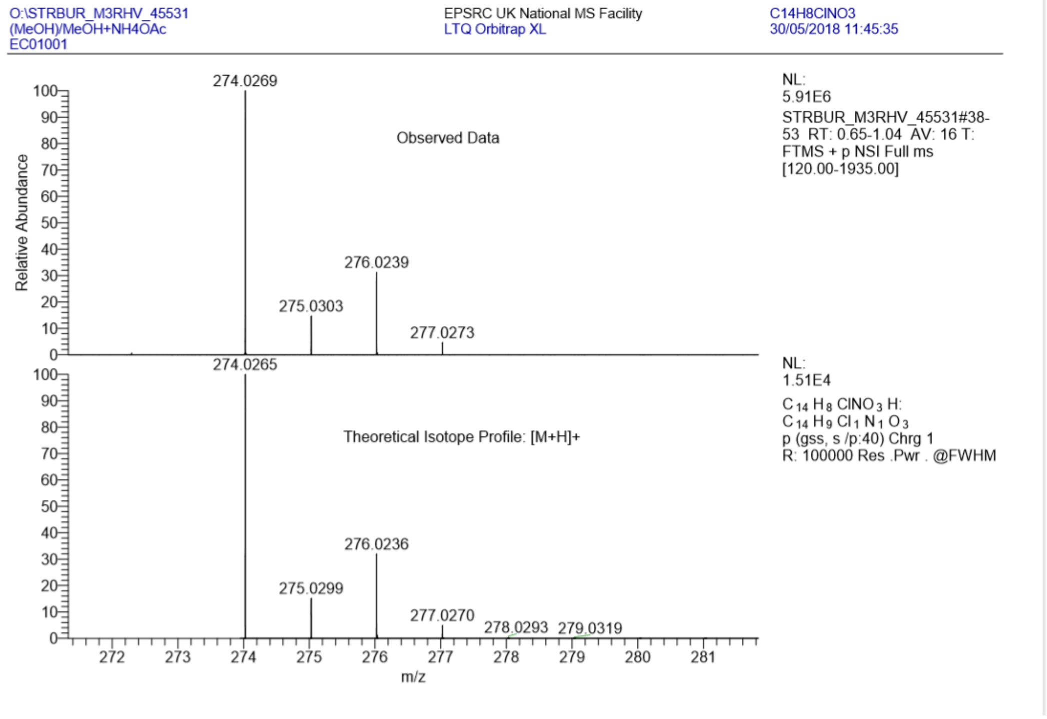

Figure S163. HRMS analysis of 2-(2-chloro-4-hydroxyphenyl)isoindoline-1,3-dione.

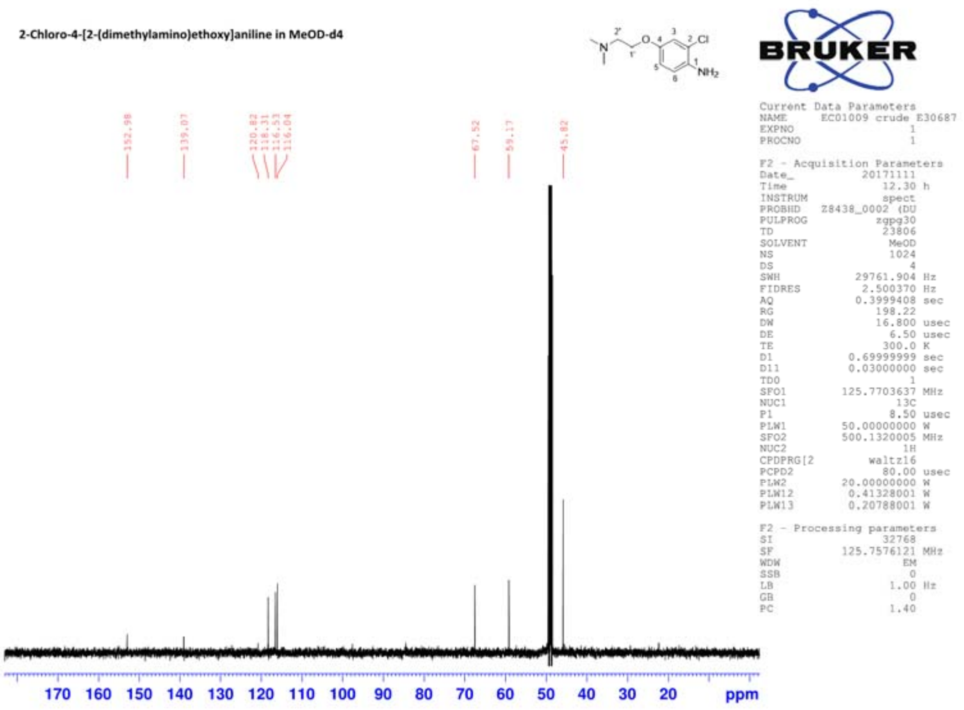

Figure S164. $^{13}C\{^{1}H\}$ NMR spectrum of 2-chloro-4-[2-(dimethylamino)ethoxy]aniline.

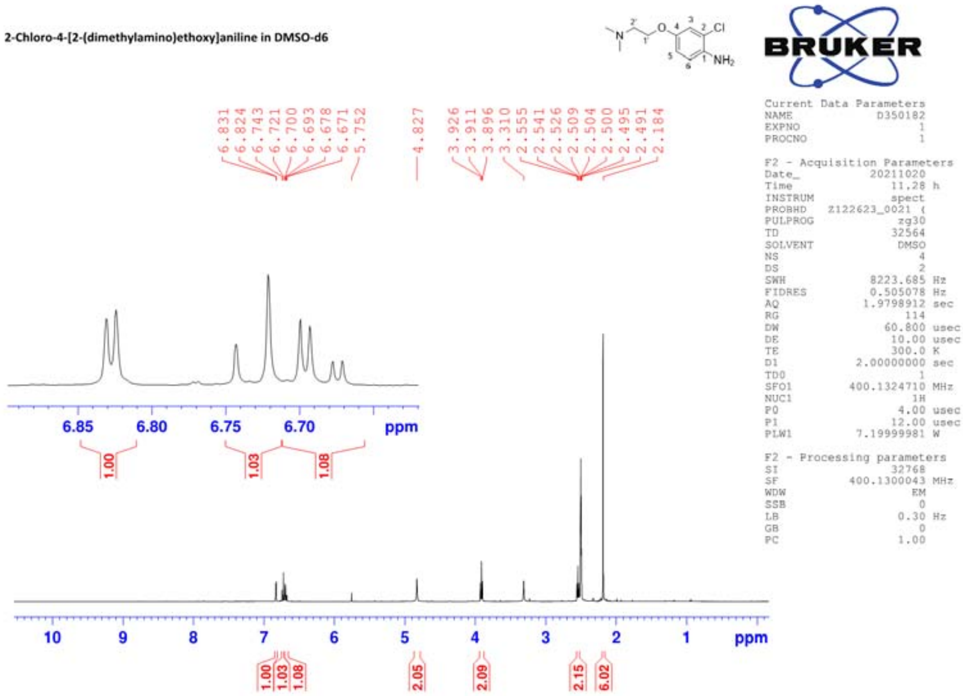

Figure S165. ^{1}H NMR spectrum of 2-chloro-4-[2-(dimethylamino)ethoxy]aniline.

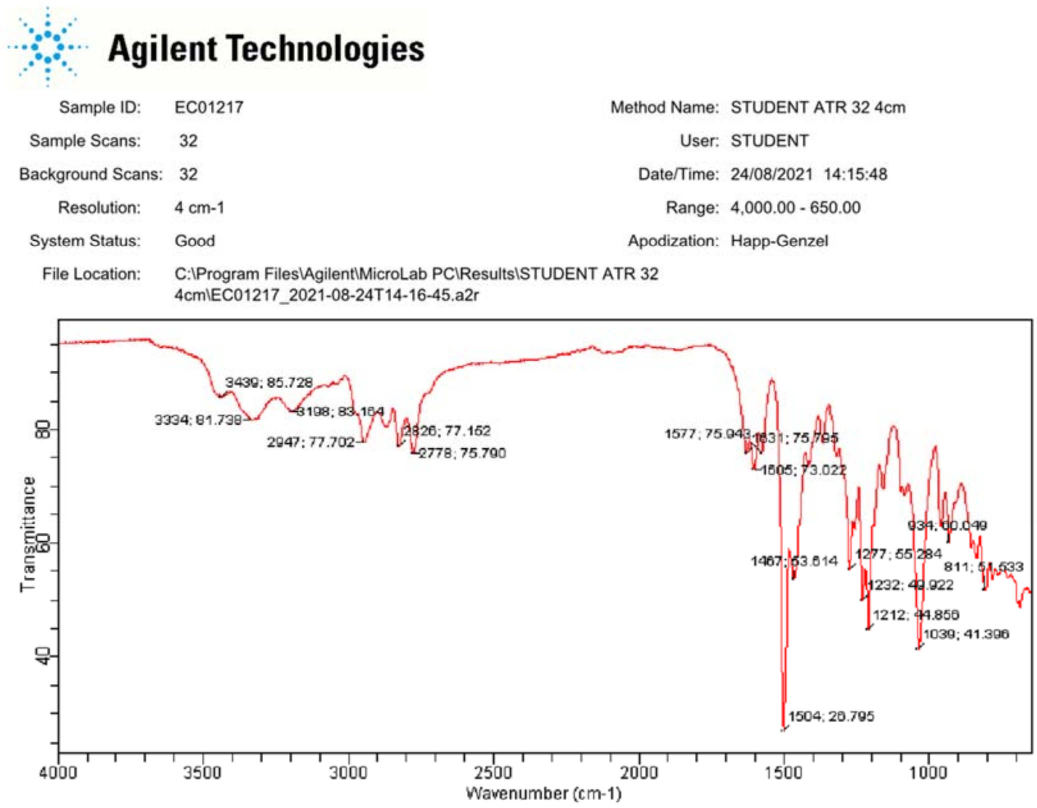

Figure S166. FT-IR spectrum of 2-chloro-4-[2-(dimethylamino)ethoxy]aniline.

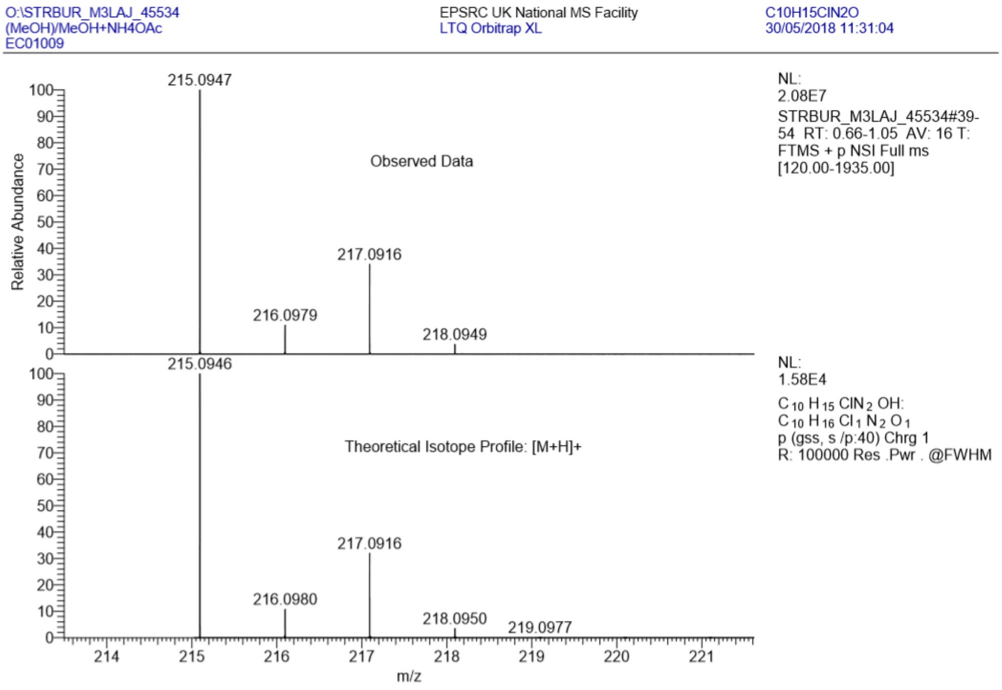

Figure S167. HRMS analysis of 2-chloro-4-[2-(dimethylamino)ethoxy]aniline.

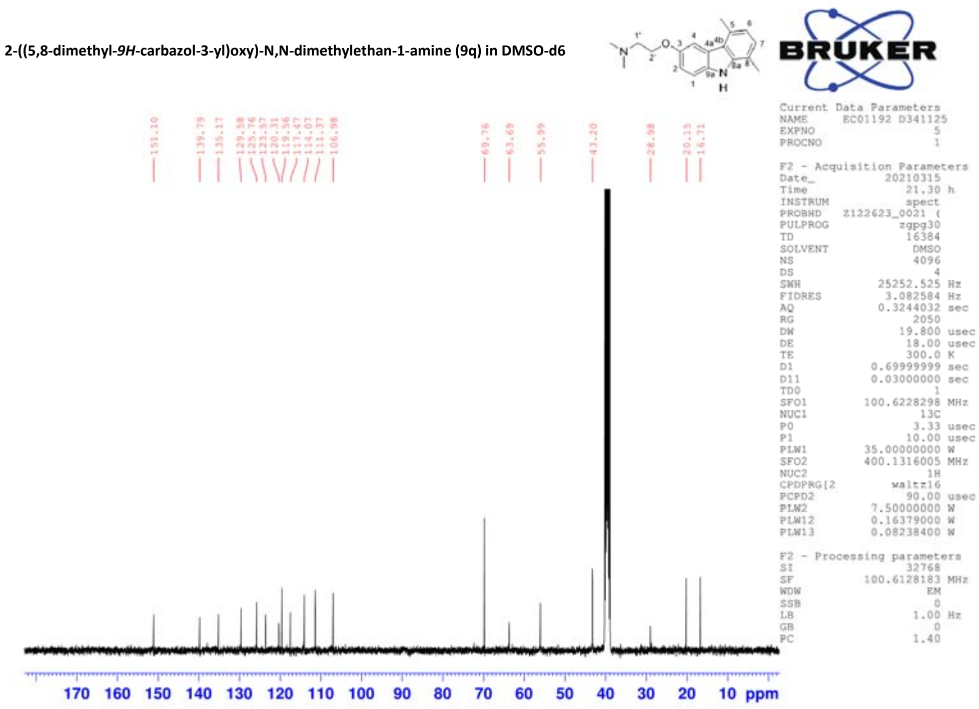

Figure S168. $^{13}C\{^{1}H\}$ NMR spectrum of 2-((5,8-dimethyl-*9H*-carbazol-3-yl)oxy)-N,N-dimethylethan-1-amine (**9q**).

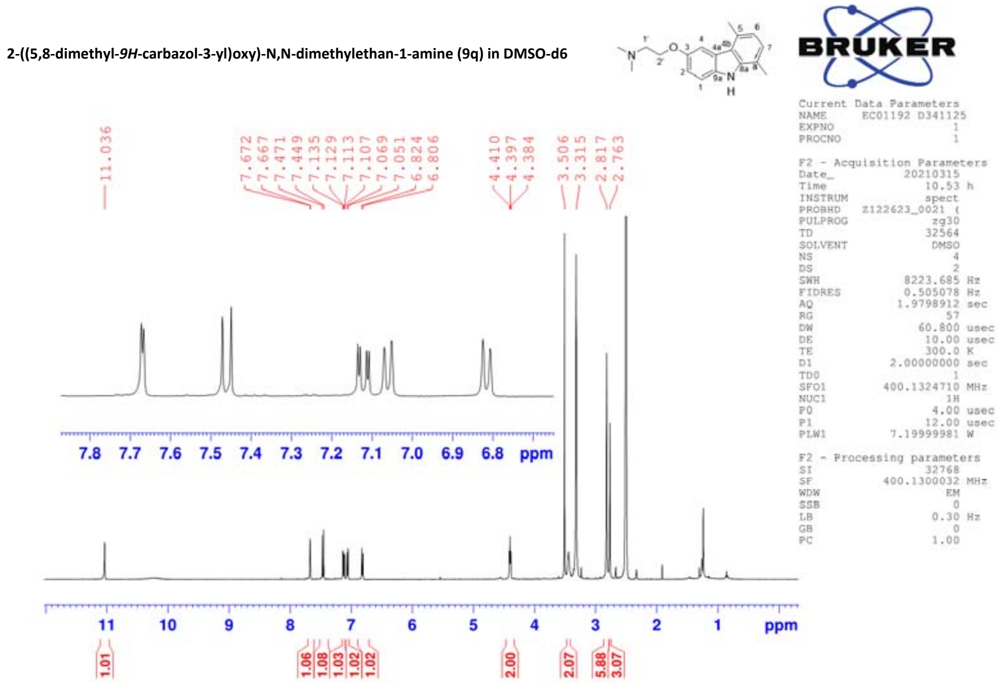

Figure S169. ^{1}H NMR spectrum of 2-((5,8-dimethyl-*9H*-carbazol-3-yl)oxy)-N,N-dimethylethan-1-amine (**9q**).

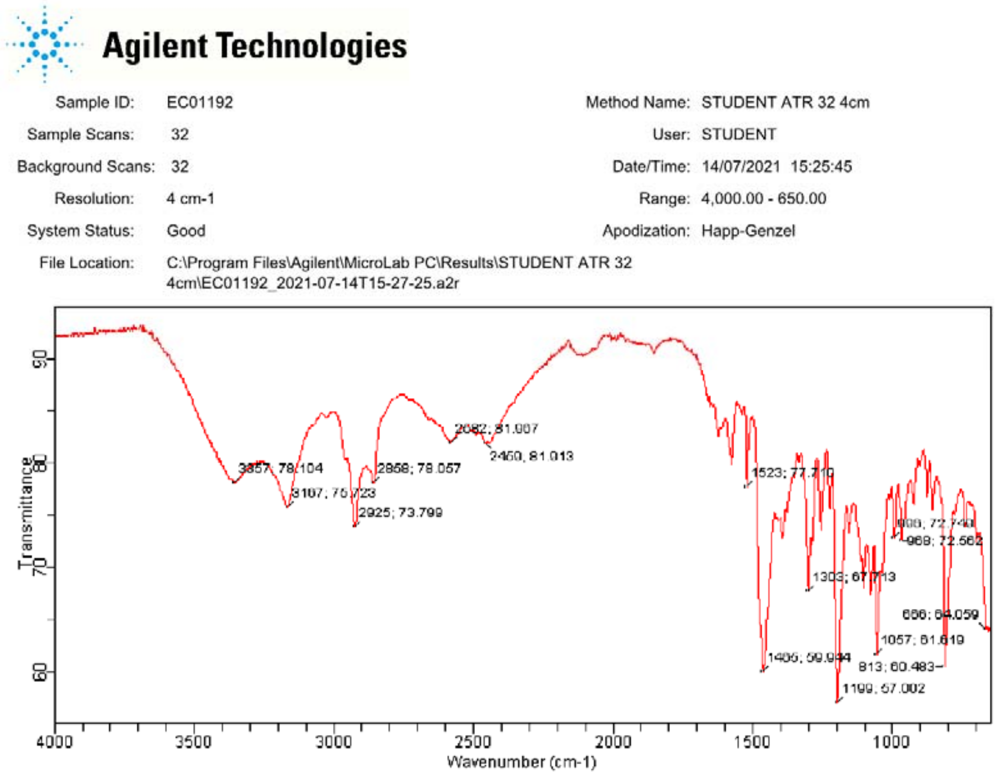

Figure S170. FT-IR spectrum of 2-((5,8-dimethyl-*9H*-carbazol-3-yl)oxy)-N,N-dimethylethan-1-amine(**9q**).

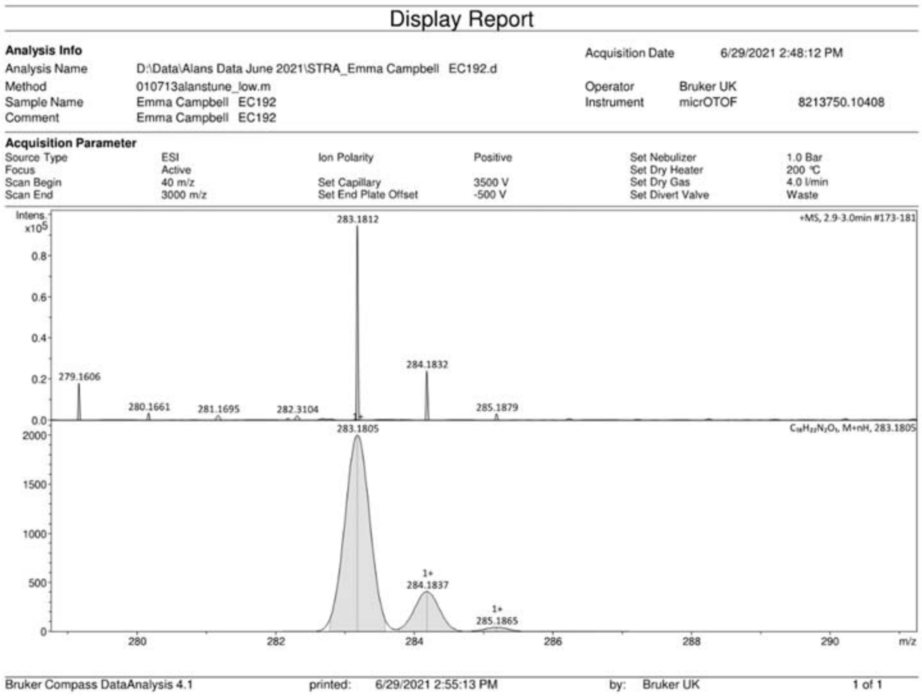

Figure S171. HRMS analysis of 2-((5,8-dimethyl-*9H*-carbazol-3-yl)oxy)-N,N-dimethylethan-1-amine (**9q**).

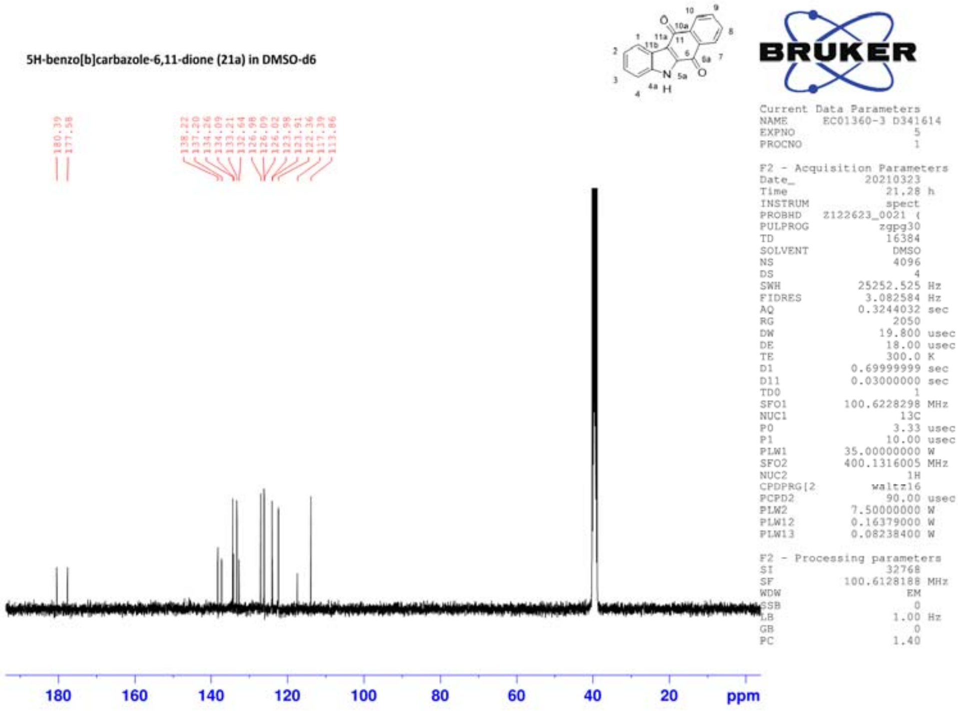

Figure S172. $^{13}C\{^{1}H\}$ NMR spectrum of *5H*-benzo[*b*]carbazole-6,11-dione (**21a**).

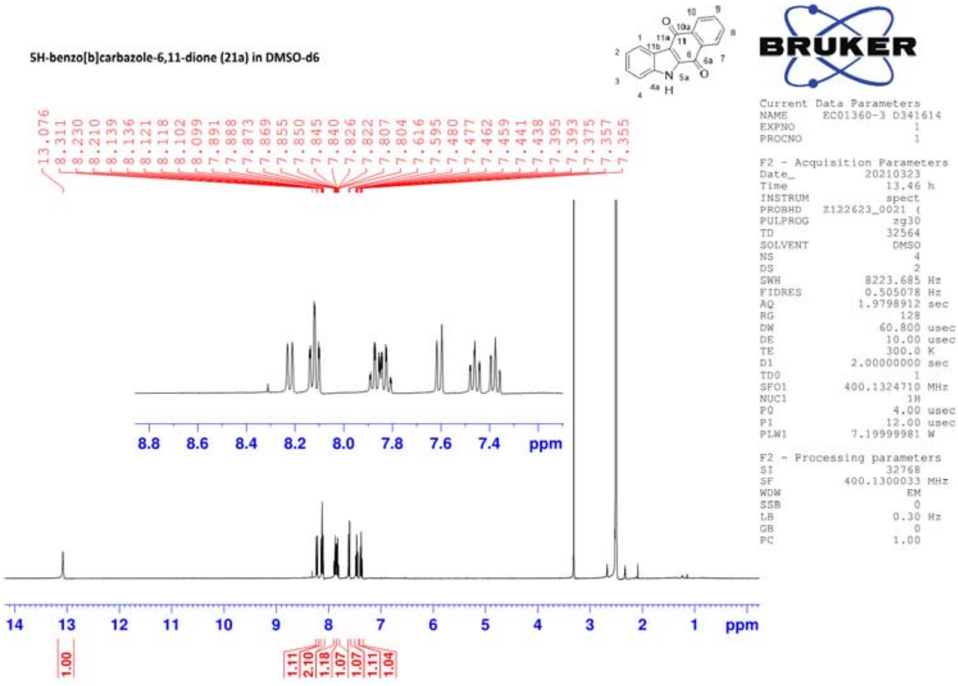

Figure S173. ^{1}H NMR spectrum of *5H*-benzo[*b*]carbazole-6,11-dione (**21a**).

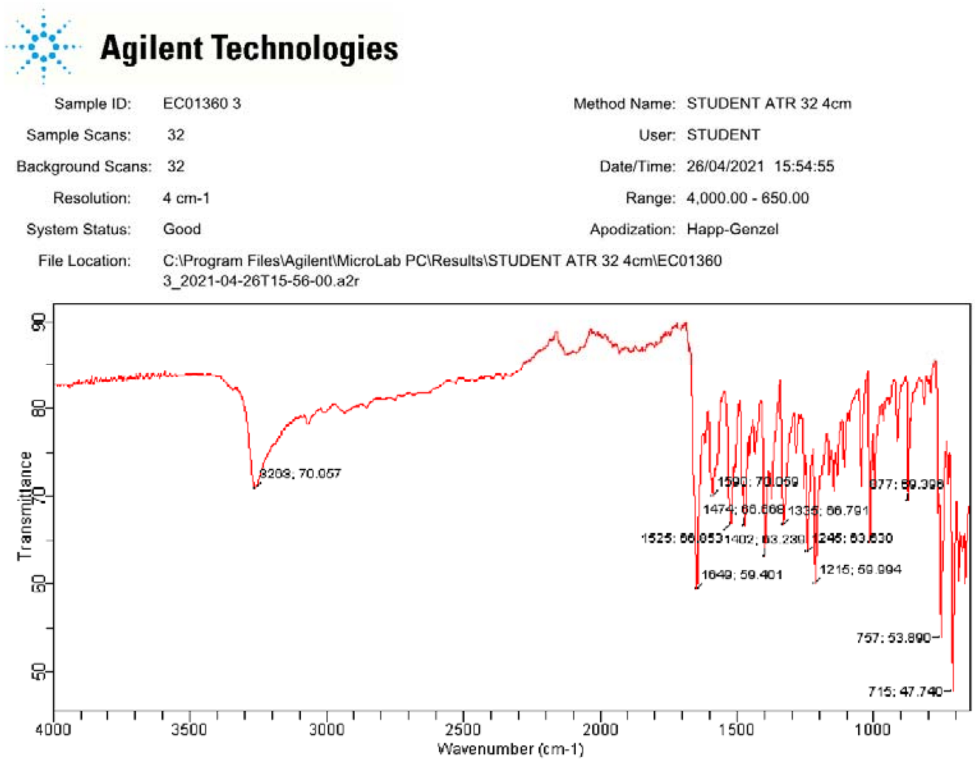

Figure S174. FT-IR spectrum of *5H*-benzo[*b*]carbazole-6,11-dione (**21a**).

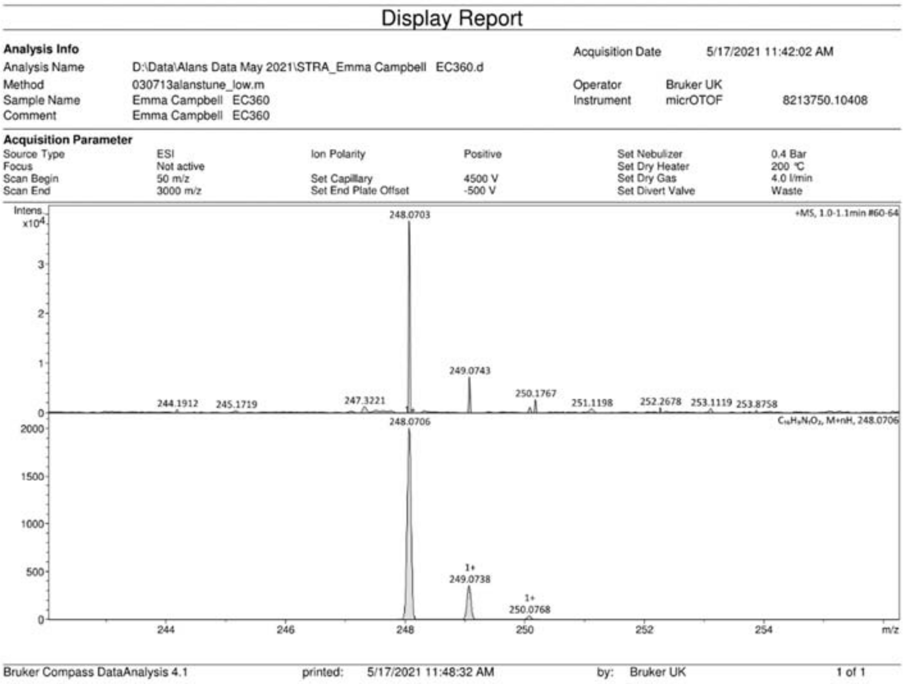

Figure S175. HRMS analysis of *5H*-benzo[*b*]carbazole-6,11-dione (**21a**).

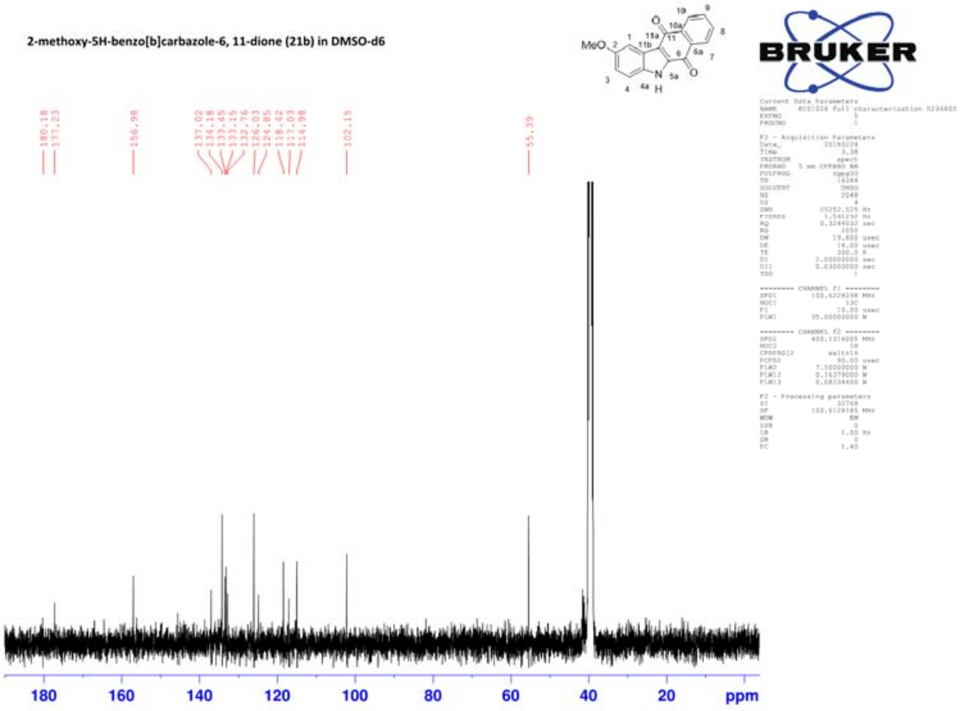

Figure S176. $^{13}C\{^{1}H\}$ NMR spectrum of 2-methoxy-*5H*-benzo[*b*]carbazole-6, 11-dione (**21b**).

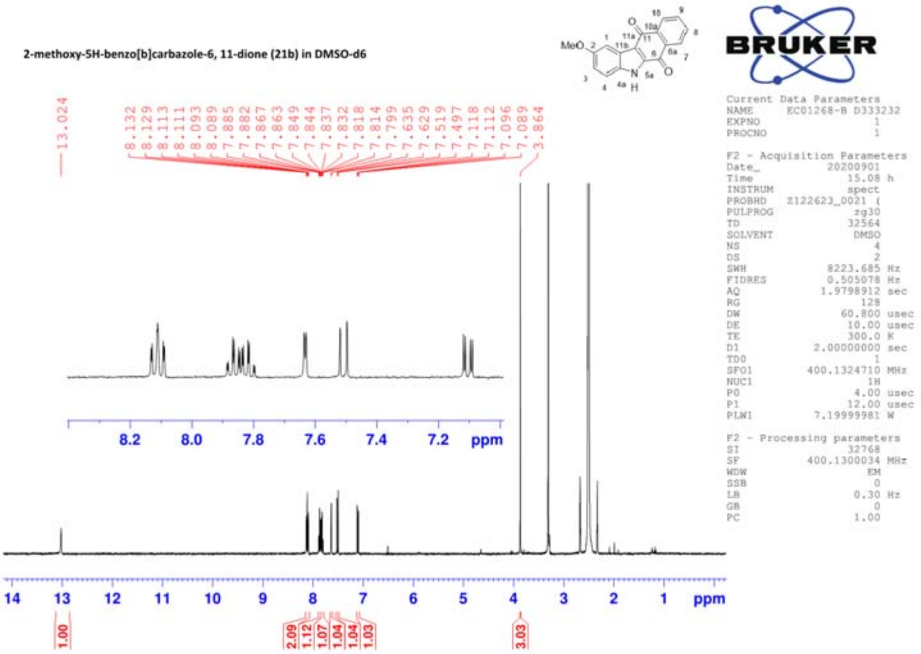

Figure S177. ^{1}H NMR spectrum of 2-methoxy-*5H*-benzo[*b*]carbazole-6, 11-dione (**21b**).

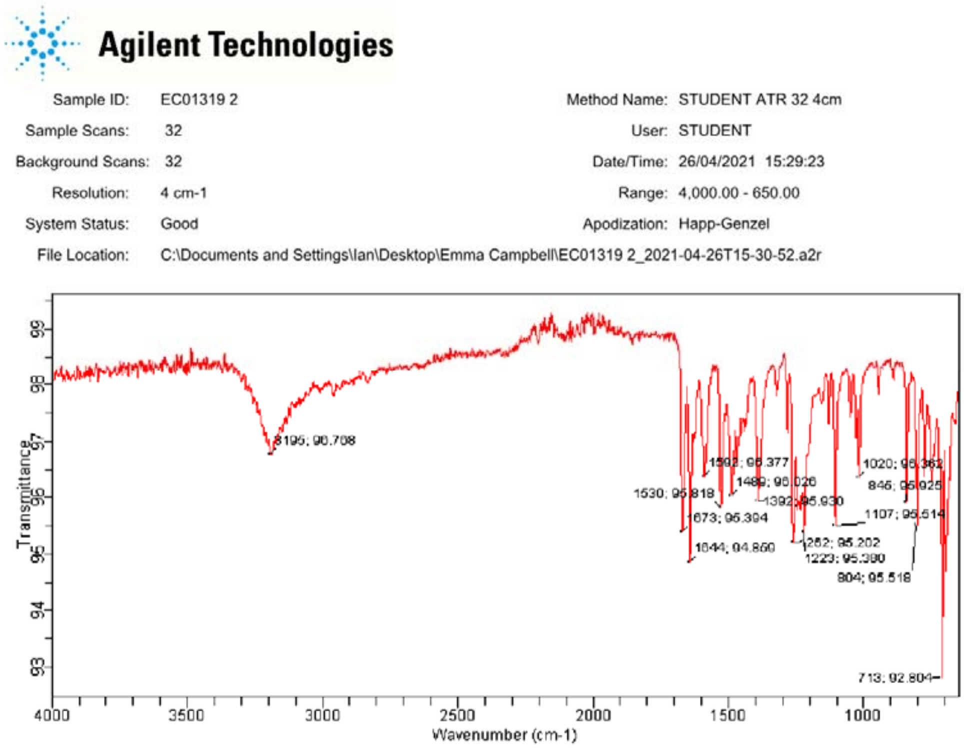

Figure S178. FT-IR spectrum of 2-methoxy-*5H*-benzo[*b*]carbazole-6, 11-dione (**21b**).

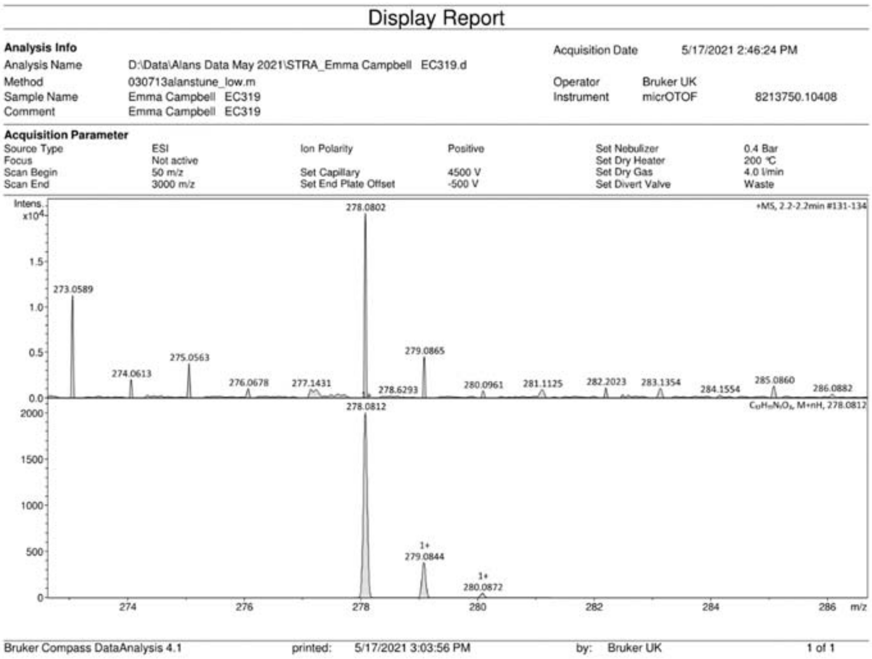

Figure S179. HRMS analysis of 2-methoxy-*5H*-benzo[*b*]carbazole-6, 11-dione (**21b**).

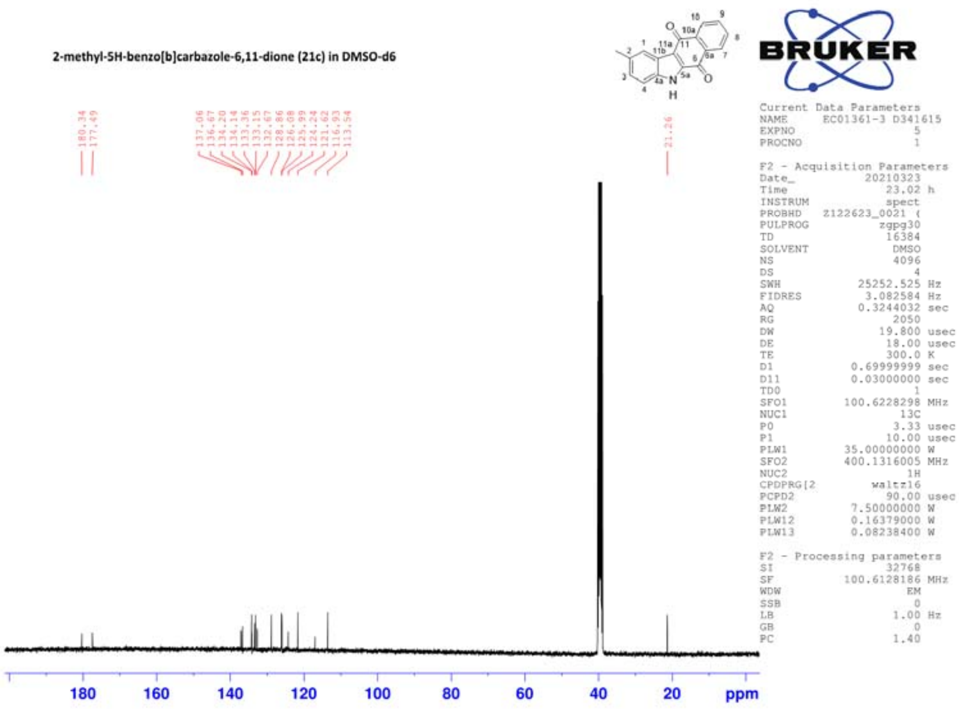

Figure S180. $^{13}C\{^{1}H\}$ NMR spectrum of 2-methyl-*5H*-benzo[*b*]carbazole-6,11-dione (**21c**).

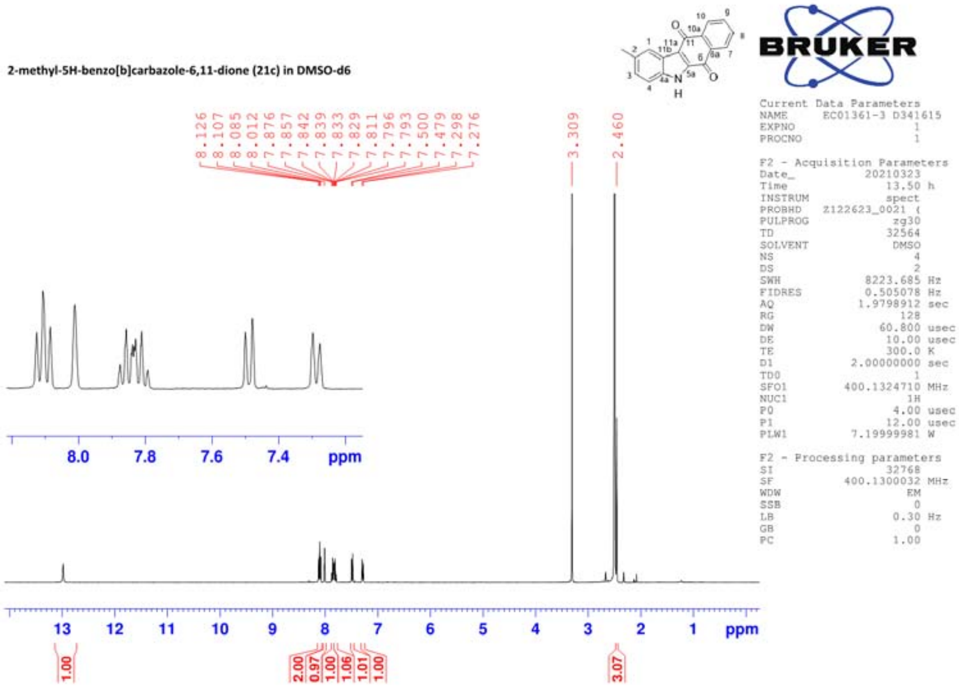

Figure S181. ^{1}H NMR spectrum of 2-methyl-*5H*-benzo[*b*]carbazole-6,11-dione (**21c**).

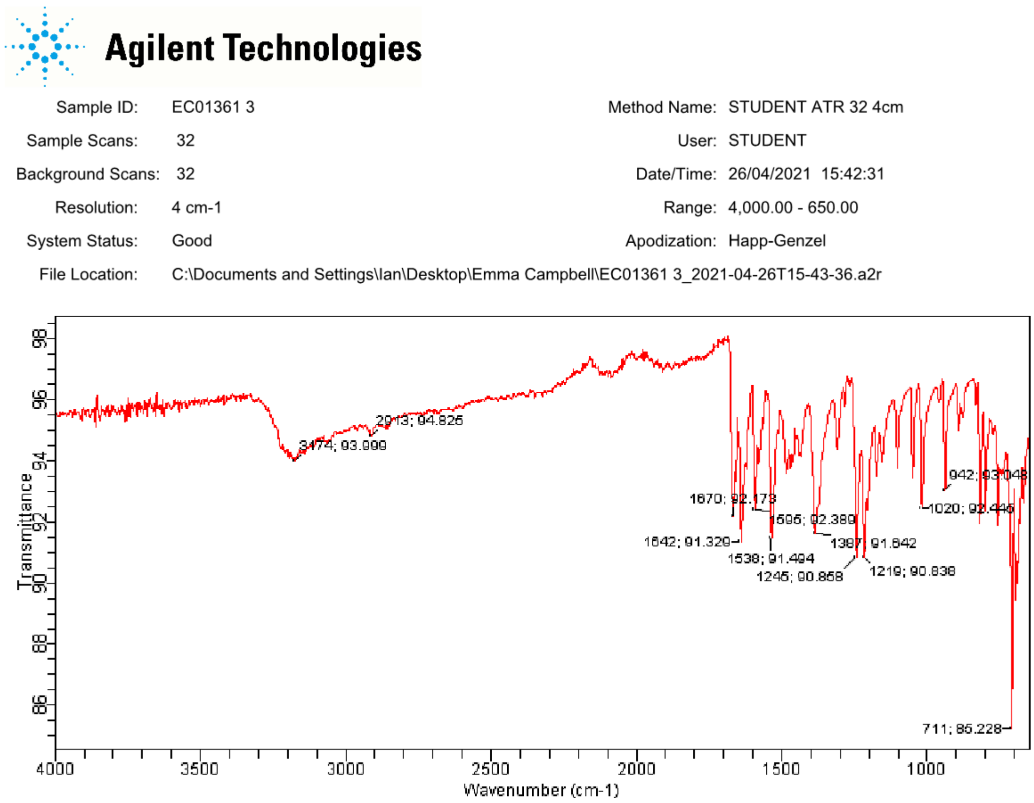

Figure S182. FT-IR spectrum of 2-methyl-*5H*-benzo[*b*]carbazole-6,11-dione (**21c**).

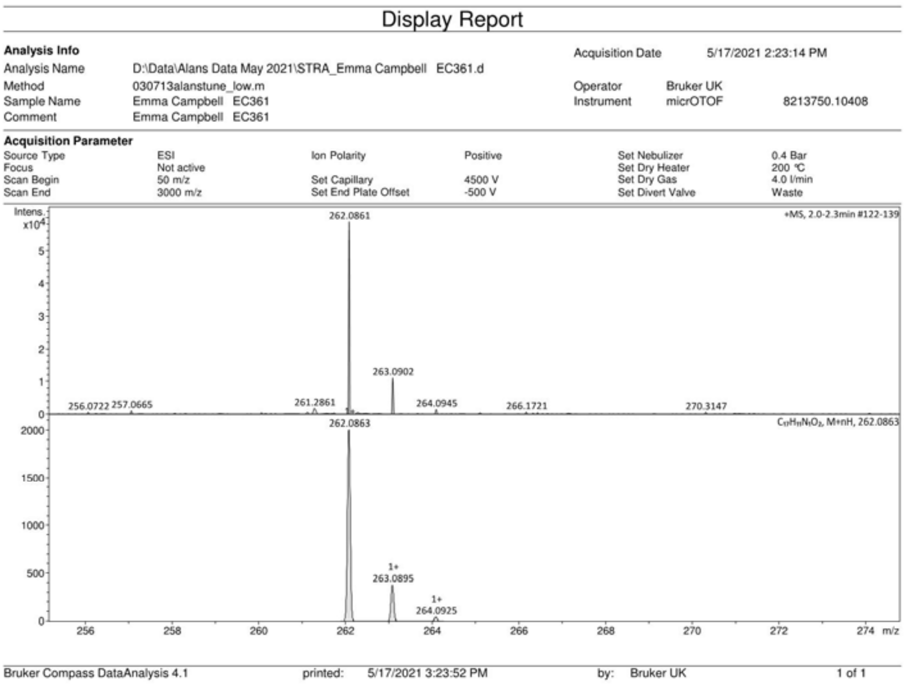

Figure S183. HRMS analysis of 2-methyl-*5H*-benzo[*b*]carbazole-6,11-dione (**21c**).

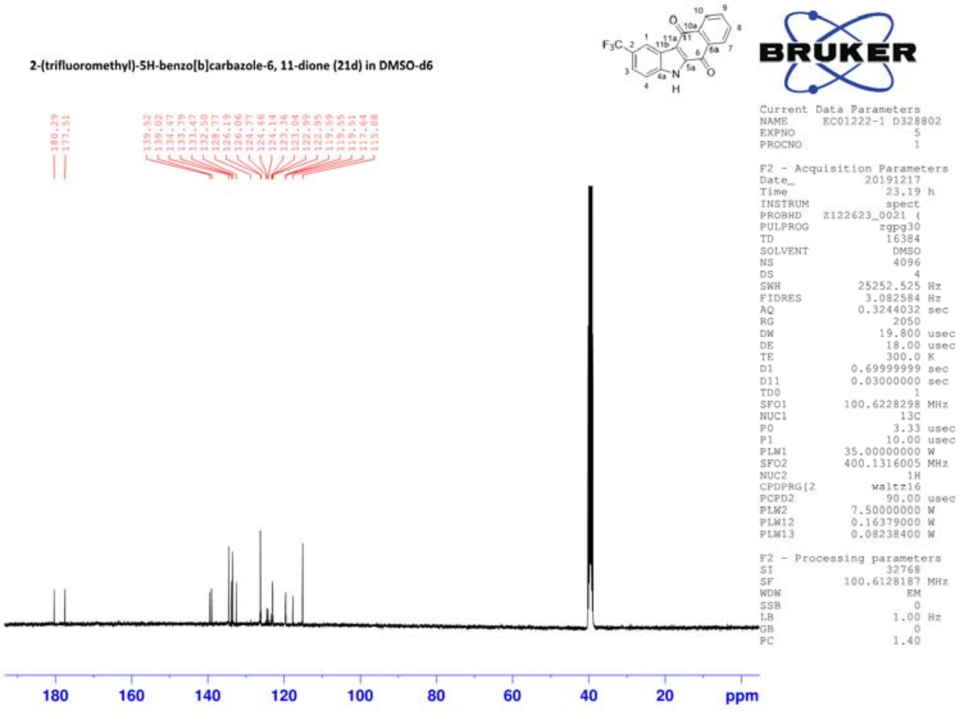

Figure S184. $^{13}C\{^{1}H\}$ NMR spectrum of 2-(trifluoromethyl)-*5H*-benzo[*b*]carbazole-6, 11-dione (**21d**).

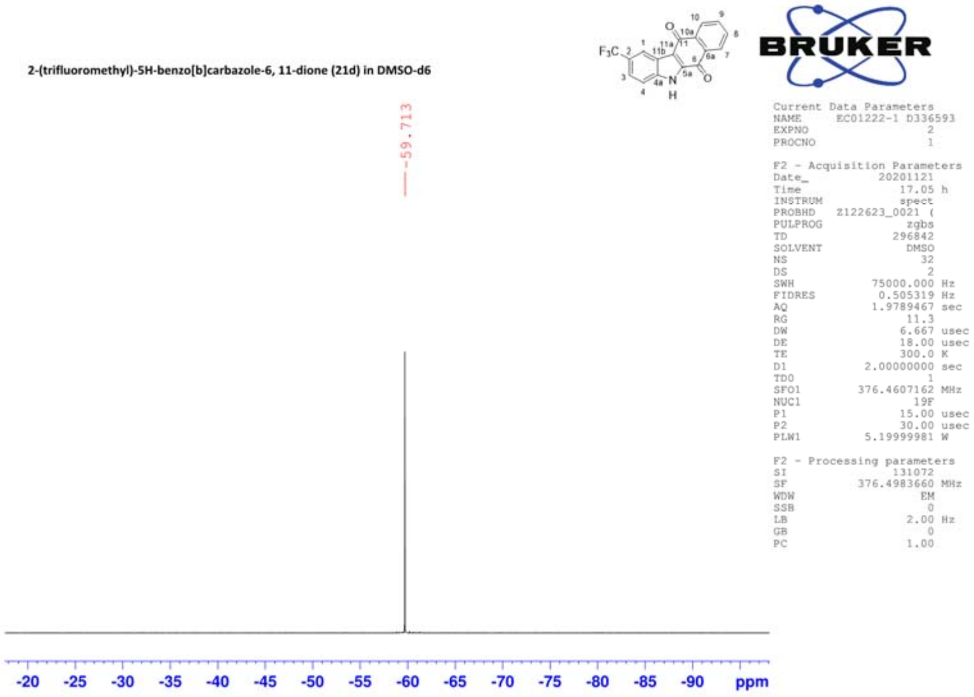

Figure S185. ^{19}F NMR spectrum of 2-(trifluoromethyl)-*5H*-benzo[*b*]carbazole-6, 11-dione (**21d**).

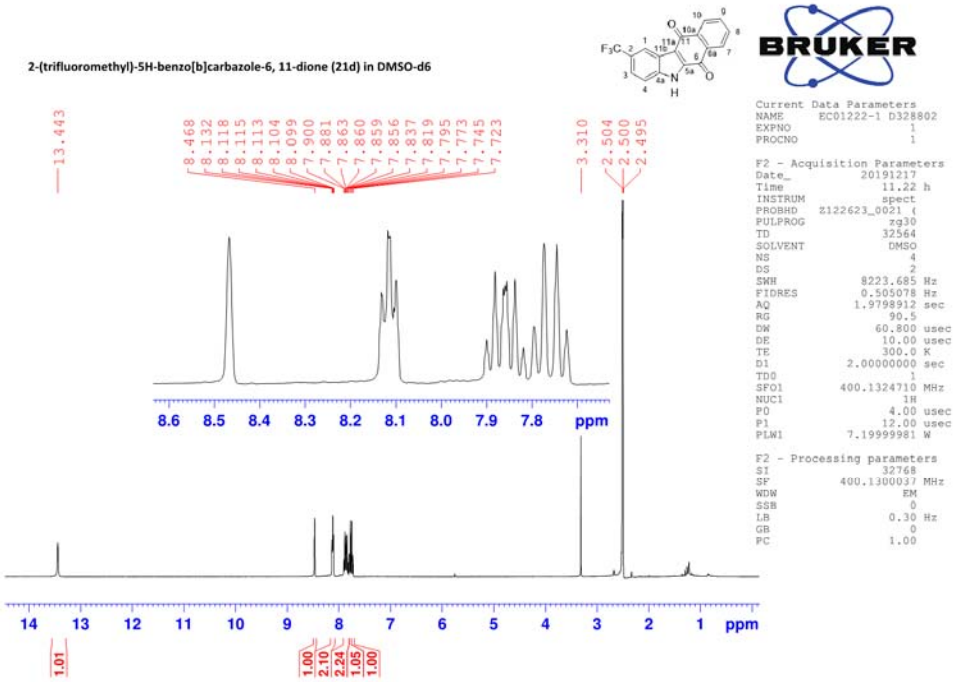

Figure S186. ^{1}H NMR spectrum of 2-(trifluoromethyl)-*5H*-benzo[*b*]carbazole-6, 11-dione (**21d**).

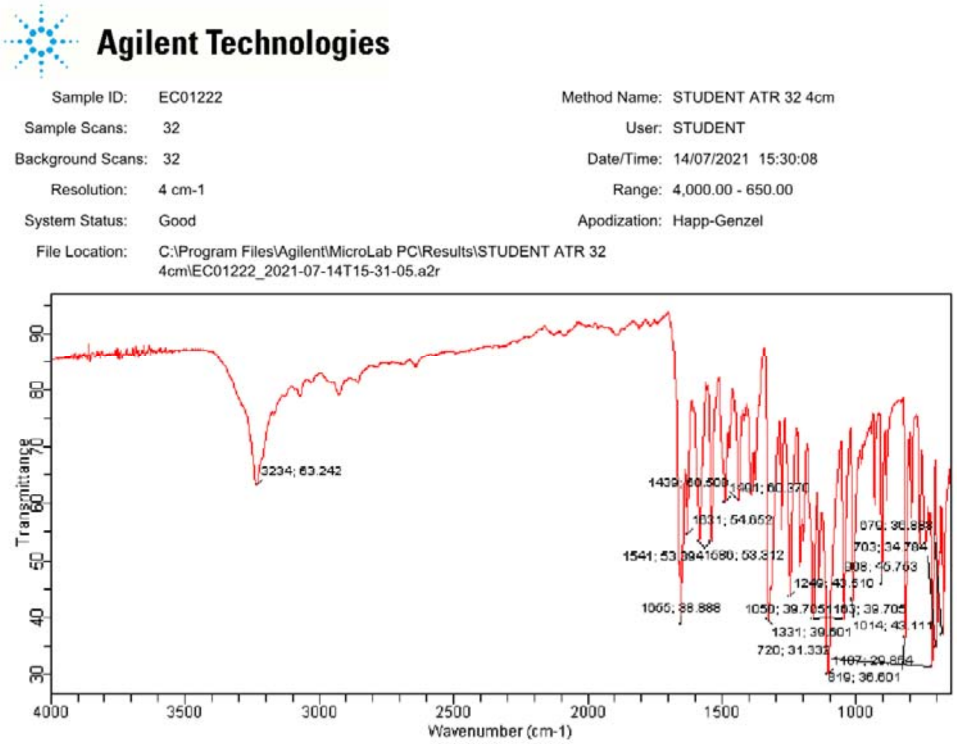

Figure S187. FT-IR spectrum of 2-(trifluoromethyl)-*5H*-benzo[*b*]carbazole-6, 11-dione (**21d**).

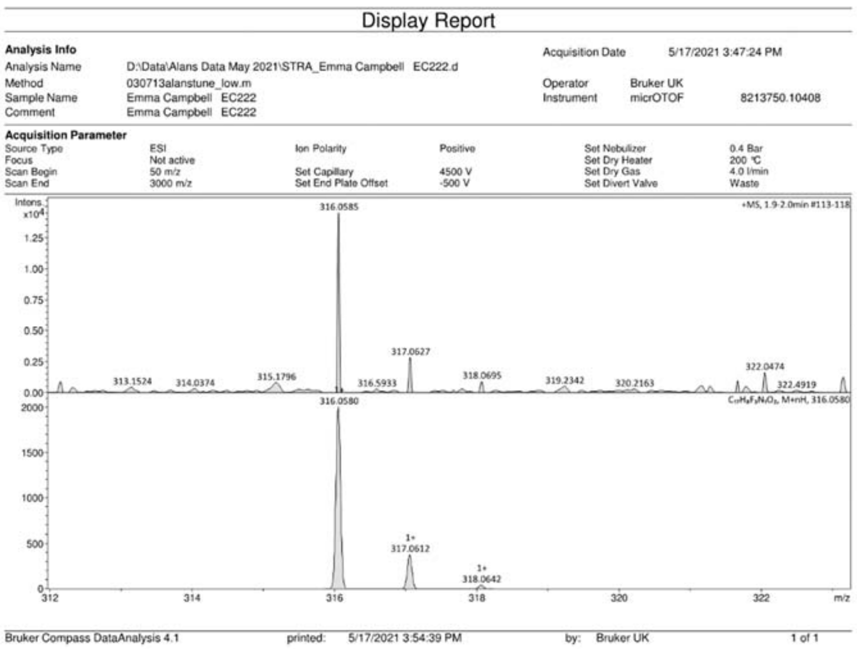

Figure S188. HRMS analysis of 2-(trifluoromethyl)-*5H*-benzo[*b*]carbazole-6, 11-dione (**21d**).

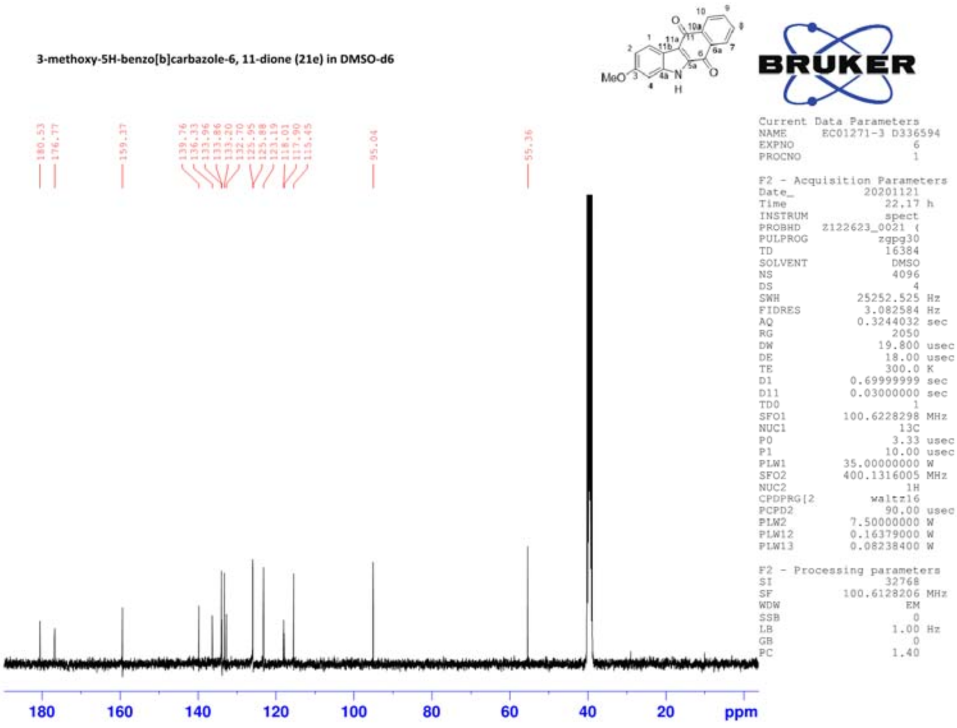

Figure S189. $^{13}C\{^{1}H\}$ NMR spectrum of 3-methoxy-*5H*-benzo[*b*]carbazole-6, 11-dione (**21e**).

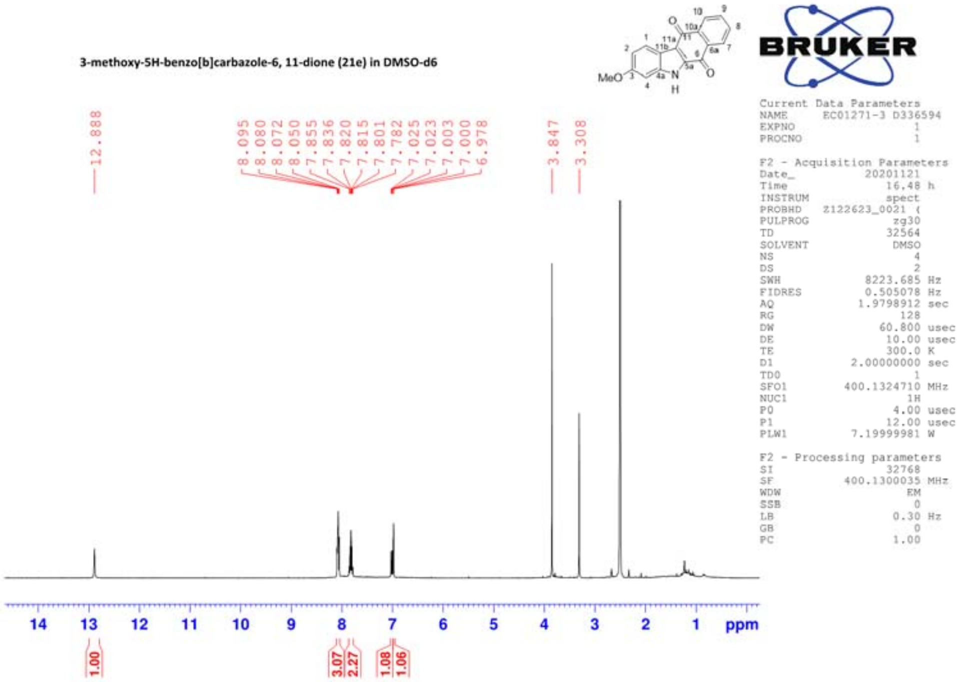

Figure S190. ^{1}H NMR spectrum of 3-methoxy-*5H*-benzo[*b*]carbazole-6, 11-dione (**21e**).

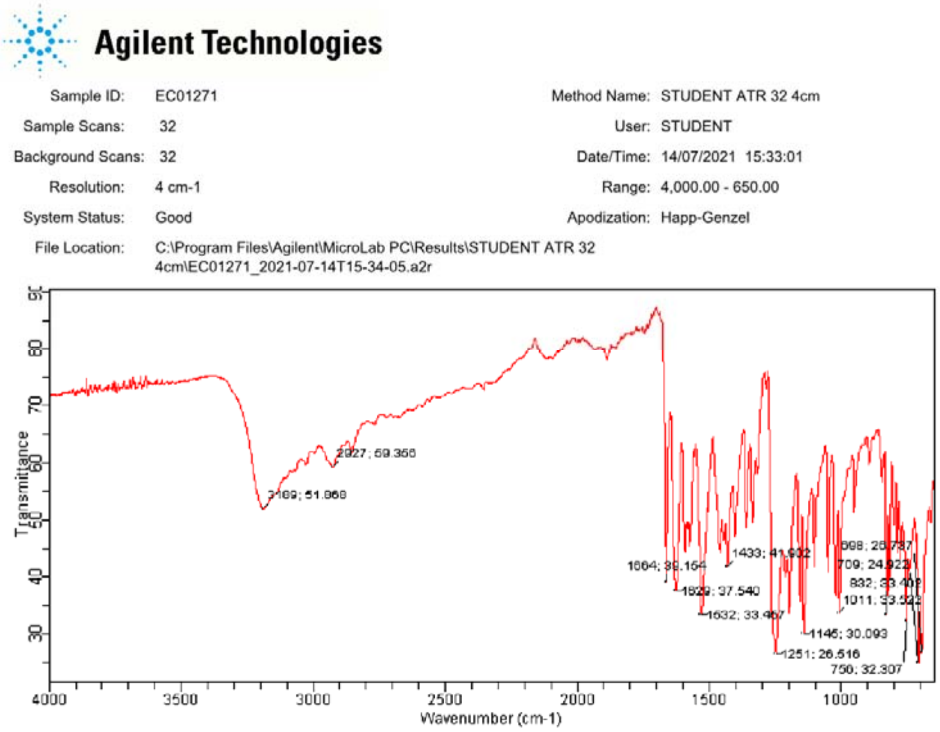

Figure S191. FT-IR spectrum of 3-methoxy-*5H*-benzo[*b*]carbazole-6, 11-dione (**21e**).

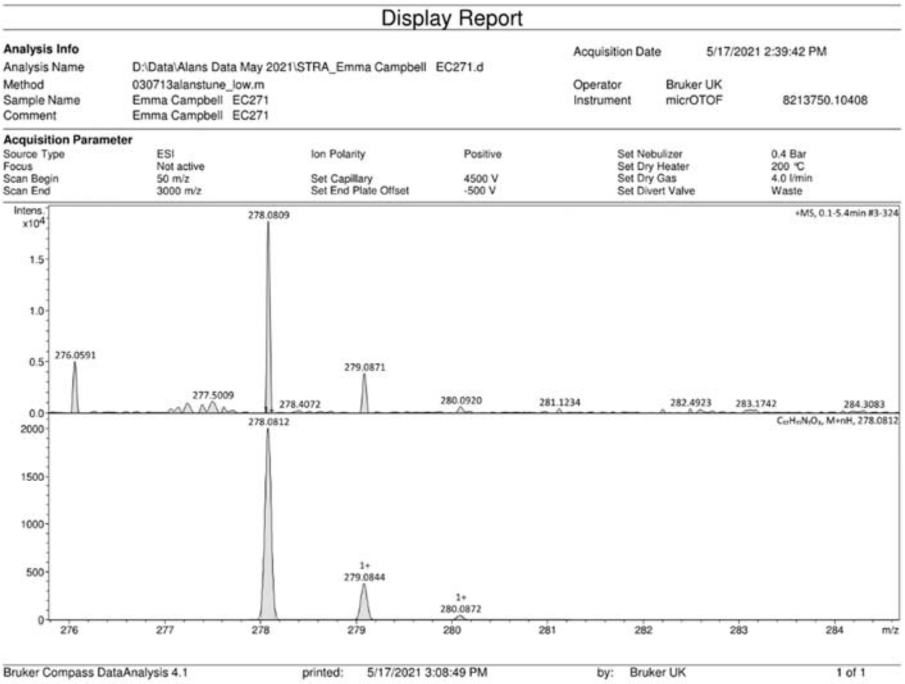

Figure S192. HRMS analysis of 3-methoxy-*5H*-benzo[*b*]carbazole-6, 11-dione (**21e**).

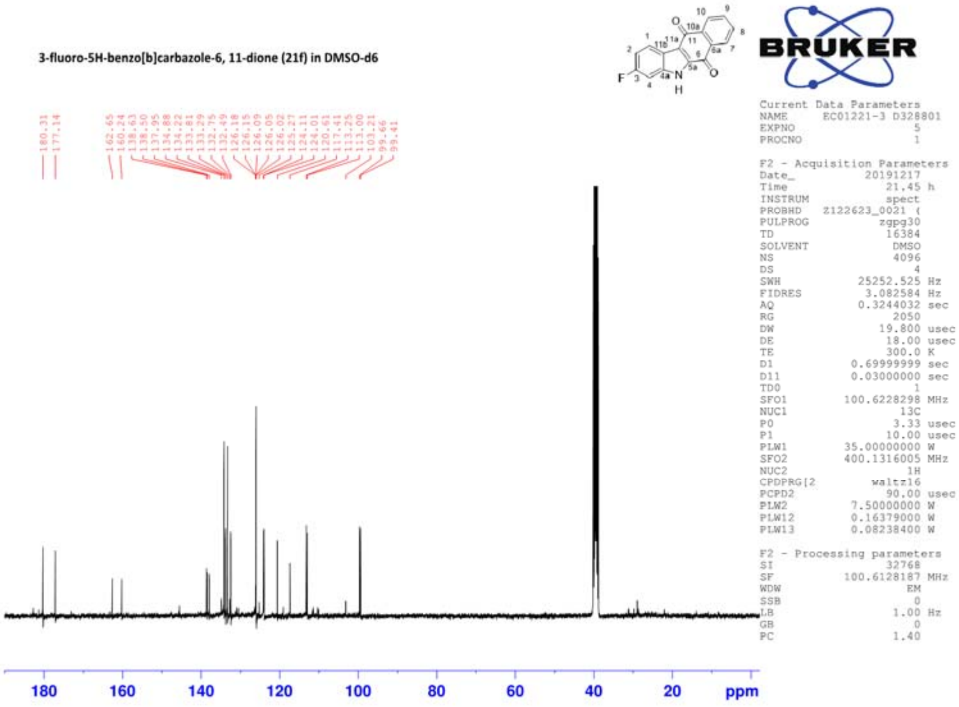

Figure S193. $^{13}C\{^1H\}$ NMR spectrum of 3-fluoro-*5H*-benzo[*b*]carbazole-6, 11-dione (**21f**).

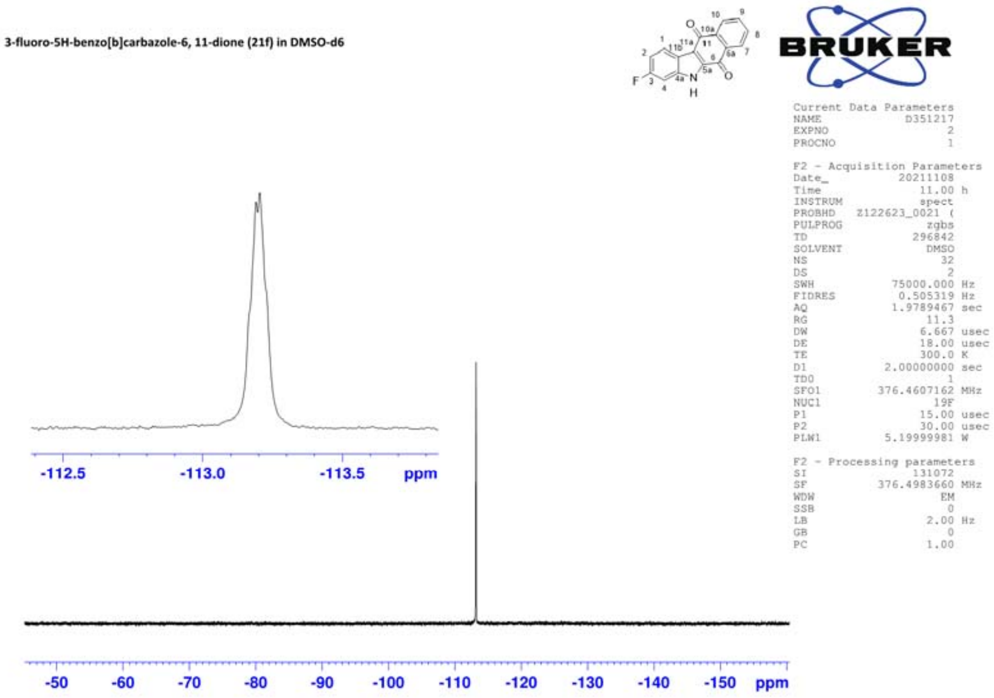

Figure S194. ^{19}F NMR spectrum of 3-fluoro-*5H*-benzo[*b*]carbazole-6, 11-dione (**21f**).

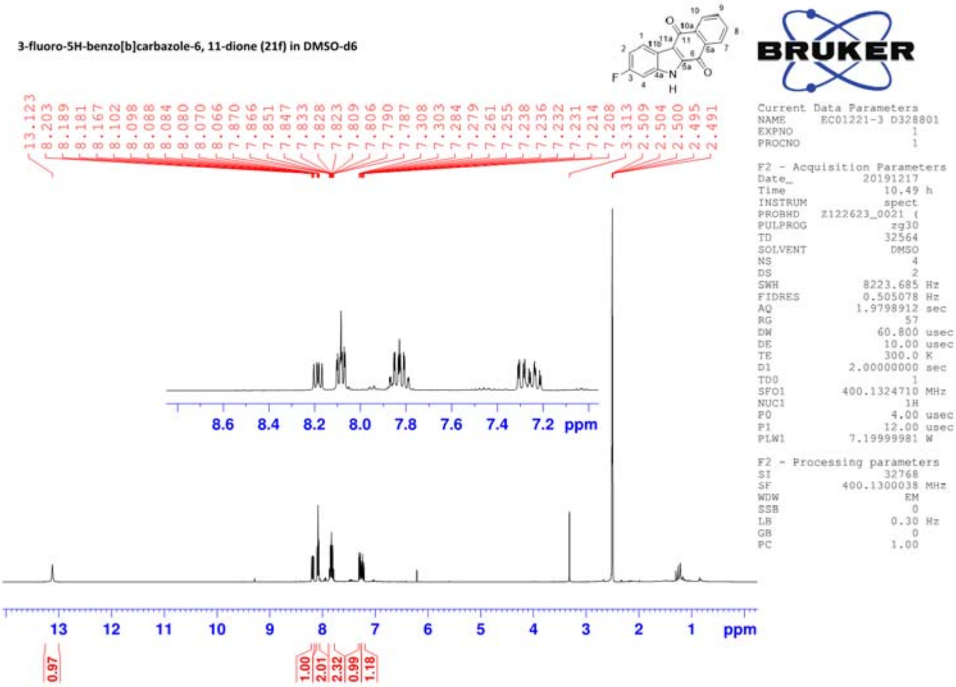

Figure S195. ^{1}H NMR spectrum of 3-fluoro-*5H*-benzo[*b*]carbazole-6, 11-dione (**21f**).

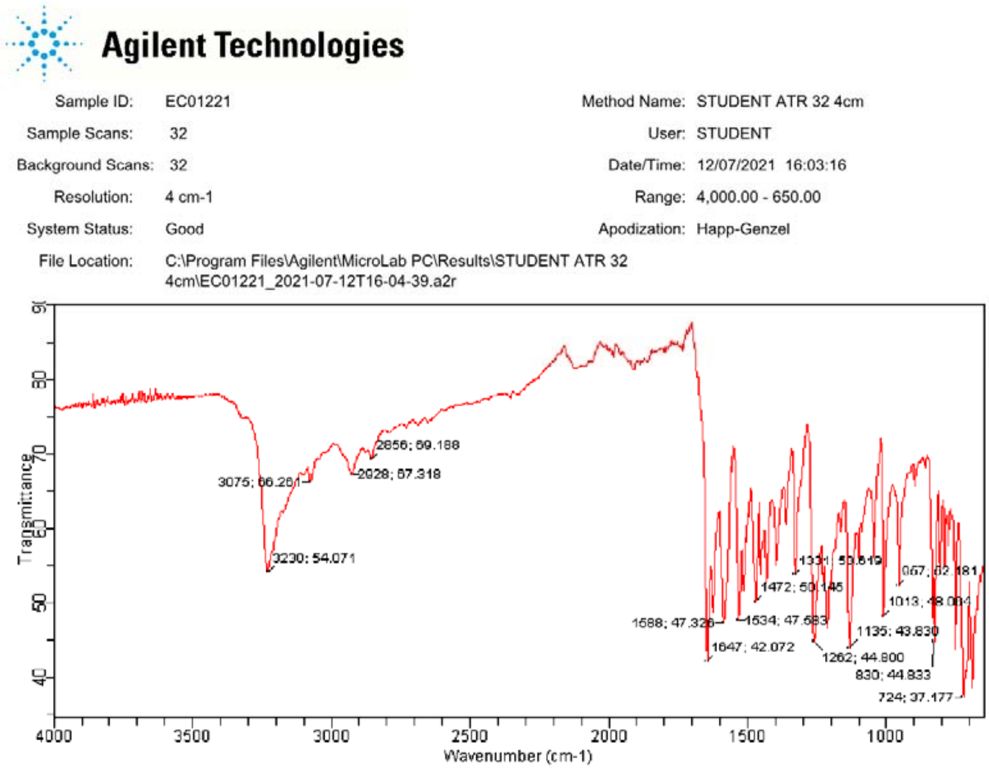

Figure S196. FT-IR spectrum of 3-fluoro-*5H*-benzo[*b*]carbazole-6, 11-dione (**21f**).

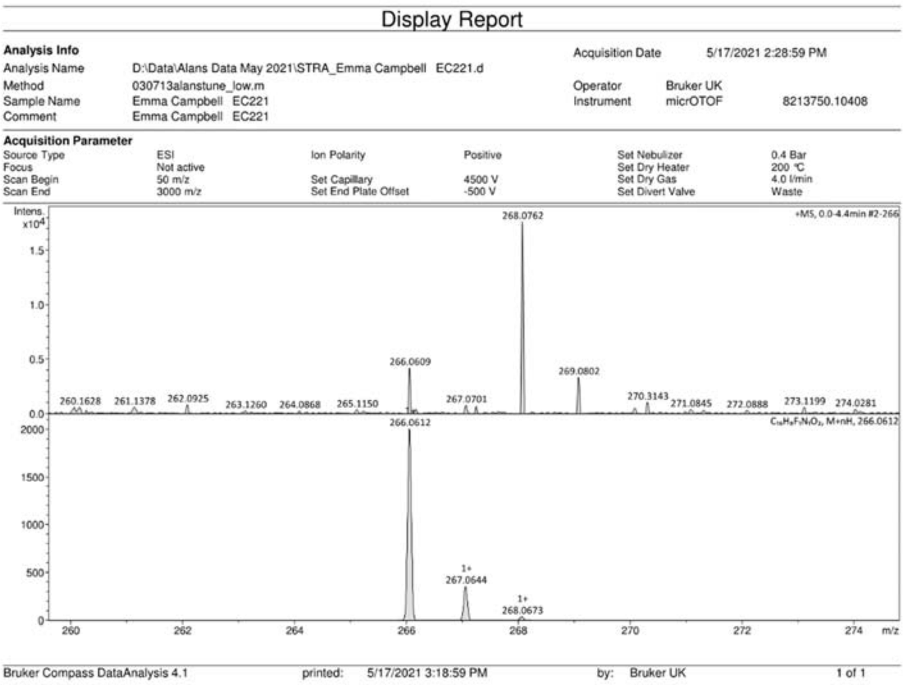

Figure S197. HRMS analysis of 3-fluoro-*5H*-benzo[*b*]carbazole-6, 11-dione (**21f**).

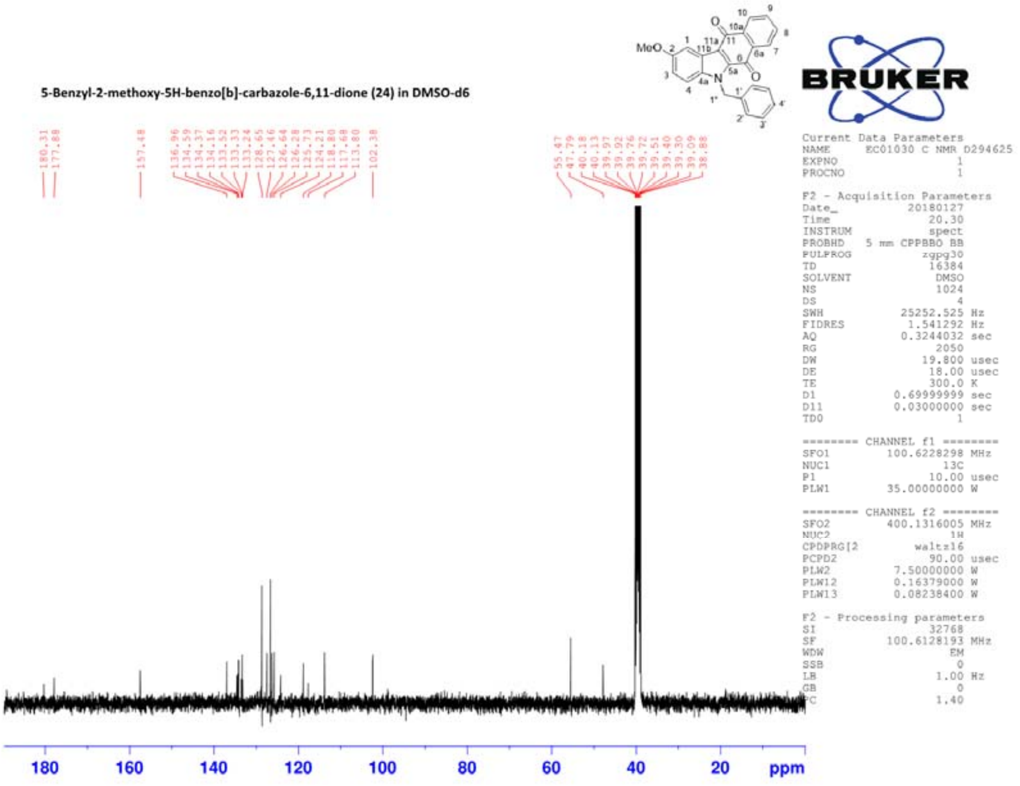

Figure S198. $^{13}C\{^1H\}$ NMR spectrum of 5-Benzyl-2-methoxy-*5H*-benzo[*b*]-carbazole-6,11-dione (**24**).

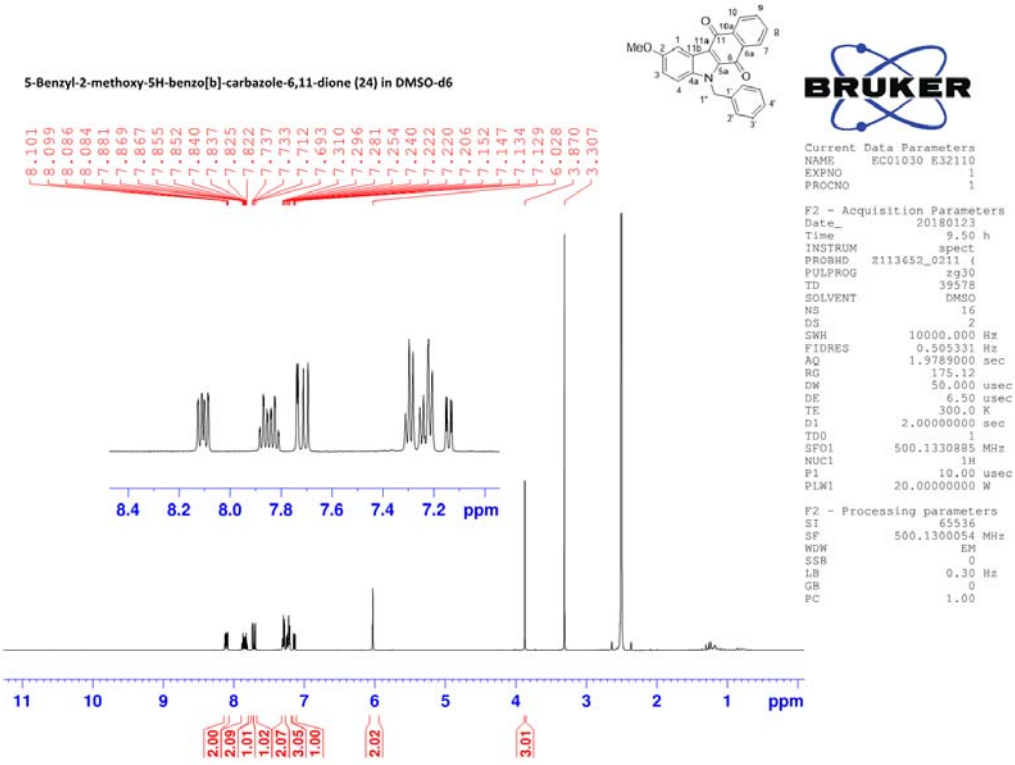

Figure S199. ^1H NMR spectrum of 5-Benzyl-2-methoxy-*5H*-benzo[*b*]-carbazole-6,11-dione (**24**).

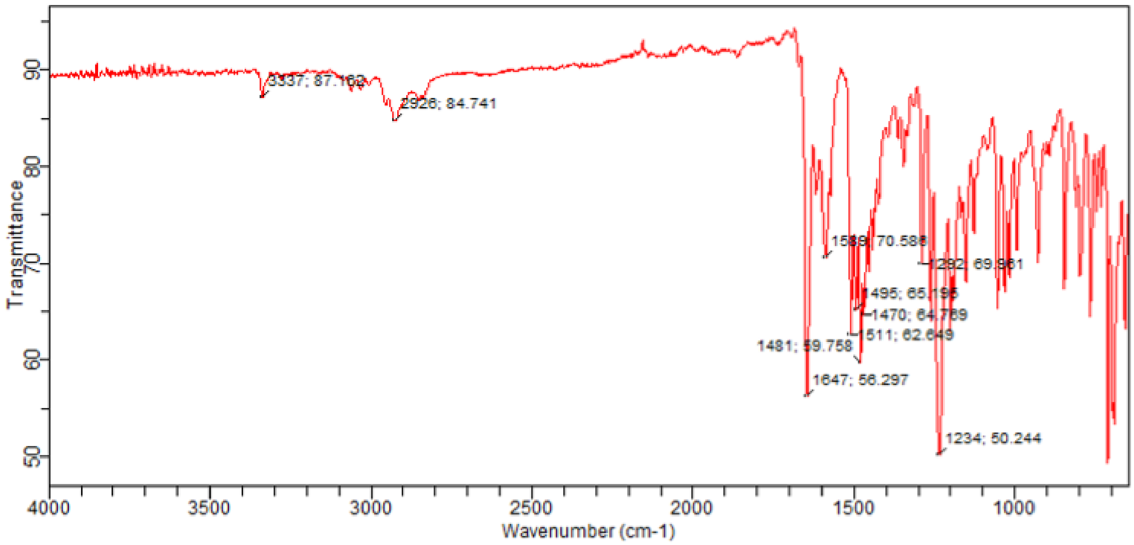

Figure S200. FT-IR spectrum of 5-Benzyl-2-methoxy-*5H*-benzo[*b*]-carbazole-6,11-dione (**24**).

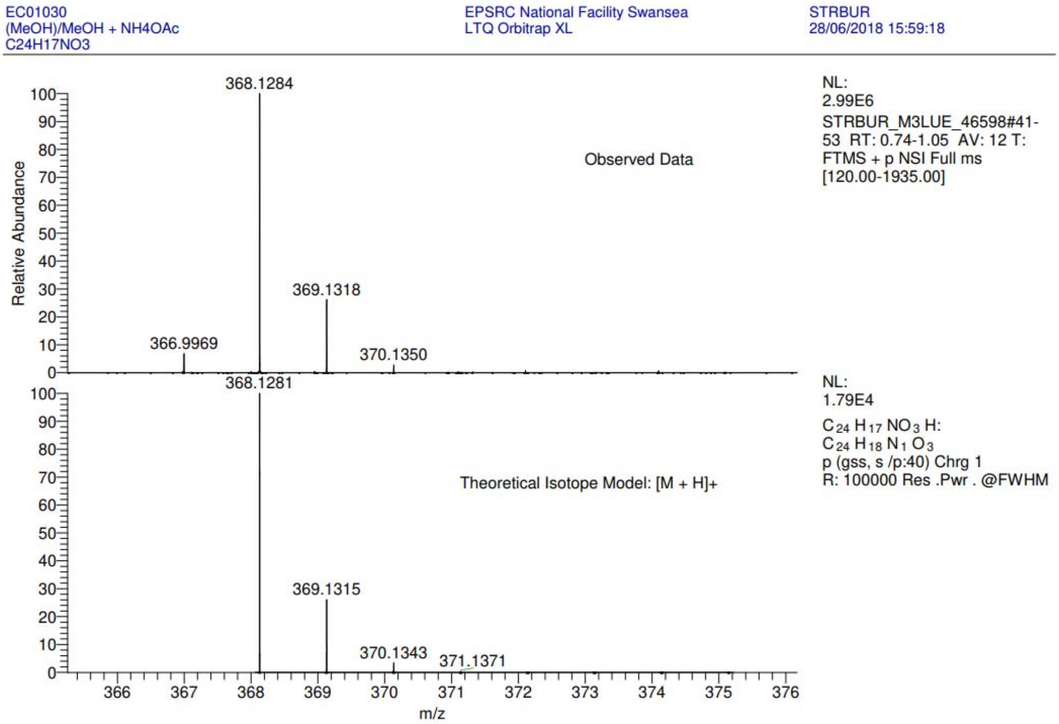

Figure S201. HRMS analysis of 5-Benzyl-2-methoxy-*5H*-benzo[*b*]-carbazole-6,11-dione (**24**).

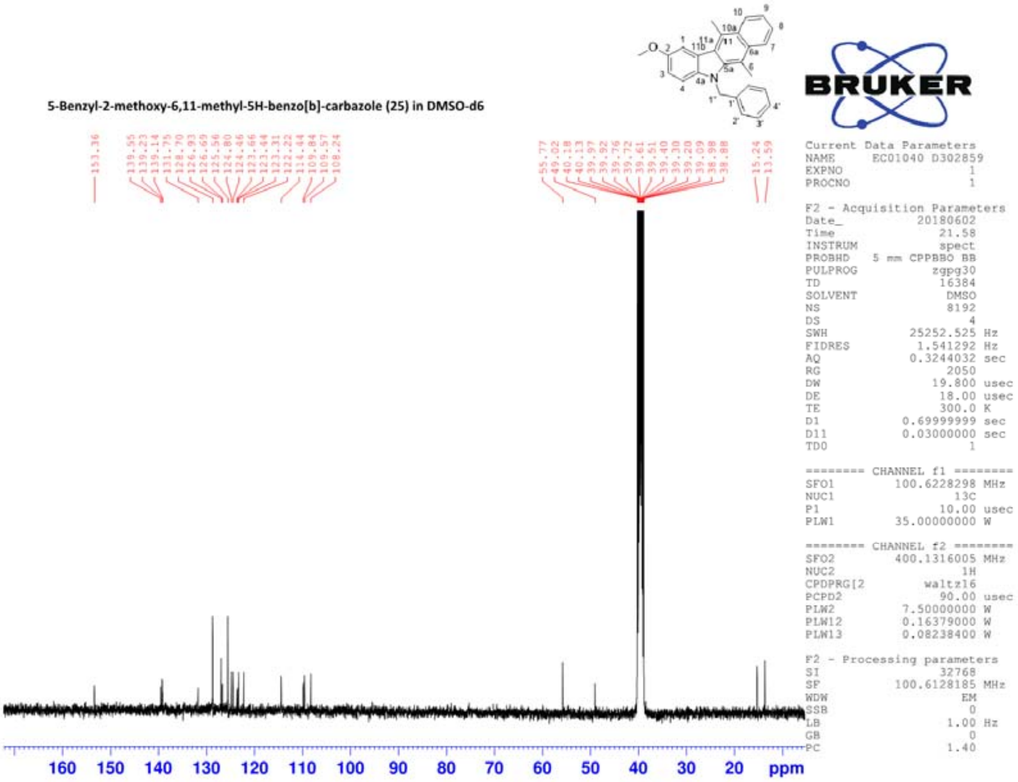

Figure S202. $^{13}C\{^{1}H\}$ NMR spectrum of 5-Benzyl-2-methoxy-6,11-methyl-*5H*-benzo[*b*]-carbazole (**25**).

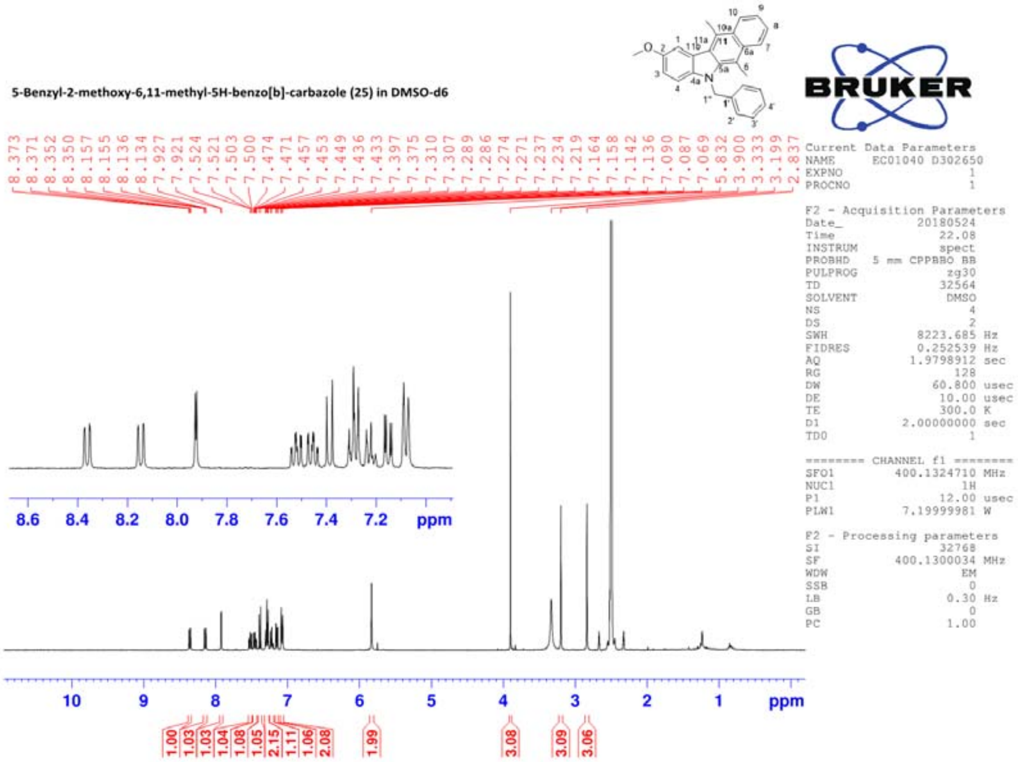

Figure S203. ^{1}H NMR spectrum of 5-Benzyl-2-methoxy-6,11-methyl-*5H*-benzo[*b*]-carbazole (**25**).

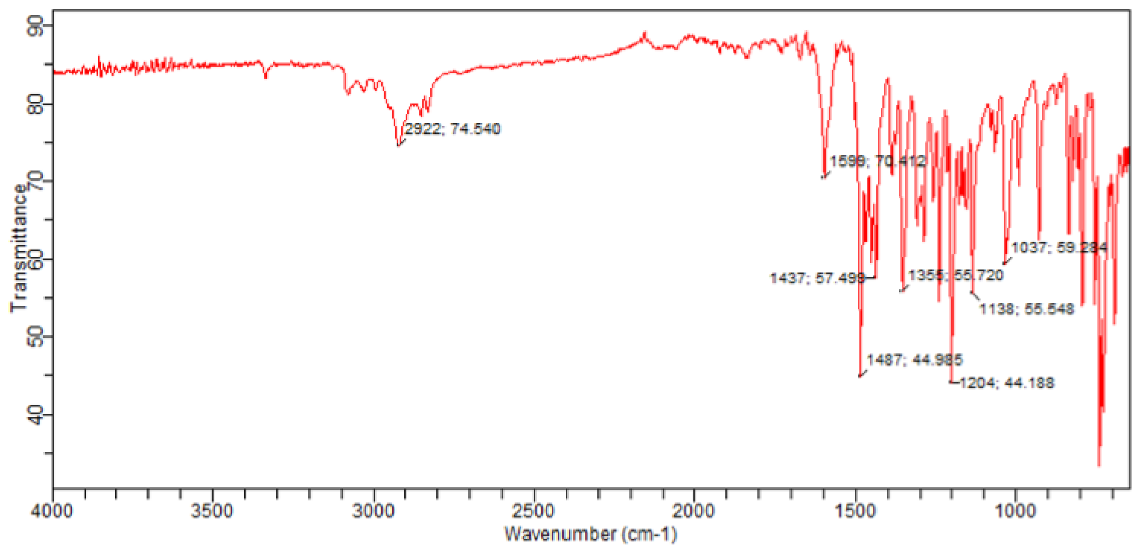

Figure S204. FT-IR spectrum of 5-Benzyl-2-methoxy-6,11-methyl-*5H*-benzo[*b*]-carbazole (**25**).

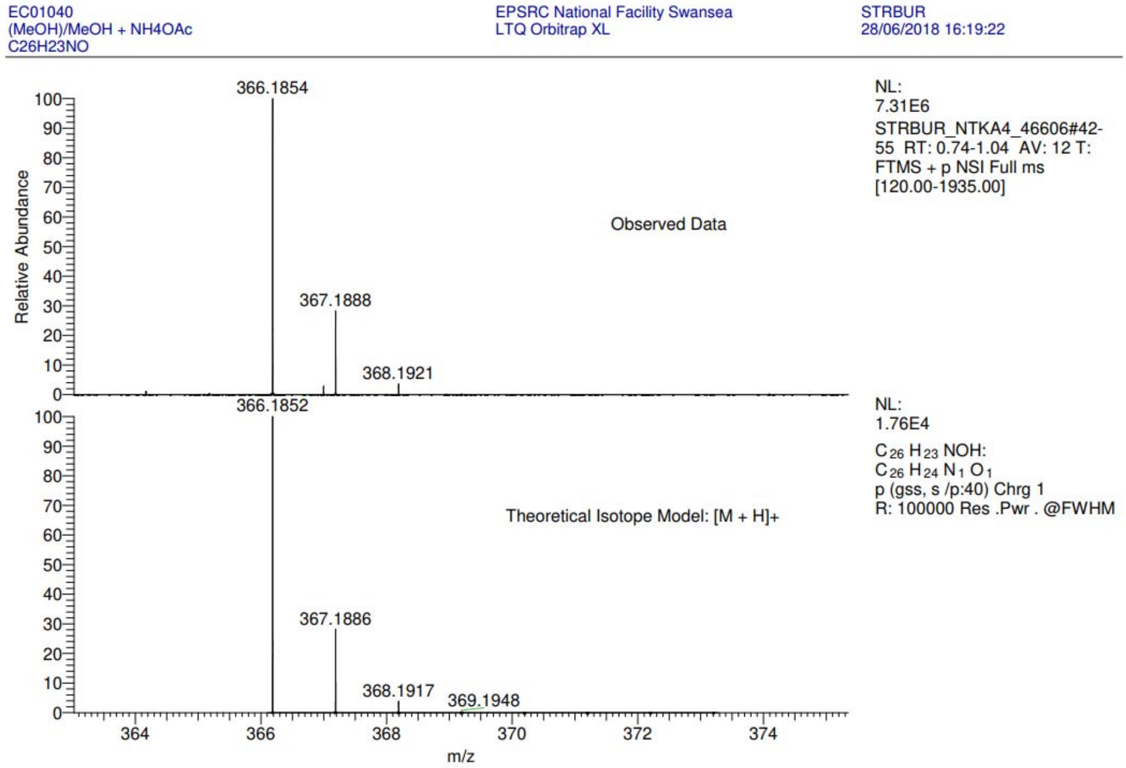

Figure S205. HRMS analysis of 5-Benzyl-2-methoxy-6,11-methyl-*5H*-benzo[*b*]-carbazole (**25**).

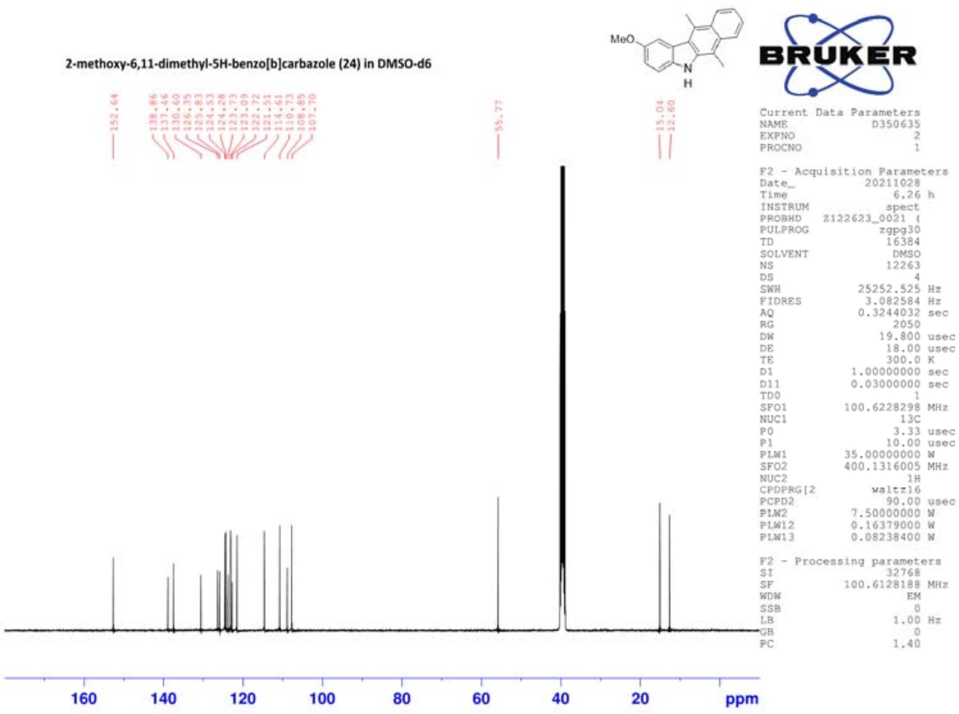

Figure S206. $^{13}C\{^1H\}$ NMR of 2-methoxy-6,11-dimethyl-5H-benzo[b]carbazole (**26**).

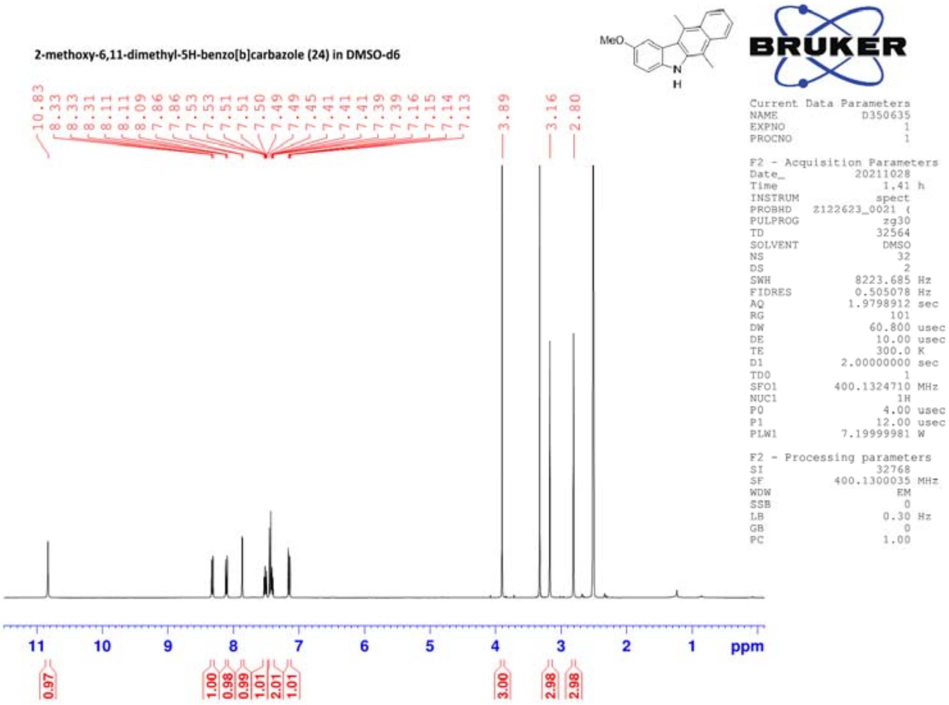

Figure S207. ^1H NMR of 2-methoxy-6,11-dimethyl-5H-benzo[b]carbazole (**26**).

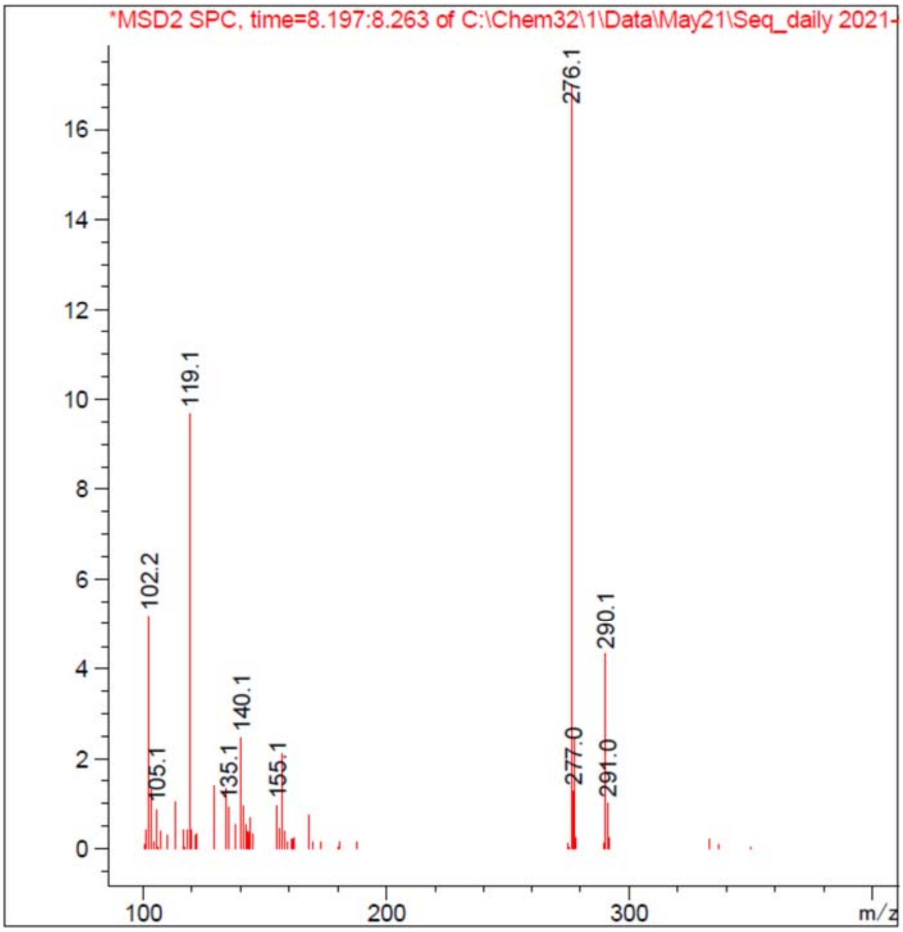

Figure S208. ESI MS of 2-methoxy-6,11-dimethyl-5H-benzo[b]carbazole (**26**).

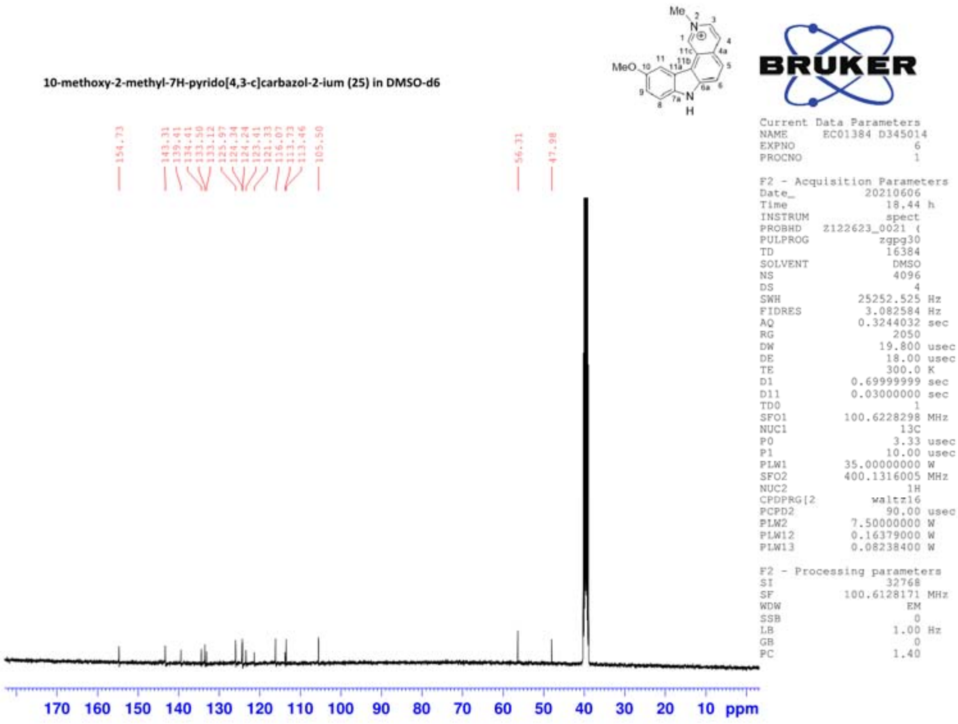

Figure S209. $^{13}C\{^{1}H\}$ NMR spectrum of 10-methoxy-2-methyl-*7H*-pyrido[4,3-*c*]carbazol-2-ium (**27**).

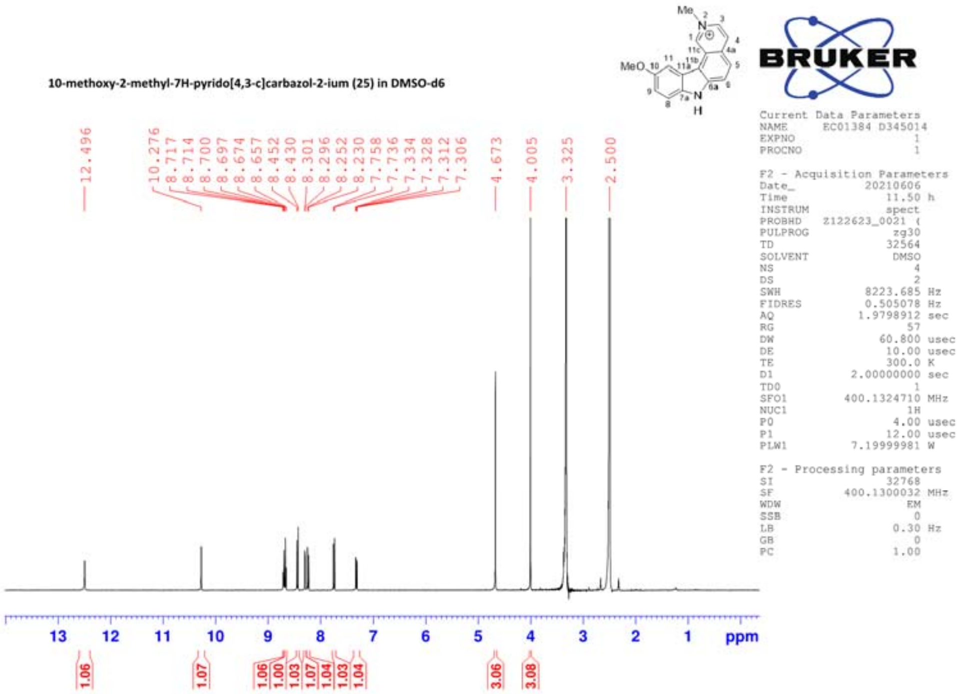

Figure S210. ^{1}H NMR spectrum of 10-methoxy-2-methyl-*7H*-pyrido[4,3-*c*]carbazol-2-ium (**27**).

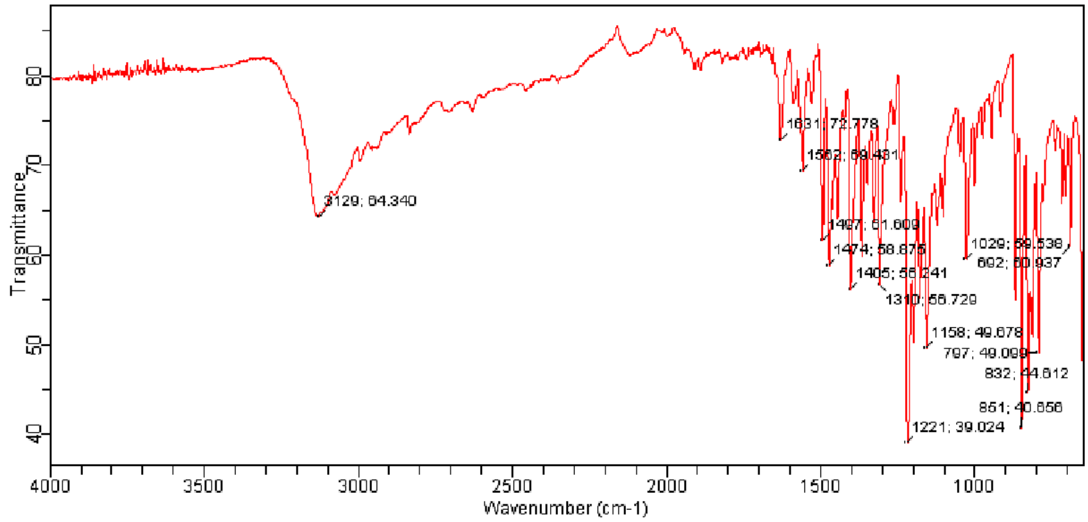

Figure S211. FT-IR spectrum of 10-methoxy-2-methyl-*7H*-pyrido[4,3-*c*]carbazol-2-ium (**27**).

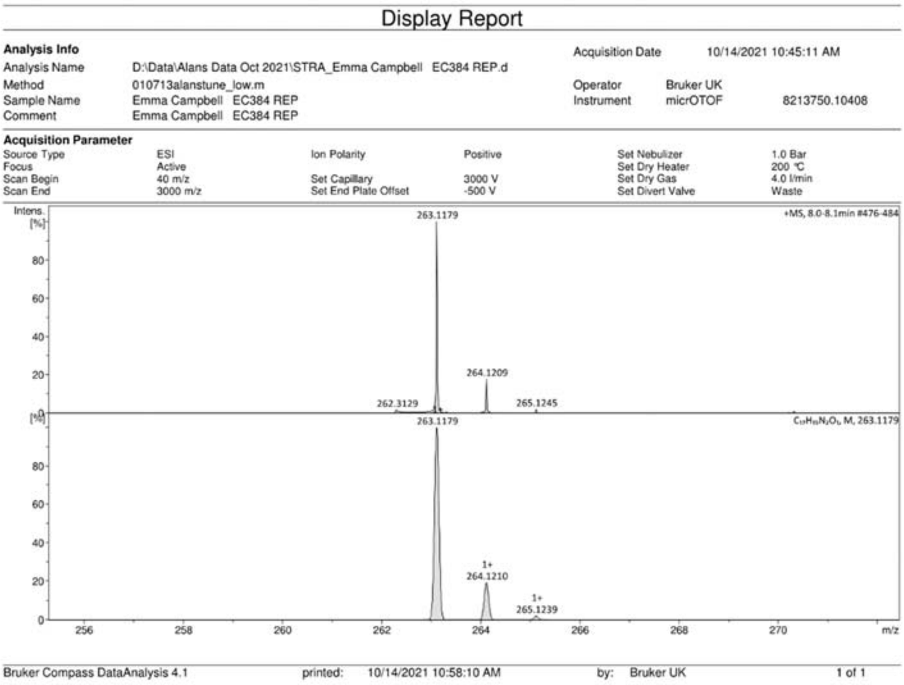

Figure S212. HRMS analysis of 10-methoxy-2-methyl-*7H*-pyrido[4,3-*c*]carbazol-2-ium (**27**).

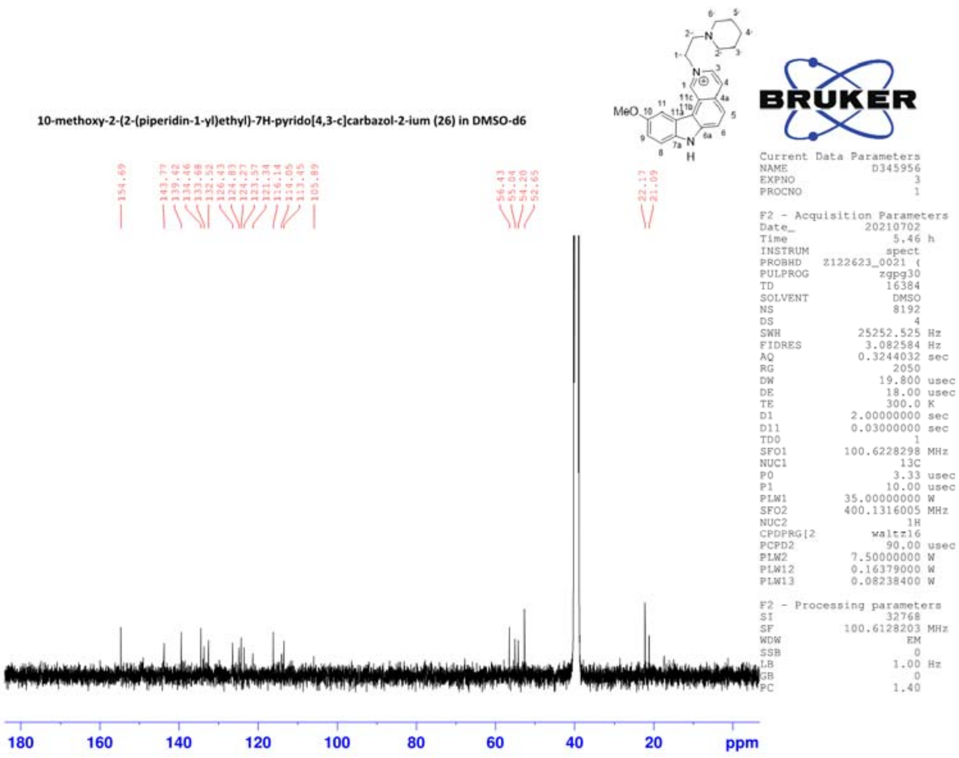

Figure S213. $^{13}C\{^{1}H\}$ NMR spectrum of 10-methoxy-2-(2-(piperidin-1-yl)ethyl)-*7H*-pyrido[4,3-*c*]carbazol-2-ium (**28**).

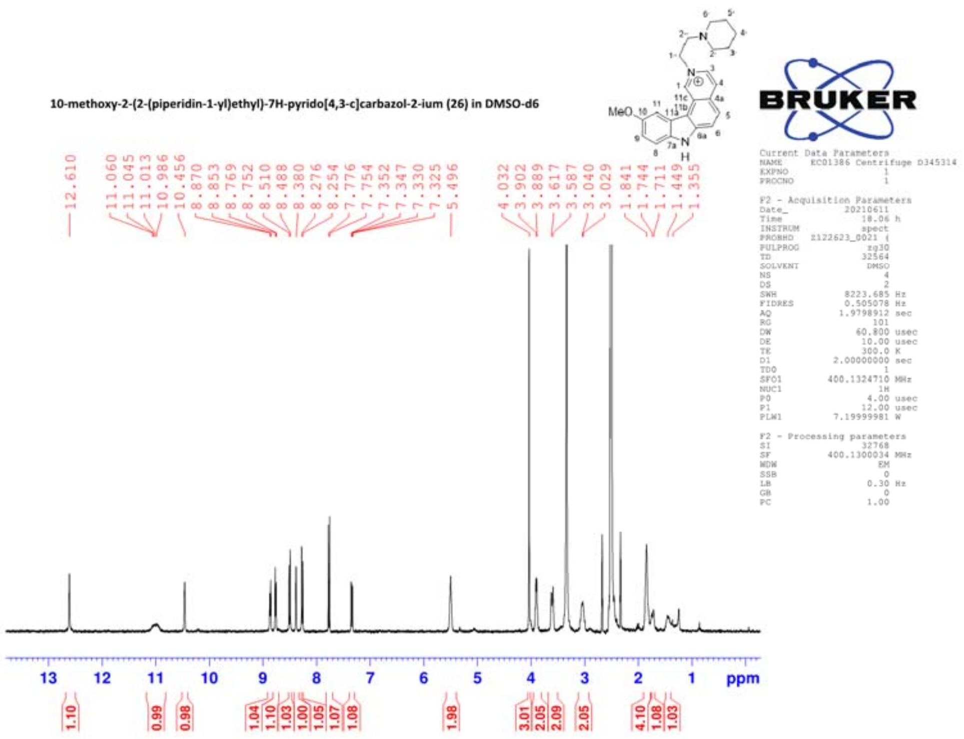

Figure S214. ^{1}H NMR spectrum of 10-methoxy-2-(2-(piperidin-1-yl)ethyl)-*7H*-pyrido[4,3-*c*]carbazol-2-ium (**28**).

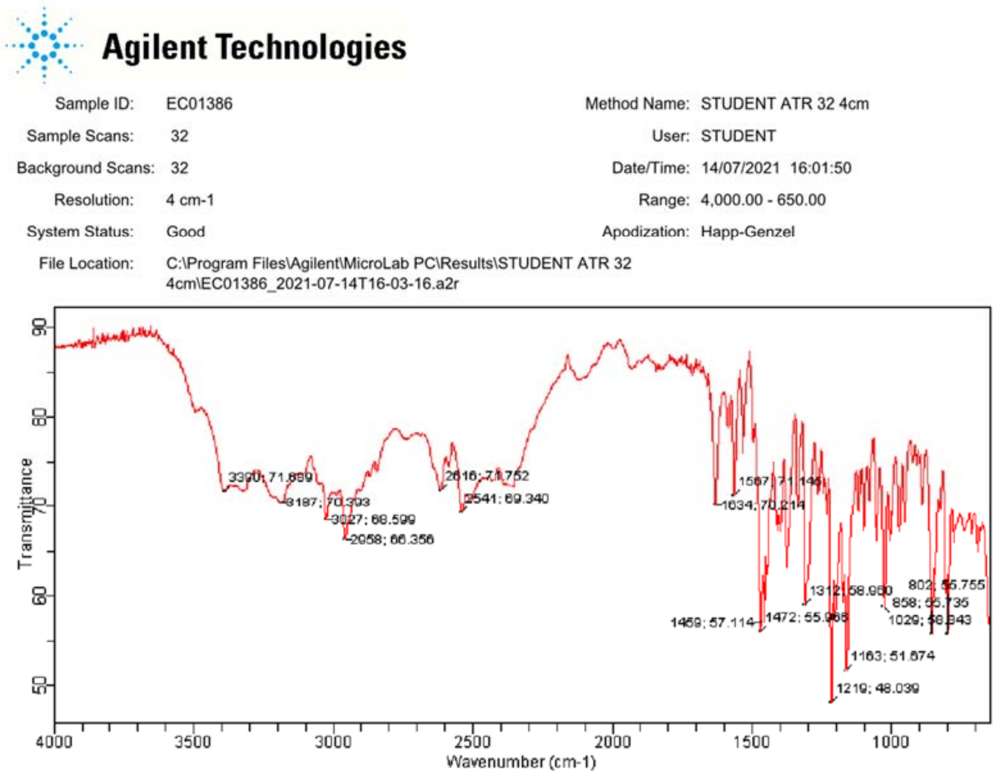

Figure S215. FT-IR spectrum of 10-methoxy-2-(2-(piperidin-1-yl)ethyl)-*7H*-pyrido[4,3-*c*]carbazol-2-ium (**28**).

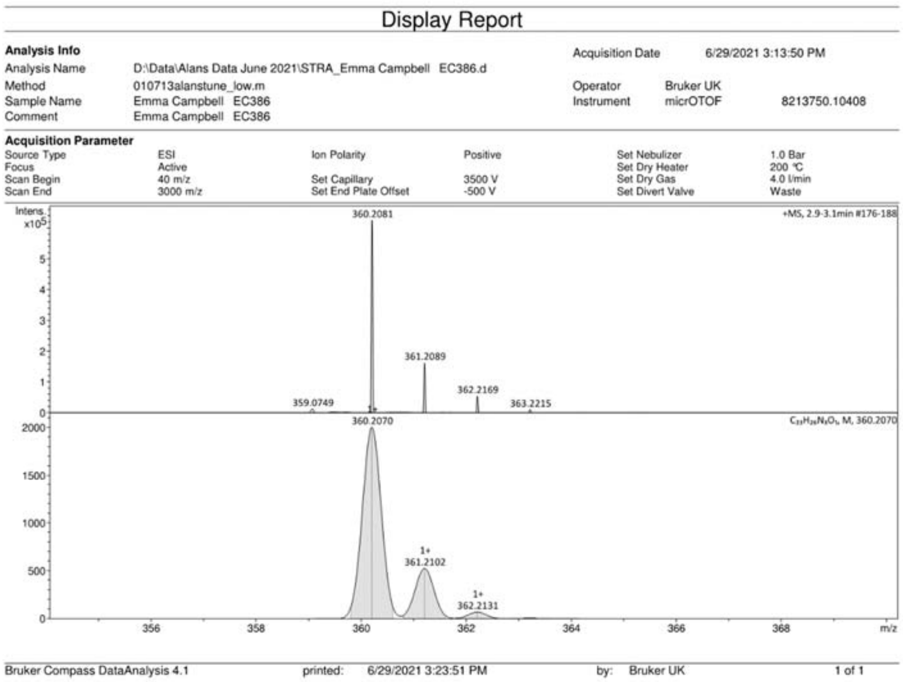

Figure S216. HRMS analysis of 10-methoxy-2-(2-(piperidin-1-yl)ethyl)-*7H*-pyrido[4,3-*c*]carbazol-2-ium (**28**).

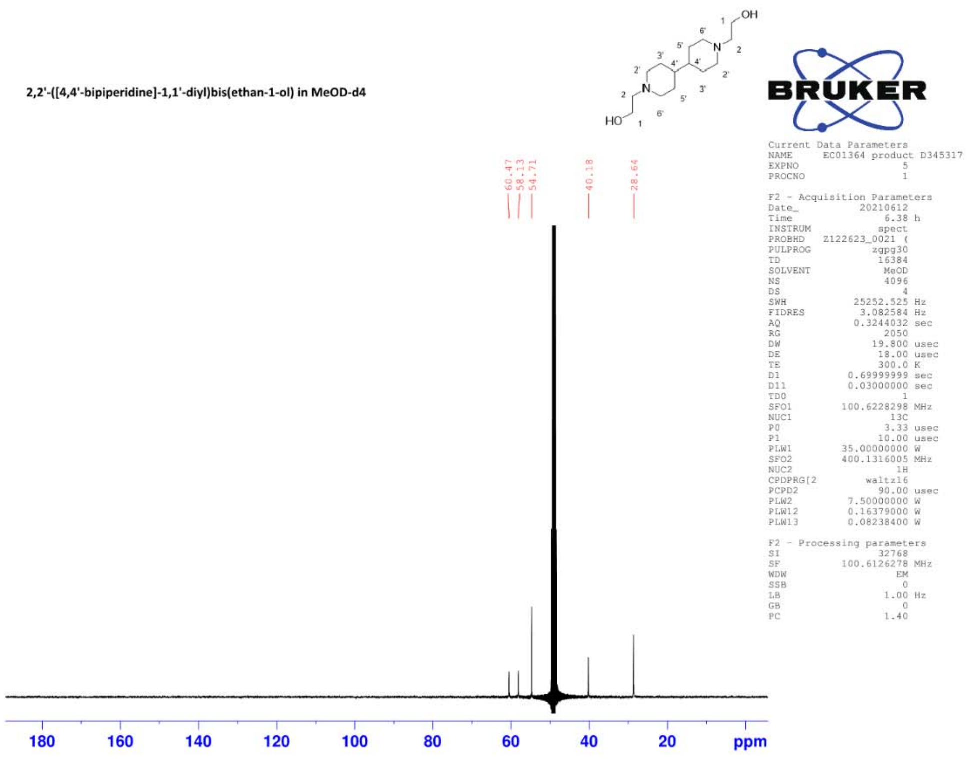

Figure S217. $^{13}C\{^1H\}$ NMR spectrum of 2,2'-([4,4'-bipiperidine]-1,1'-diyl)bis(ethan-1-ol) (**S1**).

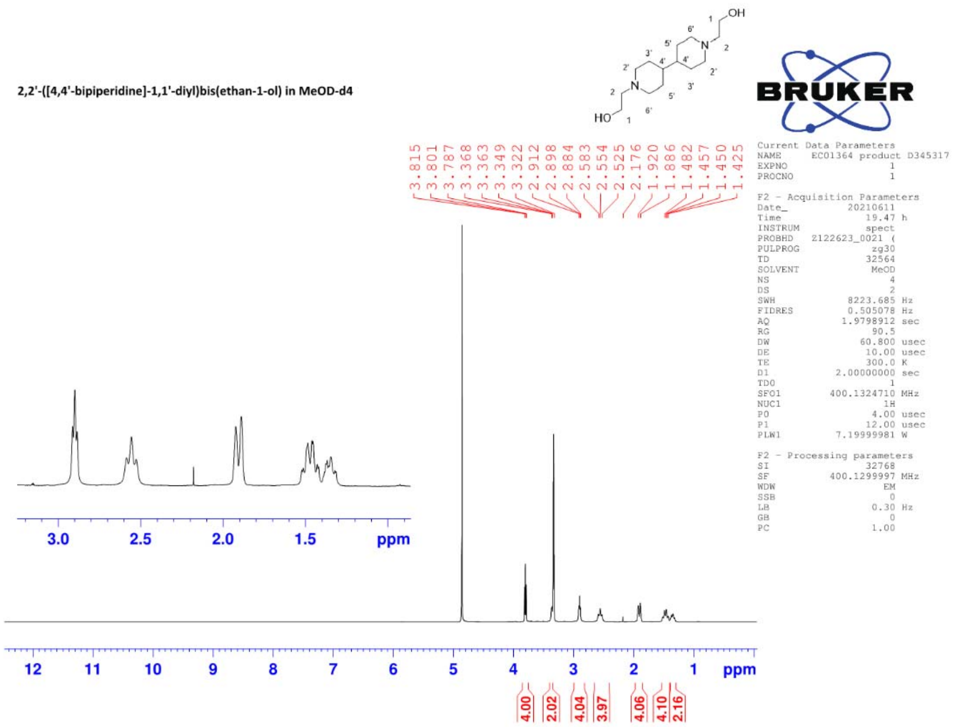

Figure S218. ^1H NMR spectrum of 2,2'-([4,4'-bipiperidine]-1,1'-diyl)bis(ethan-1-ol) (**S1**).

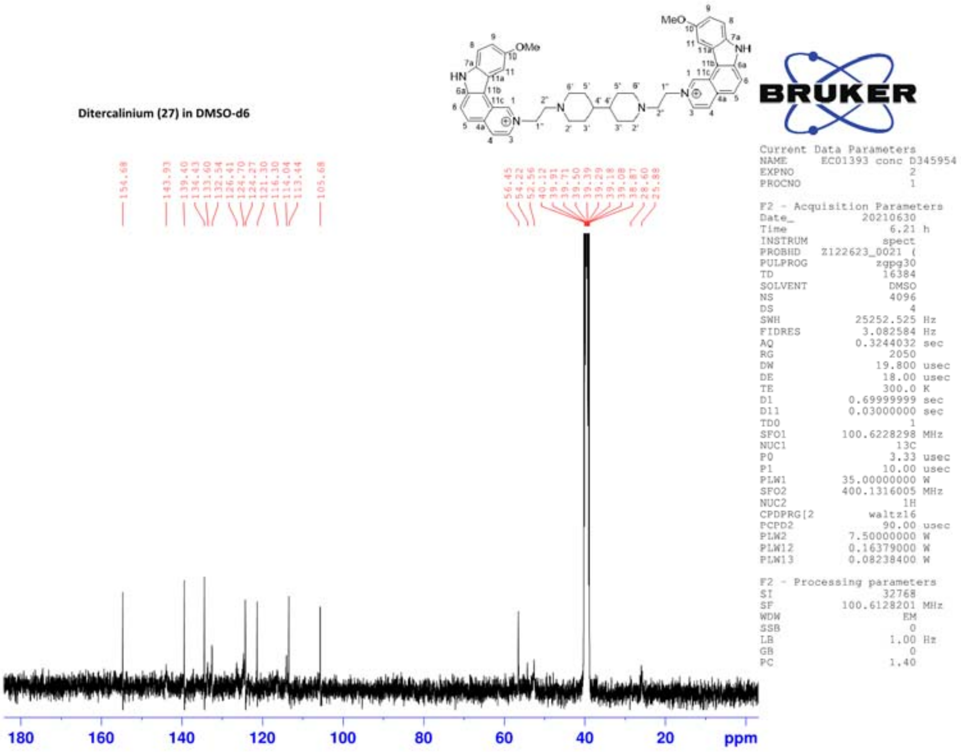

Figure S219. $^{13}C\{^{1}H\}$ NMR spectrum of Ditercalinium (**29**).

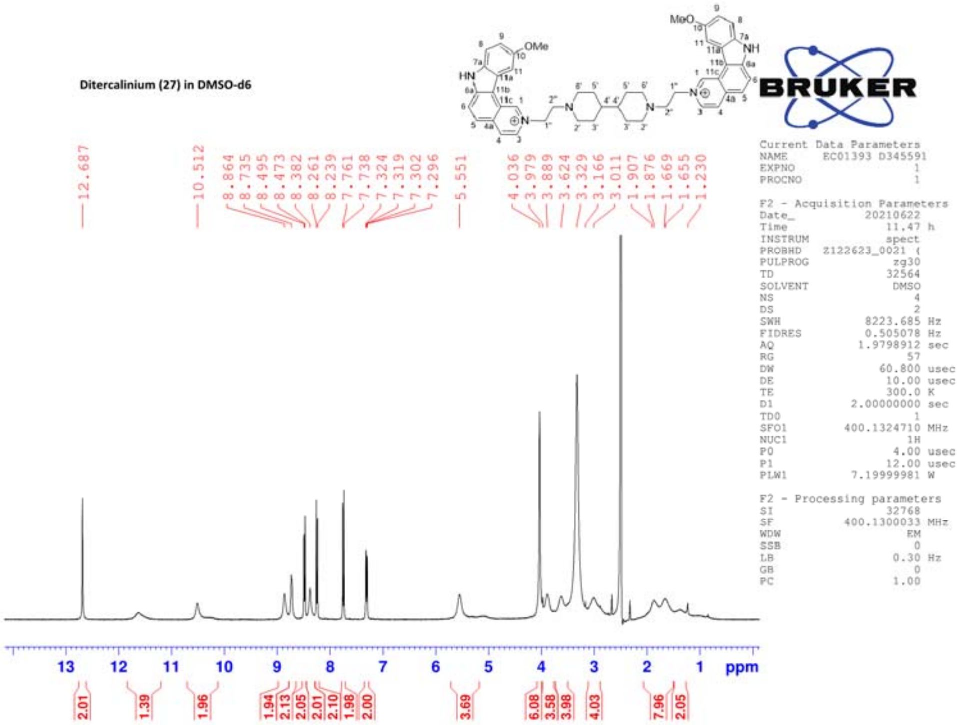

Figure S220. ^{1}H NMR spectrum of Ditercalinium (**29**).

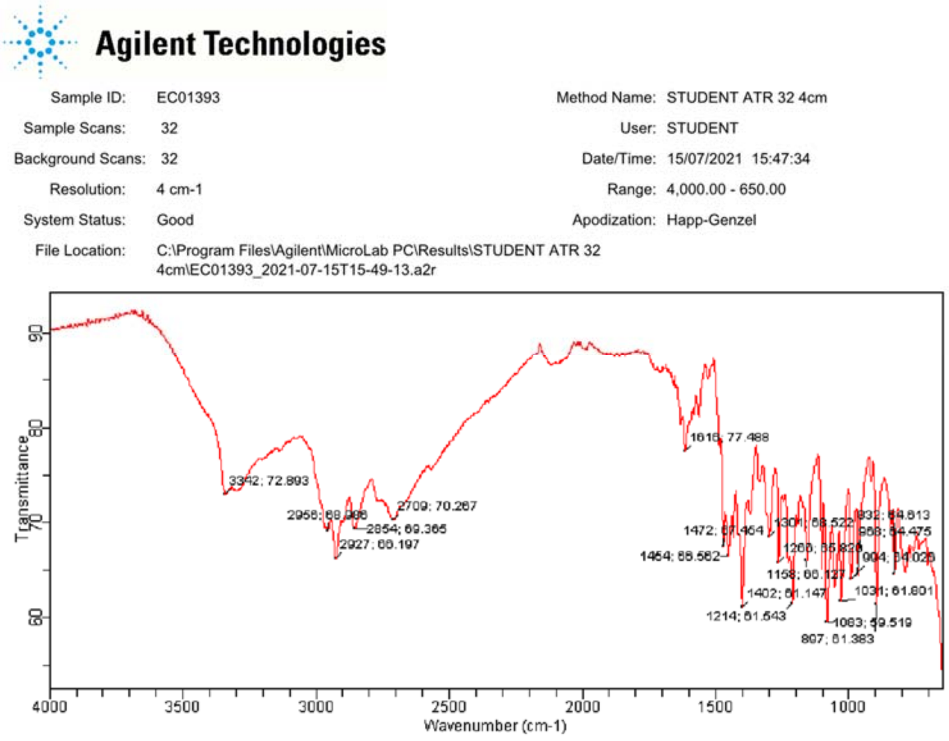

Figure S221. FT-IR spectrum of Ditercalinium (**29**).

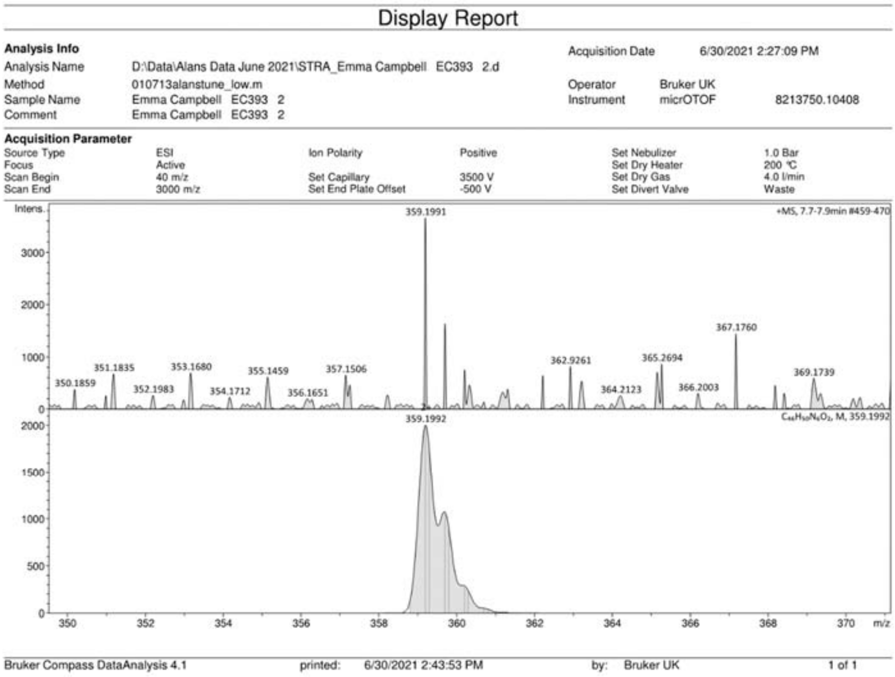

Figure S222. HRMS analysis of Ditercalinium (**29**).

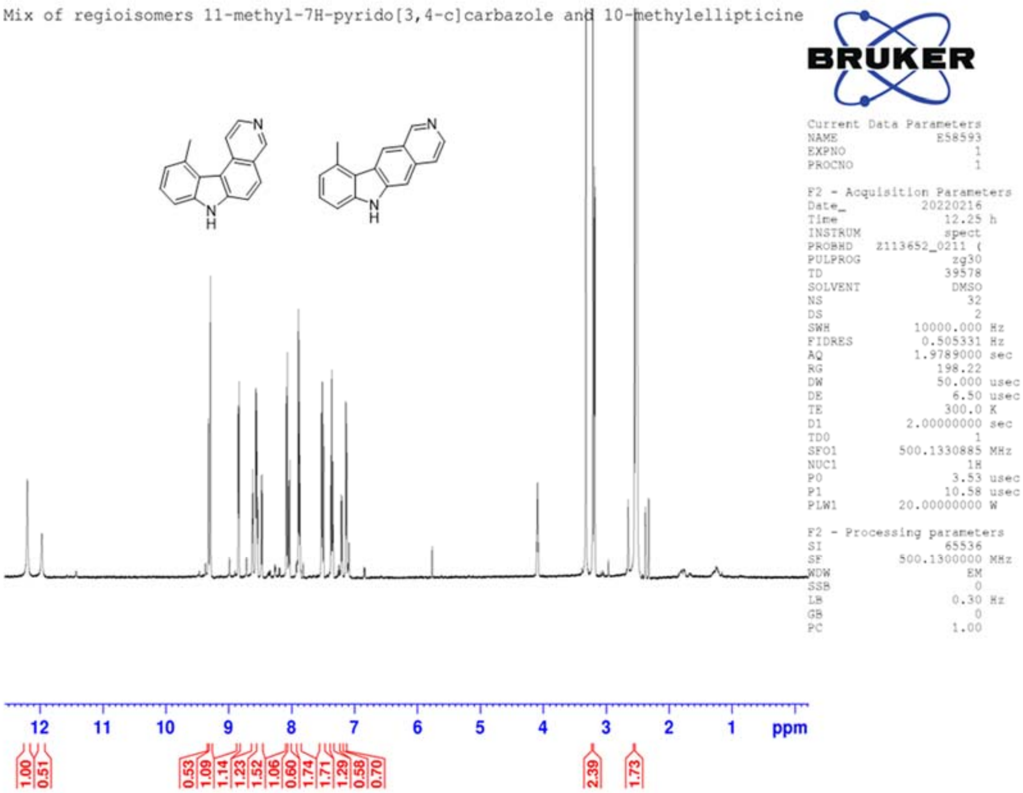

Figure S223. 1H NMR of the regioisomer mix (1:2) 11-methyl-7H-pyrido[3,4-c]carbazole and 10-methylellipticine.

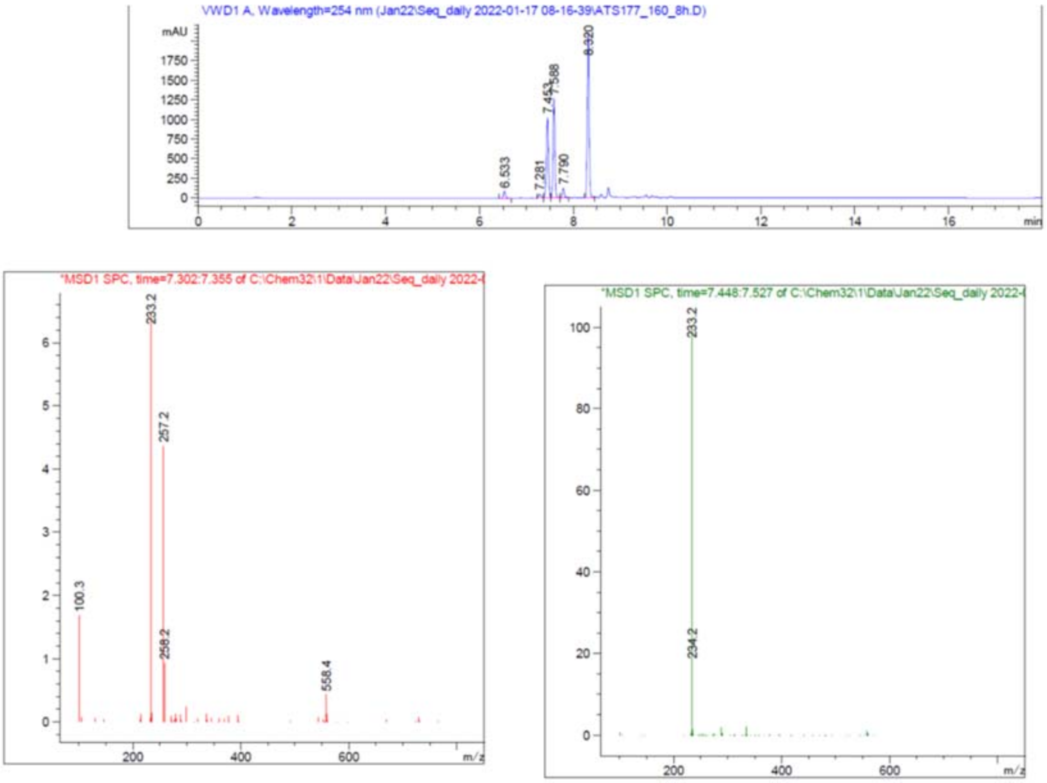

Figure S224 LCMS trace and ESI MS for the crude mixture of both regioisomers
